# Machine learning and network medicine approaches for drug repositioning for COVID-19

**DOI:** 10.1016/j.patter.2021.100396

**Published:** 2021-11-09

**Authors:** Suzana de Siqueira Santos, Mateo Torres, Diego Galeano, María del Mar Sánchez, Luca Cernuzzi, Alberto Paccanaro

**Affiliations:** 1 Escola de Matemática Aplicada, Fundação Getulio Vargas, Rio de Janeiro 22250-900, Brazil; 2 Department of Computer Science, Centre for Systems and Synthetic Biology, Royal Holloway, University of London, Egham Hill, Egham TW20 0EX, UK; 3 Facultad de Ingenieria, Universidad Nacional de Asunción, Luque 110948, Paraguay; 4 Universidad Católica “Nuestra Señora de la Asunción”, Asunción C.C. 1683, Paraguay; 5 COVID-19 International Research Team

**Keywords:** drug repurposing, COVID-19, SARS-CoV-2, non-negative matrix factorization, network medicine, kernels on graphs, graph visualization

## Abstract

We present two machine learning approaches for drug repurposing. While we have developed them for COVID-19, they are disease-agnostic. The two methodologies are complementary, targeting SARS-CoV-2 and host factors, respectively. Our first approach consists of a matrix factorization algorithm to rank broad-spectrum antivirals. Our second approach, based on network medicine, uses graph kernels to rank drugs according to the perturbation they induce on a subnetwork of the human interactome that is crucial for SARS-CoV-2 infection/replication. Our experiments show that our top predicted broad-spectrum antivirals include drugs indicated for compassionate use in COVID-19 patients; and that the ranking obtained by our kernel-based approach aligns with experimental data. Finally, we present the COVID-19 repositioning explorer (CoREx), an interactive online tool to explore the interplay between drugs and SARS-CoV-2 host proteins in the context of biological networks, protein function, drug clinical use, and Connectivity Map. CoREx is freely available at: https://paccanarolab.org/corex/.

## Highlights

• A matrix decomposition model for repurposing broad-spectrum antivirals
• A graph kernel approach to model perturbations induced by drugs on the interactome
• Graph kernels can integrate transcriptomics data to improve drug repurposing
• CoREx: a free online tool to formulate hypothesis for drug repurposing for COVID-19

## The bigger picture

The development timeline for treatments against emergent viral diseases can be significantly reduced by re-using drugs already available on the market—a concept known as drug repositioning.

We present two complementary machine learning approaches for drug repositioning that target SARS- CoV-2 and host factors, respectively. Our matrix decomposition approach exploits drug developmental information to predict the effectiveness of broad-spectrum antiviral drugs. Our graph kernel-based approach, rooted in ideas from network medicine, predicts which FDA-approved drugs are more likely to perturb the human subnetwork that is crucial for SARS-CoV-2 infection/replication. We also introduce CoREx, a freely available online tool that enables scientists to reason and formulate hypotheses about drug repurposing in the context of biological networks and pharmacological information.

While we have developed these methodologies for COVID-19, our approaches can be applied to any viral disease.

## Teaser

We present two complementary machine learning approaches for drug repositioning against COVID-19 that target SARS-CoV-2 and its cellular processes in the host, respectively. Our matrix decomposition approach exploits drug developmental information to predict broad-spectrum antivirals; our graph kernel-based approach, rooted in ideas from network medicine, predicts which FDA-approved drugs are more likely to perturb the human subnetwork that is crucial for SARS-CoV-2 infection/replication. We also introduce CoREx, a freely available online tool to reason and formulate hypothesis about drug repurposing in the context of biological networks and pharmacological information.

## Introduction

Drug discovery and development present several challenges, including high attrition rates, long development times, and substantial costs.^1^ Drug repositioning involves the use of de-risked compounds in humans, which translates into lower costs and shorter development times.^2^ Computational methods can assist drug repurposing research projects by providing rankings of drugs based on predicted therapeutic efficacy, as well as tools to help scientists reason about drug effectiveness by integrating diverse available biomedical knowledge.

Coronaviruses are notoriously difficult to manage, as there is no specific antiviral treatment that has been proven effective against the infections they induce.^3^ Identifying commercially available drugs with therapeutic effects for COVID-19 could provide early treatment options until effective therapies become widely available. A growing corpus of literature identifies several categories of treatment that revolves around the use of drugs with a mode of action that targets the molecular structure of the virus (*virally targeted agents*), or its cellular processes in the host (*host-targeted agents*), or those based on combinatorial therapies.^4^, ^5^, ^6^, ^7^

In this paper, we present two different machine learning approaches, and a webtool, for drug repurposing for COVID-19. Our first machine learning approach focuses on virally targeted agents and aims at ranking broad-spectrum antiviral (BSA) drugs. Given a small number of drugs associated with a virus, and their stage in the drug development process, our matrix decomposition algorithm assigns scores to a larger group of drugs with previously unknown associations with the virus. Our method predicts BSAs against SARS-CoV-2 by exploiting information about stages of drug development that are interpreted as probabilities of drug approval. To our knowledge, our matrix decomposition model is the first that integrates developmental-stage information to predict the efficacy of drugs against viral diseases, and we show that this is crucial to obtain better predictions.

Our second machine learning approach focuses on host-targeted agents, and prioritizes FDA-approved drugs based on ideas from network medicine.^8^ In particular, it exploits the concept of a disease module, which has been instrumental in the prediction of disease genes for hereditary diseases.^9^, ^10^, ^11^, ^12^ For a virus, a disease module can be defined as the set of human proteins (hereafter, host proteins) that interact with viral proteins, allowing the infection and replication processes. Recently, Gysi et al.^13^ have shown that, for SARS-CoV-2, most of the experimentally identified human host proteins^14^ form a distinct COVID-19 disease module in the interactome. Our network medicine-based approach is based on the idea that the binding of drugs to their protein targets causes a perturbation that propagates through the interactome. By quantifying this perturbation, it is possible to calculate the extent of the effect that a drug induces on the COVID-19 disease module. Our method ranks FDA-approved drugs based on this effect, which is estimated using graph kernels. An important aspect of our method is that it offers a natural way to model the relative importance of host proteins for the disease, and we show that our network medicine approach benefits from this prioritization of host proteins.

Finally, we present the COVID-19 repositioning explorer (CoREx), an online tool that enables scientists to analyze and reason about drug repurposing in a functional context on the interactome and thus allows the exploration of our results as well as the formulation of novel repurposing hypotheses. CoREx integrates several sources of information, connecting functional protein modules with drug targets and host proteins. CoREx also provides additional evidence for a drug of interest, such as whether the drug is on clinical trials for COVID-19, or whether the drug could reverse the gene expression signature of SARS-CoV-2 infection based on the Connectivity Map (CMAP).^15^^,^^16^

## Results

### A matrix decomposition model for antiviral discovery

Recently, Andersen et al.^17^ published a dataset containing 850 associations between 126 BSA drugs and 80 viruses for which they have been approved or are under development. Importantly, each drug-virus association was manually curated and is annotated with its stage in the drug development process.

**Figure 1:**
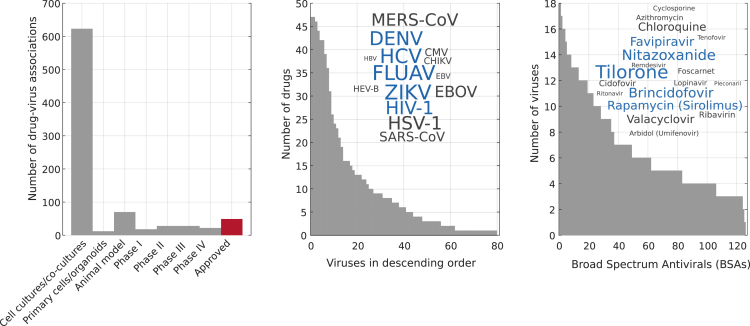
Drug-virus dataset statistics We used the dataset manually curated by Andersen et al.^17^ (Left) Number of drug-virus associations grouped by their known developmental status. The development of broad-spectrum antivirals (BSA) starts with *in vitro* experiments (e.g., cell culture), moves to animal models, and then to clinical trials in humans (phases I–IV). It terminates with the approval of the drug for commercial use (in red). (Middle) Number of drugs (BSAs) associated to each virus in the dataset. Inset: the word cloud shows the 14 viruses with most associations. The size of the word is proportional to its number of associations and the five most popular viruses among drugs are colored blue. (Right) Number of viruses associated to each drug in the dataset. Inset: the word cloud shows the 18 drugs with most associations and the five most popular drugs among viruses are colored blue.

Figure 1 shows the number of drug-virus associations that corresponds to each developmental stage, as well as histograms of the associations grouped per drug and per virus. We notice that the associations are not uniformly distributed for viruses or drugs (Figure 1, left and right panels). This type of long-tailed distribution of entries has been previously observed in datasets that appear in the recommender system literature, such as Netflix or Movielens,^18^ and we have recently exploited this property to build a recommender system based on matrix factorization for predicting drug side effect frequencies.^19^

Various types of recommender systems have recently been developed for different settings of the drug repositioning problem. A few methods are based on variations of the non-negative matrix factorization (NMF) algorithm,^20^^,^^21^ such as the NMF with L2 regularization by Bakal et al.,^22^ the TriFactor NMF by Ceddia et al.,^23^ and the indicator-regularized non-negative matrix factorization (IRNMF) method by Tang et al.,^24^ which was developed to repurpose drugs for COVID-19. Our aim is also to build a recommender system that recommends BSA drugs to viruses and the novelty of our approach lies in the realization that the stages of drug development for drug-virus associations can be related to the probability of reaching the final stage of drug development (hereafter, probability of success). This observation is motivated by the empirical evidence (e.g., Dowden and Munro^25^) that the probability of success of a candidate drug increases as the candidate drug moves to the next developmental stage in the drug development process. This led us to develop a novel objective function that models the probabilities of success of drug-virus associations using their stage in the drug development process. In this paper, we show how the integration of this type of information greatly improves prediction performance.

In recommender systems based on matrix decomposition, the fundamental assumption is that users and movies can be represented as latent feature vectors in a low-dimensional space, and that a rating value for a specific user-movie pair is obtained by the dot product of the corresponding feature vectors. In our context, each drug and each virus can be represented as low-dimensional feature vectors in a latent space such that the dot product between the vectors model effective drug-virus associations. Having collected all the associations in a binary matrix *Y*, where each entry $y_{ij}=1$ if and only if drug *i* is associated to virus *j* in the Andersen et al.^17^ dataset ($y_{ij}=0$ otherwise), for each drug *i* we learn a low-dimensional feature vector $p_{i}\in R^{k}$ (the *drug signature*) and for each virus *j* a low-dimensional feature vector $q_{j}\in R^{k}$ (the *virus signature*), such that $y_{ij}\approx p_{i}^{T}q_{j}$. Therefore, our algorithm amounts to decomposing the n×m matrix *Y* into the product of two matrices $P\in R^{n\times k}$ in which each row is a drug signature $p_{i}^{T}$, and $Q\in R^{k\times m}$ in which each column is a virus signature $q_{j}$, and k≪min(n,m). Indicating their product with $\hat{Y}$, we have $Y\approx PQ=\hat{Y}$. Matrices *P* and *Q* are learned by minimizing the following cost function:

(Equation 1) $$\left\{ \begin{array}{c} \underset{P,Q}{\text{min}}L\left(P,Q\right)=\underset{\text{approved},\text{ phase IV}}{\underset{︸}{\frac{1}{2}{\left||M^{A}\circ \left(Y-PQ\right)|\right|}_{F}^{2}}}+\underset{\text{In vitro},\text{ animal model},\text{ clinical trials}}{\underset{︸}{\frac{1}{2}\sum_{s\in \left\{B,C,D,E\right\}}\alpha_{s}{\left\|M^{s}\circ \left(Y-PQ\right)\right\|}_{F}^{2}}}+\underset{\text{zero}-\text{driven regularisation}}{\underset{︸}{\frac{\alpha_{z}}{2}{\left||M^{z}\circ \left(PQ\right)|\right|}_{F}^{2}}}\\ \text{subject to non}-\text{negative constraints}\;P,Q\geq 0, \end{array}\right.$$

where $∥.{∥}_{F}$ is the Frobenius norm of a matrix, ∘ is the element-wise (Hadamard) product, and the letters A,B,C,D,E indicate disjoint subsets of entries in *Y* that are defined according to the known developmental stages of drug-virus associations, as explained below. Let us now analyze Equation 1 to understand how the information about drug developmental stages is integrated into our system to model probabilities of success of drug-virus associations.

During learning, the drug-virus associations are divided into groups according to their stage of development. The first term in Equation 1 is the fitting constraint on the approved and phase IV drug-virus associations (set *A*). Matrix $M^{A}$ is used to apply the summation only to entries in *Y* belonging to the set of approved associations *A*, being defined as: $M_{ij}^{A}=1$ if drug *i* was approved or is in phase IV for virus *j*, or 0 otherwise. Thus, the first term of Equation 1 is attempting to find a decomposition PQ to reconstruct the associations in set *A* exactly. The second term in Equation 1 has an equivalent role for the remaining known associations in *Y*, corresponding to earlier stages in the drug development process—sets *B*, *C*, and *D* contain entries in clinical trials phases I, II, and III, respectively, while set *E* contains associations in *in vitro* and animal model stages. Here the corresponding $M^{s}$ matrices are used to apply the summations only to entries belonging to the corresponding sets ($M_{ij}^{s}=1$ if the entry $y_{i,j}$ belongs to set *s*). However, for these sets, their contributions to the loss are weighted differently using the parameters $\alpha_{s}\in \left[0,1\right]$. These parameters have the key role of downweighting these terms in the minimization, in a way that reflects their higher uncertainty of success due to their earlier stage of drug development, thus effectively coding probabilities of success for each subset. Similarly, the third term in Equation 1 is used to downweight the importance of the zero entries of *Y* while also serving as a regularization term.^19^ Finally, we impose non-negative constraints on *P* and *Q* to favor the interpretability of the learned representations.^19^^,^^20^

Thus, our model is closely related to NMF.^20^ Both models seek to decompose a data matrix *Y* into the product of two non-negative matrices *P* and *Q*. However, the NMF model considers all the entries in *Y* equally during the learning—this works well when entries have the same meaning, e.g., pixels in an image.^20^ Instead, in our approach, we assign different levels of importance to subsets of entries to reflect the drug stages of development, thus coding the probability of drug success, which is what we are trying to predict. This gives rise to a loss function in Equation 1 that is different from NMF. Finally, notice that, in our model, if we set the values of all the α parameters to 1—which amounts to discarding the role of probabilities of success—we obtain the original NMF model.

**Figure 2:**
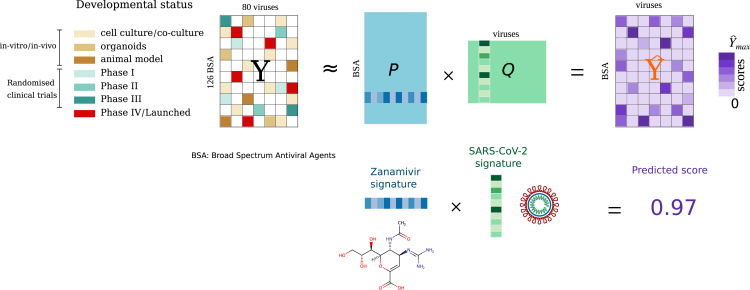
Overview of our matrix decomposition model for predicting effective drug-virus associations Totals of 850 associations for n=126 different BSAs and m=80 distinct viruses were collected from the Andersen et al.^17^ database. The observed associations were arranged into an n×m matrix *Y* by setting $y_{ij}=1$. Unobserved associations were encoded with zeros. Our algorithm decomposes the matrix *Y* into the product of two matrices, *P* (of size n×k) and *Q* (of size k×m). By multiplying the matrices *P* and *Q*, we obtain $\hat{Y}$, which models *Y*, where all the entries are replaced with real numbers—these correspond to our predicted scores. Rows of *P* are the BSA feature vectors (or BSA signature); columns of *Q* are the virus feature vectors (virus signature). The lower illustration depicts how our model discovers a low-dimensional signature vector for the antiviral drug zanamivir, and a low-dimensional signature vector for SARS-CoV-2. The dot product of these two signatures is the predicted efficacy of zanamivir against SARS-CoV-2.

An overview of our matrix decomposition model is illustrated in Figure 2. Our starting point is the matrix *Y* containing binary drug-virus associations. We learn the matrices *P* and *Q*, which minimize the loss function in Equation 1, by employing an iterative algorithm that uses a simple multiplicative update rule (see the Experimental procedures). Our algorithm, inspired by the diagonally rescaled principle of NMF,^20^ is fast, it does not require setting a learning rate or applying a projection function and it satisfies the Karush-Kuhn-Tucker (KKT) complementary conditions of convergence (see the Experimental procedures). Having learned *P* and *Q* such that Y≈PQ, we calculate the matrix $\hat{Y}=PQ$. Note that, while *Y* contains binary entries, $\hat{Y}$ contains real positive numbers that are our predicted scores.

#### Predicting effective BSA drugs against viruses

To perform an *in silico* evaluation of the performance of our model, we formulated a matrix completion task under a leave-one-out cross-validation (LOOCV) procedure using the 49 BSA drugs that have been approved for use, and the 22 that reached phase IV of clinical trials for 28 viruses. To prevent overfitting and biases during hyperparameter tuning, we performed a different LOOCV by using clinical trials associations from phases I, II, and III to set the model parameters. Our final model parameters were: $k=5,\alpha_{B}=0.16,\alpha_{C}=0.27,\alpha_{D}=0.71,\alpha_{E}=0.01$, and $\alpha_{z}=2$ (see the Experimental procedures).

We compared the performance of our algorithm with the other drug-repurposing approaches that we mentioned earlier, namely the NMF with L2 regularization,^22^ the TriFactor NMF,^23^^,^^26^ and the IRNMF,^24^ which was also developed for COVID-19. Moreover, we also included standard NMF and truncated singular value decomposition (tSVD)^18^ as baselines. The relation between previous NMF-based drug repositioning methods and our model is explained in Note S1.

Following other works that used LOOCV evaluations,^9^^,^^27^^,^^28^ we evaluated the performance at predicting one drug at a time, measuring how often that drug was found within the first 1, 5, 10, 15, 20, 25, and 30 drugs predicted by the different algorithms. Here, it is important to remind ourselves that our model takes as input an incomplete sparse drug-virus matrix, with only 8.43% non-zero entries, and outputs predicted scores for all the entries in the matrix. In the evaluation presented here, we focus on validating predictions corresponding to the interesting case where drug-virus associations are not yet under development (see Note S2 for the case of predicting drugs already under development, but not approved, for specific viruses). Therefore, in our LOOCV procedure, one drug-virus association (approved or phase IV) was removed at a time from the drug-virus matrix *Y* (by setting the corresponding entry to zero). We then trained the model, and scores were predicted for all drugs. Finally, we ranked drugs that had no known association with that virus and checked the percentage of cases in which the correct (effective) drug for the virus was found among the top *K* predictions.

**Figure 3:**
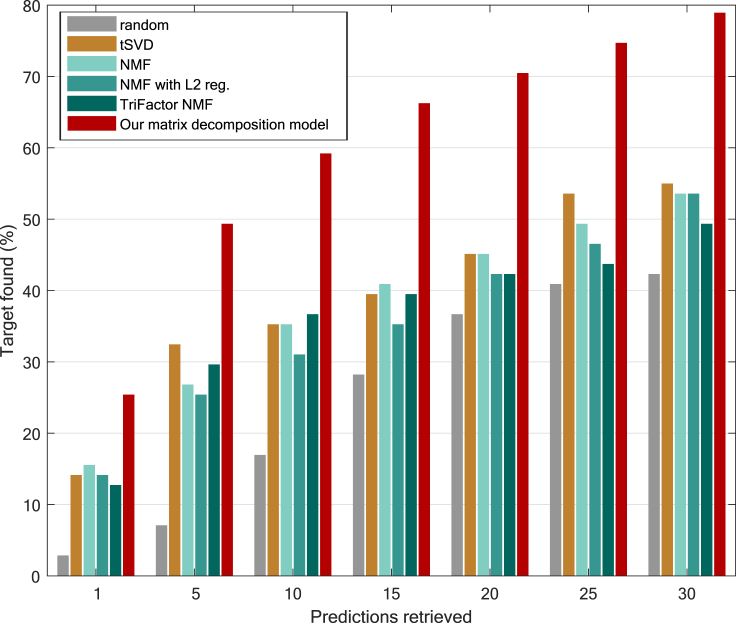
Performance at predicting approved/phase IV BSAs for 28 viruses Percentage of approved or phase IV BSA drugs found for a specific virus in the top *K* predictions retrieved. The performance of our method is compared with different matrix decomposition algorithms in a leave-one-out fashion. NMF, non-negative matrix factorization; tSVD, truncated singular value decomposition. A baseline based on random scores sampled from a uniform distribution is also included.

Figure 3 shows the performance of the methods at predicting effective (approved/phase IV) BSA drugs against specific viruses. Our model outperforms the competitors for each number of predictions retrieved: by 9.8%–22.5% in the top 1, by 16.9%–42.2% in the top 5, by 22.5%–42.2% in the top 10, and by 25.3%–38% in the top 20. Overall, our method could recover 70% of the phase IV/approved BSA drugs for 28 distinct viruses in the top 20 predictions. We also observed that, in some cases, tSVD and TriFactor NMF perform slightly better than NMF. The comparison of our method’s performance with IRNMF was performed in a smaller subset of the matrix *Y* (see Figure S1 in Note S1).

The good prediction performance of our model prompted us to ask how much of the prediction power could be attributed to the integration of developmental stages information in our cost function (see Equation 1). Our model significantly improves performance over two control baselines that: (1) randomize developmental stage information in the training set and (2) remove developmental stage information from our cost function (see a detailed discussion in Note S3).

The analysis of our predictions for SARS-CoV-2 is presented in detail in the “Evaluation” section, together with the results of our network medicine approach.

### Repositioning FDA-approved drugs with network medicine

The majority of BSAs considered previously target viral proteins. In our work, we also explored approaches that consider drugs targeting human proteins. Human proteins interact with each other, forming a protein-protein interaction (PPI) network. This and other biological networks have been explored in relation to disease—this area of research has often been called network medicine. It has been shown that proteins associated with specific hereditary diseases tend to cluster in neighborhoods of the interactome (the disease module),^8^^,^^29^^,^^30^ and successful applications of molecular network analysis have been reported for the identification of disease genes,^9^ drug development,^10^ and drug efficacy prediction.^29^

The use of network medicine for assisting drug repositioning was originally applied to genetic diseases.^29^ A drug induces its effects on a human PPI subnetwork by binding to its target proteins,^31^^,^^32^ and this causes a perturbation in the interactome that is then propagated. Thus, drug efficacy for a genetic disease can be associated to how likely the drug is to affect its disease module through the perturbations propagated in the human PPI network.^29^ To implement this idea, Guney et al.^29^ proposed a distance (hereafter, the Guney distance) based on the shortest path length between the disease module and the drug targets.

**Figure 4:**
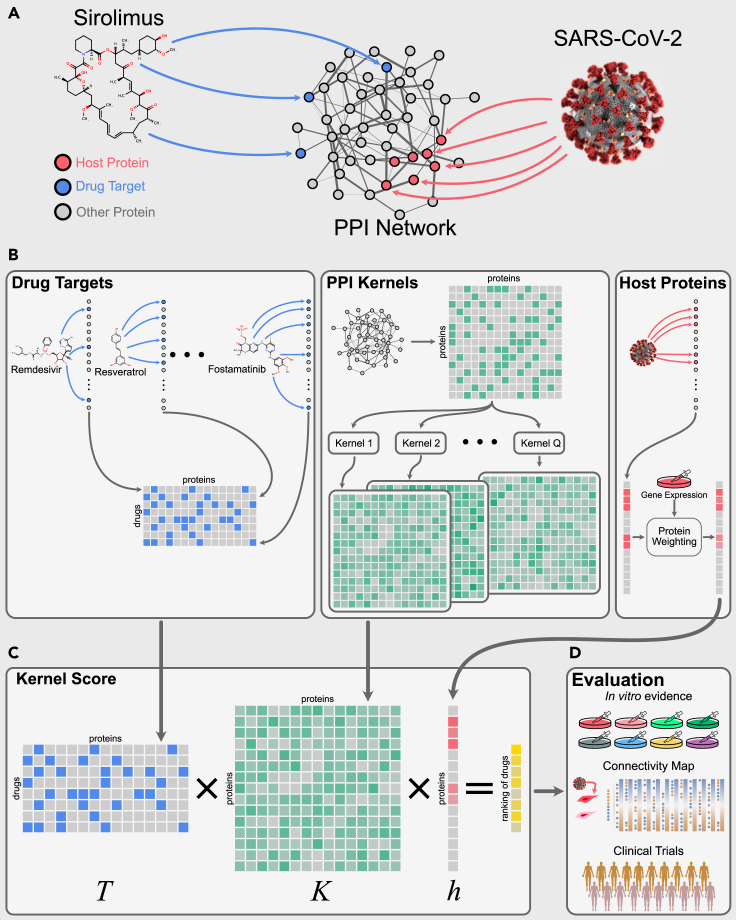
Overview of our network medicine approach (A) The human interactome containing both host proteins (red) and drug targets (blue). (B) The totals of 14,941 drug target associations between *N =* 2,197 FDA-approved drugs and *n*_*V*_ = 18,505 proteins are represented by a binary matrix *T* (blue matrix). Multiple graph kernels are calculated on the interactome, resulting in $n_{V}\times n_{V}$ matrices (green matrices). The host proteins are represented by a vector *h* of size $n_{V}$ (red vector) indicating their weights (based on gene expression data). (C) Our kernel score is calculated using a matrix multiplication to obtain a prediction score for each drug. (D) The obtained ranking is evaluated using different types of evidence: *in vitro* efficacy against SARS-CoV-2, Connectivity Map, and clinical trials.

Recent studies suggest that an analogous approach can be useful for infectious diseases such as COVID-19.^13^^,^^33^ Viruses hijack host proteins to facilitate their replication, and hence the inhibition or knockdown of such host proteins can block viral replication.^34^ Gysi et al.^13^ have shown that, for SARS-CoV-2, most of the experimentally identified host proteins^14^ group together in a large connected component, forming a COVID-19 disease module, as illustrated in Figure 4A with red nodes (host protein subnetwork). Therefore, the idea here is to find drugs that, by binding to their targets (blue nodes in Figure 4A), are likely to perturb this module.

We can think of the perturbation caused by a drug as a process in which the effect of the drug *diffuses* on the PPI network starting from its targets. Thus, our drug repurposing problem translates into the problem of the diffusion between drug targets and the set of host proteins. Gysi et al.^13^ implemented this idea for COVID-19 by using the diffusion state distance (DSD).^35^

Kernels on graphs are appealing for modeling a diffusion process on a network. They are theoretically well founded in statistical learning theory,^36^^,^^37^ and have shown good empirical results in many applications.^35^^,^^38^^,^^39^ Graph kernels can be interpreted as measures of similarity between nodes in a network. There are different types of kernels. The *p*-step random walk kernel, for example, is directly associated to the number of times a random walker starting from a node *i* visits a node *j* after *p* steps.^36^ Another example is the diffusion kernel (or heat kernel), which can be thought of as a random walk with an infinite number of infinitesimally small steps. An alternative interpretation is that this kernel corresponds to the amount of heat that reaches a node *j* after diffusing an initial heat from node *i*.^36^

Importantly, kernels on graphs can be applied in a natural way to nodes with weights. This property can be particularly useful for our problem: we can assign weights to the host proteins to model the different roles that they have for the infection/replication of the virus. For example, it has been shown that the ACE2 protein receptor is the viral entry factor of SARS-CoV-2.^40^ Another study based on gene expression experiments on infected SARS-CoV-2 cell lines suggests that certain protein-coding genes play a key role during the infection process.^41^ Then, the amount of change in gene expression after SARS-CoV-2 infection may be associated with the level of importance of the protein for the infection. In addition, perturbing host proteins whose expression levels change the most may be important for reverting the effect that the infection causes in gene expression. Predicting drugs that might revert this effect has been shown to be a good strategy for assisting in the discovery of effective small molecules for different diseases.^15^^,^^42^

To assist the repositioning of drugs for COVID-19, we used five different kernels on graphs and weighted the host proteins with differential gene expression data (absolute value of the log fold change between the gene expression levels of COVID-19 patients, and controls—see experimental procedures for details on the RNA-seq data). We used the interactome assembled by Gysi et al.,^13^ and a set of 336 human proteins that were identified as hosts of SARS-CoV-2 (see experimental procedures). Every FDA-approved drug with known targets in this interactome was ranked by each of the kernels in our approach (see experimental procedures). The selected kernels are defined in terms of the graph Laplacian (see experimental procedures), as shown in Table 1. For each drug, we obtain the graph kernel-based similarities between each of its targets and each of the host proteins. The final score of a drug is the sum of these similarities weighted by the amount of change in the host protein expression levels after infection. Drug scores are then ordered, obtaining a drug ranking which is evaluated. We also calculated an aggregated ranking, which we called *avgRank*, where the ordinal position of each drug was obtained by simply averaging the ranking position that the drug had obtained in each of the kernels.

**Table 1:** Graph kernels

Kernel	Formula
*p*-step random walk	$K={\left(aI-\tilde{L}\right)}^{p}$
Diffusion process	$K=\text{exp}\left(-\sigma^{2}/2\tilde{L}\right)$
Regularized Laplacian	$K={\left(I+\sigma^{2}\tilde{L}\right)}^{-1}$
Commute time kernel	$K=L^{+}$
Inverse cosine	$\text{cos}\tilde{L}\pi /4$

Definition of graph kernels based on the normalized Laplacian ($\tilde{L}$), and pseudoinverse of the Laplacian ($L^{+}$), where *a*, *p*, and σ are given parameters.

The mathematical formulation of this approach turns out to be quite simple. Let $n_{V}$ the number of proteins in the PPI network, *N* the number of FDA-approved drugs, and *T* an $N\times n_{V}$ matrix of drug target associations, where $T_{ij}=1$ if *j* is a target of drug *i*, and 0 otherwise (see drug targets box in Figure 4B). Let *K* be a square matrix of dimensions $n_{V}\times n_{V}$ representing kernel-based similarities between proteins on the PPI network (see PPI kernels box in Figure 4B). Let *h* be an $n_{V}$-dimensional column vector containing weights related to the differential expression data of the host proteins and zeros for the remaining proteins (see host proteins box in Figure 4B). We obtain prediction scores simultaneously for all drugs with the following matrix multiplication $S_{d}=TKh$ (also illustrated in Figure 4C), resulting in a vector of drug scores, $S_{d}$.

### Evaluation

To evaluate the performance of our methods, we used three different sources of evidence from ongoing research: *in vitro* experiments, clinical trials, and CMAP scores. These sources are independent of each other; hence they can be used to provide an independent evaluation of the efficacy of repurposing methods. Note that none of these three sources of evidence can be considered a gold standard, as none of them can ensure therapeutic effects for COVID-19 patients. Yet, they represent a proxy of effectiveness of drugs for COVID-19.

*In vitro* experiments involving drugs with antiviral efficacy indicate their potential to be effective at reducing viral infection and replication in the host cell. Evaluating our models with this kind of evidence allows us to assess whether they prioritize drugs with molecular antiviral efficacy versus other drugs.

Clinical trial studies are used to assess pharmacokinetics, dosage, therapeutic efficacy, and safety of drugs.^43^ Each phase in clinical trials involves an increasing number of patients, thus achieving higher statistical significance while minimizing the number of patients that risk developing side effects.^44^ Indicating a drug in a clinical trial requires satisfying several conditions set by biologists and medics, and arguments of why it might be effective. This suggests the investigators believe that the drug is safe and a potential candidate to treat the disease. Evaluating our models with clinical trial evidence allows us to determine if they prioritize drugs that would be included in such trials.

We use the CMAP^15^^,^^16^ to contrast changes in gene expression levels caused by a drug (drug expression profile) with changes induced by SARS-CoV-2 infection (disease expression profile). The hypothesis is that, if a drug expression profile is opposite to a disease expression profile, then it could potentially “revert” the disease signature and have therapeutic effects—this idea has already been used before^15^^,^^42^ to predict new therapeutic indications for drugs and has also been applied to COVID-19.^45^^,^^46^ Therefore, evaluating our models with this source of evidence allows us to assess whether they prioritize drugs with potentially therapeutic effects.

For the matrix decomposition approach, the evaluation was carried out using the 126 BSAs in the drug-virus dataset.^17^ For the network medicine approach, the evaluation was done on an interactome of 18,505 proteins with 327,924 interactions.^13^ With this approach we ranked 2,197 approved drugs from DrugBank.^47^

We used the types of evidence described above to create three datasets where drugs were classified as either effective or non-effective for COVID-19 (see experimental procedures). This allowed us to assess the performance of a prediction method by formulating a binary classification problem, where the task is to discriminate the two sets of drugs, and then calculating binary classification metrics based on the analysis of the confusion matrix.

**Figure 5:**
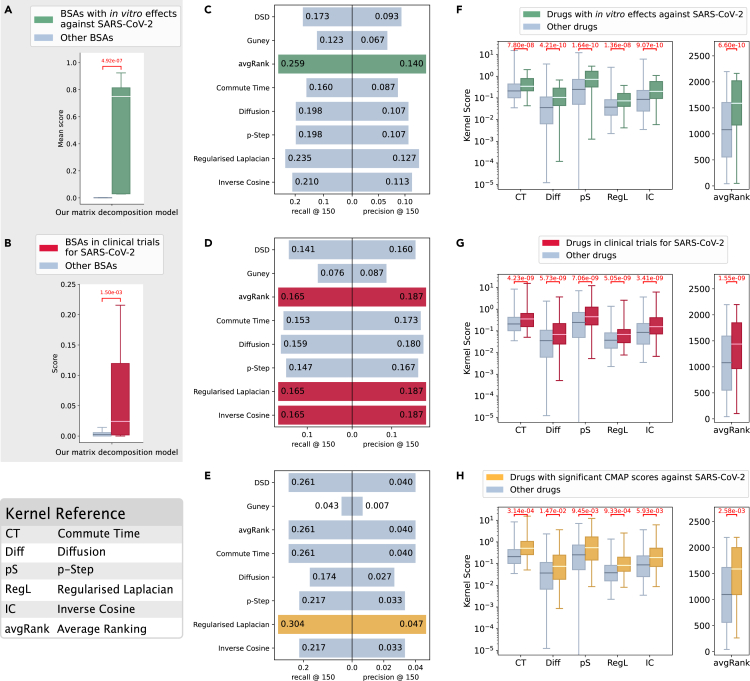
Analysis of the predictions for COVID-19 We used three different sources of evidence: *in vitro* (A, C, and F), clinical trials (B, D, and G), and CMAP (E and H). We compared scores for drugs with evidence of efficacy against SARS-CoV2 versus scores for the remaining drugs. Our matrix factorization model (A and B) and kernel-based methods (F, G, and H) provide scores that are significantly different between the two groups of drugs in every case (Wilcoxon-Mann-Whitney p < 0.05). We formulated a binary classification problem to discriminate between drugs with evidence of efficacy against SARS-CoV2 and the remaining drugs. (C, D, and F) Comparison of precision and recall at top 150 for our kernel-based methods (commute time, diffusion, *p*-step, regularized Laplacian, and inverse cosine kernels, and avgRank), DSD, and Guney’s distance. The highest values are colored.

However, we note that the lack of a set of drugs with proven therapeutic effect against COVID-19 (i.e., a gold standard), poses a challenge for this type of evaluation—this problem has also been described before, e.g., in Zhou et al.^48^ and Gysi et al.^13^ We hypothesized that drugs with evidence against COVID-19 should behave differently from the remaining drugs. This hypothesis has an actionable consequence: a method can be evaluated by assessing whether it can discriminate between the two groups of drugs (effective and non-effective)—if it can, this is an indication that we can possibly trust the predictions it makes. Therefore, together with traditional metrics for binary classification, we also assessed whether prediction methods provided scores that were statistically different for the two classes of drugs. Our results (Figures 5A, 5B, and 5F–5H) show that the differences between the scores are significant for our matrix decomposition approach as well as our kernel methods across several evaluation settings. We observe that other network-based methods do not pass this test with such consistency (see Notes S5–S7). In the following, we present the results for each type of evidence, separately.

#### *In vitro* evaluation

Of the 126 BSAs in the drug-virus dataset, 10 have shown *in vitro* efficacy against SARS-CoV-2.^13^^,^^49^ In our evaluation, these drugs were removed one at a time from the drug-virus matrix *Y* (by setting the corresponding entry to zero). We then trained our matrix decomposition model, and scores were predicted for all the drugs. We used the Wilcoxon-Mann-Whitney p value to assess the difference between the scores obtained for those 10 drugs and the rest of the drugs. Figure 5A shows that our matrix decomposition method significantly assigns higher scores to BSAs with *in vitro* efficacy (Wilcoxon-Mann-Whitney p value = 4.92 × 10^−7^). Precision and recall are shown in Figure S2 (Note S1).

Scores predicted by the kernel-based methods are shown in Figure 5F. Of the 2,197 FDA-approved drugs considered by our network medicine approach, 81 have shown *in vitro* efficacy against SARS-CoV-2.^13^^,^^49^ We observed that the scores of drugs with *in vitro* efficacy against SARS-CoV-2 are significantly higher than those of the remaining drugs for all kernels and the average ranking (avgRank).

In Figure 5C, we show that the kernel-based methods performed better than the competitors for the *in vitro* evaluation. The recall@150 of the average ranking is 49.71% higher than DSD, and 110.57% higher than the Guney distance. The precision@150 of the average ranking is 50.54% higher than DSD, and 108.96% higher than the Guney distance.

#### Clinical trial evaluation

Of the 126 BSAs in the drug-virus dataset, 28 are in clinical trials (see experimental procedures). Figure 5C shows that prediction scores by our matrix decomposition method are significantly higher for drugs in clinical trials (Wilcoxon-Mann-Whitney p value = 1.5 × 10^−3^). Our method can recover 50% of the correct BSAs in the top-20 predictions retrieved (see Figure S2 in Note S1).

Scores predicted by the kernel-based methods are shown in Figure 5G. Of the 2,197 FDA-approved drugs considered by our network medicine approach, 170 are in clinical trials. We observed that the scores of drugs in clinical trials for COVID-19 are significantly higher than those of the remaining drugs for all kernels and the average ranking (avgRank).

In Figure 5D, we show that the kernel-based methods performed better than the competitors for the clinical trials evaluation. The recall@150 of the average ranking is 17.02% higher than DSD, and 117.11% higher than the Guney distance. The precision@150 of the average ranking is 16.88% higher than DSD, and 114.94% higher than the Guney distance.

#### CMAP evaluation

We queried CMAP^15^^,^^16^ obtaining a list of 23 FDA-approved drugs that present an expression profile opposite to the one expressed by SARS-CoV-2 infected cells with a τ score between −90 and −100 (see experimental procedures). Figure 5H shows that the scores of FDA-approved drugs with strongly negative CMAP correlation are significantly higher than those of the remaining drugs for all kernels and the average ranking (avgRank).

In Figure 5E, we compared the performance of the kernel-based methods and competitors for the CMAP evaluation. Our average ranking has the same performance as DSD and better performance than Guney’s distance (recall@150 is 50.72% higher, and precision@150 is 47.43% higher). The regularized Laplacian kernel had the best performance, with recall@150 16.48% higher than DSD, and 60.7% higher than Guney’s distance, and precision@150 17.5% higher than DSD, and 57.14% higher than Guney’s distance.

##### On the importance of integrating transcriptomics data

An interesting question is whether weighting host proteins by differential expression improves our network medicine approach. To answer this, we compared results based on weighted host proteins and unweighted/binary host proteins. For *in vitro* and clinical trials evidence, we observed that the Wilcoxon-Mann-Whitney p values are smaller (more significant) when using weighted host proteins when compared with considering all host proteins equally. The recall@150 and precision@150 are consistently higher when we use weights for the three types of evidence. These results are presented in Note S8.

##### Our results hold for different PPI networks and evaluation settings

An important question is whether results are consistent across different interactomes and how sensitive they are to different choices of the PPI network. We re-computed the kernel-based scores using the recently released HuRI PPI^50^ as well as the interactome compiled by Cheng et al.^30^ Results are presented in Note S7. For most of the kernels, FDA-approved drugs with *in vitro*, and clinical trials evidence have a significantly higher prediction score than the remaining drugs. For the three sources of evidence, the kernel-based methods have the higher recall@150 and precision@150 when compared with competitors. This indicates that our results have a high consistency across different interactomes.

##### Comparison with the approaches by Gysi et al.

We also extensively compared our kernel methods with the methods recently proposed by Gysi et al.,^13^ although the comparison could only be carried on the Gysi et al. dataset—this consists of 918 drugs including approved, investigational, experimental, nutraceutical, and withdrawn drugs. Overall, our kernel methods perform better with respect to *in vitro* and CMAP evidence—note that, in several cases, the scores obtained by the Gysi et al. methods for sets of effective and ineffective drugs are not significantly different. GNN methods perform better than kernel methods only with respect to clinical trial evidence. A summary of the different datasets used can be found in Note S4. A detailed description of all the experiments comparing our approaches with those from Gysi et al. is presented in Note S6.

### CoREx

As a further way to evaluate drug repurposing against SARS-CoV-2, we developed CoREx, a web-based tool that enables scientists to study drug repurposing in a functional context on the interactome. Given a set of drug targets, CoREx offers the users a panoramic point of view that puts together several biologically relevant contexts (i.e., functional relationships, PPIs, clinical trial status, CMAP scores, and drug’s anatomical therapeutic chemical [ATC] categories). Our goal is to assist researchers to reason about drug alternatives, drug combinations, and mechanisms of actions by analyzing the interplay between drug targets and host proteins in these different contexts.

**Figure 6:**
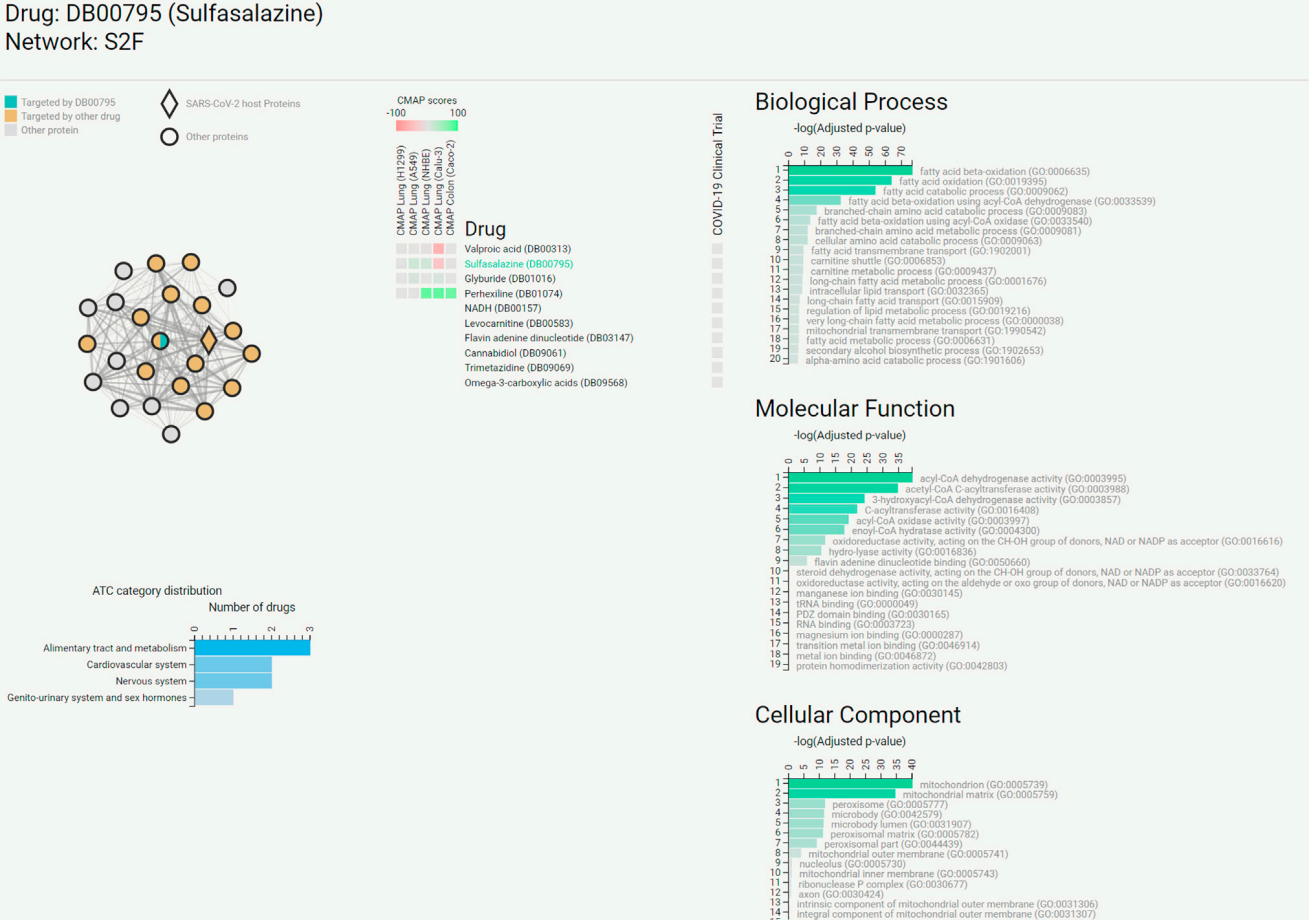
Screenshot of CoREx displaying a functional module for Sulfasalazine (highlighted in green in the “Drug” list) The module is depicted as a network on the top left where nodes represent proteins, edges represent shared functional characteristics, and the thickness of the edges represents the strength of such functional similarity. Host proteins are depicted as diamonds, drug targets are colored. The list of drugs with at least one target in this functional module is presented in the center, alongside CMAP scores for five cell lines (on the left), and an indicator of whether the drug is currently in clinical trials (on the right). The bar plots on the right part correspond to the functional enrichment scores for each GO domain. The bar plot on the bottom left section of the image summarizes the ATC categories of the drugs targeting this functional module.

Centered around ideas from network medicine, CoREx provides two different tools: the *functional analysis tool* and *interactome analysis tool*. The functional analysis tool allows the user to study the relationships between drug targets and host proteins. A functional interactome is built by integrating protein networks available in the STRING database^51^ in a way that maximizes the probability that two interacting proteins share functional characteristics (see Note S10 for details on the network combination). Then, we use the ClusterONE algorithm^52^ to identify functionally similar groups of proteins, and filter those that contain at least one SARS-CoV-2 host protein, and at least one drug target. The functional enrichment of these groups is then analyzed using Enrichr.^53^ All the drugs that interact with the module through their targets are enriched with their ATC categories, CMAP evidence, and clinical trial status against COVID-19. All of these results are presented to the user in a user-friendly interactive graphical interface, as shown in Figure 6.

The interactome analysis tool allows the user to visualize the perturbation caused by a drug on the SARS-CoV-2 host protein subnetwork. When a drug is selected, each node (host protein) is colored based on the strength of the resulting kernel score. This tool complements CoVex, by Sadegh et al.,^54^ which analyzes the interplay within the virus-host-drug triad using paths on the interactome. Instead, CoREx calculates the effects that drugs have on individual host proteins through the different graph kernels. We have preloaded our interactome analysis tool with those FDA-approved drugs that have available drug targets from DrugBank.^47^ Users can also submit a list of drug targets, and visualize the perturbation that a hypothetical drug (or drug combination) with those targets would have on the host proteins subnetwork.

CoREx is available at https://paccanarolab.org/corex and supporting datasets are updated every 2 weeks. The project is also open-source, and the repository is publicly available at https://github.com/paccanarolab/corex.

## Discussion

The development of computational approaches that can assist in the rational and fast discovery of treatments is critical for emergent infectious diseases such as COVID-19.^1^, ^2^, ^3^^,^^6^^,^^48^ Drug repositioning, the re-use of drugs already on the market, can help to speed up the development of such treatments by prioritizing known safe-in-human drugs for clinical trials involving COVID-19 patients. In this paper, we proposed two machine learning approaches that can assist in the prioritization of drugs, together with a human-in-the-loop website tool, CoREx, to assist current research efforts for finding drugs with therapeutic efficacy against SARS-CoV-2.

Li and De Clercq^4^ indicated that finding potential repositioning candidates for COVID-19 should be focused on two main strategies: virally targeted agents and host-targeted agents. Our matrix decomposition approach is aimed at the first repositioning strategy, whereas our network medicine approach, together with CoREx, is aimed at the second one. Our first approach ranks 126 BSAs by their predicted efficacy against SARS-CoV-2, and our second approach ranks 2,197 therapeutically diverse FDA-approved drugs by their predicted ability to perturb the COVID-19 disease module.

The objective function of our matrix decomposition approach in Equation 1 is inspired by our recent work to predict the frequencies of drug side effects.^55^ The main feature of this new model is that it can account for varying levels of uncertainties in the data. We realized that different levels of drug developmental evidence can be thought of as indicating different levels of confidence in drug-virus associations and can be interpreted as probabilities. Our new model exploits the richness of this information and its outputs can be interpreted as probabilities of drug approval. Experiments in which we randomized or removed information about drug developmental stages show that such information is key to achieve a good performance (see Note S3). The implementation of our algorithm is freely available: https://github.com/paccanarolab/DrugRepoCOVID.

Our network medicine approach aims at prioritizing FDA-approved candidates based on their network-modulated effects on the COVID-19 disease protein module. In contrast to our first approach, our network medicine approach does not explicitly model the clinical efficacy of drugs, but rather their mechanistic effects on the protein interaction network. This means that a high score points to a high probability for the drug to perturb the disease module. Note, however, that our kernel methods, like most network-based approaches,^13^ can quantify the perturbation on the interactome, but cannot predict in which way the host will ultimately be affected by such perturbations (see Note S11.2).

An important advantage of our kernel approaches is that they offer a natural way to integrate gene expression data and thus allow us to focus the models on particular proteins that play a key role in the infection. Our experiments show that the integration of transcriptomics data improves the results (see Note S8). Furthermore, we have shown that our kernels have similar performance across multiple interactomes (see Note S7).

We have shown that our predictions from both approaches are aligned to ongoing *in vitro* experiments and clinical trial studies. An interesting question is whether there is additional biological evidence of efficacy for the best scoring drugs from our approaches. We manually curated the top 20 predicted drugs obtained from each approach. Our analysis reveals that many of these drugs are linked to ongoing efforts against COVID-19: several top-ranked BSAs from our matrix decomposition model are part of ongoing clinical trials for COVID-19, or are even already approved for compassionate use in COVID-19 patients^56^, ^57^, ^58^, ^59^; several top-ranked drugs from our network medicine approach have also shown efficacy either as therapeutic alternatives or as instruments for reducing risk of infection and transmission.^60^, ^61^, ^62^, ^63^ An in-depth analysis of the top 20 predictions, including an analysis of their ATC classification and references to the literature, is presented in Note S11. A comparison with the set of drugs predicted by Gordon et al.^14^ is also provided in Note S9. Finally, while the datasets that we used in our two approaches are different, a few drugs could be predicted by both methodologies—these are analyzed in Note S12.

Our computational approaches leverage available data to produce the predictions. As more reliable data becomes available, we expect the performance of our models to increase accordingly. Recently, COVID-19 atlases have been published, including single-cell transcriptomics data^64^^,^^65^ that could be exploited with our approaches.

We also point out that, while we have developed and tested our two approaches for COVID-19, both of them are disease agnostic. The general principles underlying our matrix decomposition and network medicine approaches will remain valid for any other viral disease, and therefore our methods could be applied for drug repurposing in these scenarios, as long as the data are available.

Finally, the integration of heterogeneous sources of omics information with multiple layers of interconnection is a challenge in itself. Prime examples of such complex data are the molecular datasets involved in drug repositioning. We built CoREx (https://paccanarolab.org/corex ) with the goal of providing the research community with a tool for the analysis and the formulation of hypothesis about drugs that can be repurposed for COVID-19. CoREx combines transcriptomics, proteomics, and functional information about the human genome together with knowledge about drugs and their protein targets, and we make it available for the scientific community.

### Limitations of the study

Our matrix decomposition approach is applicable to any drug for which the developmental stage associating it to a viral disease is known. The drug may or may not be virally targeted, and the model itself will not impose such a restriction. The main limitation of the method is that it relies on drug-virus associations annotated with their stage of development, and publicly available data of this type is currently scarce—we only found this type of information in the manually curated dataset by Andersen et al.^17^ that we used in our study. The main limitation of our network medicine approach is that it can only be applied to drugs with known protein targets on the host interactome.

## Experimental procedures

### Resource availability

#### Lead contact

The lead contact for this work is Alberto Paccanaro (alberto.paccanaro@rhul.ac.uk).

#### Materials availability

This study did not generate new unique reagents.

### Datasets

• *The drug-virus dataset.* We used the dataset curated by Andersen et al.^17^ (downloaded April 6, 2020). Drugs were mapped to DrugBank IDs, when available. Each drug-virus association was annotated with their developmental status/stage. There are eight stages of development in the dataset, namely: cell culture/co-culture, primary cells/organoids, animal model, clinical trials phase I, phase II, phase III, phase IV, and approved. In total, our dataset contains 850 associations between 126 BSAs and 80 viruses.
• *Protein interaction network.* The PPI network was obtained from Gysi et al.,^13^ which contains 18,505 human proteins, and 327,924 interactions.
• *FDA-approved drugs and drug targets.* FDA-approved drugs and their drug targets were retrieved from DrugBank^47^ and Gysi et al.^13^ Our set of drugs consisted of 2,197 FDA-approved drugs consisted. Our set of drug target associations consisted of 14,941 pairs of drug and targets.
• *Host proteins.* Our COVID-19 disease module consisted of 336 host proteins. It includes 332 host proteins reported by Gordon et al.,^14^ the entry receptor ACE2,^66^ and three SARS-CoV-2 entry-associated proteases TMPRSS2,^67^ CTSB, and CTSL.^68^
• *Gene expression data.* To weight the host proteins in our kernel-based methods, we used gene expression data from 430 COVID-19 patients, and 54 controls, collected from nasopharyngeal swabs.^69^ The RNA-seq raw counts are available in the Gene Expression Omnibus (GEO),^70^^,^^71^ with accession number GSE152075. We processed the data using the edgeR package,^72^ and obtained the absolute value of the log fold change comparing the expression levels between COVID-19 patients and controls. For 47 host proteins with missing mRNA levels, we assigned the minimum absolute value of the log fold change. The final weights of the host proteins are available from Mendeley Data (see Table S7).
• *In vitro data.* We built a binary dataset, assigning positive labels to drugs that were reported to show efficacy against SARS-CoV-2 infection *in vitro*, and negative labels to all other drugs. Data for drug efficacy *in vitro* was built as the union of experiments reported by Riva et al.^49^ and Gysi et al.^13^ Eighty-one FDA-approved drugs show *in vitro* effects (see Table S7).
• *Clinical trials data.* We built a binary dataset and assigned positive labels to drugs that are involved in clinical trial studies, and negative labels to all other drugs. Information for clinical trials studies was downloaded from ClinicalTrials.gov on December 1, 2020.^73^ Drugs were mapped to the DrugBank database^47^ by matching their names (see Table S7).
• *CMAP data.* For the CMAP query, we used a COVID-19 signature by Ghandikota et al.^74^ This gave us a list of 106 genes upregulated and 41 genes downregulated in three different models of SARS-CoV-2 infection from transcriptomics data. Two are models *in vitro* (Calu-3 and Vero E6 cells), and one model is *in vivo* (Ad5-hACE2-sensitized mice). The query with these data resulted in 30 drugs with significant negative τ score (τ < −90) that were mapped to DrugBank. Twenty-three of these 30 drugs are FDA approved and have targets in the Gysi et al. interactome. The list of 30 drugs with CMAP evidence is available from Mendeley Data (see Table S7). All supplementary files available from Mendeley data and external data sources are listed in Table S7 (Note S13).

### The multiplicative learning algorithm for the matrix decomposition model

To minimize Equation 1 subject to non-negative constraints, we developed an efficient multiplicative learning algorithm inspired by the diagonally rescaled principle of NMF.^21^ The algorithm consists of iteratively applying the following multiplicative update rules:

(Equation 2) $$\begin{array}{l} P_{ia}\leftarrow P_{ia}\frac{{\left(\left[M^{A}\circ Y+\sum_{s\in \left\{B,C,D,E\right\}}\alpha_{s}\left(M^{s}\circ Y\right)\right]Q^{T}\right)}_{ia}}{{\left(\left[M^{A}\circ \left(PQ\right)+\sum_{s\in \left\{B,C,D,E\right\}}\alpha_{s}M^{s}\circ \left(PQ\right)+\alpha_{z}M^{z}\circ \left(PQ\right)\right]Q^{T}\right)}_{ia}}\\ Q_{aj}\leftarrow Q_{aj}\frac{{\left(P^{T}\left[M^{A}\circ Y+\sum_{s\in \left\{B,C,D,E\right\}}\alpha_{s}\left(M^{s}\circ Y\right)\right]\right)}_{aj}}{{\left(P^{T}\left[M^{A}\circ \left(PQ\right)+\sum_{s\in \left\{B,C,D,E\right\}}\alpha_{s}M^{s}\circ \left(PQ\right)+\alpha_{z}M^{z}\circ \left(PQ\right)\right]\right)}_{aj}}\text{.} \end{array}$$

Following the guidelines to implement NMF,^75^ a small number $\varepsilon =10^{-8}$ was added to the denominators in Equation 2 to prevent division by zero, and we initialized *P* and *Q* as random dense matrices uniformly distributed in the range [0,0.1]. Furthermore, to avoid the well-known degeneracy^20^ associated with the invariance PQ under the transformation P→PΛ and $Q\to {\text{Λ}}^{-1}Q$, for a diagonal matrix Λ, we normalized *P* at each iteration as follows:

(Equation 3) $$Q_{aj}\leftarrow \frac{Q_{aj}}{q_{a}}\text{,}$$

where $q_{a}$ denotes the *a*th row vector of *Q*.

The stopping criteria of our algorithm was based on the maximum tolerance of the relative change in the elements of *P* and *Q*. The default value was $tolX<10^{-3}$, which occurred typically in about 1,000 iterations for k=5.

Using a similar procedure to Galeano and Paccanaro,^55^ it can be easily shown that our algorithm in Equation 2 satisfies the KKT conditions of convergence.

### Cross-validation procedure and model selection for the matrix decomposition approach

We used a LOOCV procedure to evaluate the performance of our matrix decomposition model. To set the model hyperparameters: *k*, $\alpha_{E}$ and $\alpha_{Z}$, we performed LOOCV on the drug-virus associations with clinical trials developmental stages (validation set). We performed a grid-search and selected the hyperparameters that maximize the mean recall across the top 1, 5, 10, 15, 20, 25, and 30 predictions retrieved. We found that k=5, $\alpha_{E}=0.01$, and $\alpha_{Z}=2$ provided a good performance. The other hyperparameters of our model were set based on the probabilities of success reported by Dowden and Munro^25^ for anti-infective drugs on distinct phases of clinical trials, i.e., $\alpha_{B}=0.16$ (phase I), $\alpha_{C}=0.27$ (phase II), and $\alpha_{D}=0.71$ (phase III). Having set all these hyperparameters, we performed an LOOCV on the test set corresponding to drug-virus associations that have been approved or are in phase IV of clinical trials. The model selection for the competitors was performed on the same validation sets (see details in Note S1).

The trained model that we used in the “Evaluation” section was obtained by training the model 1,000 times using all the available data with optimal hyperparameters. We then selected the solution that gave the lowest value in the loss function.

### Graph kernels

A PPI network is represented by a graph G=(V,E), in which $V=\left\{1,2,\ldots ,n_{V}\right\}$ is the set of nodes (proteins), and *E* the a set of links connecting the nodes (protein interactions). If the graph is weighted, then for each edge e∈E we associate a non-negative real value w(e). Let H∈V denote the set of host proteins. Our goal is then to perturb the subnetwork induced by H, i.e., the host protein subnetwork.

Here, we rely on different graph kernels described in the literature.^35^^,^^36^^,^^76^ In the following, graph kernels and their properties are defined as in Kondor and Vert.^77^ A graph kernel k:V×V↦R provides a similarity metric on the set of nodes *V* based on the graph structure. It is positive definite, that is, for any i,j∈V and any $c_{i},c_{j}\in R$ we have that $\sum_{i=1}^{n_{V}}\sum_{j=1}^{n_{V}}c_{i}c_{j}k\left(i,j\right)\geq 0$.

We can use it to define distances or similarities on a latent feature space. More specifically, there exists the feature mapping φ:V↦F such that k(i,j)=⟨φ(i),φ(j)⟩ for all i,j∈V. A graph kernel can be represented by an $n_{V}\times n_{V}$ matrix *K* whose elements correspond to $K_{i,j}=k\left(i,j\right)$ for every i,j∈V. It is usually defined in terms of the normalized Laplacian, which we explain below.

Let *W* be an $n_{V}\times n_{V}$ matrix denoting the weighted adjacency matrix of *G*. That is, $W_{i,j}=w\left(e\right)$ if there is an edge *e* connecting *i* and *j*, and $W_{i,j}$ = 0, otherwise. If *G* is unweighted, we assume that w(e)=1 for every edge e∈E. Let *D* denote an $n_{V}\times n_{V}$ diagonal matrix in which each diagonal element corresponds to the node degree, that is, $D_{i,i}=\sum_{j=1}^{n_{V}}W_{i,j}$ for every i∈V. The Laplacian is defined as D−A, and its pseudoinverse (Moore-Penrose inverse) is denoted by $L^{+}$. The normalized Laplacian is defined as $\tilde{L}:=I-D^{-\frac{1}{2}}WD^{-\frac{1}{2}}$, where *I* denotes the identity matrix.

There are different ways to define *K* and we focus on five graph kernels^35^^,^^36^^,^^76^: regularized Laplacian, diffusion process, and *p*-step random walk in terms of the normalized Laplacian^36^ (see Table 1).

In the *p*-step random walk, p≥1 and a≥2 are given parameters.^36^ The element $K_{i,j}$ measures how likely it is to go from node *i* to node *j* after *p* steps in a random walk. If we generalize it to a continuous time (infinitesimally small steps) and take an infinite number of steps, we have the diffusion process $K=\text{exp}\left(-\sigma^{2}/2\tilde{L}\right)$, where σ is a parameter controlling the diffusion. Finally, the regularized Laplacian kernel can be thought of as the convergence of an iterative process in which nodes spread information to their neighbors at each step.

We used different kernels from Smola and Kondor,^36^ Cao et al.,^35^ and Zhou et al.,^76^ which are implemented in the R package *diffuStats*^78^ for the commute time, diffusion, *p*-step, regularized Laplacian, and inverse cosine kernels. We set the parameter *p* to 2 for the *p*-step kernel. For the remaining kernels, we used the default parameters in *diffuStats*.

### CMAP evaluation details

We consider that a drug has CMAP evidence against COVID-19 if the changes that it causes to gene expression are opposite to the ones caused by the disease.^15^ To build the CMAP evaluation set, we used the CMAP pipeline^15^^,^^16^ to measure how similar or opposite the drug and COVID-19 expression profiles are. We used version 1.0 of the CMAP L1000 dataset^16^ available on clue.io website (https://clue.io/).

We began by obtaining a list of up-/downregulated genes in COVID-19 (genes that have higher/lower expression levels in SARS-CoV-2 infected cells compared with non-infected cells). Then, we queried the COVID-19 signature in CMAP. For each drug, CMAP has a list of genes ordered from the most expressed to the least expressed after treatment (in comparison with the expression levels with no treatment). If the upregulated genes in COVID-19 are located on the bottom of the list (that is, if they have low expression levels in cells treated with the drug), and the downregulated genes are located on the top (that is, they have high expression levels in cells treated with the drug), we say that the drug and disease signatures have a strong negative correlation. If we observe the opposite (upregulated genes on top, and downregulated genes on bottom), we say that they have a strong positive correlation.

For each drug, CMAP outputs an enrichment score that is positive when the correlation between the drug and disease signatures is positive (the drug mimics the disease), and negative when the correlation is negative (the drug reverses the disease). The final values (denoted by τ) are compared with a reference database and normalized between −100 and 100.

## Data Availability

Original data have been deposited to Mendeley Data: https://doi.org/10.17632/p7y5wmschg.1. The implementation of our matrix factorization model can be found at https://github.com/paccanarolab/DrugRepoCOVID. CoREx is available at https://paccanarolab.org/corex, and the source code is publicly available at https://github.com/paccanarolab/corex.

## Supplemental information

Document S1. Figures S1–S20, Tables S1–S7, and Notes S1–S13

Document S2. Article plus supplemental information
