# Supplementary material for: Machine learning and network medicine approaches for drug repositioning for COVID-19
Source: Patterns (N Y). 2021 Nov 9;3(1):100396. doi: 10.1016/j.patter.2021.100396 (PMC8576113; doi:10.1016/j.patter.2021.100396)
Supplement: Document S1. Figures S1–S20, Tables S1–S7, and Notes S1–S13 [file mmc1.pdf]

Patterns, Volume *3*

Supplemental information

Machine learning and network medicine approaches for drug repositioning for COVID-19

Suzana de Siqueira Santos, Mateo Torres, Diego Galeano, María del Mar Sánchez, Luca Cernuzzi, and Alberto Paccanaro

1 Note S1. On the differences with other NMF-based drug repositioning methods

Drug repositioning hypotheses can be formulated from different types of biological data, including chemical, molecular[1], cellular[2] and clinical[3].

In computational pharmacology, the drug repositioning problem is often framed in terms of a incomplete association matrix Y of $n \times m$, where the goal is to predict missing associations in Y. In our case, Y contains relationships between n drugs and m viruses, where $y_{ij} = 1$ if drug i is associated to virus j, or $y_{ij} = 0$ otherwise.

Several computational methods have proposed new models based on Non-negative matrix factorisation (NMF)[4,5] for distinct settings of the drug repositioning problem. Here we review some of these methods and highlight key differences with our matrix decomposition model. We start with the standard or vanilla NMF of Lee and Seung[4,5].

Vanilla NMF There are two main differences between our model and the vanilla NMF model of Lee and Seung[4]: the cost function and the learning algorithm. To explain these difference in more detail, let us denote a generic data matrix of $n \times m$ as Y. The goal of NMF is to obtain a low-rank decomposition of Y by minimising the following cost function:

$$\begin{aligned} &\min_{P,Q} \mathscr{J}(P,Q) = \frac{1}{2}\|Y - PQ\|_F^2 = \frac{1}{2}\sum_{ij}(Y_{ij} - (PQ)_{ij})^2 \\ &\text{subject to } P,Q \geq 0 \end{aligned} \tag{1}$$

where P is a matrix of $n \times k$ and Q is a matrix of $k \times m$, k is the rank of the approximation and $||.||$ is the Frobenius norm of a matrix.

Notice that Equation (1) is non-convex in P and Q. Lee and Seung[4,5] derived multiplicative learning rules that can find a local minimum solution of Equation (1) under non-negative constraints. The multiplicative algorithm consist on iteratively applying the following update rule assuming that P and Q are initialised from random values in $t = 0, t \in \{1, 2, ...\text{maxiter}\}$ (e.g. uniform distribution in the range $[0,1]$):

$$\begin{aligned} P &\longleftarrow P\frac{YQ^T}{PQQ^T} \\ Q &\longleftarrow Q\frac{P^TY}{P^TPQ} \end{aligned} \tag{2}$$

The multiplicative learning rules in Equation (2) have the advantage that they do not require setting a learning rate or applying projection functions to guarantee the non-negative constraints.

Notice that when minimising Equation (1), all the entries in Y are considered equally. This makes sense when entries in Y have the same meaning or importance. For instance, if it contains pixels of images[4]. However, in our problem, the data matrix Y contains drug-virus associations that are at different stages of drug development. Therefore, the strength of each known drug-virus association in Y varies according to the evidence of efficacy. In our model, we account for the varying types of evidence of efficacy of drug-virus associations by weighting them differently in our cost function. The weighting is simple: groups of drug-virus associations are weighted according to their probability of success (motivated by the study of Dowden et al.[6]). In this way, those groups in early stages of development have less importance than those in the later stages of development during the learning. Furthermore, since our cost function is different from the vanilla NMF, our learning rule to minimise it is also different. We showcase our learning algorithm in the Methods section of the main manuscript.

A recent paper by Sosnina et al. has shown that vanilla NMF[5] provide a better performance at predicting missing interactions than other algorithms used for predicting missing data in recommender systems. In their study, Sosnina et al.[7] used a dataset consisting of cell-assay derived small molecule- antiviral activity interactions.

NMF with L2 regularization Bakal et al.[8] recently proposed the use of with L2 regularisation model to predict missing drug-disease associations in the repoDB database[9], which contains approved and failed drug-disease associations for distinct categories of diseases (including genetic, neurologicals, syndromes, neoplastic, etc.). Following our previous notation, the cost function of NMF with L2 regularization is as follows:

$$\begin{aligned} &\min_{P,Q} \mathscr{J}(P,Q) = \frac{1}{2}\|Y - PQ\|_F^2 + \frac{\beta}{2}(\|P\| + \|Q\|) \\ &\text{subject to } P,Q \geq 0 \end{aligned} \tag{3}$$

That can be minimised with the following multiplicative learning rules:

$$\begin{aligned} P &\longleftarrow P\frac{YQ^T}{PQQ^T+\beta P} \\ Q &\longleftarrow Q\frac{P^TY}{P^TPQ+\beta Q} \end{aligned} \tag{4}$$

The main difference between vanilla NMF and NMF with L2 reg. is that an L2 norm is added to the loss function to improve generalization and reduce model complexity.

TriFactor NMF Ceddia et al.[10] recently proposed using non-negative matrix tri-factorization (TriFactor NMF) model for predicting missing drug-target interactions, missing drug-therapeutic categories, and missing drug-disease associations using diverse datasets. TriFactor NMF seeks to decompose the matrix Y as follows $Y \simeq FSG^T$, by minimising the following loss function:

$$\begin{aligned} &\min_{F,S,G} \mathscr{L}(F,S,G) = \frac{1}{2}\left\|Y - FSG^T\right\|_F^2 \\ &\text{subject to } F,S,G \geq 0 \end{aligned} \tag{5}$$

That can be minimised with the following multiplicative learning rules[11,12]:

$$\begin{aligned} G &\longleftarrow G\sqrt{\frac{Y^TFS}{GG^TY^TFS}} \\ F &\longleftarrow F\sqrt{\frac{YGS^T}{FF^TYGS^T}} \\ S &\longleftarrow S\sqrt{\frac{F^TYG}{F^TFSG^TG}} \end{aligned} \tag{6}$$

IRNMF Tang et al.[13] addresses the problem of predicting missing drug-virus associations with potential applications on drug repositioning for COVID-19. The model they proposed is a variation of NMF called Indicator Regularised NMF (IRNMF).

IRNMF is different from our model in the type of information it uses: IRNMF integrates chemical similarities between drugs and sequence similarities between viruses, while our model does not use this information; our model uses drug developmental stages, which are not used in the IRNMF model.

Comparison with NMF and its variations Although the specific drug repositioning problems addressed by Sosnina et al.[7], Bakal et al.[8] and Ceddia et al.[10] are different from ours (as we are predicting drug-virus associations), we can still compare their specific variant of NMF against our model on our drug-virus association data.

To this end, we implemented the NMF with L2 regularization and the triFactor NMF model. We then used our training and validation sets to set the hyperparameters of each model: (i) For the NMF with L2 model, we found optimal performance in the validation set with: number of latent factors = 5, and the L2 regularization penalty = 0.1; (ii) For the triFactor NMF, we found optimal performance in the validation set with: number of factors = 15.

Using the Leave-One-Out Cross-Validation (LOOCV) procedure described in the paper, we assessed the performance of each of these methods at retrieving missing phaseIV/approved drug-virus associations. The Figure 3 in the main manuscript shows that our matrix decomposition model significantly outperforms the two variants of NMF. We also observed that NMF with L2 regularization and triFactor NMF perform slightly better than vanilla NMF.

In order to run IRNMF on our dataset, we followed the procedure in Tang et al.[13] to obtain chemical similarities for our set of drugs and sequence similarities for our set of viruses.

To obtain chemical similarities between our set of 126 drugs, we mapped drug names to DrugBank 5.1 (`https://go.drugbank.com/`) identifiers. 109 out of the 126 drugs could be mapped to DrugBank. We then obtained the chemical SMILES representation for each drug from DrugBank. Finally, using the open source RDKit Chemoinformatics tool (`https://www.rdkit.org/`), we obtained the MACCS chemical fingerprint and computed the 2D Tanimoto chemical similarities.

To obtain virus similarities for our set of 80 viruses, we downloaded the most complete reference genomes of each virus from NCBI (`https://www.ncbi.nlm.nih.gov/genome/`). Following Tang et al.[13], we used the MAFFT algorithm

to get the distance matrices. This could only be applied to the viruses with a single DNA segment, which resulted in 64 out of 80 viruses in our dataset.

In this way, we obtained the three matrices required to run by IRNMF: a binary drug-virus matrix containing 654 associations between 109 drugs and 64 viruses; a chemical similarity matrix of 109x109; a sequence similarity matrix of 64x64. In this drug-virus matrix, 66 associations are in phases I, II or III of development, and 49 associations are in phase IV/approved stages.

Having created the datasets, we performed the evaluation of the two models. First, we used the 66 phase I-III associations to set the model parameters both for our model and for IRNMF (optimal performance for IRNMF was obtained with: number latent factors = 10; L2 regularization penalty = 0.1; side information regularization = 10). Then, we then repeated our leave-one-out evaluation on the 49 associations that are in phase IV/approved stages: For each of these associations, we removed it from our dataset; trained the model with the remaining data matrix; and then ranked drugs based on the predicted scores. To run IRNMF in our dataset, we used the code provided by the authors in `https://github.com/dukebai/IRNMF`.

Figure S1 below shows that our matrix decomposition model significantly outperforms IRNMF at recovering missing phase IV/approved drug-virus associations at different number of top predictions retrieved. We observed that our method performs from 26.53% to 53% better than IRNMF at recovering phase IV/approved missing drug-virus associations.

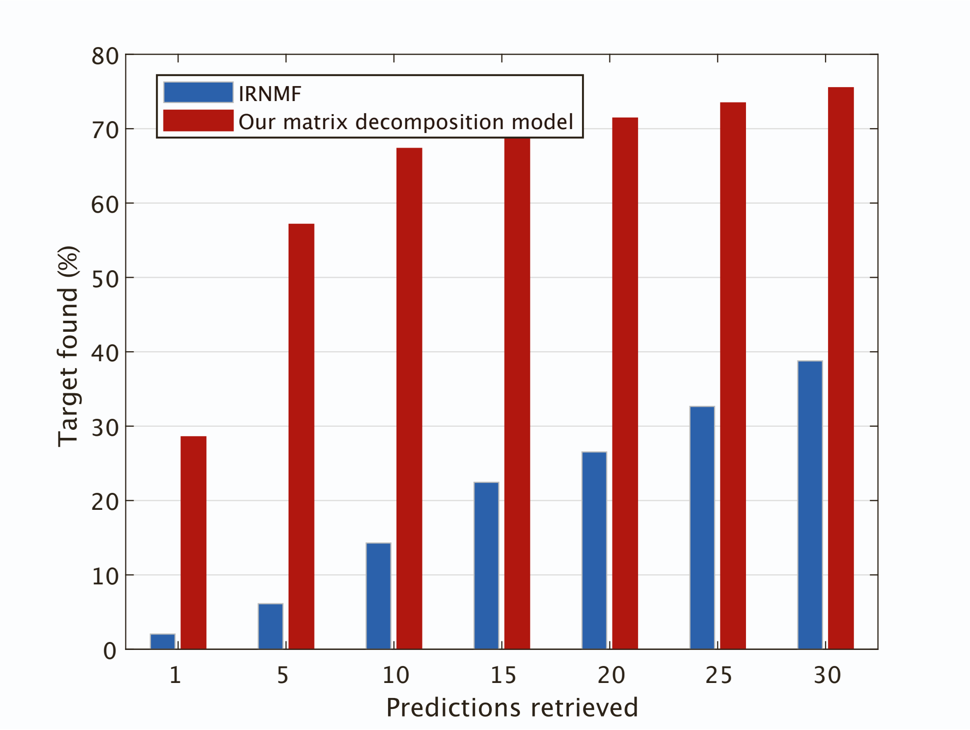

Figure S1. Performance comparison between IRNMF[13] and our matrix decomposition model at predicting missing phase IV/approved drug-virus associations in the Andersen et al.[14] drug-virus dataset.

Comparison in the SARS-CoV-2 specific evaluation dataset. We also seek to compare the performance of the methods on the specific evaluation datasets that we collected for SARS-CoV-2. In the evaluation that follows, we used the *in vitro* and *clinical trials* datasets, but not the CMAP dataset, because only one drug with CMAP activity was found in the Andersen et al.[14] dataset. In the *in vitro* dataset, we found 10 BSA drugs with *in vitro* efficacy against SARS-CoV-2; 4 of which were already in the Andersen et al.[14] dataset. In the clinical trials dataset, we found 28 BSA drugs in clinical trials for COVID-19; 6 of which were already in the Andersen et al.[14] dataset.

We performed a leave-one-out evaluation experiment in the *in vitro* dataset, where one of the ten drugs was left out and the other nine were also used for training together with the ten associations from the Andersen et al.[14] dataset. In our evaluations, we only ranked drugs without known associations in training. Therefore, the final set of labels consisted on 10 positive labels and 110 negative labels. In the clinical trials dataset, we could not repeat the leave-one-out evaluation experiment because we do not know the specific phase of clinical trial. In this case, we simply run the methods using all the data available in the Andersen et al.[14] dataset combined with the *in vitro* evaluation dataset. The performance was also calculated only on drugs without associations in training, i.e., 20 positive labels and 90 negative labels. Finally, all the methods were run using their optimal hyperparameters.

Figure S2 below shows the recall and precision of the methods at different values of predictions retrieved. In the clinical trials evaluation dataset, our matrix decomposition model greatly outperforms the competitors. It can recover 50% of BSA drugs already in clinical trials for COVID-19 in the top-20 drugs retrieved. In the *in vitro* evaluation dataset, we noticed that the best performing method changes in different tops. For instance, our matrix decomposition model performs on part of vanilla NMF in the top-10. We notice here that our method was not tuned to fit *in vitro* data in the Andersen et al. dataset , but rather phase IV/approved stages (i.e., clinical success of drugs). This might explain why our method performs much better than other competitors on the clinical trials dataset but not so well on the *in vitro* data. In fact, predicted scores by our matrix decomposition model on the *in vitro* developmental stages are very low (see Figure S4). Another explanation for the variation of the performance of the methods in the *in vitro* data could be due to the fewer number of positive labels available.

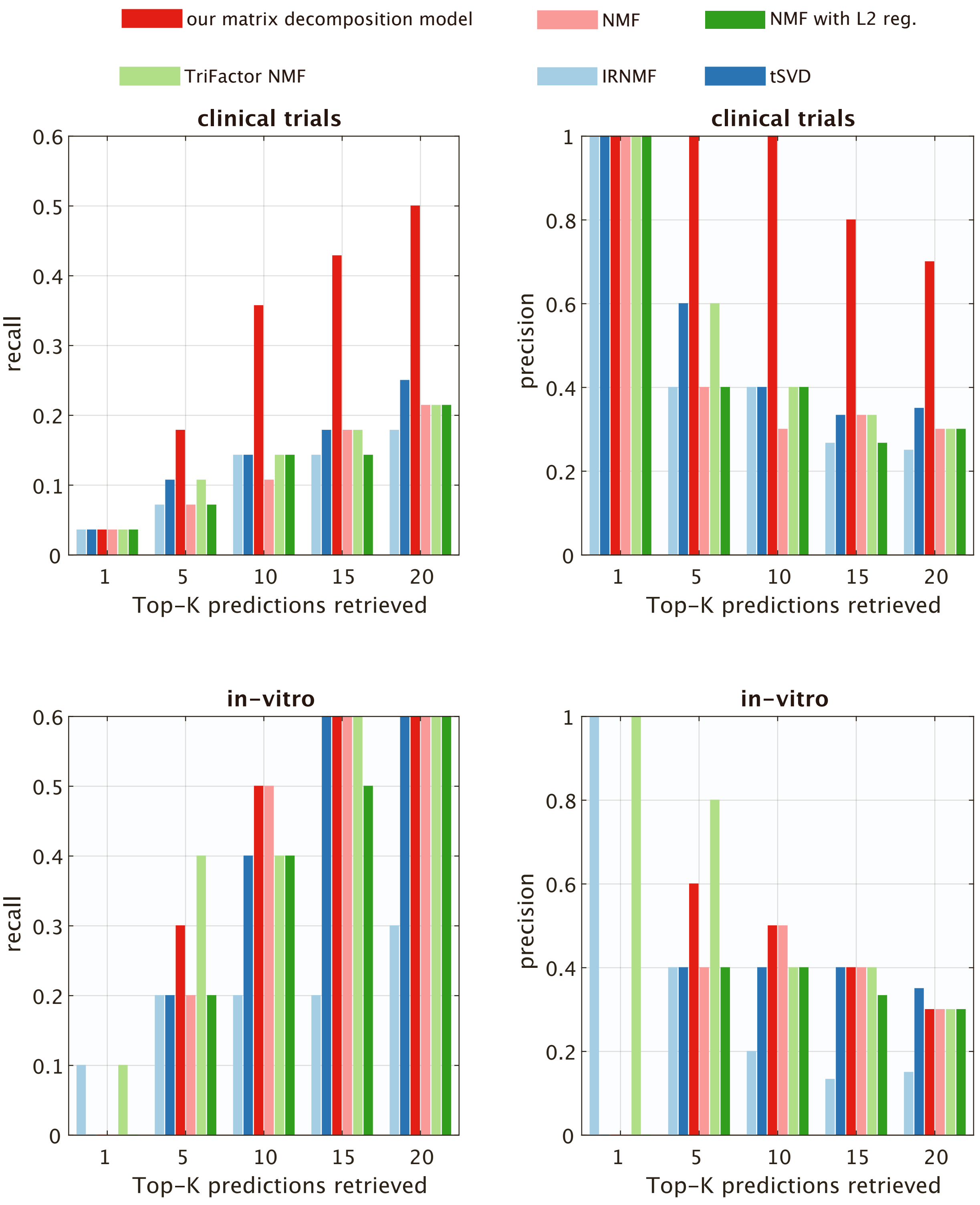

Figure S2. Precision and recall at different number of BSA drugs retrieved by different matrix factorization drug repositioning methods in two evaluation datasets specific to SARS-CoV-2. **clinical trials:** 20 BSA undergoing clinical trials for COVID-19 vs 90 remaining drugs. ***in vitro*:** 10 BSA with activity *in vitro* against SARS-CoV-2 vs 110 remaining drugs.

2 Note S2. On whether the use of developmental information can bias the predictions

One question that may arise when using our matrix decomposition model is whether the grouping by developmental stages in our cost function introduces a bias in the predictions. In other words, whether our model is only predicting drugs that are in the late stages of development, rather than the most effective ones. We will show that, in the intended use of our matrix decomposition model, when predicting missing drug-virus associations, the bias of developmental information cannot affect the predictions because developmental information is not available for them.

To understand how the grouping influences the predictions made by our matrix decomposition model, let us consider that our model takes as input an incomplete sparse drug-virus matrix with only 8.43% non-zero entries, and outputs predicted scores for all the entries in the matrix (see Figure S3 below). These predicted scores can be categorised into two classes:

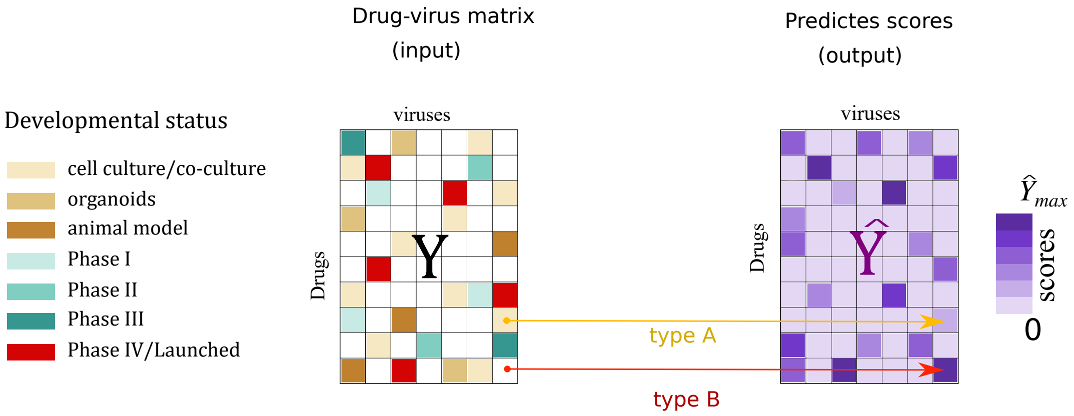

Figure S3. The predictions made by our matrix decomposition model can be categorised based on whether the predicted scores are used to rank non-approved drug-virus associations available for training (type A, an example shown with an arrow in yellow) or missing drug-virus associations, without *any* evidence of efficacy (type B, an example shown with an arrow in red). Our model is intended for type B predictions.

- **Type A predictions**. These corresponds to entries y_{ij} in the original matrix Y that are non-zero. Here we are predicting whether drug i, which is already in some stage of development with respect to virus j, will eventually reach drug approval for virus j.
- **Type B predictions**. These corresponds to entries y_{ij} in the original matrix Y that contains zeros. Here we are predicting whether drug i, which is not currently being developed for virus j, will reach drug approval for virus j.

In our manuscript, we focus on validating predictions of type B, because it corresponds to the interesting case where the scores predicted by our model can be used to rank drug-virus associations that are not yet in drug development, and for which novel drug repositioning hypotheses can be made. In the manuscript, we have showcased the utility of our approach in this scenario by using a leave-one-out cross-validation (LOOCV) procedure. In this procedure, only one drug-virus association (in phase IV or approved) was removed from the drug-virus matrix Y (by setting the corresponding entry to zero). We then trained the model, and scores were predicted for all the drugs. We then ranked drugs that had no known association with that virus and checked the percentage of cases in which the effective drug for the virus was found among the top-K predictions. Fig. 3 in the main manuscript shows that our matrix decomposition model can predict 60% the correct drug among the top-10 predictions retrieved.

Predictions of type A typically require a different setting where the goal is to predict clinical success of candidate compounds in early stages of drug development given molecular, chemical, cellular and clinical features[15]. Therefore, using our model for type A predictions is likely to generate predictions that are biased towards the known developmental stages. One way to verify this is to look at the scores that we obtain for drug-virus associations for the 850 known drug-virus associations that were used during training. These are shown in the Figure S4 below, which presents the scores grouped based on their known developmental status. We can see that, on average, the higher the developmental stage, the higher the score output by our model.

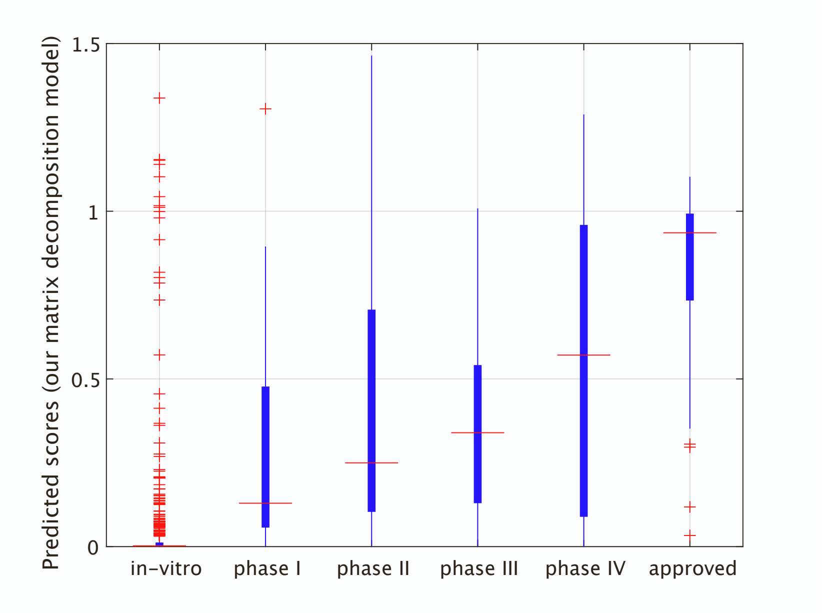

Figure S4. Predicted scores by our matrix decomposition model for drug-virus associations used for training the model. 850 drug-virus associations were grouped based on their known developmental status.

We analysed the top-20 predictions obtained from our system, separating them into Type A and Type B predictions. The top type B predictions, corresponding to predicted Broad-Spectrum Antiviral drugs not known to be under development against SARS-CoV-2 according to the Andersen et al. dataset, are listed in Table S1.

Rank	Predicted score	Drug name (ID)	Additional curated evidence for COVID-19
5	0.46	Tenofovir (DB14126)	combinational therapy, currently on ClinicalTrials.gov (NCT04519125)
6	0.43	Lamivudine (DB00709)	combinational therapy, case report in Brasil[16]
8	0.37	Zanamivir (DB00558)	*in silico* molecular docking[17]
10	0.21	Azithromycin (DB00207)	combinational therapy, clinical trials[18], compassionate use[19]
11	0.20	Amiodarone (DB01118)	currently on ClinicalTrials.gov (NCT04351763)
12	0.19	Artesunate (DB09274)	combinational therapy, *in vitro*[20], currently under WHO Solidarity PLUS clinical Trials
14	0.13	Thymalfasin (DB04900)	currently on ClinicalTrials.gov (NCT04487444)
15	0.12	Brincidofovir (DB12151)	NA
16	0.12	Rapamycin (DB00877)	currently on ClinicalTrials.gov (NCT04461340), geroprotective[21]
17	0.11	Nitazoxanide (DB00507)	currently on ClinicalTrials.gov (NCT04463264)
18	0.10	Sunitinib (DB01268)	AI-evidence[22]
19	0.08	Erlotinib (DB00530)	AI-evidence[22]
20	0.07	Valacyclovir (DB00577)	NA
21	0.07	Interleukin-7 (DB11997)	clinicalTrials.gov (NCT04379076), compassionate use[23,24]

Table S1. Predictions of type B obtained with our matrix decomposition model:. Predictions of type B correspond to predicted broad-spectrum antiviral drugs not known to be under development against SARS-CoV-2 according to the Andersen et al. dataset. These predictions had a value of zero in the drug-virus matrix Y used for training. Only predicted drugs within the top-20 predictions are shown.

Interestingly, this set of drugs includes drugs for which we could find in the literature some indication of efficacy, and

for several of them we found that they are already in clinical trials for COVID-19 (third column in Table S1). Note that our 12th top predicted drug is the anti-malarial drug Artesunate, which was selected for the currently ongoing WHO Solidary Plus Clinical Trials[25].

The top predictions of type A, corresponding to predicted Broad-Spectrum Antiviral drugs already known to be under development, but not approved against SARS-CoV-2 according to the Andersen et al. dataset, are shown in Table S2. As expected, for the six drugs known to be under development for SARS-CoV-2, predictions are biased towards drugs that are in later stages of development. The drug with the highest predicted score was Favipiravir, already known to be in phase II of clinical trials for COVID-19 according to the Andersen et al. dataset. Favipiravir is an RNA polymerase inhibitor that was developed in Japan as an antiviral treatment for influenza. In October 2020, already 37 clinical trials were registered in ClinicalTrials.org to assess its efficacy and safety for COVID-19 patients[26].

**Rank**	**Predicted score**	**Drug name (ID)**	**Dev. Stage available for training**	**Additional curated evidence for COVID-19**
1	1.03	Favipiravir (DB12466)	phase II	*in vitro*[27], several clinical trials[28,29], compassionate use[19]
2	0.95	Arbidol (DB13609)	phase IV	*in vitro*[30], under clinical trials[31]
3	0.58	Lopinavir (DB01601)	phase IV	combinational therapy, clinical trials in severe COVID-19[32], compassionate use[19], little or no clinical benefit[33]
4	0.58	Ritonavir (DB00503)	phase III	combinational therapy, clinical trials in severe COVID-19[32], compassionate use[19]
7	0.42	Remdesivir (DB14761)	phase III	compassionate use[34], clinical trials[27]
9	0.28	Hydroxychloroquine (DB01611)	phase III	compassionate use[19], Emergency Use Authorisation (EUA) by FDA[35] (revoked on June, 2020), little or no clinical benefit[33].
13	0.17	Chloroquine (DB00608)	cell culture/co-culture	Emergency Use Authorisation (EUA) by FDA (revoked on June, 2020), little or no clinical benefit[33,36].

Table S2. Predictions of type A obtained with our matrix decomposition model:. Predictions of type A correspond to predicted Broad-Spectrum Antiviral drugs already known to be under development, but not approved, against SARS-CoV-2 infection. Only predicted drugs within the top-20 predictions are shown.

However, a systematic review of the randomised clinical trials suggest that Favipiravir has shown low efficacy for patients with mild or moderate COVID-19[26]. In fact, other drugs including Remdesivir, Lopinavir and Hydroxychloroquine have shown little to no clinical efficacy in COVID-19 patients according to the WHO Solidarity Trial, conducted in more than 11,000 patients across 405 hospitals in 30 countries[33]. Therefore, the lack of efficacy of BSA drugs that were under development at the time of release of the Andersen et al. dataset suggests that it is important to consider predicted BSA drugs that were not yet under development against SARS-CoV-2 (presented in Table S1).

3 Note S3. How much our model exploits developmental stages information?

An important question is how much of the performance of our model was due to the use of the developmental stages' information in our modelling. To understand this, we designed two different experiments:

- **Randomisation of developmental stages**. We randomised the developmental stages assigned to drug-virus associations in the matrix used for training. This experiment is designed to inform us about whether there is relevant information in the specific subsets of developmental stages used.
- **Removing the information of the developmental stages from our model**. To understand whether the prediction performance of our model depends on assigning different probabilities of success to different groups of entries, we set all the coefficients assigned to the different drug developmental stage groups to 1 (i.e. $\alpha_B = \alpha_C = \alpha_D = \alpha_E = 1$). Only the α_z coefficient of our original model is retained, thus effectively weighing down the information brought in by the zeros (note that our model reduces to the model by Galeano et al.[37]). In this case, the information of the developmental stages is removed altogether from the cost function as all the known drug-virus associations are considered equal during learning.

For both experiments, we followed the leave-one-out cross validation (LOOCV) that we described in the main manuscript. For each of the 71 phase IV/approved associations, we removed it from the drug-virus matrix Y by setting its corresponding entry to zero, and then trained the model with the remaining data. Then, we ranked the drugs with not known association in training based on their predicted scores. The prediction performance was then measured on the ability of the method to predict the correct association among the top-K predictions retrieved.

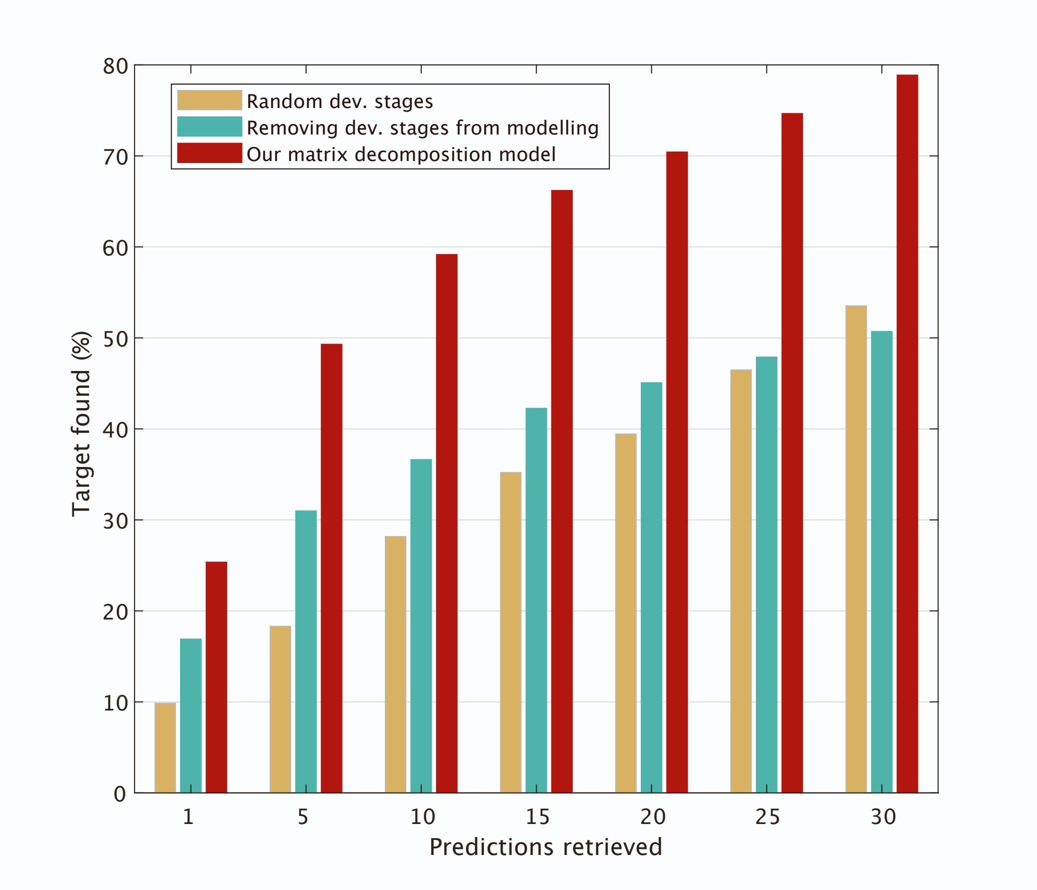

Figure S5. Comparison of the performance of our matrix decomposition model at predicting approved/phase IV Broad Spectrum Antivirals (BSA) for 28 viruses under two control procedures to illustrate the importance of the modelling of the developmental stages. In the first experiment, we randomised the developmental stages of the drug-virus associations used for training (brown bars). In the second experiment, we removed the modelling of the developmental stages from the cost function (green bars).

Figure S5 shows a comparison of the prediction performance of the two competitors against our matrix decomposition model. Our method outperforms significantly both competitors. It predicts 15-31% better than the competitor that randomises the developmental stages (brown bars), and by 8.45-28% better than when the developmental stage information is removed from the cost function (green bars). Our experiments suggest that the integration of the developmental stages in our cost function leads to significant improvements in prediction performance.

4 Note S4. Evaluation datasets for the network medicine approach

Our network medicine approach was thoroughly evaluated and compared using three different PPI networks, as well as several evaluation datasets. In this section, we provide details of all the datasets involved in each setting.

We consider 3 PPI networks, as described in Table S3. Ultimately, the set of drugs that we are able to place in the interactome will vary with the set of proteins included in each interactome. Our main evaluation setting, and the one that involves the largest set of approved drugs, is related to the interactome put together by Gysi et al.[38]. Once the interactome is set, we retrieve all the FDA approved drugs from the DrugBank database[39]. Every approved drug with at least one protein target in the interactome is suitable for our network medicine approach.

PPI	number of proteins	number of links
**Cheng et al.[40]**	15,646	218,092
**HuRI[41]**	16,091	353,435
**Gysi et al.[38]**	18,505	327,924

Table S3. Protein-Protein Interaction networks. Three PPI networks were used throughout this study to evaluate our network medicine approach. Cheng et al.[40] and HuRI[41] were restricted to the reviewed entries in UniProtKB (UniProtKB/Swiss-Prot)[42]. The interactome put together by Gysi et al.[38] uses Entrez identifiers [https://doi.org/10.1093/nar/gks1189].

To evaluate the results, we rely on three sources of evidence:

- ***in vitro* experiments:** The union of *in vitro* screenings from Riva et al.[43] and Gysi et al.[38]. They total 99 drugs in DrugBank that show effects against SARS-CoV-2 in the screenings. 81 such drugs are FDA approved.
- **Clinical trials:** Retrieved from `ClinicalTrials.gov` on December 1st, 2020. They total 244 drugs mapped to DrugBank listed as an intervention on a clinical trial. 186 such drugs are FDA approved.
- **CMAP:** Obtained by querying CMAP[44,45] using differentially expressed genes on COVID-19 patients vs. a control group[46]. They total 30 drugs with a significant τ score (< -90). 23 such drugs are FDA approved.

Dataset	Evidence Type	Number of Drugs	Number of Positives	Number of negatives
**IV-2197**	In Vitro Evidence (Riva et al.[43] & Gysi et al.[38])	2197	81	2116
**CT-2197**	Clinical Trials retrieved on December 1, 2020	2197	170	2027
**CM-2197**	CMap evidence	2197	23	2174
**IV-1853**	In Vitro Evidence (Riva et al.[43] & Gysi et al.[38])	1853	78	1775
**CT-1853**	Clinical Trials retrieved on December 1, 2020	1853	153	1700
**CM-1853**	CMap evidence	1853	21	1832
**E918-IV**	In Vitro Evidence from Gysi et al.[38]	918	77	841
**E918-CT**	Clinical Trials retrieved on April 15, 2020	918	37	881
**E918-CM**	CMap evidence	918	19	899

Table S4. Datasets for the network medicine approach

The final step required to build an evaluation set is to assign labels to the drugs based on the available evidence. To do it, we simply intersect the suitable drugs for a given interactome with the set of drugs from one of the groups described above. The resulting evaluation settings are summarised in Table S4. The main evaluation settings are IV-2197, CT-2197, and CM-2197, which go together with the Gysi et al.[38] interactome. Settings IV-1853, CT-1853, and CM-1853 are the result of building the

set for the Cheng et al.[40] and HuRI[41] interactomes. the last 3 evaluation sets in the table (E918-IV, E918-CT, and E918-CM) are slightly different, as they are used to compare our results to those of Gysi et al.[38]. Some of the 918 drugs included in these settings were not approved by FDA, which frames a fundamentally different problem than the other evaluation sets. Details of our comparison using E918-IV, E918-CT, and E918-CM are available in Note 8.

5 Note S5. Evaluation of the network medicine approach on Gysi et al PPI

We ran network medicine methods on the Gysi et al PPI, and compared the results on three different evaluation sets: IV-2197 (*in vitro*), CT-2197 (clinical trials), and CM-2197 (CMAP).

In Figure S6, we compare prediction scores between drugs with evidence for COVID-19 and the remaining drugs for different types of evidence. We also show the corresponding One-Sided Wilcoxon-Mann-Whitney p-values. Results are shown for our network medicine approach, and two network-based competitors: the DSD method[47], and the Guney distance[48]. The kernel-based methods and DSD[47] have significant p-values in all scenarios, while the Guney distance[48] has non-significant p-value for CMAP. In every case, the kernel-based methods have the smaller p-values.

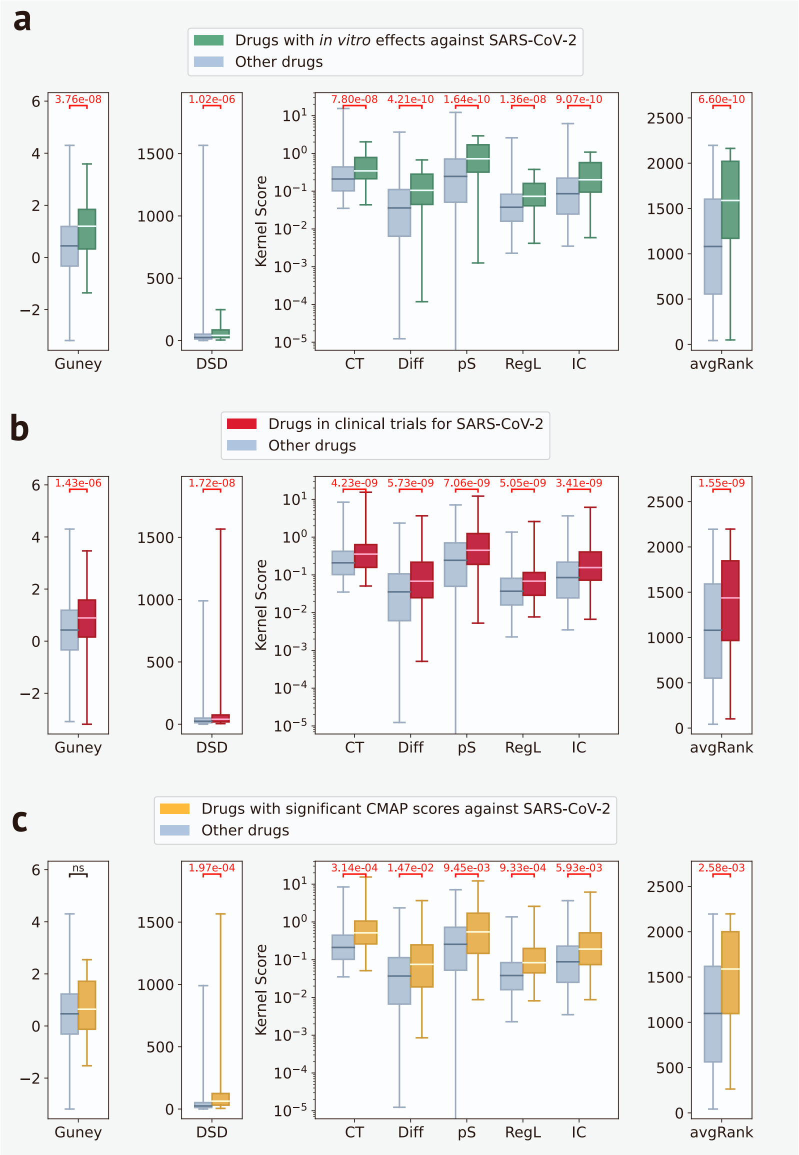

Figure S6. Prediction scores by the network medicine approach on the Gysi et al. PPI. We used boxplots to compare prediction scores between drugs with and without evidence for COVID-19 on three different evaluation sets: *in vitro* evidence (IV-2197), clinical trials evidence (CT-2197), and CMAP evidence (CM-2197). For each comparison, we computed the One-Sided Wilcoxon-Mann-Whitney p-value (one tailed test). Non-significant p-values (below 0.05) are indicated with 'ns'. Significant p-values are shwon in red. We show results obtained by our network medicine approach and competitors. Our approach includes five different kernels on graphs (commute time, diffusion, inverse cosine, p-step kernel, and regularised laplacian), and a combined ranking across the kernels (avgRank). The competitors include DSD[47], and the Guney distance[48].

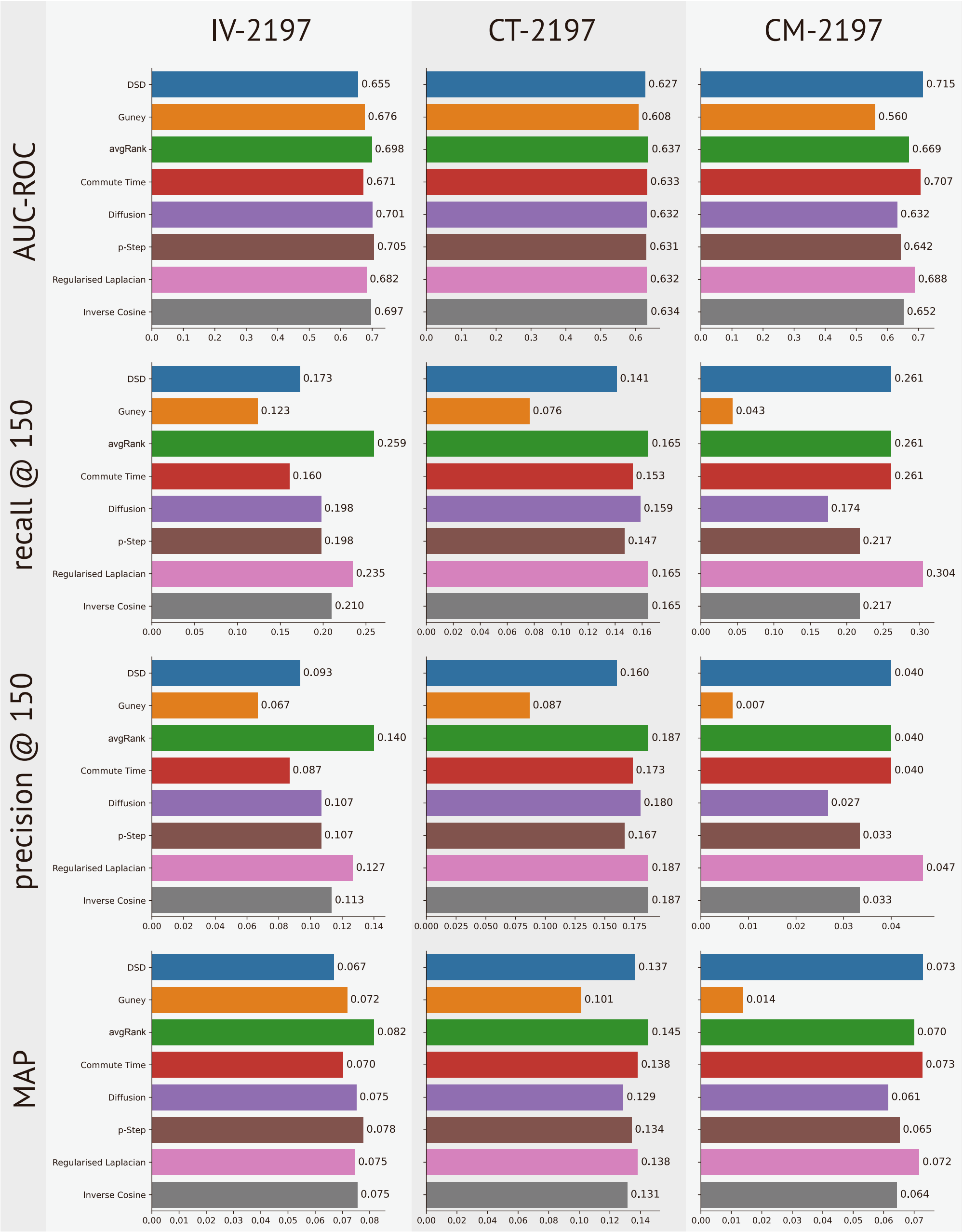

Figure S7. Classical machine learning metrics obtained by the network medicine approach on the Gysi et al. PPI. We computed the area under the ROC curve (AUC-ROC), recall at top 150, precision at top 150, and mean average precision (MAP) for three different evaluation sets. They correspond to *in vitro* evidence (IV-2197), clinical trials evidence (CT-2197), and CMAP evidence (CM-2197). For each type of evidence, we show results obtained by our network medicine approach and competitors. Our approach includes five different kernels on graphs (commute time, diffusion, inverse cosine, p-step kernel, and regularised Laplacian), and a combined ranking across the kernels (avgRank). The competitors include DSD[47], and the Guney distance[48].

Figure S7 shows traditional machine learning metrics for comparing the performance across different network medicine methods. When evaluating on the *in vitro* dataset (IV-2197), we observe that the average ranking aggregation (avgRank) is the best performing method in the recall and precision at top 150, and MAP metrics. The best performance in the AUC-ROC metric is achieved by calculating the score using our network medicine approach with the p-Step kernel.

Evaluating on the clinical trial dataset (CT-2197), we observe that the best performer throughout the four evaluation metrics is the average ranking (avgRank). In the recall and precision at top 150, it is matched only by using our network medicine

approach with the regularised Laplacian or inverse cosine kernels.

Finally, evaluating on CMAP data (CM-2197), we observe that the regularised Laplacian is the best performing method in the recall and precision at top 150, and MAP metrics. The best performance in the AUC-ROC metric is achieved by the DSD method[47] with a very similar performance achieved by calculating the score using our network medicine approach with the commute time kernel.

All in all, the main pattern we observe when comparing the performance using traditional machine learning metrics is that our network medicine approach is very robust in comparison to the competitors. This especially true for the recall and precision metrics which are, arguably, the most interesting metrics to observe when planning drug repurposing experiments.

6 Note S6. Comparison between our network medicine approach and methods by Gysi et al

Gysi et al.[38] tested 918 drugs *in vitro* against SARS-CoV-2. They proposed computational approaches to predict drugs with efficacy against SARS-CoV-2. However, the problem from Gisy et al. is slightly different from ours. Out of the 918 experimentally screened drugs, 833 were approved, with the remaining 85 drugs distributed between investigational, experimental, nutraceutical, and withdrawn. Our problem, however, focuses on ranking approved drugs exclusively. Nevertheless, we thought it was interesting to compare the performance of our network medicine approach with Gysi et al.'s AI based methods. We did this by running our network medicine approach to rank the 918 drugs used in their evaluation. For a fair comparison, we used the same dataset than Gysi et al.[38], as described below.

- PPI with 18,505 proteins and 327,924 interactions.
- 24,648 associations between 6253 and 3910 targets used by Gysi et al.[38]. The dataset contains data from DrugBank (`https://go.drugbank.com/`), and other additional 25 drugs with targets curated by the literature.
- 332 host proteins identified by Gordon et al.[49].
- A set of 918 drugs (E918) that were tested *in vitro* against SARS-CoV-2 on Vero E6 cell lines[38].
- Evaluation set of drugs with *in vitro* evidence, which contains 77 compounds with weak or strong evidence on the *in vitro* experiment (we refer to this evaluation setting as E918-IV, see Table S4).
- Evaluation set of drugs with clinical trials evidence, which contains 37 drugs that are both in E918 set and clinical trials of April 15th 2020 (we refer to this evaluation setting as E918-CT, see Table S4).

In addition, we used drugs predicted by Connectivity Map (CMAP)[44,45] as an additional source of evidence. For running CMAP, we used the consensus dataset by Ghandikota et al.[46] as the COVID-19 signature, with genes that are up- or down-regulated in different *in vitro* and *in vivo* models of SARS-CoV-2 infections. Our query on CMAP resulted in 19 drugs with significant CMAP τ score (< -90) that overlap with the list of 918 drugs tested *in vitro* (we refer to this evaluation setting as E918-CM, see Table S4).

We evaluated the performance of our network medicine methods and competitors on different types of evidence for COVID-19: E918-IV (*in vitro* evidence), E918-CT (clinical trials), and CMAP evidence (E918-CM). Our approach includes five different kernels (commute time, diffusion, inverse cosine, p-Step with $p = 2$, and regularised Laplacian), and an aggregated ranking (avgRank). As competitors. we considered methods used by Gysi et al.[38], which includes AI-based methods (based on Graph Neural Networks - GNN), diffusion-based methods (variations of DSD[47]), proximity-based methods (variations of the Guney's distance[48]), and cRank[REF] (a combination of all the rankings). We then computed the same evaluation metrics as Gysi et al., that is, the AUC-ROC (area under the ROC curve), recall at top 100, and precision at top 100). These results are shown in Figure S8.

We observed that for all evaluation datasets, our approach has an AUC-ROC higher than the diffusion-based methods, and similar or higher than the proximity based methods. We also have a higher AUC-ROC than the AI based methods in all types of evidence except for clinical trials (E918-CT). Our performance is always higher than a random classifier (AUC-ROC > 0.5). In contrast, A4 (AI-based method), and the diffusion based methods (variations of DSD) have an AUC below 0.5 for CMAP evidence, and D3 and D5 (variations of DSD) have an AUC-ROC below 0.5 for *in vitro* evidence.

We also observed that our matrix decomposition model predicts earlier drugs with *in vitro* or CMAP evidence. As shown in Figure S8, we have the largest recall and precision at top 100 for these sets of drugs. The only scenario in which we do not have the higher AUC-ROC nor the higher recall is for predicting drugs in clinical trials on April 15th 2020. In this case, the AI-based methods (GNN) have the best performance. Nevertheless, in this scenario our approach has the highest recall and precision compared to all proximity and diffusion based methods.

Finally, in Figures S9, S10, and S11, we compare prediction scores of drugs on each evaluation set (E918-IV, E918-CT, and E918-CM) in terms of the Wilcoxon-Mann-Whitney p-values. In the work by Gysi et al.[38] the pre-computed embeddings are available only for the AI-based methods. For the other methods, only the rankings are available. Therefore, we computed the raw scores of a proximity based method (the Guney distance[48]) and a diffusion based method (the DSD method[47]). We used the original implementation of these two methods, and due to considerable computational costs, we used the Guney distance[48] as the representative for all the Proximity based methods, and DSD[47] as the representative for all the diffusion based methods. We observed that our approach (based on kernels) and DSD are the only methods with significant p-values in all evaluation sets. Notably, the AI based methods are only aligned with the clinical trial data, but not to the other two sources of evidence.

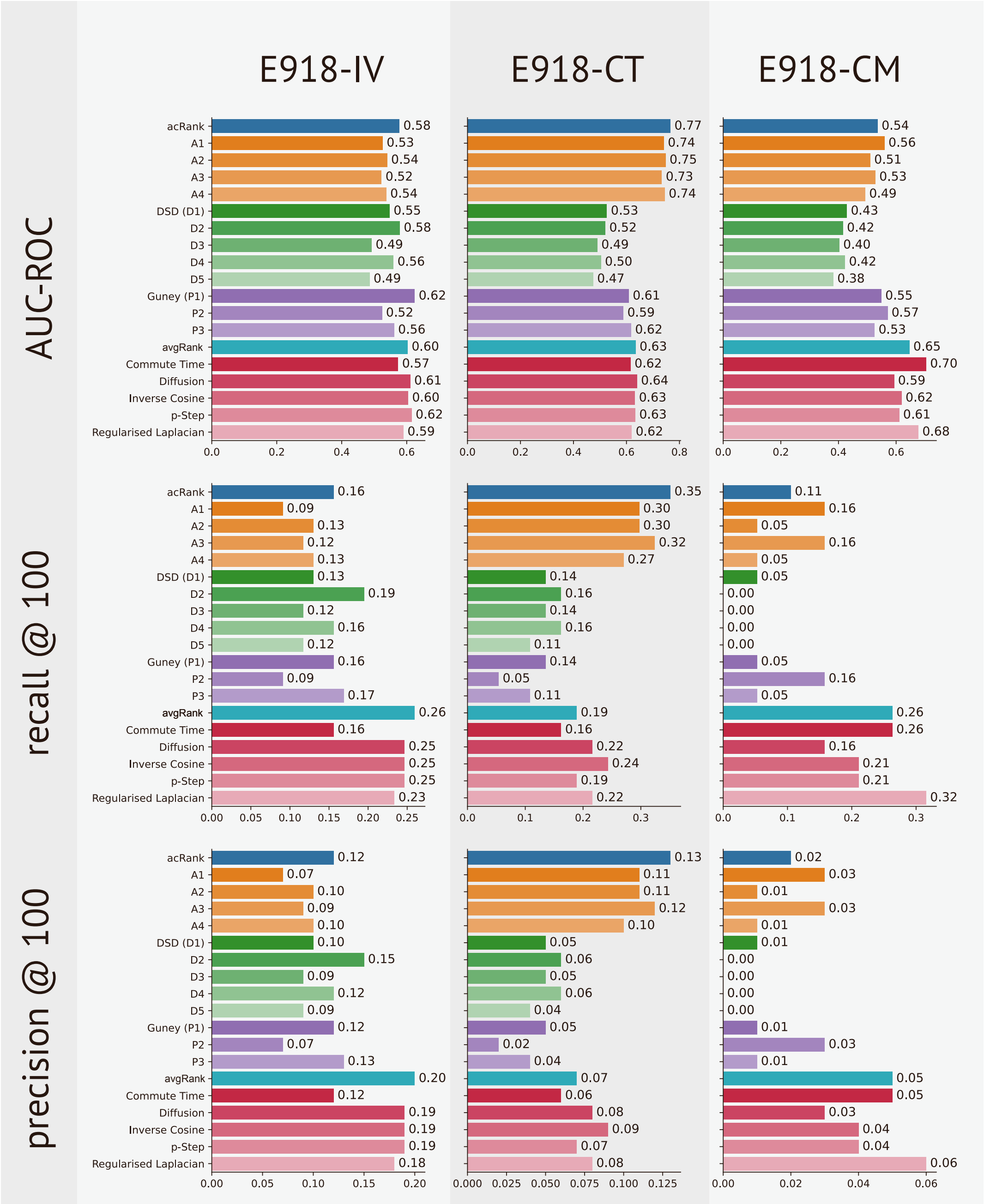

Figure S8. Performance comparison of our methods to the methods used in Gysi et al.[38]. We observe that our network medicine approach is competitive across all evidence types with the Proximity based methods (based on the Guney distance), as well as the diffusion methods (based on the DSD method). In these cases, our network medicine approach is competitive in the AUC-ROC metric, but stronger in the precision and recall metrics. When compared to the GNN based methods by Gysi et al. , we see that the AI based methods perform quite well on the clinical trial evidence, but an overall lower performance on other types of evidence.

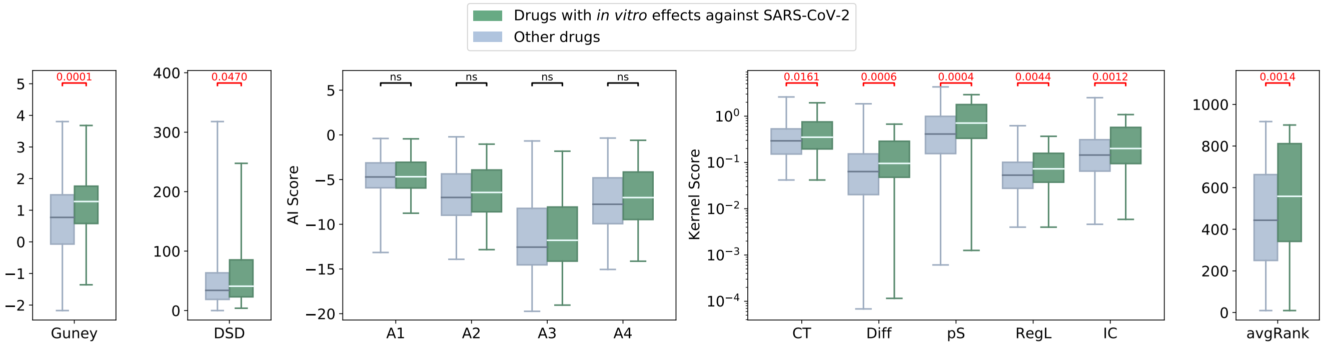

Figure S9. Score comparison between our network medicine approach, and methods used in Gysi et al.[38] on the E918-IV (*in vitro*) evaluation set. Notably, none of the AI methods assign significantly different scores to drugs with *in vitro* effect vs. all other drugs in this evaluation set. The Guney distance, the DSD method, the average ranking (avgRank), and all of our network medicine approach methods do align to this type of evidence.

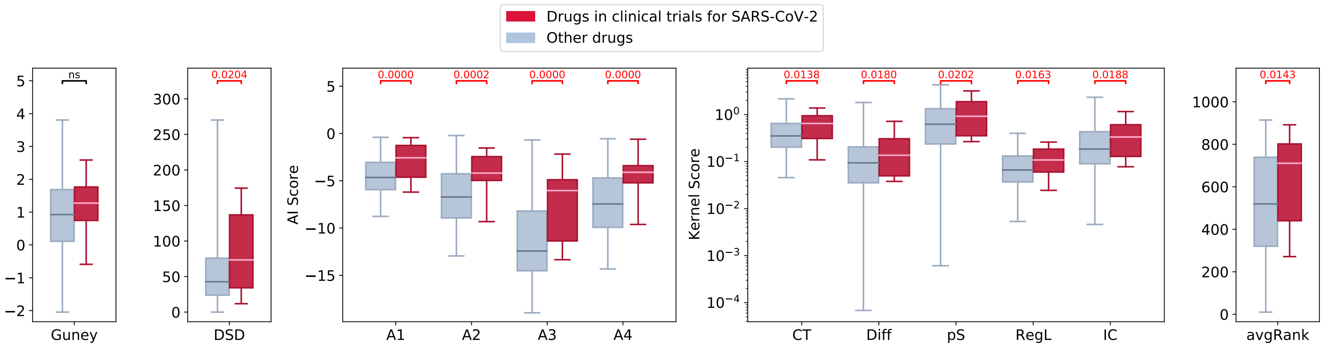

Figure S10. Score comparison between our network medicine approach, and methods used in Gysi et al.[38] on the E918-CT (clinical trials) evaluation set. For this evaluation setting, with the exception of the Guney distance, every method assigns significantly different scores to drugs in Clinical trials on April 15th, 2020.

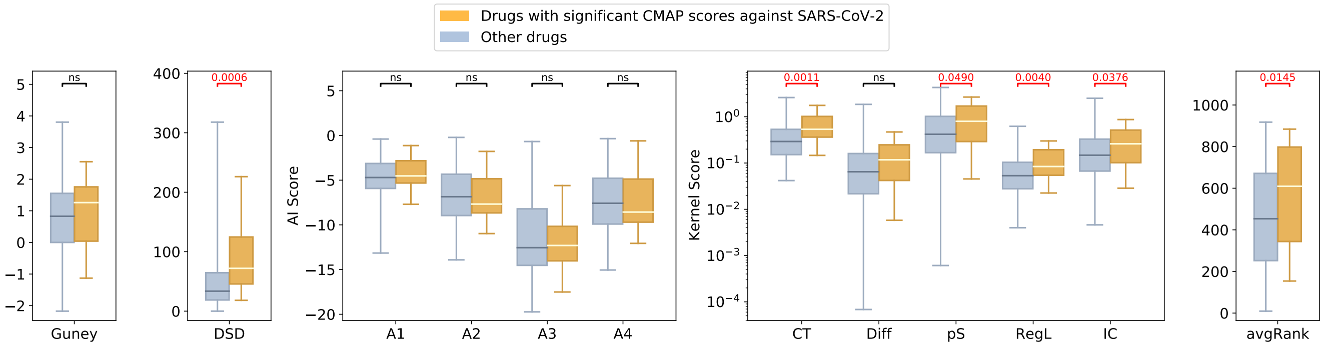

Figure S11. Score comparison between our network medicine approach, and methods used in Gysi et al.[38] on the E918-CM evaluation set. For this evaluation setting, the AI is also unable to assign significantly different scores to drugs with significant CMAP scores. This is also the case for the Guney distance, and our network medicine score calculated on the Diffusion kernel.

7 Note S7. Evaluation of the network medicine approach on additional PPIs

We evaluated the network medicine approach on two additional interactomes: the Cheng et al.[40] PPI, and the HuRI PPI[41], as described in Table S3. We considered 1853 FDA-approved drugs with targets in these interactomes. Out of the 1853 drugs, 78 have *in vitro* evidence (IV-1853 set), 153 have clinical trials evidence (CT-1853 set), and 21 have CMAp evidence. Details on our evaluation dataset are described in Section 4.

In Figure S12, we compare prediction scores between drugs with evidence for COVID-19 and the remaining drugs, on the different PPIs and types of evidence. We also show the corresponding Wilcoxon-Mann-Whitney p-values. Compared to the competitors (DSD[47], and the Guney distance[48]), our network medicine is the only one with predictions significantly aligned with *in vitro*, and CMAP evidence in all PPIs.

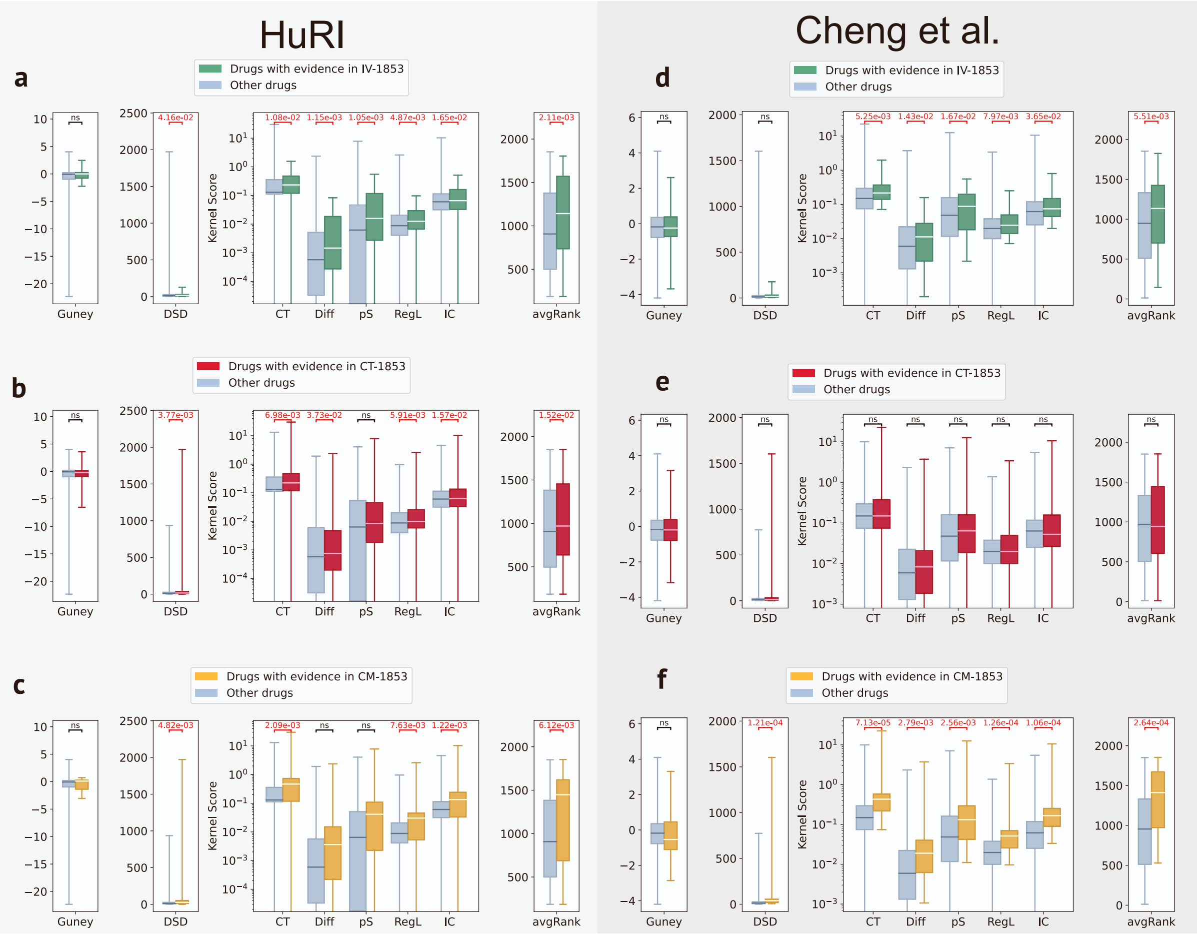

Figure S12. Evaluation of the network medicine approach on additional interactomes. We used boxplots to compare prediction scores between drugs with and without evidence for COVID-19 on three different evaluation sets: *in vitro* evidence (IV-1853), clinical trials evidence (CT-1853), and CMAP evidence (CM-1853). For each comparison, we computed the Wilcoxon-Mann-Whitney p-value (one tailed test). Non-significant p-values (below 0.05) are indicated with 'ns'. Significant p-values are shown in red. We show results obtained by our network medicine approach and competitors. Our approach includes five different kernels on graphs (commute time, diffusion, inverse cosine, p-step kernel, and regularised Laplacian), and a combined ranking across the kernels (avgRank). The competitors include DSD[47], and the Guney distance[48].

The only scenario in which our approach does not have a significant alignment is when we evaluate predictions for clinical trials evidence on the Cheng et al. PPI. In this case, none of the competitors had significant p-values. Notice that, however, in the HuRI PPI prediction scores are significantly different between drugs on clinical trials and the remaining drugs for both our approach and DSD.

It is also interesting to see how our network medicine approach and the competitors perform with traditional machine learning metrics. We show the area under the ROC curve (AUC-ROC), recall at top 150, precision at top 150, and MAP for the Cheng et al., and HuRI PPIs in Figures S13, and S14 respectively. We noticed that in most scenarios the kernel-based

methods have the highest values of AUC-ROC, and MAP. However, even when the AUC-ROC, and MAP are not the highest, our approach predicts earlier on the top 150. That is, in every case, our network medicine approach has the highest recall and precision at the top 150.

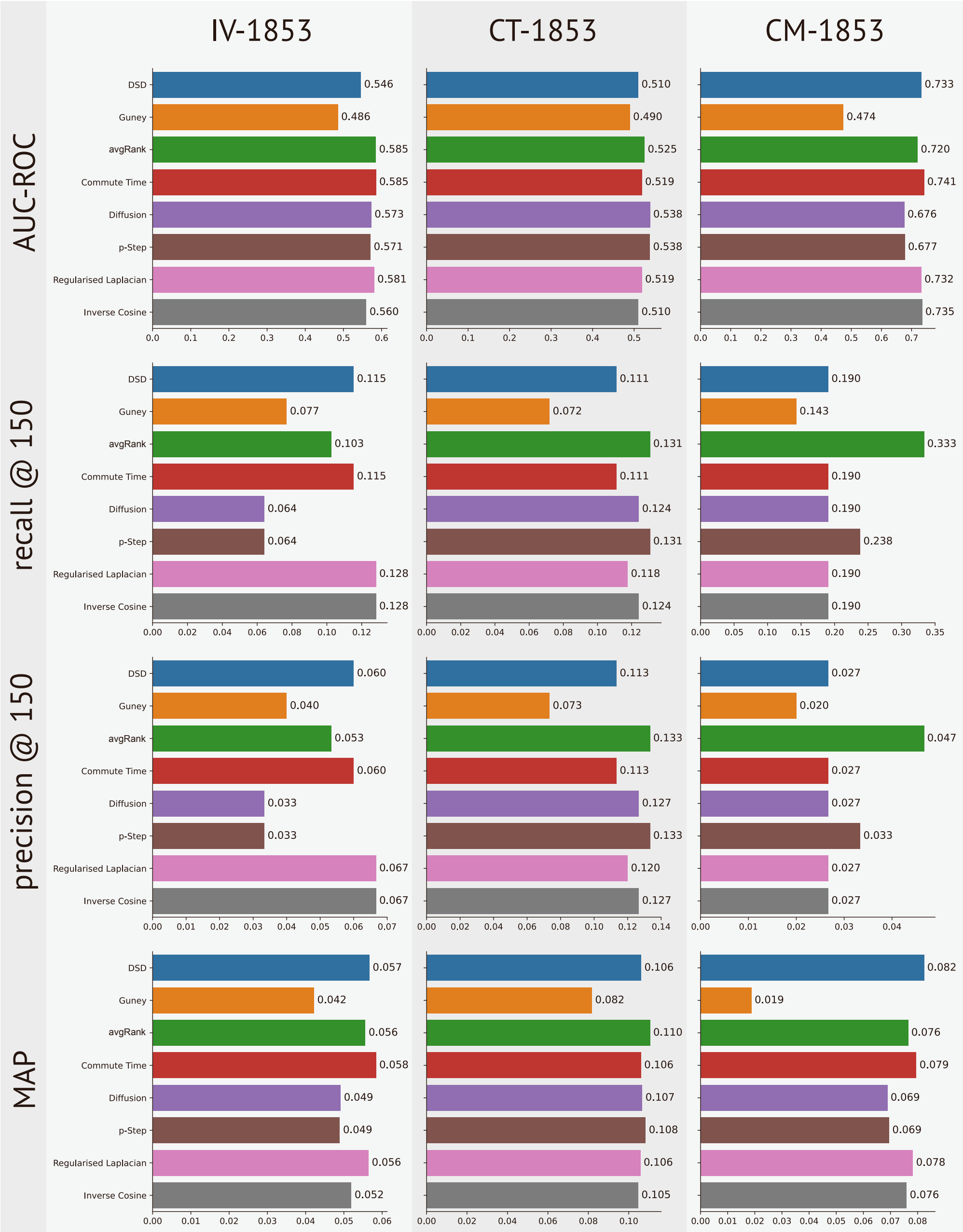

Figure S13. Classical machine learning metrics obtained by the network medicine approach on Cheng et al. PPI. We computed the area under the ROC curve (AUC-ROC), recall at top 150, precision at top 150, and mean average precision (MAP) for three different evaluation sets. They correspond to *in vitro* evidence (IV-1853), clinical trials evidence (CT-1853), and CMAP evidence (CM-1853). For each type of evidence, we show results obtained by our network medicine approach and competitors. Our approach includes five different kernels on graphs (commute time, diffusion, inverse cosine, p-step kernel, and regularised Laplacian), and a combined ranking across the kernels (avgRank). The competitors include DSD[47], and the Guney distance[48].

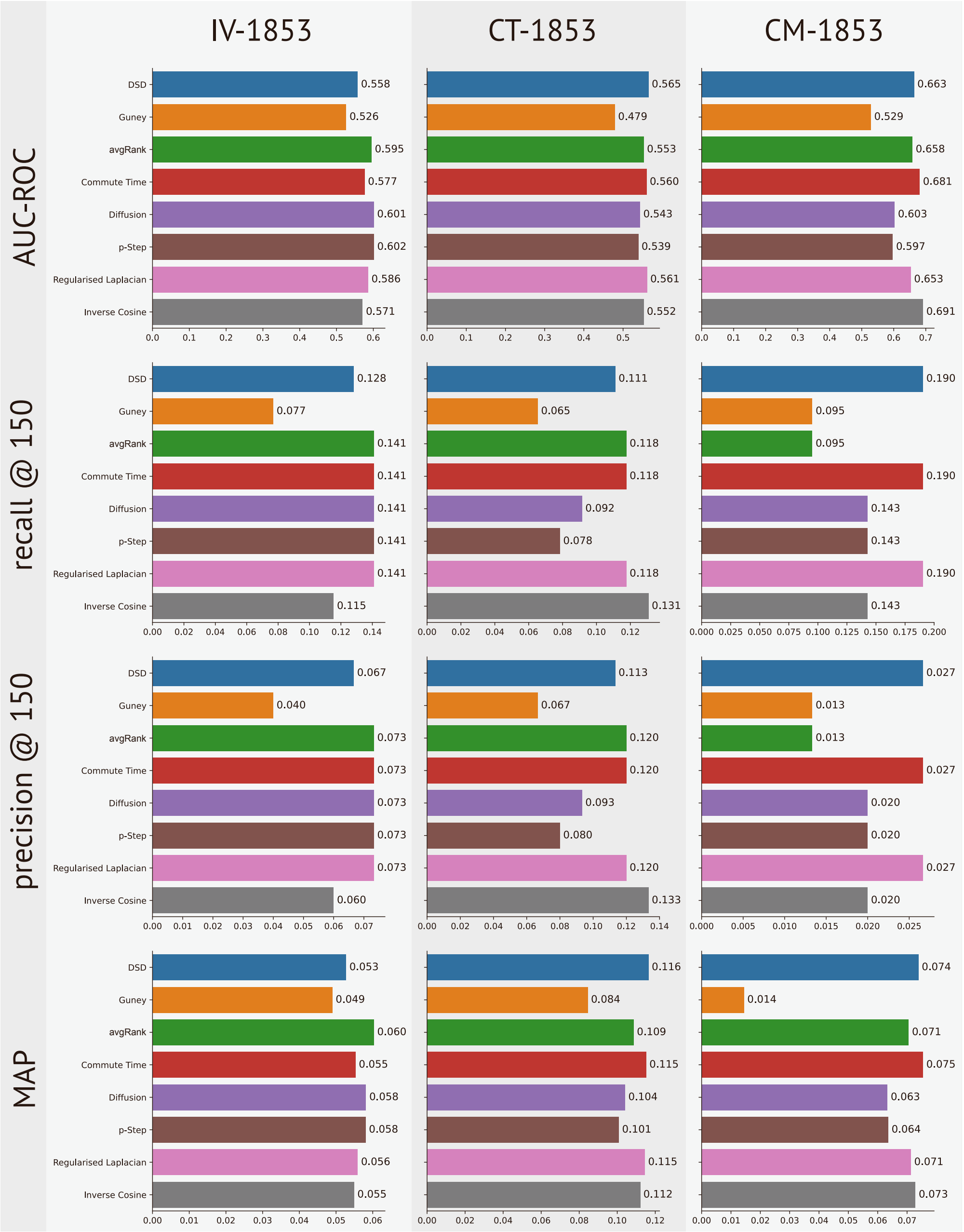

Figure S14. Classical machine learning metrics obtained by the network medicine approach on HuRI et al PPI. We computed the area under the ROC curve (AUC-ROC), recall at top 150, precision at top 150, and mean average precision (MAP) for three different evaluation sets. They correspond to *in vitro* evidence (IV-1853), clinical trials evidence (CT-1853), and CMAP evidence (CM-1853). For each type of evidence, we show results obtained by our network medicine approach and competitors. Our approach includes five different kernels on graphs (commute time, diffusion, inverse cosine, p-step kernel, and regularised Laplacian), and a combined ranking across the kernels (avgRank). The competitors include DSD[47], and the Guney distance[48].

Therefore, similarly to results with the Gysi PPI[38] used throughout the manuscript, our predictions on additional PPIs are the most consistently aligned with the three sources of evidence for COVID-19 (*in vitro*, clinical trials, and CMAP).

8 Note S8. Integration of gene expression for network medicine

We compared results obtained with the binary and weighted versions of the network medicine approach on our three evaluation sets (*in vitro*, clinical trials, and CMAP).

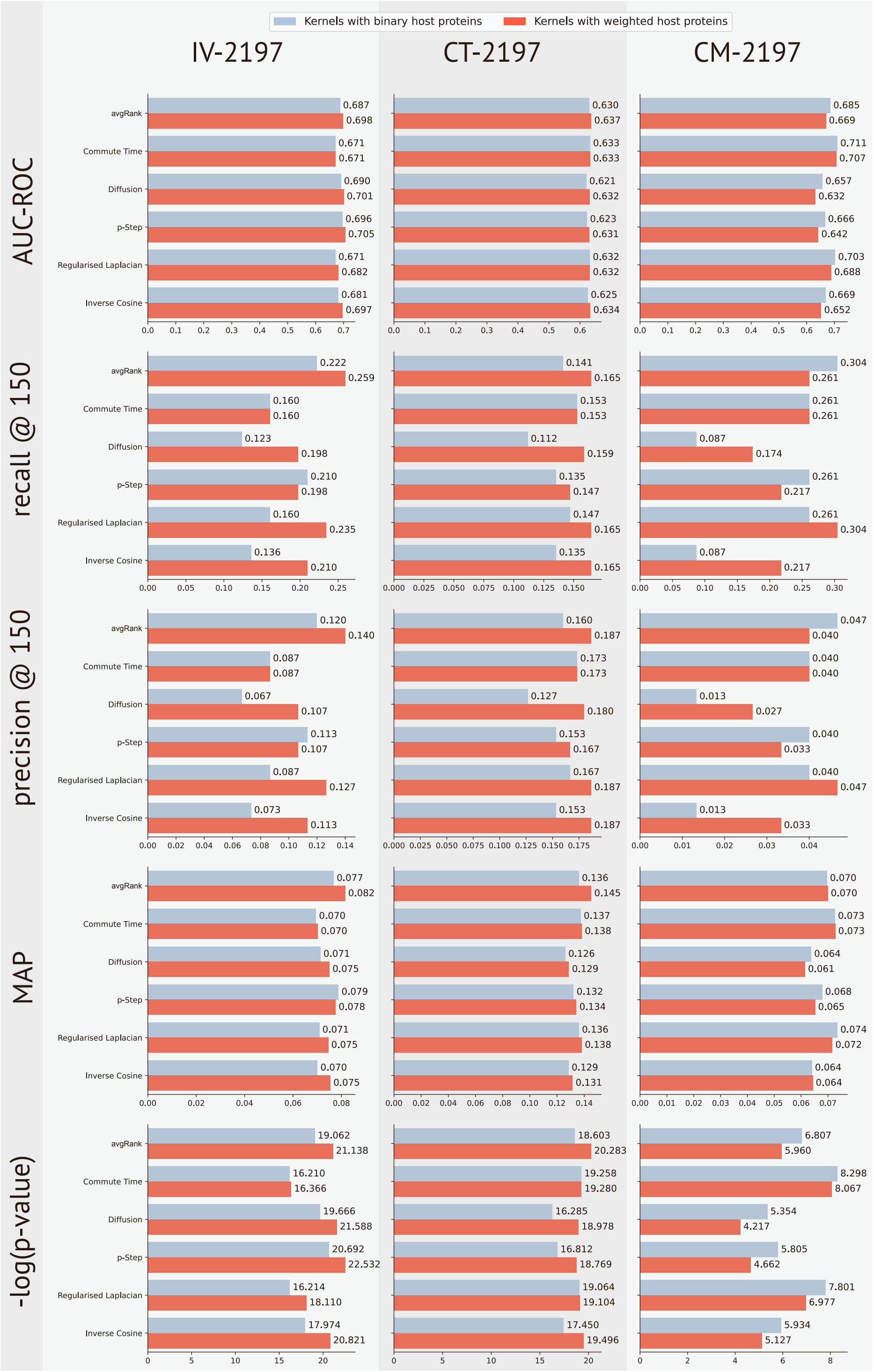

Figure S15. Comparison between binary and weighted kernel scores. We observe consistent improvement on the results of the weighted version for *in vitro* (IV-2197) and clinical trial (CT-2197) evidence. For CMAP (CM-2197), the AUC-ROC, MAP, and p-value metrics are comparable, while a notable improvement is observed in the recall and precision at top 150 metrics. Notice that we show the negative logarithm of the p-values. Thus, the higher the value, the more significant the p-value is.

Figure S15, Figure S16 and Table S5 present, for each evaluation set, four different machine learning metrics for evaluating the performance (AUC-ROC, recall@top 150, precision@top 150, MAP); as well as, and the Wilcoxon-Mann-Whitney p-value obtained comparing prediction scores between set of drugs (as described in the paper).

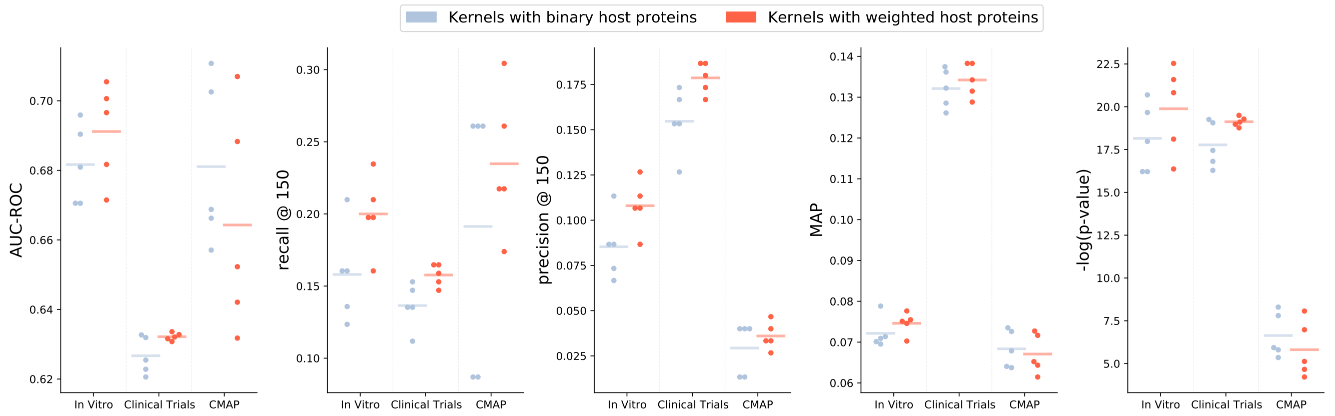

Figure S16. Distribution of five different performance metrics (y-axis) across the five kernels used by the network medicine approach. Each point corresponds to an evaluation metric obtained by a kernel on a specific type of evidence (x-axis). The horizontal lines correspond to the average values. When comparing the binary vs. weighted versions of the kernel methods, we observe a consistent improvement in the weighted version for all performance measures on *in vitro* and Clinical Trials evidence. For CMAP, the AUC-ROC, MAP, and p-value metrics are comparable, while a notable improvement is observed in the recall and precision at top 150 metrics. Notice that we show the negative logarithm of the p-values. Thus the higher the value, the more significant the p-value is.

Evidence	Metric	Mean		Standard deviation	
		Binary	Weighted	Binary	Weighted
In Vitro	recall @ 150	0.169	**0.210**	0.040	0.034
	precision @ 150	0.091	**0.113**	0.021	0.018
	MAP	0.073	**0.076**	0.004	0.004
	AUC	0.683	**0.692**	0.010	0.013
	-log(p-value)	18.303	**20.093**	1.844	2.351
**Clinical Trials**	recall @ 150	0.137	**0.159**	0.014	0.007
	precision @ 150	0.156	**0.180**	0.016	0.008
	MAP	0.133	**0.136**	0.005	0.006
	AUC	0.627	**0.633**	0.005	0.002
	-log(p-value)	17.912	**19.318**	1.240	0.535
**CMAP**	recall @ 150	0.210	**0.239**	0.097	0.046
	precision @ 150	0.032	**0.037**	0.015	0.007
	MAP	**0.069**	0.068	0.004	0.005
	AUC	**0.682**	0.665	0.021	0.029
	-log(p-value)	**6.667**	5.835	1.181	1.469

Table S5. Average and standard deviation of evaluation metrics across the five different kernels (commute time, diffusion, inverse cosine, p-step, and regularised Laplacian). The largest mean values are highlighted in bold. We compared results between kernel-based methods with (weighted) and without (binary) weights on the host proteins. We observed a consistent improvement in the weighted version for all performance measures on *in vitro* and Clinical Trials evidence. For CMAP, the AUC, MAP, and p-value metrics do not show improvement, while a notable increase is observed in the recall and precision at top 150 metrics. Notice that we show the negative logarithm of the p-values. Thus the higher the value, the more significant the p-value is.

Our results show that the AUC-ROC and MAP increase, and p-values became more significant in the weighted version for

both *in vitro* and clinical trials evidence. Importantly, the gene expression data is helping in retrieving drugs with evidence earlier in our ranking.

In fact, there is a consistent increase in the recall and precision for all types of evidence in the weighted version. On average, the recall increased 31%, 16.9%, and 50% for *in vitro*, clinical trials and CMAp evidence, respectively. Similar improvements can be seen for the precision. Notice that for the CMAP evaluation, recall and precision became higher even for kernels for which the AUC-ROC, and MAP did not increase, as shown in Figure S15. The AUC-ROC increases on average 1.4% and 0.9% on *in vitro* and clinical trials, respectively. Proportionally, MAP increases more by incorporating gene expression data into the network medicine approach. It has an average increase of 4.1% for *in vitro* evidence, and 1,3% for clinical trials evidence.

9 Note S9. Overlap between our predictions and predictions by Gordon et al

Gordon et al. identified 69 compounds that target 66 out of the 332 host proteins used by SARS-CoV-2 infection and replication[49]. A subset of these and 28 additional compounds were screened in viral assays[49]. Two sets of pharmacological agents displayed antiviral activity: inhibitors of mRNA translation and predicted regulators of the sigma-1 and sigma-2 receptors.

Altogether, the analyses by Gordon et al. resulted in four lists of candidate drugs against COVID-19, described below:

Set 1 **(Chemoinformatics)** 37 compounds identified by chemoinformatics searches.

Set 2 **(Literature)** 32 compounds identified by target- and pathway-specific literature search.

Set 3 **(Multiple)** 75 compounds tested for *in vitro* activity against SARS-CoV-2. It contains a subset of 47 compounds identified by 1 and 2, plus 13 to expand testing of the sigma-1 and sigma-2 receptors and mRNA translation targets, and 15 additional molecules that had been prioritized by other methods.

Set 4 **(In vitro)** a subset of 3, consisting of 13 compounds with *in vitro* activity against COVID-19.

Some of our top predictions include compounds in these lists. In the top 20, our matrix decomposition approach predicts Chloroquine (set 1), Azithromycin (set 3), Nitazoxanide (set 3), and Hydroxychloroquine (set 4); and our network medicine approach predicts Progesterone (set 3).

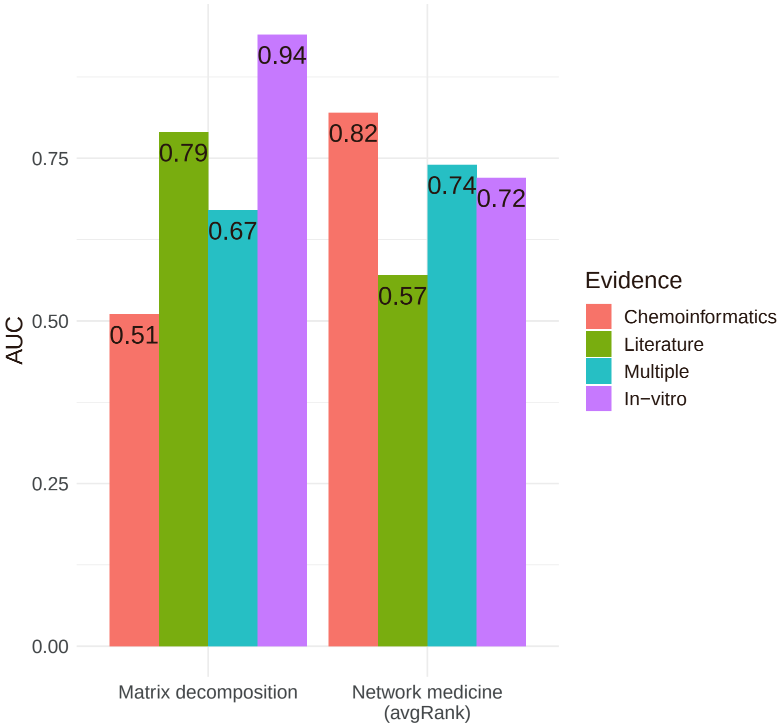

Figure S17. Area under the ROC curve for predicting drugs identified by Gordon et al.[49], which are classified into four different sets. The first set (Chemoinformatics) includes drugs identified by chemoinformatics search. The second set (Literature) contains compounds found by target- and pathway-specific literature search. The third set has compounds identified by multiple sources (Multiple), including chemoinformatics, literature, and other methods. Finally, the last set (*in vitro*) includes a subset of the third set with *in vitro* activity in the assays by Gordon et al.[49].

Another interesting question is how the entire set of drugs selected by Gordon et al. is ranked across our predictions. To answer this question, we formulated an equivalent binary classification problem, so that we could use ROC curve analysis. For each set of drugs, we labelled the compounds within the set as "positive", and the remaining ranked drugs as "negative". Thus, for the matrix decomposition approach, our dataset with 126 drugs contains 5 compounds from set 1, 3 drugs from set 2, 10

drugs from set 3, and 1 drug from set 4 that we labelled as positive. For the network medicine approach, our dataset of 2197 approved drugs, contains 14 drugs from set 1, 7 from set 2, and 27 from set 3, and 3 drugs from set 4. Results are shown in Figure S17, which presents the AUC ROC for our two approaches, separately on the 4 different sets of drugs.

Our matrix decomposition model has a better alignment with predictions by Gordon et al.[49] for drugs with *in vitro* efficacy in theirs assays ("*in vitro*" - set 4), compounds identified by target- and pathway-specific literature ("Literature" - set 2), and multiple sources of evidence ("Multiple", set 3). This is in accordance with the data used by this approach, which contains associations between viruses and drugs based on the literature.

For the network medicine approach, we show the results obtained by the avgRank model, which has an AUC larger than 0.5 for all sets identified by Gordon et al.[49]. Our network medicine approach is best at prioritizing drugs identified by chemoinformatics search and a possible reason for this is that these drugs often target a host factor or its close by neighbours.

10 Note S10. CoREx Network Combination

We begin by using InterPro[50] predictions $R \in \mathbb{R}^{n \times t}$ for n proteins and t GO terms. We then build a network of functional similarities T, defined as:

$$T_{ij} = \frac{|N_i \cap N_j|}{|N_i \cup N_j|}$$

where N_i and N_j are the sets of all GO terms above a threshold τ that are associated to proteins i and j respectively in R, that is, $N_i = \{k | R_{ik} > \tau\}$, and $N_j = \{k | R_{jk} > \tau\}$. Therefore, T_{ij} is the Jaccard similarity between sets of GO terms that are assigned by InterPro to proteins i and j.

Given p networks $W^{(r)}$ with $r \in (1 \cdots p)$, we combine them into a single network W using a weighted linear combination. The vector of weights $\hat{c} \in \mathbb{R}^p$ is learnt by minimising the square of the difference between T and the linear combination:

$$(\hat{c}, \hat{b}) = \underset{c,b}{\operatorname{argmin}} \sum_{i,j} \left(b + \sum_{r=1}^{p} c_r W_{ij}^{(r)} - T_{ij} \right)^2$$

and $\hat{b}$ is used to remove the bias in T. This linear regression can be solved efficiently, and we can interpret each learnt coefficient c_r as representing how much each network r contributes to the combination.

11 Note S11. Analysis of the top predictions by the two approaches

11.1 Matrix decomposition approach

Tables S1 and S2 show the top predicted drugs using our matrix decomposition approach. Table S2 shows predictions of type A — drugs under development, but not approved, for SARS-CoV-2 according to the Andersen et al.[14] dataset (their developmental stages were used for training).

We observed that these predicted BSA drugs are known to interfere with SARS-CoV-2 infection through different molecular mechanisms. The nucleotide analogue inhibitors Remdesivir[51] and Fapinavir[52] inhibit the RNA-dependent RNA polymerase (RdRp) of SARS-CoV-2. Lopinavir/ritonavir is a protease inhibitor, which may inhibit the 3Clike protease of SARS-CoV-2, and Chloroquine/Hydroxychloroquine might interfere with the replication of SARS-CoV-2 by multiple mechanisms[53]. Arbidol has been found effective against SARS-CoV-2 *in vitro* by inhibiting viral attachment and release of SARS-CoV-2 from intracellular vesicles[30].

The drug with the highest predicted score was Favipiravir, already known to be in phase II of clinical trials for COVID-19 according to the Andersen et al. dataset. Favipiravir is a RNA polymerase inhibitor that was developed in Japan as an antiviral treatment for influenza. In October 2020, already 37 clinical trials were registered in ClinicalTrials.gov to assess its efficacy and safety for COVID-19 patients[26]. A systematic review of the randomised clinical trials suggest that Favipiravir have shown low efficacy for patients with mild or moderate COVID-19[26]. In fact, other drugs in Table 2, including Remdesivir, Lopinavir and Hydroxychloroquine have shown little to no clinical efficacy in COVID-19 patients according to the WHO Solidarity Trial, conducted in 405 hospitals in 30 countries[33].

However, although several of these BSA drugs have been found effective *in vitro* or in vivo against SARS-CoV-2, many of them have proven with little to no efficacy in clinical trials in COVID-19 patients. For instance, our drug with the highest predicted score is Favipiravir, already known to be in phase II of clinical trials for COVID-19 according to the Andersen et al.[14] dataset. In October 2020, already 37 clinical trials were registered in ClinicalTrials.org to assess its efficacy and safety for COVID-19 patients[26]. A systematic review of the randomised clinical trials suggests that Favipiravir has shown low efficacy for patients with mild or moderate COVID-19[26]. In fact, other drugs in Table S2, including Remdesivir, Lopinavir and Hydroxychloroquine have shown little to no clinical efficacy in COVID-19 patients according to the WHO Solidarity Trial, the largest clinical trial conducted in more than 11,000 patients across 405 hospitals in 30 countries[33].

The lack of efficacy of BSA drugs that were under development at the time of release of the Andersen et al. dataset[14] suggests that it is important to consider predicted BSA drugs that were not yet under development against SARS-CoV-2 — i.e., our predictions of type B.

Our top predicted drugs of type B, not under development for SARS-CoV-2 according to the Andersen et al.[14] dataset, are shown in Table S1. Overall, we found that several of these drugs are already in clinical trials for COVID-19. We comment more in detail on a few entries in the table.

Tenofovir, our top predicted drug, is a broad-spectrum antiviral drug active against HIV and hepatitis B, and it was found to interfere with the SARS CoV-2 ribonucleic acid (RNA)-dependent RNA polymerase (RdRp), an enzyme indispensable for SARS-CoV-2 replication, in experiments *in vitro*[54], and *in vivo*[55]. In addition, a recent phase II clinical trials in two hospitals in France show that Tenofovir, in combination with Emtricitabine, accelerated the natural clearance of nasopharyngeal SARS-CoV-2 viral burden[56].

Lamiduvine is the second predicted BSA drug in Table S1. The potential of repurposing Lamivudine for COVID-19 is currently under investigation. There is evidence that indicates that Lamiduvine might work as an inhibitor of the SARS-CoV-2 RdRp RNA polymerase[57]. Yet, further experiments *in vitro* and *in vivo* are needed to confirm this hypothesis.

Interestingly, we also predicted the anti-malarial drug Artesunate, which has been found effective against SARS-CoV-2 in several *in vitro* assays[58]. In fact, Artesunate is one of the three drugs that have been selected for the currently ongoing WHO Solidary Plus Clinical Trials to assess its efficacy in COVID-19 patients[25].

Rank	Drug name (ID)	Main ATC Category	Additional curated evidence for COVID-19
1	Fostamatinib (DB12010)	Blood and blood forming organs (B)	several clinical trials (NCT04579393, NCT04581954, NCT04629703), *in silico* evidence[59]
2	NADH (DB00157)		in Clinical trials (NCT04604704), *in silico* evidence[60–62]
3	Copper (DB09130)		Combinatorial therapy[63], *in silico* evidence[64]
4	Cannabidiol (DB09061)	Nervous System (N)	In Clinical Trials (NCT04467918)
5	Glutathione (DB00143)	Various (V)	In Clinical Trials CTRI/2021/01/030793
6	Doxorubicin (DB00997)	Antineoplastic and immunomodulating agents (L)	*in silico* evidence[65]
7	Flavin adenine dinucleotide (DB03147)		*in silico* evidence[64]
8	Verapamil (DB00661)	Cardiovascular System (C)	multiple clinical trials (NCT04351763, NCT04330300, NCT04467931)
9	Zinc (DB01593)	Cardiovascular System (C)	included in more than 60 clinical trials, *in silico* evidence[64]
10	Zinc acetate (DB14487)	Cardiovascular System (C), Alimentary tract and metabolism (A)	included in more than 60 clinical trials, *in silico* evidence[64]
11	Zinc chloride (DB14533)	Blood and blood forming organs, Cardiovascular System (B)	included in more than 60 clinical trials, *in silico* evidence[64]
12	Moexipril (DB00691)	Cardiovascular System (C)	in clinical trials (NCT04467931)
13	Conjugated estrogens (DB00286)	Genito-urinary system and sex hormones (G)	NA
14	Clozapine (DB00363)	Nervous System (N)	NA
15	Rifampicin (DB01045)	Antiinfectives for systemic use (J)	*in silico* evidence[66]
16	Amitriptyline (DB00321)	Nervous System (N)	*in vitro* evidence[38]
17	Phenobarbital (DB01174)	Nervous System (N)	NA
18	Desipramine (DB01151)	Nervous System (N)	NA
19	Progesterone (DB00396)	Genito-urinary system and sex hormones (G)	in several clinical trials (NCT04365127, NCT04539626, NCT04865029 - combinatorial therapy)
20	Ethanol (DB00898)	Dermatologicals (D), Various (V)	in more than 100 clinical trials.

Table S6. Kernel-based top-20 predicted drugs. For each drug we show whether there is evidence from other *in silico* approaches, *in vitro* experiments, or clinical trials.

11.2 Network medicine approach

To obtain the scores for the 2197 FDA-approved drugs, we used the average ranking (avgRank) across the five different graph kernels. In our calculations, we used the interactome provided by Gysi et al.[38], and the SARS-CoV-2 host-proteins were weighted in our model using gene expression data from nasopharyngeal swabs[67] (GEO:GSE152075).

We started by analysing whether there was a trend among the top predicted drugs. Figure S18 below shows the distribution of main Anatomical, Therapeutic and Chemical (ATC) classification of the top-20 predicted drugs. We observed that half of the predicted drugs belong to either Nervous System (N) or Cardiovascular System (C) drugs. In fact, we found an enrichment of nervous system drugs (psychoanaleptics, N06) and cardiovascular system drugs (vasoprotectives, C05) amongst the top-100 predictions (p = 0.01 and p = 0.03, respectively, hypergeometric Test of Significance with FDR multiple testing correction).

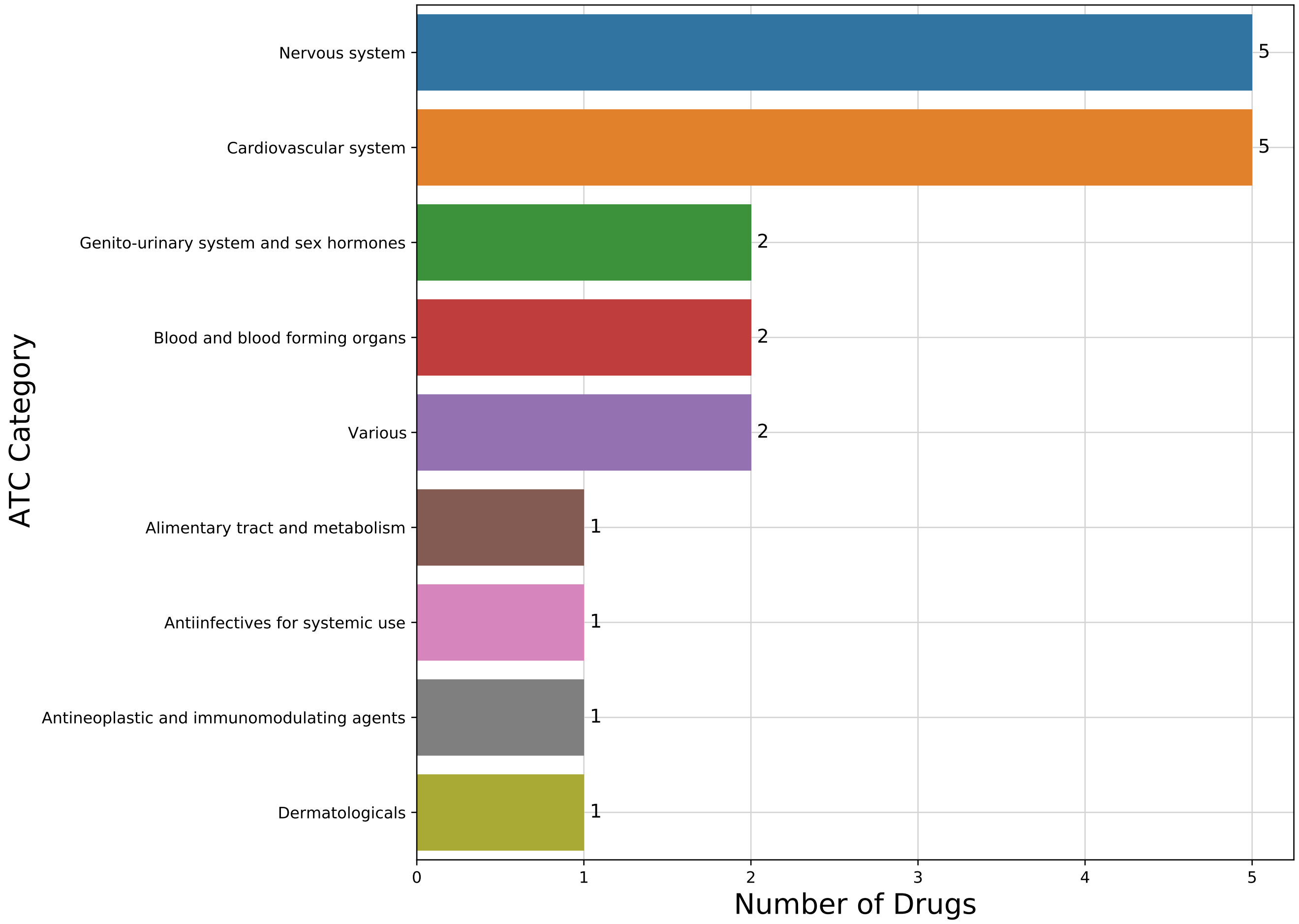

Figure S18. Distribution of main Anatomical, Therapeutic and Chemical (ATC) categories of the top-20 predicted drugs with our network medicine approach

We further analysed each of these top-20 predicted drugs in more detail (see Table S6).

The top scoring drug is Fostamatinib, that was identified as a candidate for rapid repurposing for COVID-19 patients through a high content screen for Mucin-1 (MUC1) reducing compounds[68]. Elevated levels of MUC1 predict the development of Acute Lung Injury (ALI). By its MUC1 reducing effect, the potential of Fostamatinib to manage the acute respiratory distress syndrome (ARDS), often fatal in COVID-19 patients is highglighted[68]. Furthermore, there is evidence that Fostamatinib counteracts the antiinflammatory response caused by the anti-spike IgG, instrumental in the worsening of COVID-19 cases[69].

Our top predictions also include drugs that interact with the SARS-CoV-2 host receptor protein, angiotensin-converting enzyme 2 (ACE2) and/or the ACE2/TMPRSS2 pathway[70,71]. For example, cannabidiol (top 4) has been shown to modulate ACE2 expression in COVID-19 gateway tissues, providing scientific rationale for clinical trials[72]. Conjugated estrogens (top 13) are linked to the reduced effect of SARS-CoV-2 on females[73], and there is promising evidence of using them as therapeutics for COVID-19 patients through their interaction with the ACE2/TMPRSS2 pathway[73,74]. Another drug used in hormone therapy that is on our top predictions is Progesterone. It shows promising therapeutic characteristics to improve the immune dysregulation that leads to the COVID-19 cytokine storm[75].

Another interesting group of drugs in Table S6 is zinc, zinc acetate, and zinc chloride (top 9, 10, and 11 respectively). Noticeably, most risk groups described for COVID-19 are associated with zinc deficiency[76], and there is encouraging evidence that zinc supplements are helpful to prevent pathogen entry[76]. Treatment with zinc acetate and zinc gluconate have also accelerated the recovery of COVID-19 patients[77], and they are linked to rapid resolution of COVID-19n shortness of breath[78].

In the ranking, there are drugs that have been found useful in other applications for COVID-19. For instance, vitamin supplements ingredients NADH (top 2), and Flavin adenine dinucleotide (top 7) have been identified as potential biomarkers for fluorescence, label free detection of COVID-19 on early stages employing portable optical detection systems[79]. There is evidence suggesting that copper (top 3) in nasal lavages might be both safe and could be used to reduce or even stop viral transmission[80].

It is important to note that our network medicine approach works by modelling the mechanistic effects on the interactome. This means that a high score points to a high probability of molecular interplay between the drug and the COVID-19 disease module. However, while our kernel methods, like most network approaches[38], can quantify the perturbation on the interactome but cannot predict in which way the host will ultimately be affected by such perturbations. Therefore, some drugs that interact closely to the COVID-19 disease module, do not show therapeutic potential. Examples are Clozapine, that was linked to increased risk of COVID-19 infection[81,82], and Rifampicin, with associated warnings against using it together with Ritonavir, as well as caution when prescribing it in combination with Rifapentine for HIV/COVID-19 co-infection due to potential drug-drug interactions[83].

12 Note S12. Analysis of the overlap between both approaches

We analyzed the overlap between predictions by both approaches. Out of 126 drugs predicted by our matrix decomposition model, 80 were also considered by the network medicine approach.

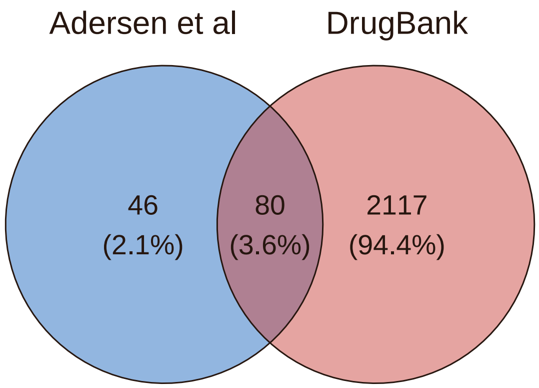

Figure S19. Venn diagram of drug sets used by the matrix decomposition and network medicine approaches. 80 were ranked by both approaches. Our matrix decomposition model uses data by Andersen et al.[14] (blue set on the left). Our network medicine approach uses all FDA-approved drugs in DrugBank that have known targets in the interactome (red set on the right).

Figure S20 shows where the 80 drugs in the intersection were ranked by the network medicine approach. The drugs in the overlap tend to be ranked in the top section of the ranking.

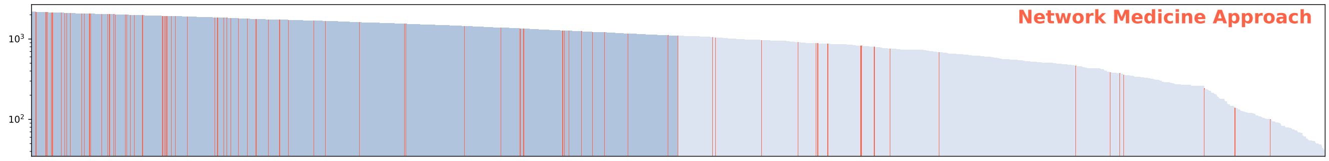

Figure S20. The 2197 drugs ranked by the network medicine approach, sorted by the output score of the method (higher to lower). Orange bars correspond to BSAs that are also ranked by the matrix decomposition approach. We observe that the vast majority of BSAs are ranked high by the network medicine approach. This suggests that BSAs are, perhaps unsurprisingly, good repurposing candidates for a viral disease such as COVID-19.

Among the top predictions by the matrix decomposition model which are also ranked high by the network medicine approach we find, for example, Ritonavir. It is the 4th best in the matrix decomposition model and is among the top 30 predictions by the network medicine approach. It acts not only as a HIV protease inhibitor, but also as a pharmacokinetic enhancer of other protease inhibitors, mainly due to its potent inhibition of the cytochrome P450 3A4 isoenzyme[84]. Thus, it can be considered both a direct acting antiviral drug, and a host targeted agent.

In addition, there are drugs predicted by both approaches that target only host factors. For example, Amiodarone (on the top 20 by the matrix decomposition model) is on the top 100 predictions by the network medicine approach. It was originally proposed as an antiarrhythmic, but also has proven efficacy against RNA viruses *in vitro* (including SARS-CoV-1)[85,86], and it went into clinical trials Phase 3 for treating Ebola (NCT02307591). Its antiviral activities are probably related to its interference with the endocytic pathway, and additional mechanisms[85].

Another interesting example is Tamoxifen, which was ranked on the top 30 predictions by both approaches. It is a selective estrogen receptor modulator used to treat or reduce the risk of breast cancer[87]. Later, Tamoxifen, and other estrogen receptor modulators have shown *in vitro* and *in vivo* activity against a wide range of human pathogens, including viruses, fungi, parasites, and bacteria[88]. The *in vitro* efficacy of Tamoxifen against HIV replication was attributed to inhibition of PKC and interaction with other targets in the NF-κB pathway[89]. Another study suggested that Tamoxifen inhibits HCV replication by interfering with the association of estrogen receptor alpha with RNA-dependent RNA polymerase NS5B, which affects the formation of the replication complex[90].

13 Note S13. Supplementary Datasets

Data	Location
Drug-Virus dataset	Andersen et al.[14], available for download at https://drugvirus.info/
Predictions from our matrix decomposition approach	Supplementary File 1
protein-protein interaction network (Gysi et al.)	Gysi et al.[38]
Drug target associations	Gysi et al.[38]
HuRI interactome (SwissProt only)	Supplementary File 2
Cheng et al. interactome (SwissProt only)	Supplementary File 3
Drug target associations (SwissProt only)	Supplementary File 4
Host Proteins (336 proteins - UniProt accession numbers)	Supplementary File 5
Host Proteins (336 proteins - Entrez IDs)	Supplementary File 6
DrugBank to ATC category map	Supplementary File 7
DrugBank entries with significant τ score in CMAP	Supplementary File 8
DrugBank entries with *in vitro* evidence	Supplementary File 9
DrugBank entries in Clinical Trials in December 1, 2020	Supplementary File 10
Predictions from our network medicine approach (on 2197 FDA approved drugs)	Supplementary File 11
COVID-19 signature for CMAP query (106 upregulated genes and 41 downregulated genes)	Table S2 in work by Ghandikota et al.[46]
Raw counts of RNAseq data expression data from 430 COVID-19 patients, and 54 controls[67]	https://www.ncbi.nlm.nih.gov/gds (GEO[91,92] accession number GSE152075[67])
Weights assigned to the host proteins by the kernel-based methods	Supplementary File 12

Table S7. Datasets and Supplementary Files:. This table summarises the datasets used throughout our experiments, as well as the results. When possible, we provide the data as a supplementary file available from Mendeley Data at http://dx.doi.org/10.17632/p7y5wmschg.1. References to the original sources are provided when redistribution rights are not granted.

References

1. Campillos, M., Kuhn, M., Gavin, A.-C., Jensen, L. J. & Bork, P. Drug target identification using side-effect similarity. *Science* **321**, 263–266 (2008).
2. Sirota, M. *et al.* Discovery and preclinical validation of drug indications using compendia of public gene expression data. *Sci Transl Med* **3**, 96ra77, DOI: 10.1126/scitranslmed.3001318 (2011).
3. Cami, A., Arnold, A., Manzi, S. & Reis, B. Predicting adverse drug events using pharmacological network models. *Sci. translational medicine* **3**, 114ra127–114ra127 (2011).
4. Lee, D. D. & Seung, H. S. Learning the parts of objects by non-negative matrix factorization. *Nature* **401**, 788–791 (1999).
5. Lee, D. D. & Seung, H. S. Algorithms for non-negative matrix factorization. In *Advances in neural information processing systems*, 556–562 (2001).
6. Dowden, H. & Munro, J. Trends in clinical success rates and therapeutic focus. *Nat. reviews. Drug discovery* **18**, 495 (2019).
7. Sosnina, E. A. *et al.* Recommender systems in antiviral drug discovery. *ACS omega* **5**, 15039–15051 (2020).
8. Bakal, G., Kilicoglu, H. & Kavuluru, R. Non-negative matrix factorization for drug repositioning: experiments with the repodb dataset. In *AMIA Annual Symposium Proceedings*, vol. 2019, 238 (American Medical Informatics Association, 2019).
9. Brown, A. S. & Patel, C. J. A standard database for drug repositioning. *Sci. data* **4**, 1–7 (2017).
10. Ceddia, G., Pinoli, P., Ceri, S. & Masseroli, M. Matrix factorization-based technique for drug repurposing predictions. *IEEE journal biomedical health informatics* **24**, 3162–3172 (2020).
11. Ding, C., Li, T., Peng, W. & Park, H. Orthogonal nonnegative matrix t-factorizations for clustering. In *Proceedings of the 12th ACM SIGKDD international conference on Knowledge discovery and data mining*, 126–135 (2006).

12. Li, T. & Ding, C. The relationships among various nonnegative matrix factorization methods for clustering. In *Sixth International Conference on Data Mining (ICDM'06)*, 362–371 (IEEE, 2006).

13. Tang, X. *et al.* Indicator regularized non-negative matrix factorization method-based drug repurposing for covid-19. *Front. Immunol.* **11**, 3824 (2021).

14. Andersen, P. I. *et al.* Discovery and development of safe-in-man broad-spectrum antiviral agents. *Int. J. Infect. Dis.* (2020).

15. Gayvert, K. M., Madhukar, N. S. & Elemento, O. A data-driven approach to predicting successes and failures of clinical trials. *Cell chemical biology* **23**, 1294–1301 (2016).

16. Cipolat, M. M. & Sprinz, E. Covid-19 pneumonia in an hiv-positive woman on antiretroviral therapy and undetectable viral load in porto alegre, brazil. *The Braz. J. Infect. Dis.* (2020).

17. Hall Jr, D. C. & Ji, H.-F. A search for medications to treat covid-19 via in silico molecular docking models of the sars-cov-2 spike glycoprotein and 3cl protease. *Travel. medicine infectious disease* 101646 (2020).

18. Gautret, P. *et al.* Hydroxychloroquine and azithromycin as a treatment of covid-19: results of an open-label non-randomized clinical trial. *Int. journal antimicrobial agents* 105949 (2020).

19. Kalil, A. C. Treating covid-19—off-label drug use, compassionate use, and randomized clinical trials during pandemics. *Jama* **323**, 1897–1898 (2020).

20. Gendrot, M. *et al.* Antimalarial artemisinin-based combination therapies (act) and covid-19 in africa: In vitro inhibition of sars-cov-2 replication by mefloquine-artesunate. *Int. J. Infect. Dis.* **99**, 437–440 (2020).

21. Zhavoronkov, A. Geroprotective and senoremediative strategies to reduce the comorbidity, infection rates, severity, and lethality in gerophilic and gerolavic infections. *Aging (Albany NY)* **12**, 6492 (2020).

22. Stebbing, J. *et al.* Covid-19: combining antiviral and anti-inflammatory treatments. *The Lancet Infect. Dis.* **20**, 400–402 (2020).

23. Laterre, P. F. *et al.* Association of interleukin 7 immunotherapy with lymphocyte counts among patients with severe coronavirus disease 2019 (covid-19). *JAMA network open* **3**, e2016485–e2016485 (2020).

24. Monneret, G. *et al.* Immune monitoring of interleukin-7 compassionate use in a critically ill covid-19 patient. *Cell. & molecular immunology* **17**, 1001–1003 (2020).

25. Who's solidarity clinical trial enters a new phase with three new candidate drugs. https://www.who.int/news/item/11-08-2021-who-s-solidarity-clinical-trial-enters-a-new-phase-with-three-new-candidate-drugs. Accessed: August 2021.

26. Hassanipour, S. *et al.* The efficacy and safety of favipiravir in treatment of covid-19: A systematic review and meta-analysis of clinical trials. *Sci. reports* **11**, 1–11 (2021).

27. Wang, M. *et al.* Remdesivir and chloroquine effectively inhibit the recently emerged novel coronavirus (2019-ncov) in vitro. *Cell research* **30**, 269–271 (2020).

28. Cai, Q. *et al.* Experimental treatment with favipiravir for covid-19: an open-label control study. *Engineering* (2020).

29. Du, Y.-X. & Chen, X.-P. Favipiravir: pharmacokinetics and concerns about clinical trials for 2019-ncov infection. *Clin. Pharmacol. & Ther.* (2020).

30. Wang, X. *et al.* The anti-influenza virus drug, arbidol is an efficient inhibitor of sars-cov-2 in vitro. *Cell Discov.* **6**, 1–5 (2020).

31. Omolo, C. A., Soni, N., Fasiku, V. O., Mackraj, I. & Govender, T. Update on therapeutic approaches and emerging therapies for sars-cov-2 virus. *Eur. J. Pharmacol.* **883**, 173348 (2020).

32. Cao, B. *et al.* A trial of lopinavir–ritonavir in adults hospitalized with severe covid-19. *New Engl. J. Medicine* (2020).

33. Consortium, W. S. T. Repurposed antiviral drugs for covid-19—interim who solidarity trial results. *New Engl. journal medicine* **384**, 497–511 (2021).

34. Grein, J. *et al.* Compassionate use of remdesivir for patients with severe covid-19. *New Engl. J. Medicine* **382**, 2327–2336 (2020).

35. Zhai, M. Z., Lye, C. T. & Kesselheim, A. S. Need for transparency and reliable evidence in emergency use authorizations for coronavirus disease 2019 (covid-19) therapies. *JAMA Intern. Medicine* (2020).

36. Réa-Neto, Á. *et al.* An open-label randomized controlled trial evaluating the efficacy of chloroquine/hydroxychloroquine in severe covid-19 patients. *Sci. reports* **11**, 1–10 (2021).

37. Galeano, D., Li, S., Gerstein, M. & Paccanaro, A. Predicting the frequencies of drug side effects. *Nat. Commun.* **11**, 1–14 (2020).

38. Gysi, D. M. *et al.* Network medicine framework for identifying drug-repurposing opportunities for COVID-19. *PNAS* **118**, DOI: 10.1073/pnas.2025581118 (2021).

39. Wishart, D. S. *et al.* DrugBank 5.0: a major update to the DrugBank database for 2018. *Nucleic Acids Res.* **46**, D1074–D1082, DOI: 10.1093/nar/gkx1037 (2018).

40. Cheng, F. *et al.* Network-based approach to prediction and population-based validation of in silico drug repurposing. *Nat. Commun.* **9**, 1–12, DOI: 10.1038/s41467-018-05116-5 (2018). Number: 1 Publisher: Nature Publishing Group.

41. Luck, K. *et al.* A reference map of the human binary protein interactome. *Nature* **580**, 402–408, DOI: 10.1038/s41586-020-2188-x (2020).

42. Consortium, T. U. UniProt: a worldwide hub of protein knowledge. *Nucleic Acids Res.* **47**, D506–D515, DOI: 10.1093/nar/gky1049 (2019).

43. Riva, L. *et al.* Discovery of SARS-CoV-2 antiviral drugs through large-scale compound repurposing. *Nature* 1–11, DOI: 10.1038/s41586-020-2577-1 (2020). Publisher: Nature Publishing Group.

44. Lamb, J. *et al.* The Connectivity Map: Using Gene-Expression Signatures to Connect Small Molecules, Genes, and Disease. *Science* (2006). Publisher: American Association for the Advancement of Science.

45. Subramanian, A. *et al.* A Next Generation Connectivity Map: L1000 Platform and the First 1,000,000 Profiles. *Cell* **171**, 1437–1452.e17, DOI: 10.1016/j.cell.2017.10.049 (2017).

46. Ghandikota, S., Sharma, M. & Jegga, A. G. Secondary analysis of transcriptomes of SARS-CoV-2 infection models to characterize COVID-19. *Patterns* **2**, 100247, DOI: https://doi.org/10.1016/j.patter.2021.100247 (2021).

47. Cao, M. *et al.* Going the Distance for Protein Function Prediction: A New Distance Metric for Protein Interaction Networks. *PLOS ONE* **8**, e76339, DOI: 10.1371/journal.pone.0076339 (2013). Publisher: Public Library of Science.

48. Guney, E., Menche, J., Vidal, M. & Barábasi, A.-L. Network-based in silico drug efficacy screening. *Nat. Commun.* **7**, 10331, DOI: 10.1038/ncomms10331 (2016).

49. Gordon, D. E. *et al.* A SARS-CoV-2 protein interaction map reveals targets for drug repurposing. *Nature* **583**, 459–468, DOI: 10.1038/s41586-020-2286-9 (2020).

50. Mitchell, A. L. *et al.* InterPro in 2019: improving coverage, classification and access to protein sequence annotations. *Nucleic Acids Res.* **47**, D351–D360, DOI: 10.1093/nar/gky1100 (2019).

51. Kokic, G. *et al.* Mechanism of SARS-CoV-2 polymerase stalling by remdesivir. *Nat. Commun.* **12**, 279, DOI: 10.1038/s41467-020-20542-0 (2021).

52. Naydenova, K. *et al.* Structure of the SARS-CoV-2 RNA-dependent RNA polymerase in the presence of favipiravir-RTP. *Proc. Natl. Acad. Sci.* **118**, DOI: 10.1073/pnas.2021946118 (2021). ISBN: 9782021946116 Publisher: National Academy of Sciences Section: Biological Sciences.

53. Uzunova, K., Filipova, E., Pavlova, V. & Vekov, T. Insights into antiviral mechanisms of remdesivir, lopinavir/ritonavir and chloroquine/hydroxychloroquine affecting the new SARS-CoV-2. *Biomed. & Pharmacother.* **131**, 110668, DOI: 10.1016/j.biopha.2020.110668 (2020).

54. Clososki, G. C. *et al.* Tenofovir Disoproxil Fumarate: New Chemical Developments and Encouraging in vitro Biological Results for SARS-CoV-2. *J. Braz. Chem. Soc.* 1552–1556 (2020).

55. Park, S.-J. *et al.* Antiviral Efficacies of FDA-Approved Drugs against SARS-CoV-2 Infection in Ferrets. *mBio* **11**, DOI: 10.1128/mBio.01114-20 (2020).

56. Parienti, J.-J. *et al.* Effect of Tenofovir Disoproxil Fumarate and Emtricitabine on nasopharyngeal SARS-CoV-2 viral load burden amongst outpatients with COVID-19: A pilot, randomized, open-label phase 2 trial. *EClinicalMedicine* **38**, 100993, DOI: 10.1016/j.eclinm.2021.100993 (2021).

57. García-Trejo, J. J., Ortega, R. & Zarco-Zavala, M. Putative Repurposing of Lamivudine, a Nucleoside/Nucleotide Analogue and Antiretroviral to Improve the Outcome of Cancer and COVID-19 Patients. *Front. Oncol.* **11**, 664794, DOI: 10.3389/fonc.2021.664794 (2021).

58. Zhou, Y. *et al.* In vitro efficacy of artemisinin-based treatments against SARS-CoV-2. *Sci. Reports* **11**, 14571, DOI: 10.1038/s41598-021-93361-y (2021).

59. Liu, S., Zheng, Q. & Wang, Z. Potential covalent drugs targeting the main protease of the SARS-CoV-2 coronavirus. *Bioinformatics* **36**, 3295–3298, DOI: 10.1093/bioinformatics/btaa224 (2020).

60. Hall, D. C. & Ji, H.-F. A search for medications to treat COVID-19 via in silico molecular docking models of the SARS-CoV-2 spike glycoprotein and 3CL protease. *Travel. Medicine Infect. Dis.* **35**, 101646, DOI: 10.1016/j.tmaid.2020.101646 (2020).

61. Yao, Y., Luo, Z. & Zhang, X. In silico evaluation of marine fish proteins as nutritional supplements for COVID-19 patients. *Food Funct.* **11**, 5565–5572, DOI: 10.1039/D0FO00530D (2020).

62. Martorana, A., Gentile, C. & Lauria, A. In Silico Insights into the SARS CoV-2 Main Protease Suggest NADH Endogenous Defences in the Control of the Pandemic Coronavirus Infection. *Viruses* **12**, 805, DOI: 10.3390/v12080805 (2020).

63. Mittra, I. *et al.* Resveratrol and Copper for treatment of severe COVID-19: an observational study (RESCU 002). *medRxiv* 2020.07.21.20151423, DOI: 10.1101/2020.07.21.20151423 (2020).

64. Barh, D. *et al.* Multi-omics-based identification of SARS-CoV-2 infection biology and candidate drugs against COVID-19. *Comput. Biol. Medicine* 104051, DOI: 10.1016/j.compbiomed.2020.104051 (2020).

65. Al-Motawa, M. S. *et al.* Vulnerabilities of the SARS-CoV-2 Virus to Proteotoxicity—Opportunity for Repurposed Chemotherapy of COVID-19 Infection. *Front. Pharmacol.* **11**, 1579, DOI: 10.3389/fphar.2020.585408 (2020).

66. Pathak, Y., Mishra, A., Choudhir, G., Kumar, A. & Tripathi, V. Rifampicin and Letermovir as potential repurposed drug candidate for COVID-19 treatment: insights from an in-silico study. *Pharmacol. reports: PR* **73**, 926–938, DOI: 10.1007/s43440-021-00228-0 (2021).

67. Lieberman, N. A. P. *et al.* In vivo antiviral host transcriptional response to SARS-CoV-2 by viral load, sex, and age. *PLOS Biol.* **18**, 1–17, DOI: 10.1371/journal.pbio.3000849 (2020). Publisher: Public Library of Science.

68. Kost-Alimova, M. *et al.* A High-Content Screen for Mucin-1-Reducing Compounds Identifies Fostamatinib as a Candidate for Rapid Repurposing for Acute Lung Injury. *Cell Reports Medicine* **1**, 100137, DOI: 10.1016/j.xcrm.2020.100137 (2020).

69. Hoepel, W. *et al.* High titers and low fucosylation of early human anti–SARS-CoV-2 IgG promote inflammation by alveolar macrophages. *Sci. Transl. Medicine* (2021). Publisher: American Association for the Advancement of Science.

70. Zhou, P. *et al.* A pneumonia outbreak associated with a new coronavirus of probable bat origin. *Nature* **579**, 270–273, DOI: 10.1038/s41586-020-2012-7 (2020).

71. Hoffmann, M. *et al.* SARS-CoV-2 Cell Entry Depends on ACE2 and TMPRSS2 and Is Blocked by a Clinically Proven Protease Inhibitor. *Cell* **181**, 271–280.e8, DOI: 10.1016/j.cell.2020.02.052 (2020). Publisher: Elsevier.

72. Wang, B. *et al.* In search of preventive strategies: novel high-cbd cannabis sativa extracts modulate ace2 expression in covid-19 gateway tissues. *Aging* **12**, 22425–22444, DOI: 10.18632/aging.202225 (2020).

73. Suba, Z. Prevention and therapy of COVID-19 via exogenous estrogen treatment for both male and female patients: Prevention and therapy of COVID-19. *J. Pharm. & Pharm. Sci.* **23**, 75–85, DOI: 10.18433/jpps31069 (2020).

74. Ragia, G. & Manolopoulos, V. G. Inhibition of SARS-CoV-2 entry through the ACE2/TMPRSS2 pathway: a promising approach for uncovering early COVID-19 drug therapies. *Eur. J. Clin. Pharmacol.* **76**, 1623–1630, DOI: 10.1007/s00228-020-02963-4 (2020).

75. Mauvais-Jarvis, F., Klein, S. L. & Levin, E. R. Estradiol, Progesterone, Immunomodulation, and COVID-19 Outcomes. *Endocrinology* **161**, DOI: 10.1210/endocr/bqaa127 (2020).

76. Wessels, I., Rolles, B. & Rink, L. The Potential Impact of Zinc Supplementation on COVID-19 Pathogenesis. *Front. Immunol.* **11**, 1712, DOI: 10.3389/fimmu.2020.01712 (2020).

77. Finzi, E. & Harrington, A. Zinc treatment of outpatient COVID-19: A retrospective review of 28 consecutive patients. *J. Med. Virol.* **93**, 2588–2590, DOI: 10.1002/jmv.26812 (2021). _eprint: https://onlinelibrary.wiley.com/doi/pdf/10.1002/jmv.26812.

78. Finzi, E. Treatment of SARS-CoV-2 with high dose oral zinc salts: A report on four patients. *Int. J. Infect. Dis.* **99**, 307–309, DOI: 10.1016/j.ijid.2020.06.006 (2020).

79. Rehman, A. u. & Qureshi, S. A. The role of primary and secondary bio-molecules in optical diagnosis of pandemic COVID-19 outbreak. *Photodiagnosis Photodyn. Ther.* **31**, 101953, DOI: 10.1016/j.pdpdt.2020.101953 (2020).

80. Radulesco, T., Lechien, J. R., Saussez, S., Hopkins, C. & Michel, J. Safety and Impact of Nasal Lavages During Viral Infections Such as SARS-CoV-2. *Ear, Nose & Throat J.* **100**, 188S–191S, DOI: 10.1177/0145561320950491 (2021). Publisher: SAGE Publications Inc.

81. Govind, R., Freitas, D. F. d., Pritchard, M., Hayes, R. D. & MacCabe, J. H. Clozapine treatment and risk of COVID-19 infection: retrospective cohort study. *The Br. J. Psychiatry* **219**, 368–374, DOI: 10.1192/bjp.2020.151 (2021). Publisher: Cambridge University Press.

82. Consensus statement on the use of clozapine during the COVID-19 pandemic – Journal of Psychiatry & Neuroscience.

83. Tamuzi, J. L. *et al.* Implications of COVID-19 in high burden countries for HIV/TB: A systematic review of evidence. *BMC Infect. Dis.* **20**, 744, DOI: 10.1186/s12879-020-05450-4 (2020).

84. Hull, M. W. & Montaner, J. S. G. Ritonavir-boosted protease inhibitors in HIV therapy. *Annals Medicine* **43**, 375–388, DOI: 10.3109/07853890.2011.572905 (2011). Publisher: Taylor & Francis _eprint: https://doi.org/10.3109/07853890.2011.572905.

85. Stadler, K. *et al.* Amiodarone alters late endosomes and inhibits SARS coronavirus infection at a post-endosomal level. *Am J Respir Cell Mol Biol* **39**, 142–149, DOI: 10.1165/rcmb.2007-0217OC (2008).

86. Castaldo, N. *et al.* Safety and Efficacy of Amiodarone in a Patient With COVID-19. *JACC Case Rep* **2**, 1307–1310, DOI: 10.1016/j.jaccas.2020.04.053 (2020).

87. Jordan, V. C. Fourteenth Gaddum Memorial Lecture. A current view of tamoxifen for the treatment and prevention of breast cancer. *Br J Pharmacol* **110**, 507–517 (1993).

88. Montoya, M. C. & Krysan, D. J. Repurposing Estrogen Receptor Antagonists for the Treatment of Infectious Disease. *mBio* **9**, e02272–18, DOI: 10.1128/mBio.02272-18 (2018).

89. Laurence, J., Cooke, H. & Sikder, S. K. Effect of tamoxifen on regulation of viral replication and human immunodeficiency virus (HIV) long terminal repeat-directed transcription in cells chronically infected with HIV-1. *Blood* **75**, 696–703 (1990).

90. Watashi, K. *et al.* Anti-hepatitis C virus activity of tamoxifen reveals the functional association of estrogen receptor with viral RNA polymerase NS5B. *J Biol Chem* **282**, 32765–32772, DOI: 10.1074/jbc.M704418200 (2007).

91. Edgar, R., Domrachev, M. & Lash, A. E. Gene Expression Omnibus: NCBI gene expression and hybridization array data repository. *Nucleic Acids Res* **30**, 207–210, DOI: 10.1093/nar/30.1.207 (2002).

92. Barrett, T. *et al.* NCBI GEO: archive for functional genomics data sets–update. *Nucleic Acids Res* **41**, D991–995, DOI: 10.1093/nar/gks1193 (2013).
