# Supplementary material for: Machine learning and network medicine approaches for drug repositioning for COVID-19
Source: Patterns (N Y). 2021 Nov 9;3(1):100396. doi: 10.1016/j.patter.2021.100396 (PMC8576113; doi:10.1016/j.patter.2021.100396)
Supplement: Document S2. Article plus supplemental information [file mmc2.pdf]

Patterns

Article

Machine learning and network medicine approaches for drug repositioning for COVID-19

Graphical abstract

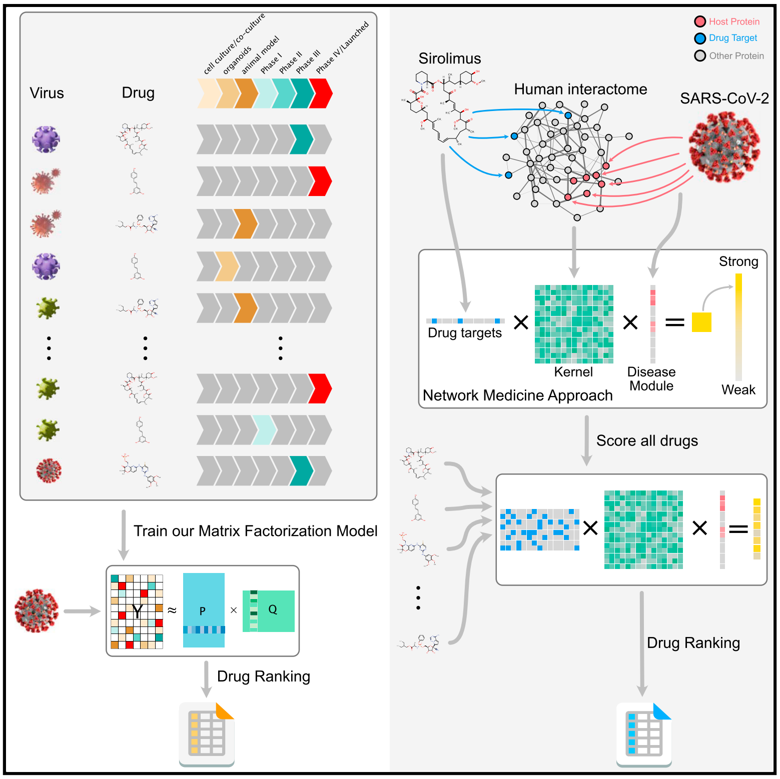

Highlights

- A matrix decomposition model for repurposing broad-spectrum antivirals
- A graph kernel approach to model perturbations induced by drugs on the interactome
- Graph kernels can integrate transcriptomics data to improve drug repurposing
- CoREx: a free online tool to formulate hypothesis for drug repurposing for COVID-19

Authors

Suzana de Siqueira Santos, Mateo Torres, Diego Galeano, María del Mar Sánchez, Luca Cernuzzi, Alberto Paccanaro

Correspondence

alberto.paccanaro@rhul.ac.uk

In brief

We present two complementary machine learning approaches for drug repositioning against COVID-19 that target SARS-CoV-2 and its cellular processes in the host, respectively. Our matrix decomposition approach exploits drug developmental information to predict broad-spectrum antivirals; our graph kernel-based approach, rooted in ideas from network medicine, predicts which FDA-approved drugs are more likely to perturb the human subnetwork that is crucial for SARS-CoV-2 infection/ replication. We also introduce CoREx, a freely available online tool to reason and formulate hypothesis about drug repurposing in the context of biological networks and pharmacological information.

Santos et al., 2022, Patterns *3*, 100396
January 14, 2022 © 2021 The Authors.
https://doi.org/10.1016/j.patter.2021.100396

CellPress

Patterns

CellPress
OPEN ACCESS

Article

Machine learning and network medicine approaches for drug repositioning for COVID-19

Suzana de Siqueira Santos,[1,5,6] Mateo Torres,[1,5,6] Diego Galeano,[1,3,5,6] María del Mar Sánchez,[4] Luca Cernuzzi,[4] and Alberto Paccanaro[1,2,5,6,7,*]
[1]Escola de Matemática Aplicada, Fundação Getulio Vargas, Rio de Janeiro 22250-900, Brazil
[2]Department of Computer Science, Centre for Systems and Synthetic Biology, Royal Holloway, University of London, Egham Hill, Egham TW20 0EX, UK
[3]Facultad de Ingenieria, Universidad Nacional de Asunción, Luque 110948, Paraguay
[4]Universidad Católica "Nuestra Señora de la Asunción", Asunción C.C. 1683, Paraguay
[5]COVID-19 International Research Team
[6]These authors contributed equally
[7]Lead contact
*Correspondence: alberto.paccanaro@rhul.ac.uk
https://doi.org/10.1016/j.patter.2021.100396

THE BIGGER PICTURE The development timeline for treatments against emergent viral diseases can be significantly reduced by re-using drugs already available on the market—a concept known as drug repositioning. We present two complementary machine learning approaches for drug repositioning that target SARS- CoV-2 and host factors, respectively. Our matrix decomposition approach exploits drug developmental information to predict the effectiveness of broad-spectrum antiviral drugs. Our graph kernel-based approach, rooted in ideas from network medicine, predicts which FDA-approved drugs are more likely to perturb the human subnetwork that is crucial for SARS-CoV-2 infection/replication. We also introduce CoREx, a freely available online tool that enables scientists to reason and formulate hypotheses about drug repurposing in the context of biological networks and pharmacological information.
While we have developed these methodologies for COVID-19, our approaches can be applied to any viral disease.

1 2 3 4 5 **Development/Pre-production:** Data science output has been rolled out/validated across multiple domains/problems

SUMMARY

We present two machine learning approaches for drug repurposing. While we have developed them for COVID-19, they are disease-agnostic. The two methodologies are complementary, targeting SARS-CoV-2 and host factors, respectively. Our first approach consists of a matrix factorization algorithm to rank broad-spectrum antivirals. Our second approach, based on network medicine, uses graph kernels to rank drugs according to the perturbation they induce on a subnetwork of the human interactome that is crucial for SARS-CoV-2 infection/replication. Our experiments show that our top predicted broad-spectrum antivirals include drugs indicated for compassionate use in COVID-19 patients; and that the ranking obtained by our kernel-based approach aligns with experimental data. Finally, we present the COVID-19 repositioning explorer (CoREx), an interactive online tool to explore the interplay between drugs and SARS-CoV-2 host proteins in the context of biological networks, protein function, drug clinical use, and Connectivity Map. CoREx is freely available at: https://paccanarolab.org/corex/.

INTRODUCTION

Drug discovery and development present several challenges, including high attrition rates, long development times, and substantial costs.[1] Drug repositioning involves the use of de-risked compounds in humans, which translates into lower costs and shorter development times.[2] Computational methods can assist drug repurposing research projects by providing rankings of

This is an open access article under the CC BY license (http://creativecommons.org/licenses/by/4.0/).

drugs based on predicted therapeutic efficacy, as well as tools to help scientists reason about drug effectiveness by integrating diverse available biomedical knowledge.

Coronaviruses are notoriously difficult to manage, as there is no specific antiviral treatment that has been proven effective against the infections they induce.[3] Identifying commercially available drugs with therapeutic effects for COVID-19 could provide early treatment options until effective therapies become widely available. A growing corpus of literature identifies several categories of treatment that revolves around the use of drugs with a mode of action that targets the molecular structure of the virus (*virally targeted agents*), or its cellular processes in the host (*host-targeted agents*), or those based on combinatorial therapies.[4–7]

In this paper, we present two different machine learning approaches, and a webtool, for drug repurposing for COVID-19. Our first machine learning approach focuses on virally targeted agents and aims at ranking broad-spectrum antiviral (BSA) drugs. Given a small number of drugs associated with a virus, and their stage in the drug development process, our matrix decomposition algorithm assigns scores to a larger group of drugs with previously unknown associations with the virus. Our method predicts BSAs against SARS-CoV-2 by exploiting information about stages of drug development that are interpreted as probabilities of drug approval. To our knowledge, our matrix decomposition model is the first that integrates developmental-stage information to predict the efficacy of drugs against viral diseases, and we show that this is crucial to obtain better predictions.

Our second machine learning approach focuses on host-targeted agents, and prioritizes FDA-approved drugs based on ideas from network medicine.[8] In particular, it exploits the concept of a disease module, which has been instrumental in the prediction of disease genes for hereditary diseases.[9–12] For a virus, a disease module can be defined as the set of human proteins (hereafter, host proteins) that interact with viral proteins, allowing the infection and replication processes. Recently, Gysi et al.[13] have shown that, for SARS-CoV-2, most of the experimentally identified human host proteins[14] form a distinct COVID-19 disease module in the interactome. Our network medicine-based approach is based on the idea that the binding of drugs to their protein targets causes a perturbation that propagates through the interactome. By quantifying this perturbation, it is possible to calculate the extent of the effect that a drug induces on the COVID-19 disease module. Our method ranks FDA-approved drugs based on this effect, which is estimated using graph kernels. An important aspect of our method is that it offers a natural way to model the relative importance of host proteins for the disease, and we show that our network medicine approach benefits from this prioritization of host proteins.

Finally, we present the COVID-19 repositioning explorer (CoREx), an online tool that enables scientists to analyze and reason about drug repurposing in a functional context on the interactome and thus allows the exploration of our results as well as the formulation of novel repurposing hypotheses. CoREx integrates several sources of information, connecting functional protein modules with drug targets and host proteins. CoREx also provides additional evidence for a drug of interest, such as whether the drug is on clinical trials for COVID-19, or whether the drug could reverse the gene expression signature of SARS-CoV-2 infection based on the Connectivity Map (CMAP).[15,16]

RESULTS

A matrix decomposition model for antiviral discovery

Recently, Andersen et al.[17] published a dataset containing 850 associations between 126 BSA drugs and 80 viruses for which they have been approved or are under development. Importantly, each drug-virus association was manually curated and is annotated with its stage in the drug development process.

Figure 1 shows the number of drug-virus associations that corresponds to each developmental stage, as well as histograms of the associations grouped per drug and per virus. We notice that the associations are not uniformly distributed for viruses or drugs (Figure 1, left and right panels). This type of long-tailed distribution of entries has been previously observed in datasets that appear in the recommender system literature, such as Netflix or Movielens,[18] and we have recently exploited this property to build a recommender system based on matrix factorization for predicting drug side effect frequencies.[19]

Various types of recommender systems have recently been developed for different settings of the drug repositioning problem. A few methods are based on variations of the non-negative matrix factorization (NMF) algorithm,[20,21] such as the NMF with L2 regularization by Bakal et al.,[22] the TriFactor NMF by Ceddia et al.,[23] and the indicator-regularized non-negative matrix factorization (IRNMF) method by Tang et al.,[24] which was developed to repurpose drugs for COVID-19. Our aim is also to build a recommender system that recommends BSA drugs to viruses and the novelty of our approach lies in the realization that the stages of drug development for drug-virus associations can be related to the probability of reaching the final stage of drug development (hereafter, probability of success). This observation is motivated by the empirical evidence (e.g., Dowden and Munro[25]) that the probability of success of a candidate drug increases as the candidate drug moves to the next developmental stage in the drug development process. This led us to develop a novel objective function that models the probabilities of success of drug-virus associations using their stage in the drug development process. In this paper, we show how the integration of this type of information greatly improves prediction performance.

In recommender systems based on matrix decomposition, the fundamental assumption is that users and movies can be represented as latent feature vectors in a low-dimensional space, and that a rating value for a specific user-movie pair is obtained by the dot product of the corresponding feature vectors. In our context, each drug and each virus can be represented as low-dimensional feature vectors in a latent space such that the dot product between the vectors model effective drug-virus associations. Having collected all the associations in a binary matrix Y, where each entry $y_{ij} = 1$ if and only if drug i is associated to virus j in the Andersen et al.[17] dataset ($y_{ij} = 0$ otherwise), for each drug i we learn a low-dimensional feature vector $p_i \in \mathbb{R}^k$ (the *drug signature*) and for each virus j a low-dimensional feature vector $q_j \in \mathbb{R}^k$ (the *virus signature*), such that $y_{ij} \approx p_i^T q_j$. Therefore, our algorithm amounts to decomposing the $n \times m$ matrix Y into the product of two matrices $P \in \mathbb{R}^{n \times k}$ in which each row is a drug signature p_i^T,

CellPress
OPEN ACCESS

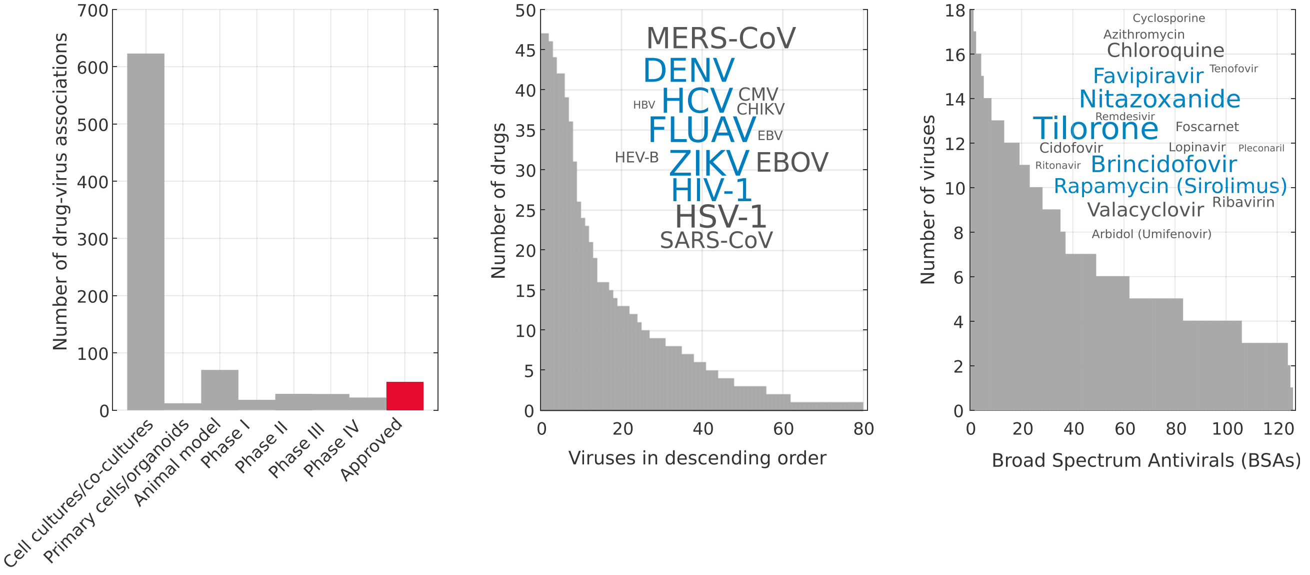

Figure 1. Drug-virus dataset statistics

We used the dataset manually curated by Andersen et al.[17] (Left) Number of drug-virus associations grouped by their known developmental status. The development of broad-spectrum antivirals (BSA) starts with *in vitro* experiments (e.g., cell culture), moves to animal models, and then to clinical trials in humans (phases I–IV). It terminates with the approval of the drug for commercial use (in red). (Middle) Number of drugs (BSAs) associated to each virus in the dataset. Inset: the word cloud shows the 14 viruses with most associations. The size of the word is proportional to its number of associations and the five most popular viruses among drugs are colored blue. (Right) Number of viruses associated to each drug in the dataset. Inset: the word cloud shows the 18 drugs with most associations and the five most popular drugs among viruses are colored blue.

and $Q \in \mathbb{R}^{k \times m}$ in which each column is a virus signature q_j, and $k \ll \min(n, m)$. Indicating their product with $\widehat{Y}$, we have $Y \approx PQ = \widehat{Y}$. Matrices P and Q are learned by minimizing the following cost function:

$$\begin{cases}\min_{P,Q}\mathcal{L}(P,Q)=\underbrace{\frac{1}{2}\left\|M^A\circ(Y-PQ)\right\|_F^2}_{\text{approved, phase IV}}+\underbrace{\frac{1}{2}\sum_{s\in\{B,C,D,E\}}\alpha_s\|M^s\circ(Y-PQ)\|_F^2}_{\text{In vitro, animal model, clinical trials}}+\underbrace{\frac{\alpha_z}{2}\|M^z\circ(PQ)\|_F^2}_{\text{zero}-\text{driven regularisation}}\end{cases}$$

$$\text{subject to non} - \text{negative constraints } P, Q \geq 0, \qquad \text{(Equation 1)}$$

where $\|.\|_F$ is the Frobenius norm of a matrix, $\circ$ is the element-wise (Hadamard) product, and the letters A, B, C, D, E indicate disjoint subsets of entries in Y that are defined according to the known developmental stages of drug-virus associations, as explained below. Let us now analyze Equation 1 to understand how the information about drug developmental stages is integrated into our system to model probabilities of success of drug-virus associations.

During learning, the drug-virus associations are divided into groups according to their stage of development. The first term in Equation 1 is the fitting constraint on the approved and phase IV drug-virus associations (set A). Matrix M^A is used to apply the summation only to entries in Y belonging to the set of approved associations A, being defined as: $M^A_{ij} = 1$ if drug i was approved or is in phase IV for virus j, or 0 otherwise. Thus, the first term of Equation 1 is attempting to find a decomposition PQ to reconstruct the associations in set A exactly. The second term in Equation 1 has an equivalent role for the remaining known associations in Y, corresponding to earlier stages in the drug development process—sets B, C, and D contain entries in clinical trials phases I, II, and III, respectively, while set E contains associations in *in vitro* and animal model stages. Here the corresponding M^s matrices are used to apply the summations only to entries belonging to the corresponding sets ($M^s_{ij} = 1$ if the entry $y_{i,j}$ belongs to set s). However, for these sets, their contributions to the loss are weighted differently using the parameters $\alpha_s \in [0, 1]$. These parameters have the key role of downweighting these terms in the minimization, in a way that reflects their higher uncertainty of success due to their earlier stage of drug development, thus effectively coding probabilities of success for each subset. Similarly, the third term in Equation 1 is used to downweight the importance of the zero entries of Y while also serving

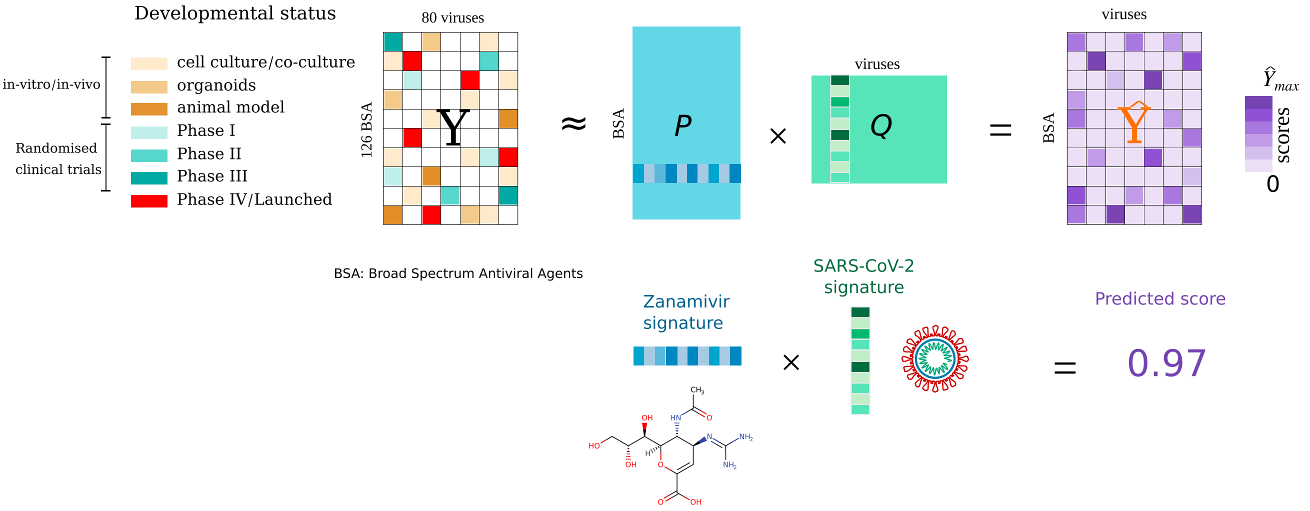

Figure 2. Overview of our matrix decomposition model for predicting effective drug-virus associations

Totals of 850 associations for $n = 126$ different BSAs and $m = 80$ distinct viruses were collected from the Andersen et al.[17] database. The observed associations were arranged into an $n \times m$ matrix Y by setting $y_{ij} = 1$. Unobserved associations were encoded with zeros. Our algorithm decomposes the matrix Y into the product of two matrices, P (of size $n \times k$) and Q (of size $k \times m$). By multiplying the matrices P and Q, we obtain $\hat{Y}$, which models Y, where all the entries are replaced with real numbers—these correspond to our predicted scores. Rows of P are the BSA feature vectors (or BSA signature); columns of Q are the virus feature vectors (virus signature). The lower illustration depicts how our model discovers a low-dimensional signature vector for the antiviral drug zanamivir, and a low-dimensional signature vector for SARS-CoV-2. The dot product of these two signatures is the predicted efficacy of zanamivir against SARS-CoV-2.

as a regularization term.[19] Finally, we impose non-negative constraints on P and Q to favor the interpretability of the learned representations.[19,20]

Thus, our model is closely related to NMF.[20] Both models seek to decompose a data matrix Y into the product of two non-negative matrices P and Q. However, the NMF model considers all the entries in Y equally during the learning—this works well when entries have the same meaning, e.g., pixels in an image.[20] Instead, in our approach, we assign different levels of importance to subsets of entries to reflect the drug stages of development, thus coding the probability of drug success, which is what we are trying to predict. This gives rise to a loss function in Equation 1 that is different from NMF. Finally, notice that, in our model, if we set the values of all the α parameters to 1—which amounts to discarding the role of probabilities of success—we obtain the original NMF model.

An overview of our matrix decomposition model is illustrated in Figure 2. Our starting point is the matrix Y containing binary drug-virus associations. We learn the matrices P and Q, which minimize the loss function in Equation 1, by employing an iterative algorithm that uses a simple multiplicative update rule (see the Experimental procedures). Our algorithm, inspired by the diagonally rescaled principle of NMF,[20] is fast, it does not require setting a learning rate or applying a projection function and it satisfies the Karush-Kuhn-Tucker (KKT) complementary conditions of convergence (see the Experimental procedures). Having learned P and Q such that $Y \approx PQ$, we calculate the matrix $\hat{Y} = PQ$. Note that, while Y contains binary entries, $\hat{Y}$ contains real positive numbers that are our predicted scores.

Predicting effective BSA drugs against viruses

To perform an *in silico* evaluation of the performance of our model, we formulated a matrix completion task under a leave-one-out cross-validation (LOOCV) procedure using the 49 BSA drugs that have been approved for use, and the 22 that reached phase IV of clinical trials for 28 viruses. To prevent overfitting and biases during hyperparameter tuning, we performed a different LOOCV by using clinical trials associations from phases I, II, and III to set the model parameters. Our final model parameters were: $k = 5$, $\alpha_B = 0.16$, $\alpha_C = 0.27$, $\alpha_D = 0.71$, $\alpha_E = 0.01$, and $\alpha_z = 2$ (see the Experimental procedures).

We compared the performance of our algorithm with the other drug-repurposing approaches that we mentioned earlier, namely the NMF with L2 regularization,[22] the TriFactor NMF,[23,26] and the IRNMF,[24] which was also developed for COVID-19. Moreover, we also included standard NMF and truncated singular value decomposition (tSVD)[18] as baselines. The relation between previous NMF-based drug repositioning methods and our model is explained in Note S1.

Following other works that used LOOCV evaluations,[9,27,28] we evaluated the performance at predicting one drug at a time, measuring how often that drug was found within the first 1, 5, 10, 15, 20, 25, and 30 drugs predicted by the different algorithms. Here, it is important to remind ourselves that our model takes as input an incomplete sparse drug-virus matrix, with only 8.43% non-zero entries, and outputs predicted scores for all the entries in the matrix. In the evaluation presented here, we focus on validating predictions corresponding to the interesting case where drug-virus associations are not yet under development (see Note S2 for the case of predicting drugs already under development, but not approved, for specific viruses). Therefore, in our LOOCV procedure, one drug-virus association (approved or phase IV) was removed at a time from the drug-virus matrix Y (by setting the corresponding entry to zero). We then trained the model, and scores were predicted for all drugs. Finally, we ranked drugs that had no known association with that virus and checked the percentage of cases in

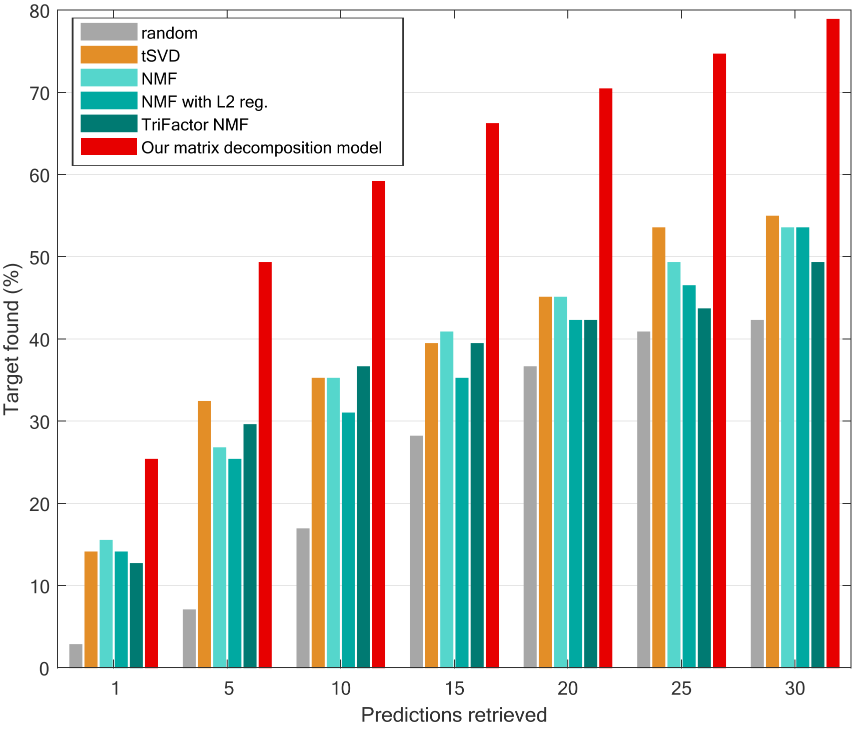

Figure 3. Performance at predicting approved/phase IV BSAs for 28 viruses

Percentage of approved or phase IV BSA drugs found for a specific virus in the top K predictions retrieved. The performance of our method is compared with different matrix decomposition algorithms in a leave-one-out fashion. NMF, non-negative matrix factorization; tSVD, truncated singular value decomposition. A baseline based on random scores sampled from a uniform distribution is also included.

which the correct (effective) drug for the virus was found among the top K predictions.

Figure 3 shows the performance of the methods at predicting effective (approved/phase IV) BSA drugs against specific viruses. Our model outperforms the competitors for each number of predictions retrieved: by 9.8%–22.5% in the top 1, by 16.9%–42.2% in the top 5, by 22.5%–42.2% in the top 10, and by 25.3%–38% in the top 20. Overall, our method could recover 70% of the phase IV/approved BSA drugs for 28 distinct viruses in the top 20 predictions. We also observed that, in some cases, tSVD and TriFactor NMF perform slightly better than NMF. The comparison of our method's performance with IRNMF was performed in a smaller subset of the matrix Y (see Figure S1 in Note S1).

The good prediction performance of our model prompted us to ask how much of the prediction power could be attributed to the integration of developmental stages information in our cost function (see Equation 1). Our model significantly improves performance over two control baselines that: (1) randomize developmental stage information in the training set and (2) remove developmental stage information from our cost function (see a detailed discussion in Note S3).

The analysis of our predictions for SARS-CoV-2 is presented in detail in the "Evaluation" section, together with the results of our network medicine approach.

Repositioning FDA-approved drugs with network medicine

The majority of BSAs considered previously target viral proteins. In our work, we also explored approaches that consider drugs targeting human proteins. Human proteins interact with each other, forming a protein-protein interaction (PPI) network. This and other biological networks have been explored in relation to disease—this area of research has often been called network medicine. It has been shown that proteins associated with specific hereditary diseases tend to cluster in neighborhoods of the interactome (the disease module),[8,29,30] and successful applications of molecular network analysis have been reported for the identification of disease genes,[9] drug development,[10] and drug efficacy prediction.[29]

The use of network medicine for assisting drug repositioning was originally applied to genetic diseases.[29] A drug induces its effects on a human PPI subnetwork by binding to its target proteins,[31,32] and this causes a perturbation in the interactome that is then propagated. Thus, drug efficacy for a genetic disease can be associated to how likely the drug is to affect its disease module through the perturbations propagated in the human PPI network.[29] To implement this idea, Guney et al.[29] proposed a distance (hereafter, the Guney distance) based on the shortest path length between the disease module and the drug targets.

Recent studies suggest that an analogous approach can be useful for infectious diseases such as COVID-19.[13,33] Viruses hijack host proteins to facilitate their replication, and hence the inhibition or knockdown of such host proteins can block viral replication.[34] Gysi et al.[13] have shown that, for SARS-CoV-2, most of the experimentally identified host proteins[14] group together in a large connected component, forming a COVID-19 disease module, as illustrated in Figure 4A with red nodes (host protein subnetwork). Therefore, the idea here is to find drugs that, by binding to their targets (blue nodes in Figure 4A), are likely to perturb this module.

We can think of the perturbation caused by a drug as a process in which the effect of the drug *diffuses* on the PPI network starting from its targets. Thus, our drug repurposing problem translates into the problem of the diffusion between drug targets and the set of host proteins. Gysi et al.[13] implemented this idea for COVID-19 by using the diffusion state distance (DSD).[35]

Kernels on graphs are appealing for modeling a diffusion process on a network. They are theoretically well founded in statistical learning theory,[36,37] and have shown good empirical results in many applications.[35,38,39] Graph kernels can be interpreted as measures of similarity between nodes in a network. There are different types of kernels. The p-step random walk kernel, for example, is directly associated to the number of times a random

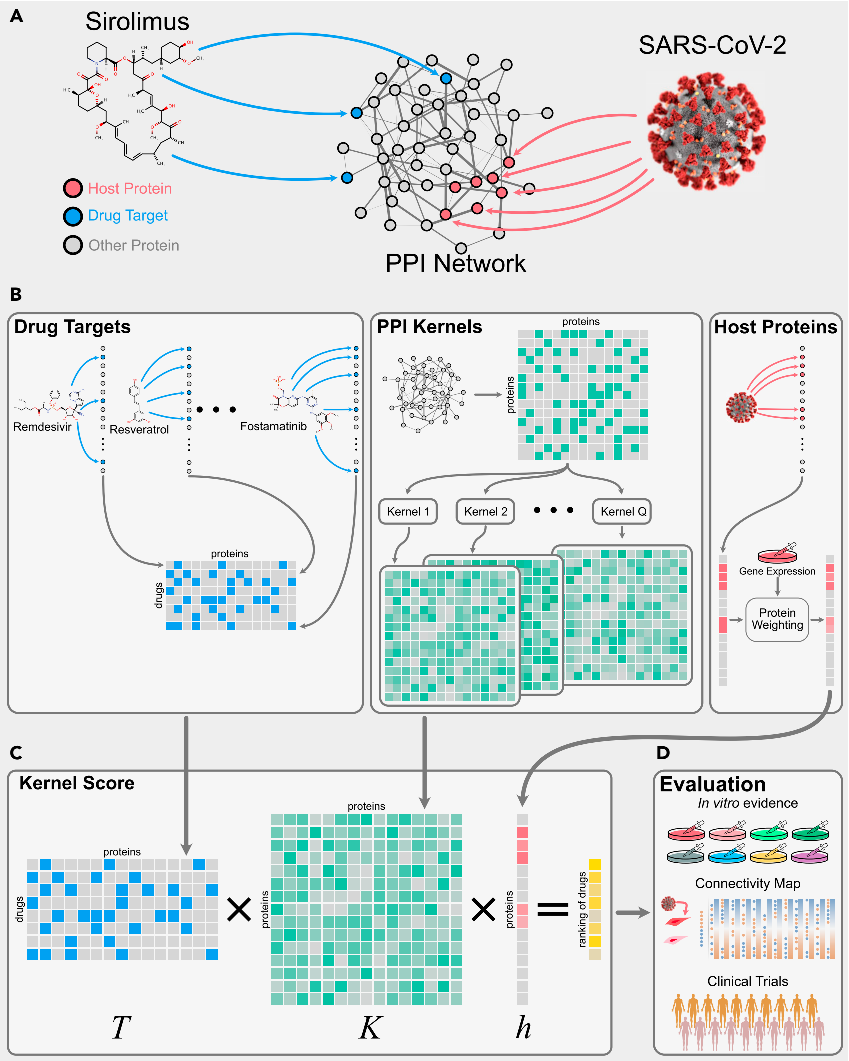

Figure 4. Overview of our network medicine approach

(A) The human interactome containing both host proteins (red) and drug targets (blue).

(B) The totals of 14,941 drug target associations between N = 2,197 FDA-approved drugs and n_V = 18,505 proteins are represented by a binary matrix T (blue matrix). Multiple graph kernels are calculated on the interactome, resulting in $n_V \times n_V$ matrices (green matrices). The host proteins are represented by a vector h of size n_V (red vector) indicating their weights (based on gene expression data).

(C) Our kernel score is calculated using a matrix multiplication to obtain a prediction score for each drug.

(D) The obtained ranking is evaluated using different types of evidence: *in vitro* efficacy against SARS-CoV-2, Connectivity Map, and clinical trials.

walker starting from a node i visits a node j after p steps.[36] Another example is the diffusion kernel (or heat kernel), which can be thought of as a random walk with an infinite number of infinitesimally small steps. An alternative interpretation is that this kernel corresponds to the amount of heat that reaches a node j after diffusing an initial heat from node i.[36]

Importantly, kernels on graphs can be applied in a natural way to nodes with weights. This property can be particularly useful for our problem: we can assign weights to the host proteins to model the different roles that they have for the infection/replication of the virus. For example, it has been shown that the ACE2 protein receptor is the viral entry factor of SARS-CoV-2.[40] Another study based on gene expression experiments on infected SARS-CoV-2 cell lines suggests that certain protein-coding genes play a key role during the infection process.[41] Then, the amount of change in gene expression after SARS-CoV-2 infection may be associated with the level of importance of the protein for the infection. In addition, perturbing host proteins whose expression levels change the most may be important for reverting the effect that the infection causes in gene expression. Predicting drugs that might revert this effect has been shown to be a good strategy for assisting in the discovery of effective small molecules for different diseases.[15,42]

To assist the repositioning of drugs for COVID-19, we used five different kernels on graphs and weighted the host proteins with differential gene expression data (absolute value of the log fold change between the gene expression levels of COVID-19 patients, and controls—see experimental procedures for details on the RNA-seq data). We used the interactome assembled by Gysi et al.,[13] and a set of 336 human proteins that were identified as hosts of SARS-CoV-2 (see experimental procedures). Every FDA-approved drug with known targets in this interactome was ranked by each of the kernels in our approach (see experimental procedures). The selected kernels are defined in terms of the graph Laplacian (see experimental procedures), as shown in Table 1. For each drug, we obtain the graph kernel-based similarities between each of its targets and each of the host proteins. The final score of a drug is the sum of these similarities weighted by the amount of change in the host protein expression levels after infection. Drug scores are then ordered, obtaining a drug ranking which is evaluated. We also calculated an aggregated ranking, which we called *avgRank*, where the ordinal position of each drug was obtained by simply averaging the ranking position that the drug had obtained in each of the kernels.

The mathematical formulation of this approach turns out to be quite simple. Let n_V the number of proteins in the PPI network, N the number of FDA-approved drugs, and T an $N \times n_V$ matrix of drug target associations, where $T_{ij} = 1$ if j is a target of drug i, and 0 otherwise (see drug targets box in Figure 4B). Let K be a square matrix of dimensions $n_V \times n_V$ representing kernel-based similarities between proteins on the PPI network (see PPI kernels box in Figure 4B). Let h be an n_V-dimensional column vector containing weights related to the differential expression data of the host proteins and zeros for the remaining proteins (see

Table 1. Graph kernels

Kernel	Formula
p-step random walk	$K = (aI - \tilde{L})^p$
Diffusion process	$K = \exp(-\sigma^2/2\tilde{L})$
Regularized Laplacian	$K = (I + \sigma^2\tilde{L})^{-1}$
Commute time kernel	$K = L^+$
Inverse cosine	$\cos\tilde{L}\pi/4$

Definition of graph kernels based on the normalized Laplacian ($\tilde{L}$), and pseudoinverse of the Laplacian (L^+), where a, p, and σ are given parameters.

host proteins box in Figure 4B). We obtain prediction scores simultaneously for all drugs with the following matrix multiplication $S_d = TKh$ (also illustrated in Figure 4C), resulting in a vector of drug scores, S_d.

Evaluation

To evaluate the performance of our methods, we used three different sources of evidence from ongoing research: *in vitro* experiments, clinical trials, and CMAP scores. These sources are independent of each other; hence they can be used to provide an independent evaluation of the efficacy of repurposing methods. Note that none of these three sources of evidence can be considered a gold standard, as none of them can ensure therapeutic effects for COVID-19 patients. Yet, they represent a proxy of effectiveness of drugs for COVID-19.

In vitro experiments involving drugs with antiviral efficacy indicate their potential to be effective at reducing viral infection and replication in the host cell. Evaluating our models with this kind of evidence allows us to assess whether they prioritize drugs with molecular antiviral efficacy versus other drugs.

Clinical trial studies are used to assess pharmacokinetics, dosage, therapeutic efficacy, and safety of drugs.[43] Each phase in clinical trials involves an increasing number of patients, thus achieving higher statistical significance while minimizing the number of patients that risk developing side effects.[44] Indicating a drug in a clinical trial requires satisfying several conditions set by biologists and medics, and arguments of why it might be effective. This suggests the investigators believe that the drug is safe and a potential candidate to treat the disease. Evaluating our models with clinical trial evidence allows us to determine if they prioritize drugs that would be included in such trials.

We use the CMAP[15,16] to contrast changes in gene expression levels caused by a drug (drug expression profile) with changes induced by SARS-CoV-2 infection (disease expression profile). The hypothesis is that, if a drug expression profile is opposite to a disease expression profile, then it could potentially "revert" the disease signature and have therapeutic effects—this idea has already been used before[15,42] to predict new therapeutic indications for drugs and has also been applied to COVID-19.[45,46] Therefore, evaluating our models with this source of evidence allows us to assess whether they prioritize drugs with potentially therapeutic effects.

For the matrix decomposition approach, the evaluation was carried out using the 126 BSAs in the drug-virus dataset.[17] For the network medicine approach, the evaluation was done on an interactome of 18,505 proteins with 327,924 interactions.[13] With this approach we ranked 2,197 approved drugs from DrugBank.[47]

We used the types of evidence described above to create three datasets where drugs were classified as either effective or non-effective for COVID-19 (see experimental procedures). This allowed us to assess the performance of a prediction method by formulating a binary classification problem, where the task is to discriminate the two sets of drugs, and then calculating binary classification metrics based on the analysis of the confusion matrix.

However, we note that the lack of a set of drugs with proven therapeutic effect against COVID-19 (i.e., a gold standard), poses a challenge for this type of evaluation—this problem has also been described before, e.g., in Zhou et al.[48] and Gysi et al.[13] We hypothesized that drugs with evidence against COVID-19 should behave differently from the remaining drugs. This hypothesis has an actionable consequence: a method can be evaluated by assessing whether it can discriminate between the two groups of drugs (effective and non-effective)—if it can, this is an indication that we can possibly trust the predictions it makes. Therefore, together with traditional metrics for binary classification, we also assessed whether prediction methods provided scores that were statistically different for the two classes of drugs. Our results (Figures 5A, 5B, and 5F–5H) show that the differences between the scores are significant for our matrix decomposition approach as well as our kernel methods across several evaluation settings. We observe that other network-based methods do not pass this test with such consistency (see Notes S5–S7). In the following, we present the results for each type of evidence, separately.

In vitro *evaluation*

Of the 126 BSAs in the drug-virus dataset, 10 have shown *in vitro* efficacy against SARS-CoV-2.[13,49] In our evaluation, these drugs were removed one at a time from the drug-virus matrix Y (by setting the corresponding entry to zero). We then trained our matrix decomposition model, and scores were predicted for all the drugs. We used the Wilcoxon-Mann-Whitney p value to assess the difference between the scores obtained for those 10 drugs and the rest of the drugs. Figure 5A shows that our matrix decomposition method significantly assigns higher scores to BSAs with *in vitro* efficacy (Wilcoxon-Mann-Whitney p value = 4.92×10^{-7}). Precision and recall are shown in Figure S2 (Note S1).

Scores predicted by the kernel-based methods are shown in Figure 5F. Of the 2,197 FDA-approved drugs considered by our network medicine approach, 81 have shown *in vitro* efficacy against SARS-CoV-2.[13,49] We observed that the scores of drugs with *in vitro* efficacy against SARS-CoV-2 are significantly higher than those of the remaining drugs for all kernels and the average ranking (avgRank).

In Figure 5C, we show that the kernel-based methods performed better than the competitors for the *in vitro* evaluation. The recall@150 of the average ranking is 49.71% higher than DSD, and 110.57% higher than the Guney distance. The precision@150 of the average ranking is 50.54% higher than DSD, and 108.96% higher than the Guney distance.

Clinical trial evaluation

Of the 126 BSAs in the drug-virus dataset, 28 are in clinical trials (see experimental procedures). Figure 5C shows that prediction scores by our matrix decomposition method are significantly

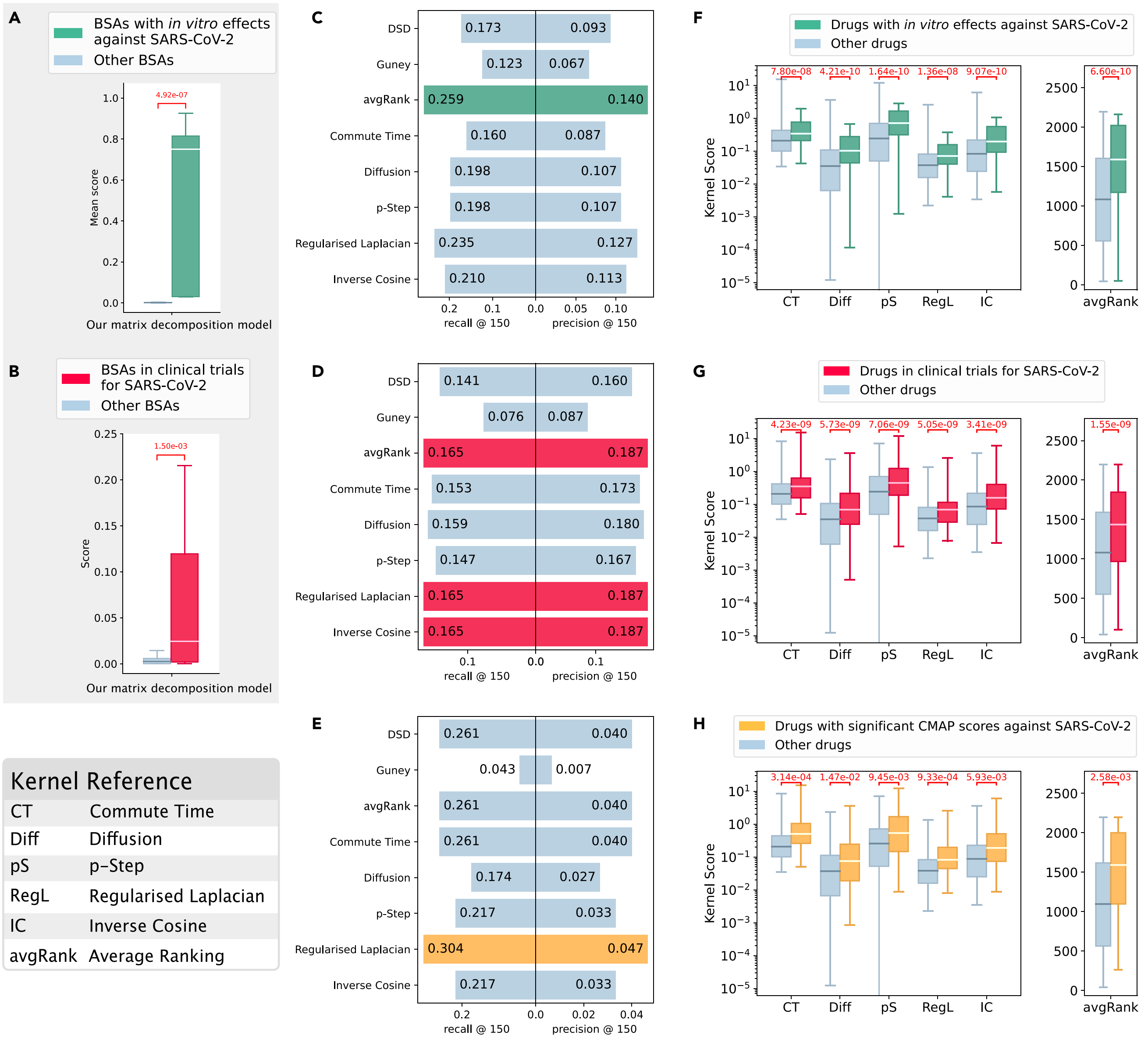

Figure 5. Analysis of the predictions for COVID-19

We used three different sources of evidence: *in vitro* (A, C, and F), clinical trials (B, D, and G), and CMAP (E and H). We compared scores for drugs with evidence of efficacy against SARS-CoV2 versus scores for the remaining drugs. Our matrix factorization model (A and B) and kernel-based methods (F, G, and H) provide scores that are significantly different between the two groups of drugs in every case (Wilcoxon-Mann-Whitney $p < 0.05$). We formulated a binary classification problem to discriminate between drugs with evidence of efficacy against SARS-CoV2 and the remaining drugs. (C, D, and F) Comparison of precision and recall at top 150 for our kernel-based methods (commute time, diffusion, *p*-step, regularized Laplacian, and inverse cosine kernels, and avgRank), DSD, and Guney's distance. The highest values are colored.

higher for drugs in clinical trials (Wilcoxon-Mann-Whitney p value = 1.5×10^{-3}). Our method can recover 50% of the correct BSAs in the top-20 predictions retrieved (see Figure S2 in Note S1).

Scores predicted by the kernel-based methods are shown in Figure 5G. Of the 2,197 FDA-approved drugs considered by our network medicine approach, 170 are in clinical trials. We observed that the scores of drugs in clinical trials for COVID-19 are significantly higher than those of the remaining drugs for all kernels and the average ranking (avgRank).

In Figure 5D, we show that the kernel-based methods performed better than the competitors for the clinical trials evaluation. The recall@150 of the average ranking is 17.02% higher than DSD, and 117.11% higher than the Guney distance. The precision@150 of the average ranking is 16.88% higher than DSD, and 114.94% higher than the Guney distance.

CMAP evaluation

We queried CMAP[15,16] obtaining a list of 23 FDA-approved drugs that present an expression profile opposite to the one expressed by SARS-CoV-2 infected cells with a τ score between

−90 and −100 (see experimental procedures). Figure 5H shows that the scores of FDA-approved drugs with strongly negative CMAP correlation are significantly higher than those of the remaining drugs for all kernels and the average ranking (avgRank).

In Figure 5E, we compared the performance of the kernel-based methods and competitors for the CMAP evaluation. Our average ranking has the same performance as DSD and better performance than Guney's distance (recall@150 is 50.72% higher, and precision@150 is 47.43% higher). The regularized Laplacian kernel had the best performance, with recall@150 16.48% higher than DSD, and 60.7% higher than Guney's distance, and precision@150 17.5% higher than DSD, and 57.14% higher than Guney's distance.

On the importance of integrating transcriptomics data. An interesting question is whether weighting host proteins by differential expression improves our network medicine approach. To answer this, we compared results based on weighted host proteins and unweighted/binary host proteins. For *in vitro* and clinical trials evidence, we observed that the Wilcoxon-Mann-Whitney p values are smaller (more significant) when using weighted host proteins when compared with considering all host proteins equally. The recall@150 and precision@150 are consistently higher when we use weights for the three types of evidence. These results are presented in Note S8.

Our results hold for different PPI networks and evaluation settings. An important question is whether results are consistent across different interactomes and how sensitive they are to different choices of the PPI network. We re-computed the kernel-based scores using the recently released HuRI PPI[50] as well as the interactome compiled by Cheng et al.[30] Results are presented in Note S7. For most of the kernels, FDA-approved drugs with *in vitro*, and clinical trials evidence have a significantly higher prediction score than the remaining drugs. For the three sources of evidence, the kernel-based methods have the higher recall@150 and precision@150 when compared with competitors. This indicates that our results have a high consistency across different interactomes.

Comparison with the approaches by Gysi et al.. We also extensively compared our kernel methods with the methods recently proposed by Gysi et al.,[13] although the comparison could only be carried on the Gysi et al. dataset—this consists of 918 drugs including approved, investigational, experimental, nutraceutical, and withdrawn drugs. Overall, our kernel methods perform better with respect to *in vitro* and CMAP evidence—note that, in several cases, the scores obtained by the Gysi et al. methods for sets of effective and ineffective drugs are not significantly different. GNN methods perform better than kernel methods only with respect to clinical trial evidence. A summary of the different datasets used can be found in Note S4. A detailed description of all the experiments comparing our approaches with those from Gysi et al. is presented in Note S6.

CoREx

As a further way to evaluate drug repurposing against SARS-CoV-2, we developed CoREx, a web-based tool that enables scientists to study drug repurposing in a functional context on the interactome. Given a set of drug targets, CoREx offers the users a panoramic point of view that puts together several biologically relevant contexts (i.e., functional relationships, PPIs, clinical trial status, CMAP scores, and drug's anatomical therapeutic chemical [ATC] categories). Our goal is to assist researchers to reason about drug alternatives, drug combinations, and mechanisms of actions by analyzing the interplay between drug targets and host proteins in these different contexts.

Centered around ideas from network medicine, CoREx provides two different tools: the *functional analysis tool* and *interactome analysis tool*. The functional analysis tool allows the user to study the relationships between drug targets and host proteins. A functional interactome is built by integrating protein networks available in the STRING database[51] in a way that maximizes the probability that two interacting proteins share functional characteristics (see Note S10 for details on the network combination). Then, we use the ClusterONE algorithm[52] to identify functionally similar groups of proteins, and filter those that contain at least one SARS-CoV-2 host protein, and at least one drug target. The functional enrichment of these groups is then analyzed using Enrichr.[53] All the drugs that interact with the module through their targets are enriched with their ATC categories, CMAP evidence, and clinical trial status against COVID-19. All of these results are presented to the user in a user-friendly interactive graphical interface, as shown in Figure 6.

The interactome analysis tool allows the user to visualize the perturbation caused by a drug on the SARS-CoV-2 host protein subnetwork. When a drug is selected, each node (host protein) is colored based on the strength of the resulting kernel score. This tool complements CoVex, by Sadegh et al.,[54] which analyzes the interplay within the virus-host-drug triad using paths on the interactome. Instead, CoREx calculates the effects that drugs have on individual host proteins through the different graph kernels. We have preloaded our interactome analysis tool with those FDA-approved drugs that have available drug targets from DrugBank.[47] Users can also submit a list of drug targets, and visualize the perturbation that a hypothetical drug (or drug combination) with those targets would have on the host proteins subnetwork.

CoREx is available at https://paccanarolab.org/corex and supporting datasets are updated every 2 weeks. The project is also open-source, and the repository is publicly available at https://github.com/paccanarolab/corex.

DISCUSSION

The development of computational approaches that can assist in the rational and fast discovery of treatments is critical for emergent infectious diseases such as COVID-19.[1–3,6,48] Drug repositioning, the re-use of drugs already on the market, can help to speed up the development of such treatments by prioritizing known safe-in-human drugs for clinical trials involving COVID-19 patients. In this paper, we proposed two machine learning approaches that can assist in the prioritization of drugs, together with a human-in-the-loop website tool, CoREx, to assist current research efforts for finding drugs with therapeutic efficacy against SARS-CoV-2.

Li and De Clercq[4] indicated that finding potential repositioning candidates for COVID-19 should be focused on two main strategies: virally targeted agents and host-targeted agents. Our matrix decomposition approach is aimed at the first repositioning strategy, whereas our network medicine approach, together with CoREx, is aimed at the second one. Our first approach ranks 126 BSAs by their predicted efficacy against SARS-CoV-2, and

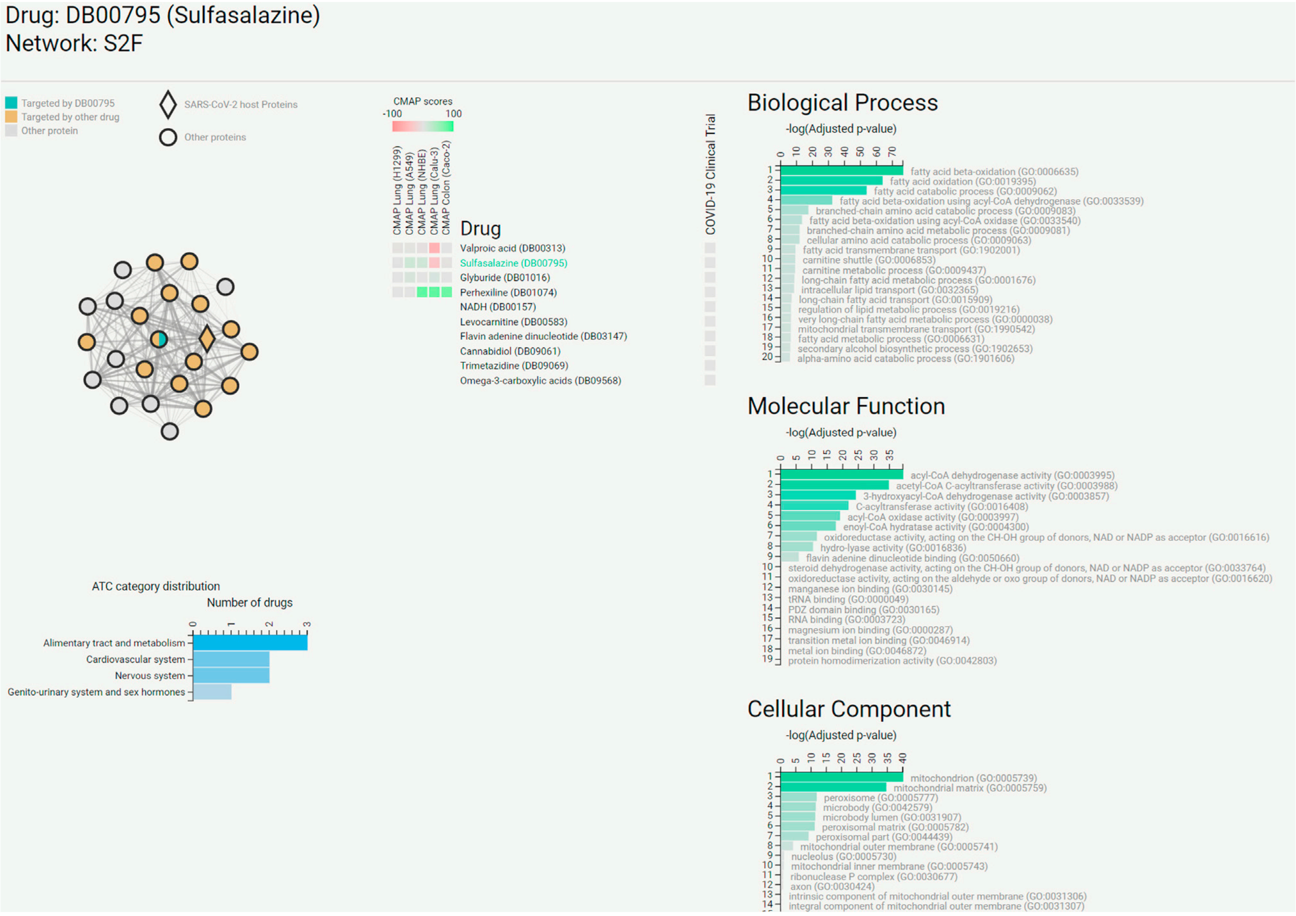

Figure 6. Screenshot of CoREx displaying a functional module for Sulfasalazine (highlighted in green in the "Drug" list)
The module is depicted as a network on the top left where nodes represent proteins, edges represent shared functional characteristics, and the thickness of the edges represents the strength of such functional similarity. Host proteins are depicted as diamonds, drug targets are colored. The list of drugs with at least one target in this functional module is presented in the center, alongside CMAP scores for five cell lines (on the left), and an indicator of whether the drug is currently in clinical trials (on the right). The bar plots on the right part correspond to the functional enrichment scores for each GO domain. The bar plot on the bottom left section of the image summarizes the ATC categories of the drugs targeting this functional module.

our second approach ranks 2,197 therapeutically diverse FDA-approved drugs by their predicted ability to perturb the COVID-19 disease module.

The objective function of our matrix decomposition approach in Equation 1 is inspired by our recent work to predict the frequencies of drug side effects.[55] The main feature of this new model is that it can account for varying levels of uncertainties in the data. We realized that different levels of drug developmental evidence can be thought of as indicating different levels of confidence in drug-virus associations and can be interpreted as probabilities. Our new model exploits the richness of this information and its outputs can be interpreted as probabilities of drug approval. Experiments in which we randomized or removed information about drug developmental stages show that such information is key to achieve a good performance (see Note S3). The implementation of our algorithm is freely available: https://github.com/paccanarolab/DrugRepoCOVID.

Our network medicine approach aims at prioritizing FDA-approved candidates based on their network-modulated effects on the COVID-19 disease protein module. In contrast to our first approach, our network medicine approach does not explicitly model the clinical efficacy of drugs, but rather their mechanistic effects on the protein interaction network. This means that a high score points to a high probability for the drug to perturb the disease module. Note, however, that our kernel methods, like most network-based approaches,[13] can quantify the perturbation on the interactome, but cannot predict in which way the host will ultimately be affected by such perturbations (see Note S11.2).

An important advantage of our kernel approaches is that they offer a natural way to integrate gene expression data and thus allow us to focus the models on particular proteins that play a key role in the infection. Our experiments show that the integration of transcriptomics data improves the results (see Note S8). Furthermore, we have shown that our kernels have similar performance across multiple interactomes (see Note S7).

We have shown that our predictions from both approaches are aligned to ongoing *in vitro* experiments and clinical trial studies. An interesting question is whether there is additional biological evidence of efficacy for the best scoring drugs from our

CellPress
OPEN ACCESS

approaches. We manually curated the top 20 predicted drugs obtained from each approach. Our analysis reveals that many of these drugs are linked to ongoing efforts against COVID-19: several top-ranked BSAs from our matrix decomposition model are part of ongoing clinical trials for COVID-19, or are even already approved for compassionate use in COVID-19 patients[56–59]; several top-ranked drugs from our network medicine approach have also shown efficacy either as therapeutic alternatives or as instruments for reducing risk of infection and transmission.[60–63] An in-depth analysis of the top 20 predictions, including an analysis of their ATC classification and references to the literature, is presented in Note S11. A comparison with the set of drugs predicted by Gordon et al.[14] is also provided in Note S9. Finally, while the datasets that we used in our two approaches are different, a few drugs could be predicted by both methodologies—these are analyzed in Note S12.

Our computational approaches leverage available data to produce the predictions. As more reliable data becomes available, we expect the performance of our models to increase accordingly. Recently, COVID-19 atlases have been published, including single-cell transcriptomics data[64,65] that could be exploited with our approaches.

We also point out that, while we have developed and tested our two approaches for COVID-19, both of them are disease agnostic. The general principles underlying our matrix decomposition and network medicine approaches will remain valid for any other viral disease, and therefore our methods could be applied for drug repurposing in these scenarios, as long as the data are available.

Finally, the integration of heterogeneous sources of omics information with multiple layers of interconnection is a challenge in itself. Prime examples of such complex data are the molecular datasets involved in drug repositioning. We built CoREx (https://paccanarolab.org/corex) with the goal of providing the research community with a tool for the analysis and the formulation of hypothesis about drugs that can be repurposed for COVID-19. CoREx combines transcriptomics, proteomics, and functional information about the human genome together with knowledge about drugs and their protein targets, and we make it available for the scientific community.

Limitations of the study

Our matrix decomposition approach is applicable to any drug for which the developmental stage associating it to a viral disease is known. The drug may or may not be virally targeted, and the model itself will not impose such a restriction. The main limitation of the method is that it relies on drug-virus associations annotated with their stage of development, and publicly available data of this type is currently scarce—we only found this type of information in the manually curated dataset by Andersen et al.[17] that we used in our study. The main limitation of our network medicine approach is that it can only be applied to drugs with known protein targets on the host interactome.

EXPERIMENTAL PROCEDURES

Resource availability

Lead contact

The lead contact for this work is Alberto Paccanaro (alberto.paccanaro@rhul.ac.uk).

Materials availability

This study did not generate new unique reagents.

Data and code availability

Original data have been deposited to Mendeley Data: https://doi.org/10.17632/p7y5wmschg.1. The implementation of our matrix factorization model can be found at https://github.com/paccanarolab/DrugRepoCOVID. CoREx is available at https://paccanarolab.org/corex, and the source code is publicly available at https://github.com/paccanarolab/corex.

Datasets

- *The drug-virus dataset.* We used the dataset curated by Andersen et al.[17] (downloaded April 6, 2020). Drugs were mapped to DrugBank IDs, when available. Each drug-virus association was annotated with their developmental status/stage. There are eight stages of development in the dataset, namely: cell culture/co-culture, primary cells/organoids, animal model, clinical trials phase I, phase II, phase III, phase IV, and approved. In total, our dataset contains 850 associations between 126 BSAs and 80 viruses.
- *Protein interaction network.* The PPI network was obtained from Gysi et al.,[13] which contains 18,505 human proteins, and 327,924 interactions.
- *FDA-approved drugs and drug targets.* FDA-approved drugs and their drug targets were retrieved from DrugBank[47] and Gysi et al.[13] Our set of drugs consisted of 2,197 FDA-approved drugs consisted. Our set of drug target associations consisted of 14,941 pairs of drug and targets.
- *Host proteins.* Our COVID-19 disease module consisted of 336 host proteins. It includes 332 host proteins reported by Gordon et al.,[14] the entry receptor ACE2,[66] and three SARS-CoV-2 entry-associated proteases TMPRSS2,[67] CTSB, and CTSL.[68]
- *Gene expression data.* To weight the host proteins in our kernel-based methods, we used gene expression data from 430 COVID-19 patients, and 54 controls, collected from nasopharyngeal swabs.[69] The RNA-seq raw counts are available in the Gene Expression Omnibus (GEO),[70,71] with accession number GSE152075. We processed the data using the edgeR package,[72] and obtained the absolute value of the log fold change comparing the expression levels between COVID-19 patients and controls. For 47 host proteins with missing mRNA levels, we assigned the minimum absolute value of the log fold change. The final weights of the host proteins are available from Mendeley Data (see Table S7).
- *In vitro data.* We built a binary dataset, assigning positive labels to drugs that were reported to show efficacy against SARS-CoV-2 infection *in vitro*, and negative labels to all other drugs. Data for drug efficacy *in vitro* was built as the union of experiments reported by Riva et al.[49] and Gysi et al.[13] Eighty-one FDA-approved drugs show *in vitro* effects (see Table S7).
- *Clinical trials data.* We built a binary dataset and assigned positive labels to drugs that are involved in clinical trial studies, and negative labels to all other drugs. Information for clinical trials studies was downloaded from ClinicalTrials.gov on December 1, 2020.[73] Drugs were mapped to the DrugBank database[47] by matching their names (see Table S7).
- *CMAP data.* For the CMAP query, we used a COVID-19 signature by Ghandikota et al.[74] This gave us a list of 106 genes upregulated and 41 genes downregulated in three different models of SARS-CoV-2 infection from transcriptomics data. Two are models *in vitro* (Calu-3 and Vero E6 cells), and one model is *in vivo* (Ad5-hACE2-sensitized mice). The query with these data resulted in 30 drugs with significant negative τ score ($\tau < -90$) that were mapped to DrugBank. Twenty-three of these 30 drugs are FDA approved and have targets in the Gysi et al. interactome. The list of 30 drugs with CMAP evidence is available from Mendeley Data (see Table S7). All supplementary files available from Mendeley data and external data sources are listed in Table S7 (Note S13).

The multiplicative learning algorithm for the matrix decomposition model

To minimize Equation 1 subject to non-negative constraints, we developed an efficient multiplicative learning algorithm inspired by the diagonally rescaled

principle of NMF.[21] The algorithm consists of iteratively applying the following multiplicative update rules:

$$P_{ia} \leftarrow P_{ia} \frac{\left(\left[M^A \circ Y + \sum_{s \in \{B,C,D,E\}} \alpha_s (M^s \circ Y) \right] Q^T \right)_{ia}}{\left(\left[M^A \circ (PQ) + \sum_{s \in \{B,C,D,E\}} \alpha_s M^s \circ (PQ) + \alpha_z M^z \circ (PQ) \right] Q^T \right)_{ia}}$$

$$Q_{aj} \leftarrow Q_{aj} \frac{\left(P^T \left[M^A \circ Y + \sum_{s \in \{B,C,D,E\}} \alpha_s (M^s \circ Y) \right] \right)_{aj}}{\left(P^T \left[M^A \circ (PQ) + \sum_{s \in \{B,C,D,E\}} \alpha_s M^s \circ (PQ) + \alpha_z M^z \circ (PQ) \right] \right)_{aj}}. \quad \text{(Equation 2)}$$

Following the guidelines to implement NMF,[75] a small number $\varepsilon = 10^{-8}$ was added to the denominators in Equation 2 to prevent division by zero, and we initialized P and Q as random dense matrices uniformly distributed in the range $[0, 0.1]$. Furthermore, to avoid the well-known degeneracy[20] associated with the invariance PQ under the transformation $P \rightarrow P\Lambda$ and $Q \rightarrow \Lambda^{-1}Q$, for a diagonal matrix Λ, we normalized P at each iteration as follows:

$$Q_{aj} \leftarrow \frac{Q_{aj}}{q_a}, \quad \text{(Equation 3)}$$

where q_a denotes the ath row vector of Q.

The stopping criteria of our algorithm was based on the maximum tolerance of the relative change in the elements of P and Q. The default value was $tolX < 10^{-3}$, which occurred typically in about 1,000 iterations for $k = 5$.

Using a similar procedure to Galeano and Paccanaro,[55] it can be easily shown that our algorithm in Equation 2 satisfies the KKT conditions of convergence.

Cross-validation procedure and model selection for the matrix decomposition approach

We used a LOOCV procedure to evaluate the performance of our matrix decomposition model. To set the model hyperparameters: k, α_E and α_Z, we performed LOOCV on the drug-virus associations with clinical trials developmental stages (validation set). We performed a grid-search and selected the hyperparameters that maximize the mean recall across the top 1, 5, 10, 15, 20, 25, and 30 predictions retrieved. We found that $k = 5$, $\alpha_E = 0.01$, and $\alpha_Z = 2$ provided a good performance. The other hyperparameters of our model were set based on the probabilities of success reported by Dowden and Munro[25] for anti-infective drugs on distinct phases of clinical trials, i.e., $\alpha_B = 0.16$ (phase I), $\alpha_C = 0.27$ (phase II), and $\alpha_D = 0.71$ (phase III). Having set all these hyperparameters, we performed an LOOCV on the test set corresponding to drug-virus associations that have been approved or are in phase IV of clinical trials. The model selection for the competitors was performed on the same validation sets (see details in Note S1).

The trained model that we used in the "Evaluation" section was obtained by training the model 1,000 times using all the available data with optimal hyperparameters. We then selected the solution that gave the lowest value in the loss function.

Graph kernels

A PPI network is represented by a graph $G = (V, E)$, in which $V = \{1, 2, \ldots, n_V\}$ is the set of nodes (proteins), and E the a set of links connecting the nodes (protein interactions). If the graph is weighted, then for each edge $e \in E$ we associate a non-negative real value $w(e)$. Let $\mathcal{H} \in V$ denote the set of host proteins. Our goal is then to perturb the subnetwork induced by $\mathcal{H}$, i.e., the host protein subnetwork.

Here, we rely on different graph kernels described in the literature.[35,36,76] In the following, graph kernels and their properties are defined as in Kondor and Vert.[77] A graph kernel $k : V \times V \mapsto \mathbb{R}$ provides a similarity metric on the set of nodes V based on the graph structure. It is positive definite, that is, for any $i, j \in V$ and any $c_i, c_j \in \mathbb{R}$ we have that $\sum_{i=1}^{n_V} \sum_{j=1}^{n_V} c_i c_j k(i,j) \geq 0$.

We can use it to define distances or similarities on a latent feature space. More specifically, there exists the feature mapping $\varphi : V \mapsto \mathcal{F}$ such that $k(i,j) = \langle \varphi(i), \varphi(j) \rangle$ for all $i, j \in V$. A graph kernel can be represented by an $n_V \times n_V$ matrix K whose elements correspond to $K_{i,j} = k(i,j)$ for every $i, j \in V$. It is usually defined in terms of the normalized Laplacian, which we explain below.

Let W be an $n_V \times n_V$ matrix denoting the weighted adjacency matrix of G. That is, $W_{i,j} = w(e)$ if there is an edge e connecting i and j, and $W_{i,j} = 0$, otherwise. If G is unweighted, we assume that $w(e) = 1$ for every edge $e \in E$. Let D denote an $n_V \times n_V$ diagonal matrix in which each diagonal element corresponds to the node degree, that is, $D_{i,i} = \sum_{j=1}^{n_V} W_{i,j}$ for every $i \in V$. The Laplacian is defined as $D - A$, and its pseudoinverse (Moore-Penrose inverse) is denoted by L^+. The normalized Laplacian is defined as $\tilde{L} := I - D^{-\frac{1}{2}} W D^{-\frac{1}{2}}$, where I denotes the identity matrix.

There are different ways to define K and we focus on five graph kernels[35,36,76]: regularized Laplacian, diffusion process, and p-step random walk in terms of the normalized Laplacian[36] (see Table 1).

In the p-step random walk, $p \geq 1$ and $a \geq 2$ are given parameters.[36] The element $K_{i,j}$ measures how likely it is to go from node i to node j after p steps in a random walk. If we generalize it to a continuous time (infinitesimally small steps) and take an infinite number of steps, we have the diffusion process $K = \exp(-\sigma^2 / 2\tilde{L})$, where σ is a parameter controlling the diffusion. Finally, the regularized Laplacian kernel can be thought of as the convergence of an iterative process in which nodes spread information to their neighbors at each step.

We used different kernels from Smola and Kondor,[36] Cao et al.,[35] and Zhou et al.,[76] which are implemented in the R package *diffuStats*[78] for the commute time, diffusion, p-step, regularized Laplacian, and inverse cosine kernels. We set the parameter p to 2 for the p-step kernel. For the remaining kernels, we used the default parameters in *diffuStats*.

CMAP evaluation details

We consider that a drug has CMAP evidence against COVID-19 if the changes that it causes to gene expression are opposite to the ones caused by the disease.[15] To build the CMAP evaluation set, we used the CMAP pipeline[15,16] to measure how similar or opposite the drug and COVID-19 expression profiles are. We used version 1.0 of the CMAP L1000 dataset[16] available on clue.io website (https://clue.io/).

We began by obtaining a list of up-/downregulated genes in COVID-19 (genes that have higher/lower expression levels in SARS-CoV-2 infected cells compared with non-infected cells). Then, we queried the COVID-19 signature in CMAP. For each drug, CMAP has a list of genes ordered from the most expressed to the least expressed after treatment (in comparison with the expression levels with no treatment). If the upregulated genes in COVID-19 are located on the bottom of the list (that is, if they have low expression levels in cells treated with the drug), and the downregulated genes are located on the top (that is, they have high expression levels in cells treated with the drug), we say that the drug and disease signatures have a strong negative correlation. If we observe the opposite (upregulated genes on top, and downregulated genes on bottom), we say that they have a strong positive correlation.

For each drug, CMAP outputs an enrichment score that is positive when the correlation between the drug and disease signatures is positive (the drug mimics the disease), and negative when the correlation is negative (the drug reverses the disease). The final values (denoted by τ) are compared with a reference database and normalized between -100 and 100.

SUPPLEMENTAL INFORMATION

Supplemental information can be found online at https://doi.org/10.1016/j.patter.2021.100396.

ACKNOWLEDGMENTS

We thank Gloria Aguilar, Martin Aguero, Rafael Adorno, Aldo Galeano, Diego Stalder, Justin Reese, Afshin Beheshti, Thiago Moreno L. Souza, Carolina de Queiroz Sacramento, Claudio Struchiner, Valdiléa Veloso, Beatriz Grinsztejn, Irina Yuri Kawashima, Teresa Gamarra, Ruben Jimenez, Santiago Noto, and Philip Ovington for useful discussions. We also thank all members of the COVID-19 International Research Team (www.cov-irt.org) for their helpful feedback during weekly meetings. A.P. was supported by Biotechnology

CellPress
OPEN ACCESS

and Biological Sciences Research Council (https://bbsrc.ukri.org/) grants BB/K004131/1, BB/F00964X/1, and BB/M025047/1; Medical Research Council (https://mrc.ukri.org/) grant MR/T001070/1; Consejo Nacional de Ciencia y Tecnología Paraguay - CONACyT (http://www.conacyt.gov.py/) grants 14-INV-088 and PINV15-315; National Science Foundation Advances in Bio Informatics (https://www.nsf.gov/) grant 1660648; Fundação de Amparo à Pesquisa do Estado do Rio de Janeiro grant E-26/201.079/2021 (260380); and the School of Applied Mathematics (EMAp), Fundação Getulio Vargas. S.d.S.S., M.T., and D.G. were supported by the School of Applied Mathematics (EMAp), Fundação Getulio Vargas.

AUTHOR CONTRIBUTIONS

S.d.S.S., M.T., D.G., L.C., and A.P. conceived the study, designed the methods, and analyzed the results. S.d.S.S., M.T., and D.G. implemented and conducted the experiments. M.T. and M.d.M.S. implemented CoREx. S.d.S.S., M.T., D.G., and A.P. wrote the manuscript. All authors reviewed the manuscript. A.P. supervised the project.

DECLARATION OF INTERESTS

The authors declare no competing interests.

Received: May 18, 2021
Revised: June 21, 2021
Accepted: November 1, 2021
Published: November 3, 2021

REFERENCES

1. Ashburn, T.T., and Thor, K.B. (2004). Drug repositioning: identifying and developing new uses for existing drugs. Nat. Rev. Drug Discov. *3*, 673–683.
2. Pushpakom, S., Iorio, F., Eyers, P.A., Escott, K.J., Hopper, S., Wells, A., Doig, A., Guilliams, T., Latimer, J., McNamee, C., et al. (2018). Drug repurposing: progress, challenges and recommendations. Nat. Rev. Drug Discov. *18*, 41–58.
3. Zumla, A., Chan, J.F.W., Azhar, E.I., Hui, D.S.C., and Yuen, K.-Y. (2016). Coronaviruses —drug discovery and therapeutic options. Nat. Rev. Drug Discov. *15*, 327–347. https://doi.org/10.1038/nrd.2015.37. https://www.nature.com/articles/nrd.2015.37.
4. Li, G., and De Clercq, E. (2020). Therapeutic options for the 2019 novel coronavirus (2019-nCoV). Nat. Rev. Drug Discov. *19*, 149–150.
5. Sanders, J.M., Monogue, M.L., Jodlowski, T.Z., and Cutrell, J.B. (2020). Pharmacologic treatments for coronavirus disease 2019 (COVID-19): a review. JAMA *323*, 1824–1836. https://doi.org/10.1001/jama.2020.6019.
6. Mei, M., and Tan, X. (2021). Current strategies of antiviral drug discovery for COVID-19. Front. Mol. Biosci. *8*, 310. https://doi.org/10.3389/fmolb.2021.671263. https://www.frontiersin.org/article/10.3389/fmolb.2021.671263.
7. Dolgin, E. (2021). The race for antiviral drugs to beat COVID—and the next pandemic. Nature *592*, 340–343. https://doi.org/10.1038/d41586-021-00958-4. https://www.nature.com/articles/d41586-021-00958-4.
8. Barabási, A.-L., Gulbahce, N., Loscalzo, J., and Network Medicine. (2011). A network-based approach to human disease. Nat. Rev. Genet. *12*, 56–68. https://doi.org/10.1038/nrg2918. https://www.ncbi.nlm.nih.gov/pmc/articles/PMC3140052/.
9. Cáceres, J.J., and Paccanaro, A. (2019). Disease gene prediction for molecularly uncharacterized diseases. PLoS Comput. Biol. *15*, e1007078.
10. Silverman, E.K., Schmidt, H.H.H.W., Anastasiadou, E., Altucci, L., Angelini, M., Badimon, L., Balligand, J.-L., Benincasa, G., Capasso, G., Conte, F., et al. (2020). Molecular networks in network medicine: development and applications. WIREs Syst. Biol. Med. *12*, e1489. https://doi.org/10.1002/wsbm.1489. https://onlinelibrary.wiley.com/doi/abs/10.1002/wsbm.1489.
11. Sharma, A., Menche, J., Huang, C.C., Ort, T., Zhou, X., Kitsak, M., Sahni, N., Thibault, D., Voung, L., Guo, F., et al. (2015). A disease module in the interactome explains disease heterogeneity, drug response and captures novel pathways and genes in asthma. Hum. Mol. Genet. *24*, 3005–3020. https://doi.org/10.1093/hmg/ddv001.
12. Wang, R.-S., and Loscalzo, J. (2018). Network-based disease module discovery by a novel seed connector algorithm with pathobiological implications. J. Mol. Biol. *430*, 2939–2950. https://doi.org/10.1016/j.jmb.2018.05.016. https://www.sciencedirect.com/science/article/pii/S0022283618304273.
13. Gysi, D.M., Valle, I.d., Zitnik, M., Ameli, A., Gan, X., Varol, O., Ghiassian, S.D., Patten, J.J., Davey, R.A., Loscalzo, J., and Barabási, A.-L. (2021). Network medicine framework for identifying drug-repurposing opportunities for COVID-19. PNAS *118*. e2025581118. https://doi.org/10.1073/pnas.2025581118. https://www.pnas.org/content/118/19/e2025581118.
14. Gordon, D.E., Jang, G.M., Bouhaddou, M., Xu, J., Obernier, K., White, K.M., O'Meara, M.J., Rezelj, V.V., Guo, J.Z., Swaney, D.L., Tummino, T.A., et al. (2020). A SARS-CoV-2 protein interaction map reveals targets for drug repurposing. Nature *583*, 459–468. https://doi.org/10.1038/s41586-020-2286-9. https://www.nature.com/articles/s41586-020-2286-9.
15. Lamb, J., Crawford, E.D., Peck, D., Modell, J.W., Blat, I.C., Wrobel, M.J., Lerner, J., Brunet, J.-P., Subramanian, A., Ross, K.N., et al. (2006). The Connectivity Map: using gene-expression signatures to connect small molecules, genes, and disease. Science *313*, 1929–1935. https://www.science.org/doi/abs/10.1126/science.1132939.
16. Subramanian, A., Narayan, R., Corsello, S.M., Peck, D.D., Natoli, T.E., Lu, X., Gould, J., Davis, J.F., Tubelli, A.A., Asiedu, J.K., et al. (2017). A next generation Connectivity Map: L1000 platform and the first 1,000,000 profiles. Cell *171*, 1437–1452.e17. https://doi.org/10.1016/j.cell.2017.10.049.
17. Andersen, P.I., Ianevski, A., Lysvand, H., Vitkauskiene, A., Oksenych, V., Bjørås, M., Telling, K., Lutsar, I., Dampis, U., Irie, Y., et al. (2020). Discovery and development of safe-in-man broad-spectrum antiviral agents. Int. J. Infect. Dis *93*, 268–276.
18. Cremonesi, P., Koren, Y., and Turrin, R. (2010). Performance of recommender algorithms on top-n recommendation tasks. In Proceedings of the Fourth ACM Conference on Recommender Systems, pp. 39–46.
19. Galeano, D., Li, S., Gerstein, M., and Paccanaro, A. (2020). Predicting the frequencies of drug side effects. Nat. Commun. *11*, 1–14.
20. Lee, D.D., and Seung, H.S. (1999). Learning the parts of objects by non-negative matrix factorization. Nature *401*, 788–791.
21. Lee, D.D., and Seung, H.S. (2001). Algorithms for non-negative matrix factorization. In Advances in Neural Information Processing Systems, T. Leen, T. Dietterich, and V. Tresp, eds. (Advances in Neural Information Processing Systems), pp. 556–562.
22. Bakal, G., Kilicoglu, H., and Kavuluru, R. (2019). Non-negative matrix factorization for drug repositioning: experiments with the repoDB dataset. In AMIA Annual Symposium Proceedings, *2019* (American Medical Informatics Association), p. 238.
23. Ceddia, G., Pinoli, P., Ceri, S., and Masseroli, M. (2020). Matrix factorization-based technique for drug repurposing predictions. IEEE J. Biomed. Health Inform. *24*, 3162–3172.
24. Tang, X., Cai, L., Meng, Y., Xu, J., Lu, C., and Yang, J. (2021). Indicator regularized non-negative matrix factorization method-based drug repurposing for COVID-19. Front. Immunol. *11*, 3824.
25. Dowden, H., and Munro, J. (2019). Trends in clinical success rates and therapeutic focus. Nat. Rev. Drug Discov. *18*, 495.
26. Li, T., and Ding, C. (2006). The relationships among various nonnegative matrix factorization methods for clustering. In Sixth International Conference on Data Mining (ICDM'06) (IEEE), pp. 362–371.
27. Vanunu, O., Magger, O., Ruppin, E., Shlomi, T., and Sharan, R. (2010). Associating genes and protein complexes with disease via network propagation. Plos Comput. Biol. *6*, e1000641.

28. Mordelet, F., and Vert, J.-P. (2011). Prodige: prioritization of disease genes with multitask machine learning from positive and unlabeled examples. BMC Bioinformatics *12*, 1–15.

29. Guney, E., Menche, J., Vidal, M., and Barábasi, A.-L. (2016). Network-based in silico drug efficacy screening. Nat. Commun. *7*, 10331. https://doi.org/10.1038/ncomms10331. https://www.nature.com/articles/ncomms10331.

30. Cheng, F., Desai, R.J., Handy, D.E., Wang, R., Schneeweiss, S., Barabási, A.-L., and Loscalzo, J. (2018). Network-based approach to prediction and population-based validation of in silico drug repurposing. Nat. Commun. *9*, 1–12. https://doi.org/10.1038/s41467-018-05116-5. https://www.nature.com/articles/s41467-018-05116-5.

31. Yıldırım, M.A., Goh, K.-I., Cusick, M.E., Barabási, A.-L., and Vidal, M. (2007). Drug-target network. Nat. Biotechnol. *25*, 1119–1126.

32. Hopkins, A.L. (2007). Network pharmacology. Nat. Biotechnol. *25*, 1110–1111.

33. Zhou, Y., Hou, Y., Shen, J., Huang, Y., Martin, W., and Cheng, F. (2020). Network-based drug repurposing for novel coronavirus 2019-nCoV/SARS-CoV-2. Cell Discov. *6*, 1–18.

34. Ji, X., and Li, Z. (2020). Medicinal chemistry strategies toward host targeting antiviral agents. Med. Res. Rev. *40*, 1519–1557. https://doi.org/10.1002/med.21664. https://onlinelibrary.wiley.com/doi/abs/10.1002/med.21664.

35. Cao, M., Zhang, H., Park, J., Daniels, N.M., Crovella, M.E., Cowen, L.J., and Hescott, B. (2013). Going the distance for protein function prediction: a new distance metric for protein interaction networks. PLoS One *8*, e76339. https://doi.org/10.1371/journal.pone.0076339. https://journals.plos.org/plosone/article?id=10.1371/journal.pone.0076339.

36. Smola, A.J., and Kondor, R. (2003). Kernels and regularization on graphs. In Learning Theory and Kernel Machines, Lecture Notes in Computer Science, B. Schölkopf and M.K. Warmuth, eds. (Springer), pp. 144–158. https://doi.org/10.1007/978-3-540-45167-9_12.

37. Vishwanathan, S.V.N., Schraudolph, N.N., Kondor, R., and Borgwardt, K.M. (2010). Graph kernels. J. Mach. Learn. Res. *11*, 1201–1242. http://jmlr.org/papers/v11/vishwanathan10a.html.

38. Re, M., and Valentini, G. (2012). Cancer module genes ranking using kernelized score functions. BMC Bioinformatics *13*, S3. https://doi.org/10.1186/1471-2105-13-S14-S3.

39. Re, M., Mesiti, M., and Valentini, G. (2012). A fast ranking algorithm for predicting gene functions in biomolecular networks. IEEE/ACM Trans. Comput. Biol. Bioinform. *9*, 1812–1818. https://doi.org/10.1109/TCBB.2012.114.

40. Yan, R., Zhang, Y., Li, Y., Xia, L., Guo, Y., and Zhou, Q. (2020). Structural basis for the recognition of SARS-CoV-2 by full-length human ACE2. Science *367*, 1444–1448.

41. Blanco-Melo, D., Nilsson-Payant, B.E., Liu, W.-C., Uhl, S., Hoagland, D., Møller, R., Jordan, T.X., Oishi, K., Panis, M., Sachs, D., et al. (2020). Imbalanced host response to SARS-CoV-2 drives development of COVID-19. Cell *181*, 1036–1045.e9. https://doi.org/10.1016/j.cell.2020.04.026. https://www.cell.com/cell/abstract/S0092-8674(20)30489-X.

42. Sirota, M., Dudley, J.T., Kim, J., Chiang, A.P., Morgan, A.A., Sweet-Cordero, A., Sage, J., and Butte, A.J. (2011). Discovery and preclinical validation of drug indications using compendia of public gene expression data. Sci. Transl Med. *3*, 96ra77. https://doi.org/10.1126/scitranslmed.3001318.

43. Food and Drug Administration (2020). Clinical Research. https://www.fda.gov/patients/drug-development-process/step-3-clinical-research#Clinical_Research_Phase_Studies.

44. Food and Drug Administration (2020). Drug Development Process. https://www.fda.gov/patients/learn-about-drug-and-device-approvals/drug-development-process.

45. Li, Z., and Yang, L. (2020). Underlying mechanisms and candidate drugs for COVID-19 based on the Connectivity Map database. Front. Genet. *11*, 1168. https://doi.org/10.3389/fgene.2020.558557. https://www.frontiersin.org/article/10.3389/fgene.2020.558557.

46. Sendama, W. (2020). L1000 Connectivity Map interrogation identifies candidate drugs for repurposing as SARS-CoV-2 antiviral therapies. Comput. Struct. Biotechnol. J. *18*, 3947–3949. https://doi.org/10.1016/j.csbj.2020.11.054. https://www.ncbi.nlm.nih.gov/pmc/articles/PMC7719280/.

47. Wishart, D.S., Feunang, Y.D., Guo, A.C., Lo, E.J., Marcu, A., Grant, J.R., Sajed, T., Johnson, D., Li, C., Sayeeda, Z., et al. (2018). DrugBank 5.0: a major update to the DrugBank database for 2018. Nucleic Acids Res. *46*, D1074–D1082. https://doi.org/10.1093/nar/gkx1037.

48. Zhou, Y., Wang, F., Tang, J., Nussinov, R., and Cheng, F. (2020). Artificial intelligence in COVID-19 drug repurposing. Lancet Digit. Health *2* (12), e667–e676. https://doi.org/10.1016/S2589-7500(20)30192-8. https://www.thelancet.com/journals/landig/article/PIIS2589-7500(20)30192-8/abstract.

49. Riva, L., Yuan, S., Yin, X., Martin-Sancho, L., Matsunaga, N., Pache, L., Burgstaller-Muehlbacher, S., De Jesus, P.D., Teriete, P., Hull, M.V., et al. (2020). Discovery of SARS-CoV-2 antiviral drugs through large-scale compound repurposing. Nature *586*, 1–11. https://doi.org/10.1038/s41586-020-2577-1. https://www.nature.com/articles/s41586-020-2577-1.

50. Luck, K., Kim, D.-K., Lambourne, L., Spirohn, K., Begg, B.E., Bian, W., Brignall, R., Cafarelli, T., Campos-Laborie, F.J., Charloteaux, B., et al. (2020). A reference map of the human binary protein interactome. Nature *580*, 402–408. https://doi.org/10.1038/s41586-020-2188-x. https://www.nature.com/articles/s41586-020-2188-x.

51. Szklarczyk, D., Gable, A.L., Lyon, D., Junge, A., Wyder, S., Huerta-Cepas, J., Simonovic, M., Doncheva, N.T., Morris, J.H., Bork, P., et al. (2019). STRING v11: protein–protein association networks with increased coverage, supporting functional discovery in genome-wide experimental datasets. Nucleic Acids Res. *47*, D607–D613. https://doi.org/10.1093/nar/gky1131. https://academic.oup.com/nar/article/47/D1/D607/5198476.

52. Nepusz, T., Yu, H., and Paccanaro, A. (2012). Detecting overlapping protein complexes in protein-protein interaction networks. Nat. Methods *9*, 471–472. https://doi.org/10.1038/nmeth.1938. https://www.nature.com/articles/nmeth.1938.

53. Kuleshov, M.V., Jones, M.R., Rouillard, A.D., Fernandez, N.F., Duan, Q., Wang, Z., Koplev, S., Jenkins, S.L., Jagodnik, K.M., Lachmann, A., et al. (2016). Enrichr: a comprehensive gene set enrichment analysis web server 2016 update. Nucleic Acids Res. *44*, W90–W97. https://doi.org/10.1093/nar/gkw377. https://academic.oup.com/nar/article/44/W1/W90/2499357.

54. Sadegh, S., Matschinske, J., Blumenthal, D.B., Galindez, G., Kacprowski, T., List, M., Nasirigerdeh, R., Oubounyt, M., Pichlmair, A., Rose, T.D., et al. (2020). Exploring the SARS-CoV-2 virus-host-drug interactome for drug repurposing. Nat. Commun. *11*, 3518. https://doi.org/10.1038/s41467-020-17189-2. https://www.nature.com/articles/s41467-020-17189-2.

55. Galeano, D., Li, S., Gerstein, M., and Paccanaro, A. (2020). Predicting the frequency of drug side effects. Nat Commun *11*, 4575. https://doi.org/10.1038/s41467-020-18305-y.

56. Hassanipour, S., Arab-Zozani, M., Amani, B., Heidarzad, F., Fathalipour, M., and Martinez-de Hoyo, R. (2021). The efficacy and safety of Favipiravir in treatment of COVID-19: a systematic review and meta-analysis of clinical trials. Sci. Rep. *11*, 1–11.

57. Consortium, W.S.T. (2021). Repurposed antiviral drugs for Covid-19—interim WHO solidarity trial results. New Engl. J. Med. *384*, 497–511.

58. Kalil, A.C. (2020). Treating COVID-19—off-label drug use, compassionate use, and randomized clinical trials during pandemics. JAMA *323*, 1897–1898.

59. Grein, J., Ohmagari, N., Shin, D., Diaz, G., Asperges, E., Castagna, A., Feldt, T., Green, G., Green, M.L., Lescure, F.-X., et al. (2020). Compassionate use of remdesivir for patients with severe COVID-19. New Engl. J. Med. *382*, 2327–2336.

60. Kost-Alimova, M., Sidhom, E.-H., Satyam, A., Chamberlain, B.T., Dvela-Levitt, M., Melanson, M., Alper, S.L., Santos, J., Gutierrez, J., Subramanian, A., et al. (2020). A high-content screen for mucin-1-reducing compounds identifies fostamatinib as a candidate for rapid repurposing

CellPress
OPEN ACCESS

for acute lung injury. Cell Rep. Med. *1*, 100137. https://doi.org/10.1016/j.xcrm.2020.100137. https://www.sciencedirect.com/science/article/pii/S2666379120301816.

61. Radulesco, T., Lechien, J.R., Saussez, S., Hopkins, C., and Michel, J. (2021). Safety and impact of nasal lavages during viral infections such as SARS-CoV-2. Ear Nose Throat J. *100*, 188S–191S. https://doi.org/10.1177/0145561320950491.
62. Wang, B., Kovalchuk, A., Li, D., Rodriguez-Juarez, R., Ilnytskyy, Y., Kovalchuk, I., and Kovalchuk, O. (2020). In search of preventive strategies: novel high-CBD *Cannabis sativa* extracts modulate ACE2 expression in COVID-19 gateway tissues. Aging *12*, 22425–22444. https://doi.org/10.18632/aging.202225.
63. Suba, Z. (2020). Prevention and therapy of COVID-19 via exogenous estrogen treatment for both male and female patients: prevention and therapy of COVID-19. J. Pharm. Pharm. Sci. *23*, 75–85. https://doi.org/10.18433/jpps31069. https://journals.library.ualberta.ca/jpps/index.php/JPPS/article/view/31069.
64. Melms, J.C., Biermann, J., Huang, H., Wang, Y., Nair, A., Tagore, S., Katsyv, I., Rendeiro, A.F., Amin, A.D., Schapiro, D., et al. (2021). A molecular single-cell lung atlas of lethal COVID-19. Nature *595*, 114–119. https://doi.org/10.1038/s41586-021-03569-1. https://www.nature.com/articles/s41586-021-03569-1.
65. Delorey, T.M., Ziegler, C.G.K., Heimberg, G., Normand, R., Yang, Y., Segerstolpe, A., Abbondanza, D., Fleming, S.J., Subramanian, A., Montoro, D.T., et al. (2021). COVID-19 tissue atlases reveal SARS-CoV-2 pathology and cellular targets. Nature *595*, 107–113. https://doi.org/10.1038/s41586-021-03570-8. https://www.nature.com/articles/s41586-021-03570-8.
66. Zhou, P., Yang, X.-L., Wang, X.-G., Hu, B., Zhang, L., Zhang, W., Si, H.-R., Zhu, Y., Li, B., Huang, C.-L., et al. (2020). A pneumonia outbreak associated with a new coronavirus of probable bat origin. Nature *579*, 270–273. https://doi.org/10.1038/s41586-020-2012-7. https://www.nature.com/articles/s41586-020-2012-7.
67. Hoffmann, M., Kleine-Weber, H., Schroeder, S., Krüger, N., Herrler, T., Erichsen, S., Schiergens, T.S., Herrler, G., Wu, N.-H., Nitsche, A., et al. (2020). SARS-CoV-2 cell entry depends on ACE2 and TMPRSS2 and is blocked by a clinically proven protease inhibitor. Cell *181*, 271–280.e8. https://doi.org/10.1016/j.cell.2020.02.052. https://www.cell.com/cell/abstract/S0092-8674(20)30229-4.
68. Zhao, M.-M., Yang, W.-L., Yang, F.-Y., Zhang, L., Huang, W.-J., Hou, W., Fan, C.-F., Jin, R.-H., Feng, Y.-M., Wang, Y.-C., and Yang, J.-K. (2021). Cathepsin L plays a key role in SARS-CoV-2 infection in humans and humanized mice and is a promising target for new drug development. Signal Transduct. Targeted Ther. *6*, 1–12. https://doi.org/10.1038/s41392-021-00558-8. https://www.nature.com/articles/s41392-021-00558-8.
69. Lieberman, N.A.P., Peddu, V., Xie, H., Shrestha, L., Huang, M.-L., Mears, M.C., Cajimat, M.N., Bente, D.A., Shi, P.-Y., Bovier, F., et al. (2020). In vivo antiviral host transcriptional response to SARS-CoV-2 by viral load, sex, and age. PLoS Biol. *18*, 1–17. https://doi.org/10.1371/journal.pbio.3000849.
70. Edgar, R., Domrachev, M., and Lash, A.E. (2002). Gene Expression Omnibus: NCBI gene expression and hybridization array data repository. Nucleic Acids Res. *30*, 207–210. https://doi.org/10.1093/nar/30.1.207.
71. Barrett, T., Wilhite, S.E., Ledoux, P., Evangelista, C., Kim, I.F., Tomashevsky, M., Marshall, K.A., Phillippy, K.H., Sherman, P.M., Holko, M., et al. (2013). NCBI GEO: archive for functional genomics data sets—update. Nucleic Acids Res. *41*, D991–D995. https://doi.org/10.1093/nar/gks1193.
72. Robinson, M.D., McCarthy, D.J., and Smyth, G.K. (2010). edgeR: a Bioconductor package for differential expression analysis of digital gene expression data. Bioinformatics *26*, 139–140. https://doi.org/10.1093/bioinformatics/btp616. https://www.ncbi.nlm.nih.gov/pmc/articles/PMC2796818/.
73. U. N. L. of Medicine (2020). Listed Clinical Studies Related to the Coronavirus Disease (Covid-19). https://clinicaltrials.gov/ct2/results?cond=COVID-19.
74. Ghandikota, S., Sharma, M., and Jegga, A.G. (2021). Secondary analysis of transcriptomes of SARS-CoV-2 infection models to characterize COVID-19. Patterns *2*, 100247. https://doi.org/10.1016/j.patter.2021.100247. https://www.sciencedirect.com/science/article/pii/S2666389921000672.
75. Berry, M.W., Browne, M., Langville, A.N., Pauca, V.P., and Plemmons, R.J. (2007). Algorithms and applications for approximate nonnegative matrix factorization. Comput. Stat. Data Anal. *52*, 155–173.
76. Zhou, D., Bousquet, O., Lal, T.N., Weston, J., and Schölkopf, B. (2004). Learning with Local and Global Consistency (Max Planck Institute for Biological Cybernetics), p. 8.
77. Kondor, R., and Vert, J.-P. (2004). Diffusion kernels. In Kernel Methods in Computational Biology, B. Schölkopf, K. Tsuda, and J.-P. Vert, eds. (The MIT Press). https://doi.org/10.7551/mitpress/4057.003.0011 https://direct.mit.edu/books/book/3898/chapter/163650/di-usion-kernels.
78. Picart-Armada, S., Thompson, W.K., Buil, A., and Perera-Lluna, A. (2018). diffuStats: an R package to compute diffusion-based scores on biological networks. Bioinformatics *34*, 533–534. https://doi.org/10.1093/bioinformatics/btx632.

Patterns, Volume *3*

Supplemental information

Machine learning and network medicine approaches for drug repositioning for COVID-19

Suzana de Siqueira Santos, Mateo Torres, Diego Galeano, María del Mar Sánchez, Luca Cernuzzi, and Alberto Paccanaro

1 Note S1. On the differences with other NMF-based drug repositioning methods

Drug repositioning hypotheses can be formulated from different types of biological data, including chemical, molecular[1], cellular[2] and clinical[3].

In computational pharmacology, the drug repositioning problem is often framed in terms of a incomplete association matrix Y of $n \times m$, where the goal is to predict missing associations in Y. In our case, Y contains relationships between n drugs and m viruses, where $y_{ij} = 1$ if drug i is associated to virus j, or $y_{ij} = 0$ otherwise.

Several computational methods have proposed new models based on Non-negative matrix factorisation (NMF)[4,5] for distinct settings of the drug repositioning problem. Here we review some of these methods and highlight key differences with our matrix decomposition model. We start with the standard or vanilla NMF of Lee and Seung[4,5].

Vanilla NMF There are two main differences between our model and the vanilla NMF model of Lee and Seung[4]: the cost function and the learning algorithm. To explain these difference in more detail, let us denote a generic data matrix of $n \times m$ as Y. The goal of NMF is to obtain a low-rank decomposition of Y by minimising the following cost function:

$$\begin{aligned} &\min_{P,Q} \mathscr{J}(P,Q) = \frac{1}{2}\|Y - PQ\|_F^2 = \frac{1}{2}\sum_{ij}(Y_{ij} - (PQ)_{ij})^2 \\ &\text{subject to } P,Q \geq 0 \end{aligned} \tag{1}$$

where P is a matrix of $n \times k$ and Q is a matrix of $k \times m$, k is the rank of the approximation and $\|.\|$ is the Frobenius norm of a matrix.

Notice that Equation (1) is non-convex in P and Q. Lee and Seung[4,5] derived multiplicative learning rules that can find a local minimum solution of Equation (1) under non-negative constraints. The multiplicative algorithm consist on iteratively applying the following update rule assuming that P and Q are initialised from random values in $t = 0, t \in \{1, 2, \ldots \text{maxiter}\}$ (e.g. uniform distribution in the range $[0, 1]$):

$$\begin{aligned} P &\longleftarrow P\frac{YQ^T}{PQQ^T} \\ Q &\longleftarrow Q\frac{P^TY}{P^TPQ} \end{aligned} \tag{2}$$

The multiplicative learning rules in Equation (2) have the advantage that they do not require setting a learning rate or applying projection functions to guarantee the non-negative constraints.

Notice that when minimising Equation (1), all the entries in Y are considered equally. This makes sense when entries in Y have the same meaning or importance. For instance, if it contains pixels of images[4]. However, in our problem, the data matrix Y contains drug-virus associations that are at different stages of drug development. Therefore, the strength of each known drug-virus association in Y varies according to the evidence of efficacy. In our model, we account for the varying types of evidence of efficacy of drug-virus associations by weighting them differently in our cost function. The weighting is simple: groups of drug-virus associations are weighted according to their probability of success (motivated by the study of Dowden et al.[6]). In this way, those groups in early stages of development have less importance than those in the later stages of development during the learning. Furthermore, since our cost function is different from the vanilla NMF, our learning rule to minimise it is also different. We showcase our learning algorithm in the Methods section of the main manuscript.

A recent paper by Sosnina et al. has shown that vanilla NMF[5] provide a better performance at predicting missing interactions than other algorithms used for predicting missing data in recommender systems. In their study, Sosnina et al.[7] used a dataset consisting of cell-assay derived small molecule- antiviral activity interactions.

NMF with L2 regularization Bakal et al.[8] recently proposed the use of with L2 regularisation model to predict missing drug-disease associations in the repoDB database[9], which contains approved and failed drug-disease associations for distinct categories of diseases (including genetic, neurologicals, syndromes, neoplastic, etc.). Following our previous notation, the cost function of NMF with L2 regularization is as follows:

$$\begin{aligned} &\min_{P,Q} \mathscr{J}(P,Q) = \frac{1}{2}\|Y - PQ\|_F^2 + \frac{\beta}{2}(\|P\| + \|Q\|) \\ &\text{subject to } P,Q \geq 0 \end{aligned} \tag{3}$$

That can be minimised with the following multiplicative learning rules:

$$\begin{aligned} P &\longleftarrow P\frac{YQ^T}{PQQ^T+\beta P} \\ Q &\longleftarrow Q\frac{P^TY}{P^TPQ+\beta Q} \end{aligned} \tag{4}$$

The main difference between vanilla NMF and NMF with L2 reg. is that an L2 norm is added to the loss function to improve generalization and reduce model complexity.

TriFactor NMF Ceddia et al.[10] recently proposed using non-negative matrix tri-factorization (TriFactor NMF) model for predicting missing drug-target interactions, missing drug-therapeutic categories, and missing drug-disease associations using diverse datasets. TriFactor NMF seeks to decompose the matrix Y as follows $Y \simeq FSG^T$, by minimising the following loss function:

$$\begin{aligned} &\min_{F,S,G} \mathscr{L}(F,S,G) = \frac{1}{2}\left\|Y - FSG^T\right\|_F^2 \\ &\text{subject to } F,S,G \geq 0 \end{aligned} \tag{5}$$

That can be minimised with the following multiplicative learning rules[11,12]:

$$\begin{aligned} G &\longleftarrow G\sqrt{\frac{Y^TFS}{GG^TY^TFS}} \\ F &\longleftarrow F\sqrt{\frac{YGS^T}{FF^TYGS^T}} \\ S &\longleftarrow S\sqrt{\frac{F^TYG}{F^TFSG^TG}} \end{aligned} \tag{6}$$

IRNMF Tang et al.[13] addresses the problem of predicting missing drug-virus associations with potential applications on drug repositioning for COVID-19. The model they proposed is a variation of NMF called Indicator Regularised NMF (IRNMF).

IRNMF is different from our model in the type of information it uses: IRNMF integrates chemical similarities between drugs and sequence similarities between viruses, while our model does not use this information; our model uses drug developmental stages, which are not used in the IRNMF model.

Comparison with NMF and its variations Although the specific drug repositioning problems addressed by Sosnina et al.[7], Bakal et al.[8] and Ceddia et al.[10] are different from ours (as we are predicting drug-virus associations), we can still compare their specific variant of NMF against our model on our drug-virus association data.

To this end, we implemented the NMF with L2 regularization and the triFactor NMF model. We then used our training and validation sets to set the hyperparameters of each model: (i) For the NMF with L2 model, we found optimal performance in the validation set with: number of latent factors = 5, and the L2 regularization penalty = 0.1; (ii) For the triFactor NMF, we found optimal performance in the validation set with: number of factors = 15.

Using the Leave-One-Out Cross-Validation (LOOCV) procedure described in the paper, we assessed the performance of each of these methods at retrieving missing phaseIV/approved drug-virus associations. The Figure 3 in the main manuscript shows that our matrix decomposition model significantly outperforms the two variants of NMF. We also observed that NMF with L2 regularization and triFactor NMF perform slightly better than vanilla NMF.

In order to run IRNMF on our dataset, we followed the procedure in Tang et al.[13] to obtain chemical similarities for our set of drugs and sequence similarities for our set of viruses.

To obtain chemical similarities between our set of 126 drugs, we mapped drug names to DrugBank 5.1 (`https://go.drugbank.com/`) identifiers. 109 out of the 126 drugs could be mapped to DrugBank. We then obtained the chemical SMILES representation for each drug from DrugBank. Finally, using the open source RDKit Chemoinformatics tool (`https://www.rdkit.org/`), we obtained the MACCS chemical fingerprint and computed the 2D Tanimoto chemical similarities.

To obtain virus similarities for our set of 80 viruses, we downloaded the most complete reference genomes of each virus from NCBI (`https://www.ncbi.nlm.nih.gov/genome/`). Following Tang et al.[13], we used the MAFFT algorithm

to get the distance matrices. This could only be applied to the viruses with a single DNA segment, which resulted in 64 out of 80 viruses in our dataset.

In this way, we obtained the three matrices required to run by IRNMF: a binary drug-virus matrix containing 654 associations between 109 drugs and 64 viruses; a chemical similarity matrix of 109x109; a sequence similarity matrix of 64x64. In this drug-virus matrix, 66 associations are in phases I, II or III of development, and 49 associations are in phase IV/approved stages.

Having created the datasets, we performed the evaluation of the two models. First, we used the 66 phase I-III associations to set the model parameters both for our model and for IRNMF (optimal performance for IRNMF was obtained with: number latent factors = 10; L2 regularization penalty = 0.1; side information regularization = 10). Then, we then repeated our leave-one-out evaluation on the 49 associations that are in phase IV/approved stages: For each of these associations, we removed it from our dataset; trained the model with the remaining data matrix; and then ranked drugs based on the predicted scores. To run IRNMF in our dataset, we used the code provided by the authors in `https://github.com/dukebai/IRNMF`.

Figure S1 below shows that our matrix decomposition model significantly outperforms IRNMF at recovering missing phase IV/approved drug-virus associations at different number of top predictions retrieved. We observed that our method performs from 26.53% to 53% better than IRNMF at recovering phase IV/approved missing drug-virus associations.

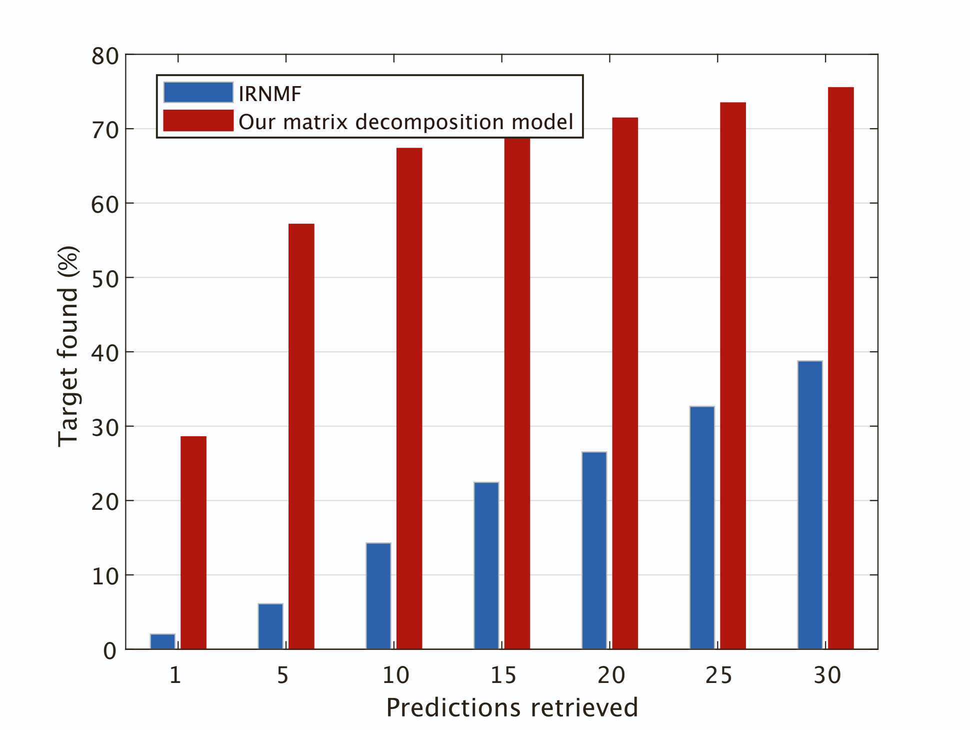

Figure S1. Performance comparison between IRNMF[13] and our matrix decomposition model at predicting missing phase IV/approved drug-virus associations in the Andersen et al.[14] drug-virus dataset.

Comparison in the SARS-CoV-2 specific evaluation dataset . We also seek to compare the performance of the methods on the specific evaluation datasets that we collected for SARS-CoV-2. In the evaluation that follows, we used the *in vitro* and *clinical trials* datasets, but not the CMAP dataset, because only one drug with CMAP activity was found in the Andersen et al.[14] dataset. In the *in vitro* dataset, we found 10 BSA drugs with *in vitro* efficacy against SARS-CoV-2; 4 of which were already in the Andersen et al.[14] dataset. In the clinical trials dataset, we found 28 BSA drugs in clinical trials for COVID-19; 6 of which were already in the Andersen et al.[14] dataset.

We performed a leave-one-out evaluation experiment in the *in vitro* dataset, where one of the ten drugs was left out and the other nine were also used for training together with the ten associations from the Andersen et al.[14] dataset. In our evaluations, we only ranked drugs without known associations in training. Therefore, the final set of labels consisted on 10 positive labels and 110 negative labels. In the clinical trials dataset, we could not repeat the leave-one-out evaluation experiment because we do not know the specific phase of clinical trial. In this case, we simply run the methods using all the data available in the Andersen et al.[14] dataset combined with the *in vitro* evaluation dataset. The performance was also calculated only on drugs without associations in training, i.e., 20 positive labels and 90 negative labels. Finally, all the methods were run using their optimal hyperparameters.

Figure S2 below shows the recall and precision of the methods at different values of predictions retrieved. In the clinical trials evaluation dataset, our matrix decomposition model greatly outperforms the competitors. It can recover 50% of BSA drugs already in clinical trials for COVID-19 in the top-20 drugs retrieved. In the *in vitro* evaluation dataset, we noticed that the best performing method changes in different tops. For instance, our matrix decomposition model performs on part of vanilla NMF in the top-10. We notice here that our method was not tuned to fit *in vitro* data in the Andersen et al. dataset , but rather phase IV/approved stages (i.e., clinical success of drugs). This might explain why our method performs much better than other competitors on the clinical trials dataset but not so well on the *in vitro* data. In fact, predicted scores by our matrix decomposition model on the *in vitro* developmental stages are very low (see Figure S4). Another explanation for the variation of the performance of the methods in the *in vitro* data could be due to the fewer number of positive labels available.

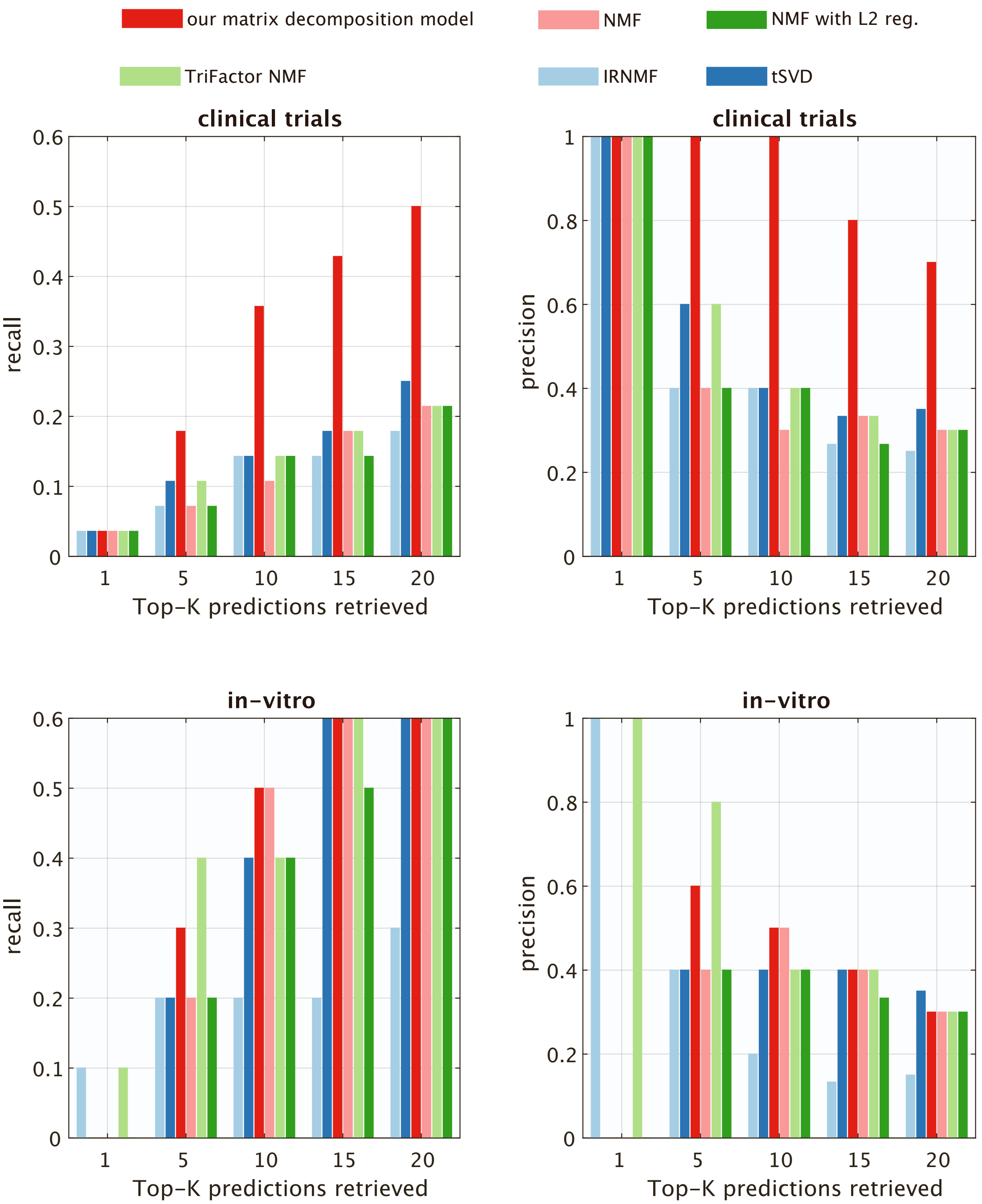

Figure S2. Precision and recall at different number of BSA drugs retrieved by different matrix factorization drug repositioning methods in two evaluation datasets specific to SARS-CoV-2. **clinical trials:** 20 BSA undergoing clinical trials for COVID-19 vs 90 remaining drugs. ***in vitro*:** 10 BSA with activity *in vitro* against SARS-CoV-2 vs 110 remaining drugs.

2 Note S2. On whether the use of developmental information can bias the predictions

One question that may arise when using our matrix decomposition model is whether the grouping by developmental stages in our cost function introduces a bias in the predictions. In other words, whether our model is only predicting drugs that are in the late stages of development, rather than the most effective ones. We will show that, in the intended use of our matrix decomposition model, when predicting missing drug-virus associations, the bias of developmental information cannot affect the predictions because developmental information is not available for them.

To understand how the grouping influences the predictions made by our matrix decomposition model, let us consider that our model takes as input an incomplete sparse drug-virus matrix with only 8.43% non-zero entries, and outputs predicted scores for all the entries in the matrix (see Figure S3 below). These predicted scores can be categorised into two classes:

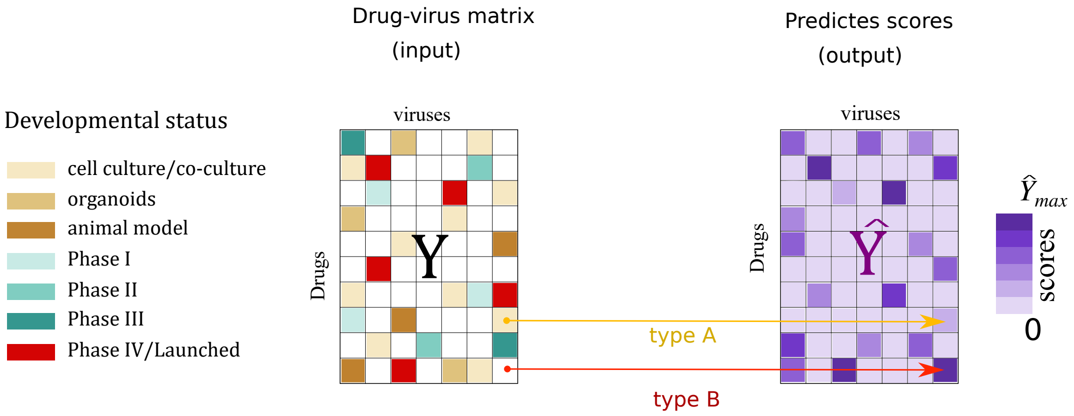

Figure S3. The predictions made by our matrix decomposition model can be categorised based on whether the predicted scores are used to rank non-approved drug-virus associations available for training (type A, an example shown with an arrow in yellow) or missing drug-virus associations, without *any* evidence of efficacy (type B, an example shown with an arrow in red). Our model is intended for type B predictions.

- **Type A predictions**. These corresponds to entries y_{ij} in the original matrix Y that are non-zero. Here we are predicting whether drug i, which is already in some stage of development with respect to virus j, will eventually reach drug approval for virus j.
- **Type B predictions**. These corresponds to entries y_{ij} in the original matrix Y that contains zeros. Here we are predicting whether drug i, which is not currently being developed for virus j, will reach drug approval for virus j.

In our manuscript, we focus on validating predictions of type B, because it corresponds to the interesting case where the scores predicted by our model can be used to rank drug-virus associations that are not yet in drug development, and for which novel drug repositioning hypotheses can be made. In the manuscript, we have showcased the utility of our approach in this scenario by using a leave-one-out cross-validation (LOOCV) procedure. In this procedure, only one drug-virus association (in phase IV or approved) was removed from the drug-virus matrix Y (by setting the corresponding entry to zero). We then trained the model, and scores were predicted for all the drugs. We then ranked drugs that had no known association with that virus and checked the percentage of cases in which the effective drug for the virus was found among the top-K predictions. Fig. 3 in the main manuscript shows that our matrix decomposition model can predict 60% the correct drug among the top-10 predictions retrieved.

Predictions of type A typically require a different setting where the goal is to predict clinical success of candidate compounds in early stages of drug development given molecular, chemical, cellular and clinical features[15]. Therefore, using our model for type A predictions is likely to generate predictions that are biased towards the known developmental stages. One way to verify this is to look at the scores that we obtain for drug-virus associations for the 850 known drug-virus associations that were used during training. These are shown in the Figure S4 below, which presents the scores grouped based on their known developmental status. We can see that, on average, the higher the developmental stage, the higher the score output by our model.

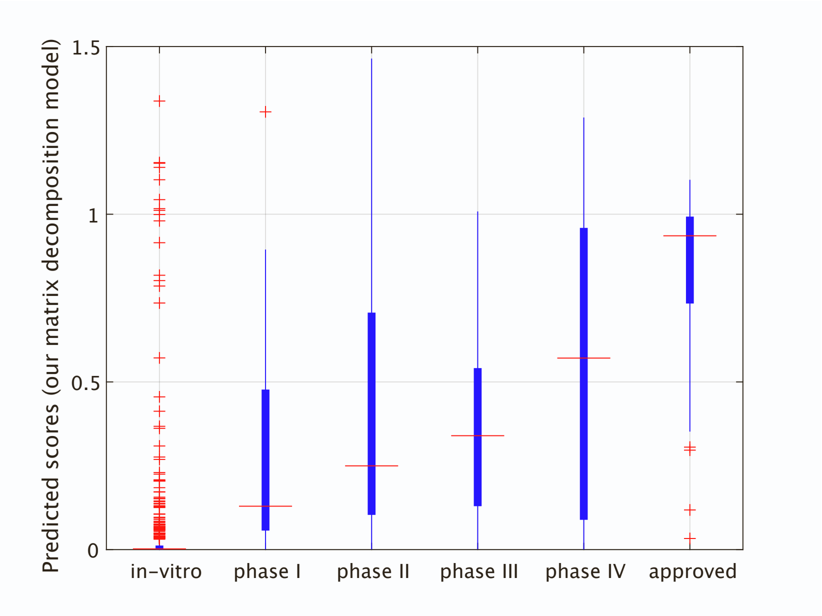

Figure S4. Predicted scores by our matrix decomposition model for drug-virus associations used for training the model. 850 drug-virus associations were grouped based on their known developmental status.

We analysed the top-20 predictions obtained from our system, separating them into Type A and Type B predictions. The top type B predictions, corresponding to predicted Broad-Spectrum Antiviral drugs not known to be under development against SARS-CoV-2 according to the Andersen et al. dataset, are listed in Table S1.

Rank	Predicted score	Drug name (ID)	Additional curated evidence for COVID-19
5	0.46	Tenofovir (DB14126)	combinational therapy, currently on ClinicalTrials.gov (NCT04519125)
6	0.43	Lamivudine (DB00709)	combinational therapy, case report in Brasil[16]
8	0.37	Zanamivir (DB00558)	*in silico* molecular docking[17]
10	0.21	Azithromycin (DB00207)	combinational therapy, clinical trials[18], compassionate use[19]
11	0.20	Amiodarone (DB01118)	currently on ClinicalTrials.gov (NCT04351763)
12	0.19	Artesunate (DB09274)	combinational therapy, *in vitro*[20], currently under WHO Solidarity PLUS clinical Trials
14	0.13	Thymalfasin (DB04900)	currently on ClinicalTrials.gov (NCT04487444)
15	0.12	Brincidofovir (DB12151)	NA
16	0.12	Rapamycin (DB00877)	currently on ClinicalTrials.gov (NCT04461340), geroprotective[21]
17	0.11	Nitazoxanide (DB00507)	currently on ClinicalTrials.gov (NCT04463264)
18	0.10	Sunitinib (DB01268)	AI-evidence[22]
19	0.08	Erlotinib (DB00530)	AI-evidence[22]
20	0.07	Valacyclovir (DB00577)	NA
21	0.07	Interleukin-7 (DB11997)	clinicalTrials.gov (NCT04379076), compassionate use[23,24]

Table S1. Predictions of type B obtained with our matrix decomposition model:. Predictions of type B correspond to predicted broad-spectrum antiviral drugs not known to be under development against SARS-CoV-2 according to the Andersen et al. dataset. These predictions had a value of zero in the drug-virus matrix Y used for training. Only predicted drugs within the top-20 predictions are shown.

Interestingly, this set of drugs includes drugs for which we could find in the literature some indication of efficacy, and

for several of them we found that they are already in clinical trials for COVID-19 (third column in Table S1). Note that our 12th top predicted drug is the anti-malarial drug Artesunate, which was selected for the currently ongoing WHO Solidary Plus Clinical Trials[25].

The top predictions of type A, corresponding to predicted Broad-Spectrum Antiviral drugs already known to be under development, but not approved against SARS-CoV-2 according to the Andersen et al. dataset, are shown in Table S2. As expected, for the six drugs known to be under development for SARS-CoV-2, predictions are biased towards drugs that are in later stages of development. The drug with the highest predicted score was Favipiravir, already known to be in phase II of clinical trials for COVID-19 according to the Andersen et al. dataset. Favipiravir is an RNA polymerase inhibitor that was developed in Japan as an antiviral treatment for influenza. In October 2020, already 37 clinical trials were registered in ClinicalTrials.org to assess its efficacy and safety for COVID-19 patients[26].

Rank	Predicted score	Drug name (ID)	Dev. Stage available for training	Additional curated evidence for COVID-19
1	1.03	Favipiravir (DB12466)	phase II	*in vitro*[27], several clinical trials[28,29], compassionate use[19]
2	0.95	Arbidol (DB13609)	phase IV	*in vitro*[30], under clinical trials[31]
3	0.58	Lopinavir (DB01601)	phase IV	combinational therapy, clinical trials in severe COVID-19[32], compassionate use[19], little or no clinical benefit[33]
4	0.58	Ritonavir (DB00503)	phase III	combinational therapy, clinical trials in severe COVID-19[32], compassionate use[19]
7	0.42	Remdesivir (DB14761)	phase III	compassionate use[34], clinical trials[27]
9	0.28	Hydroxychloroquine (DB01611)	phase III	compassionate use[19], Emergency Use Authorisation (EUA) by FDA[35] (revoked on June, 2020), little or no clinical benefit[33].
13	0.17	Chloroquine (DB00608)	cell culture/co-culture	Emergency Use Authorisation (EUA) by FDA (revoked on June, 2020), little or no clinical benefit[33,36].

Table S2. Predictions of type A obtained with our matrix decomposition model:. Predictions of type A correspond to predicted Broad-Spectrum Antiviral drugs already known to be under development, but not approved, against SARS-CoV-2 infection. Only predicted drugs within the top-20 predictions are shown.

However, a systematic review of the randomised clinical trials suggest that Favipiravir has shown low efficacy for patients with mild or moderate COVID-19[26]. In fact, other drugs including Remdesivir, Lopinavir and Hydroxychloroquine have shown little to no clinical efficacy in COVID-19 patients according to the WHO Solidarity Trial, conducted in more than 11,000 patients across 405 hospitals in 30 countries[33]. Therefore, the lack of efficacy of BSA drugs that were under development at the time of release of the Andersen et al. dataset suggests that it is important to consider predicted BSA drugs that were not yet under development against SARS-CoV-2 (presented in Table S1).

3 Note S3. How much our model exploits developmental stages information?

An important question is how much of the performance of our model was due to the use of the developmental stages' information in our modelling. To understand this, we designed two different experiments:

- **Randomisation of developmental stages**. We randomised the developmental stages assigned to drug-virus associations in the matrix used for training. This experiment is designed to inform us about whether there is relevant information in the specific subsets of developmental stages used.
- **Removing the information of the developmental stages from our model**. To understand whether the prediction performance of our model depends on assigning different probabilities of success to different groups of entries, we set all the coefficients assigned to the different drug developmental stage groups to 1 (i.e. $\alpha_B = \alpha_C = \alpha_D = \alpha_E = 1$). Only the α_z coefficient of our original model is retained, thus effectively weighing down the information brought in by the zeros (note that our model reduces to the model by Galeano et al.[37]). In this case, the information of the developmental stages is removed altogether from the cost function as all the known drug-virus associations are considered equal during learning.

For both experiments, we followed the leave-one-out cross validation (LOOCV) that we described in the main manuscript. For each of the 71 phase IV/approved associations, we removed it from the drug-virus matrix Y by setting its corresponding entry to zero, and then trained the model with the remaining data. Then, we ranked the drugs with not known association in training based on their predicted scores. The prediction performance was then measured on the ability of the method to predict the correct association among the top-K predictions retrieved.

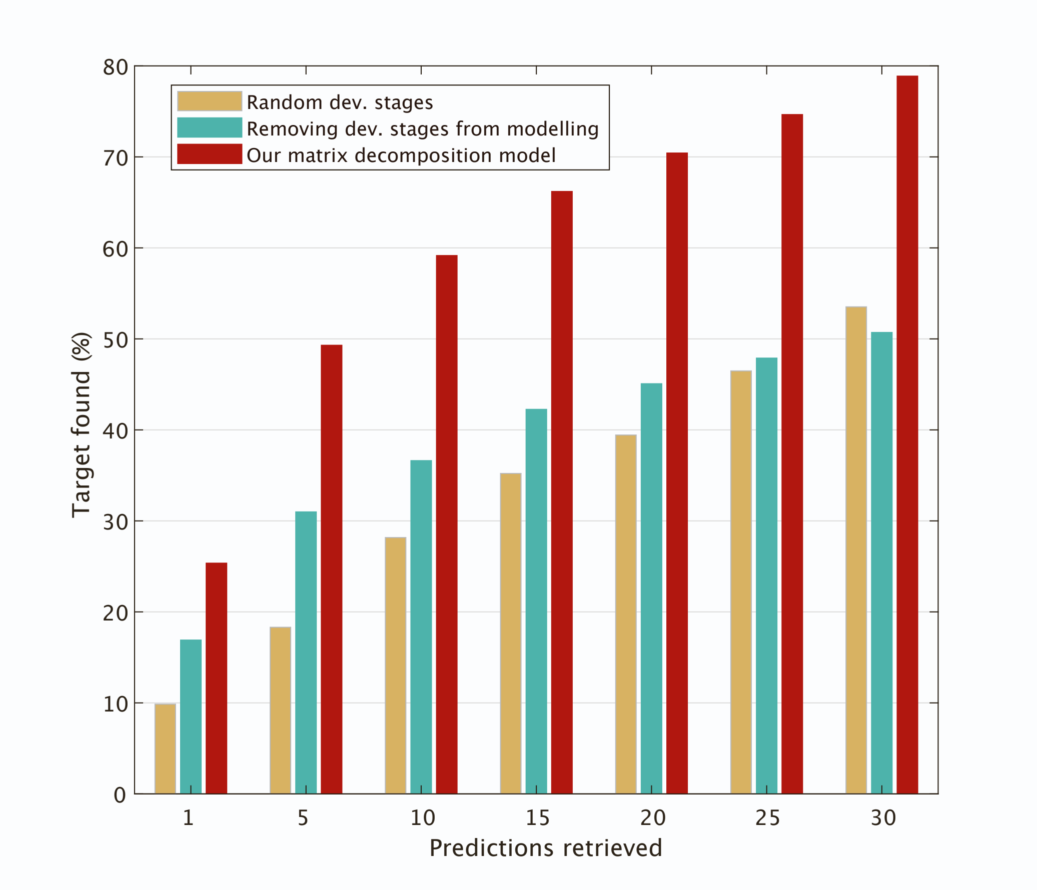

Figure S5. Comparison of the performance of our matrix decomposition model at predicting approved/phase IV Broad Spectrum Antivirals (BSA) for 28 viruses under two control procedures to illustrate the importance of the modelling of the developmental stages. In the first experiment, we randomised the developmental stages of the drug-virus associations used for training (brown bars). In the second experiment, we removed the modelling of the developmental stages from the cost function (green bars).

Figure S5 shows a comparison of the prediction performance of the two competitors against our matrix decomposition model. Our method outperforms significantly both competitors. It predicts 15-31% better than the competitor that randomises the developmental stages (brown bars), and by 8.45-28% better than when the developmental stage information is removed from the cost function (green bars). Our experiments suggest that the integration of the developmental stages in our cost function leads to significant improvements in prediction performance.

4 Note S4. Evaluation datasets for the network medicine approach

Our network medicine approach was thoroughly evaluated and compared using three different PPI networks, as well as several evaluation datasets. In this section, we provide details of all the datasets involved in each setting.

We consider 3 PPI networks, as described in Table S3. Ultimately, the set of drugs that we are able to place in the interactome will vary with the set of proteins included in each interactome. Our main evaluation setting, and the one that involves the largest set of approved drugs, is related to the interactome put together by Gysi et al.[38]. Once the interactome is set, we retrieve all the FDA approved drugs from the DrugBank database[39]. Every approved drug with at least one protein target in the interactome is suitable for our network medicine approach.

PPI	number of proteins	number of links
**Cheng et al.[40]**	15,646	218,092
**HuRI[41]**	16,091	353,435
**Gysi et al.[38]**	18,505	327,924

Table S3. Protein-Protein Interaction networks. Three PPI networks were used throughout this study to evaluate our network medicine approach. Cheng et al.[40] and HuRI[41] were restricted to the reviewed entries in UniProtKB (UniProtKB/Swiss-Prot)[42]. The interactome put together by Gysi et al.[38] uses Entrez identifiers [https://doi.org/10.1093/nar/gks1189].

To evaluate the results, we rely on three sources of evidence:

- ***in vitro* experiments:** The union of *in vitro* screenings from Riva et al.[43] and Gysi et al.[38]. They total 99 drugs in DrugBank that show effects against SARS-CoV-2 in the screenings. 81 such drugs are FDA approved.
- **Clinical trials:** Retrieved from `ClinicalTrials.gov` on December 1st, 2020. They total 244 drugs mapped to DrugBank listed as an intervention on a clinical trial. 186 such drugs are FDA approved.
- **CMAP:** Obtained by querying CMAP[44,45] using differentially expressed genes on COVID-19 patients vs. a control group[46]. They total 30 drugs with a significant τ score (< -90). 23 such drugs are FDA approved.

Dataset	Evidence Type	Number of Drugs	Number of Positives	Number of negatives
**IV-2197**	In Vitro Evidence (Riva et al.[43] & Gysi et al.[38])	2197	81	2116
**CT-2197**	Clinical Trials retrieved on December 1, 2020	2197	170	2027
**CM-2197**	CMap evidence	2197	23	2174
**IV-1853**	In Vitro Evidence (Riva et al.[43] & Gysi et al.[38])	1853	78	1775
**CT-1853**	Clinical Trials retrieved on December 1, 2020	1853	153	1700
**CM-1853**	CMap evidence	1853	21	1832
**E918-IV**	In Vitro Evidence from Gysi et al.[38]	918	77	841
**E918-CT**	Clinical Trials retrieved on April 15, 2020	918	37	881
**E918-CM**	CMap evidence	918	19	899

Table S4. Datasets for the network medicine approach

The final step required to build an evaluation set is to assign labels to the drugs based on the available evidence. To do it, we simply intersect the suitable drugs for a given interactome with the set of drugs from one of the groups described above. The resulting evaluation settings are summarised in Table S4. The main evaluation settings are IV-2197, CT-2197, and CM-2197, which go together with the Gysi et al.[38] interactome. Settings IV-1853, CT-1853, and CM-1853 are the result of building the

set for the Cheng et al.[40] and HuRI[41] interactomes. the last 3 evaluation sets in the table (E918-IV, E918-CT, and E918-CM) are slightly different, as they are used to compare our results to those of Gysi et al.[38]. Some of the 918 drugs included in these settings were not approved by FDA, which frames a fundamentally different problem than the other evaluation sets. Details of our comparison using E918-IV, E918-CT, and E918-CM are available in Note 8.

5 Note S5. Evaluation of the network medicine approach on Gysi et al PPI

We ran network medicine methods on the Gysi et al PPI, and compared the results on three different evaluation sets: IV-2197 (*in vitro*), CT-2197 (clinical trials), and CM-2197 (CMAP).

In Figure S6, we compare prediction scores between drugs with evidence for COVID-19 and the remaining drugs for different types of evidence. We also show the corresponding One-Sided Wilcoxon-Mann-Whitney p-values. Results are shown for our network medicine approach, and two network-based competitors: the DSD method[47], and the Guney distance[48]. The kernel-based methods and DSD[47] have significant p-values in all scenarios, while the Guney distance[48] has non-significant p-value for CMAP. In every case, the kernel-based methods have the smaller p-values.

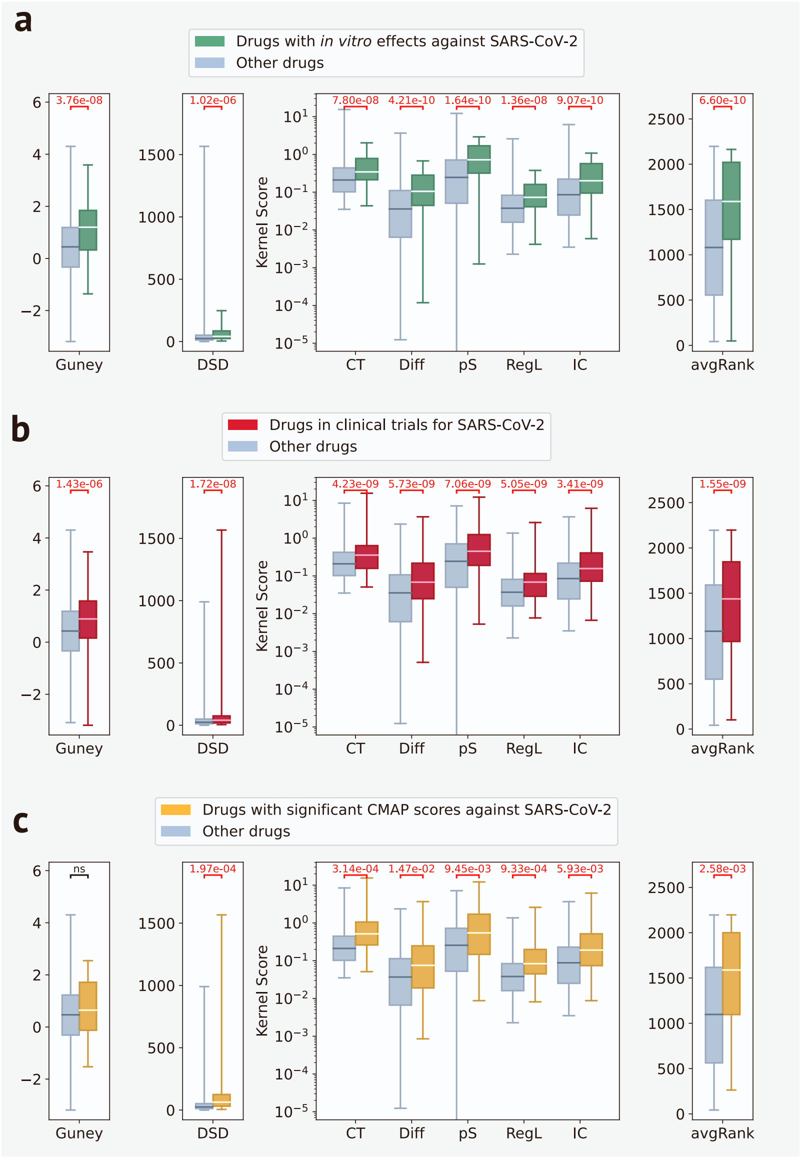

Figure S6. Prediction scores by the network medicine approach on the Gysi et al. PPI. We used boxplots to compare prediction scores between drugs with and without evidence for COVID-19 on three different evaluation sets: *in vitro* evidence (IV-2197), clinical trials evidence (CT-2197), and CMAP evidence (CM-2197). For each comparison, we computed the One-Sided Wilcoxon-Mann-Whitney p-value (one tailed test). Non-significant p-values (below 0.05) are indicated with 'ns'. Significant p-values are shwon in red. We show results obtained by our network medicine approach and competitors. Our approach includes five different kernels on graphs (commute time, diffusion, inverse cosine, p-step kernel, and regularised laplacian), and a combined ranking across the kernels (avgRank). The competitors include DSD[47], and the Guney distance[48].

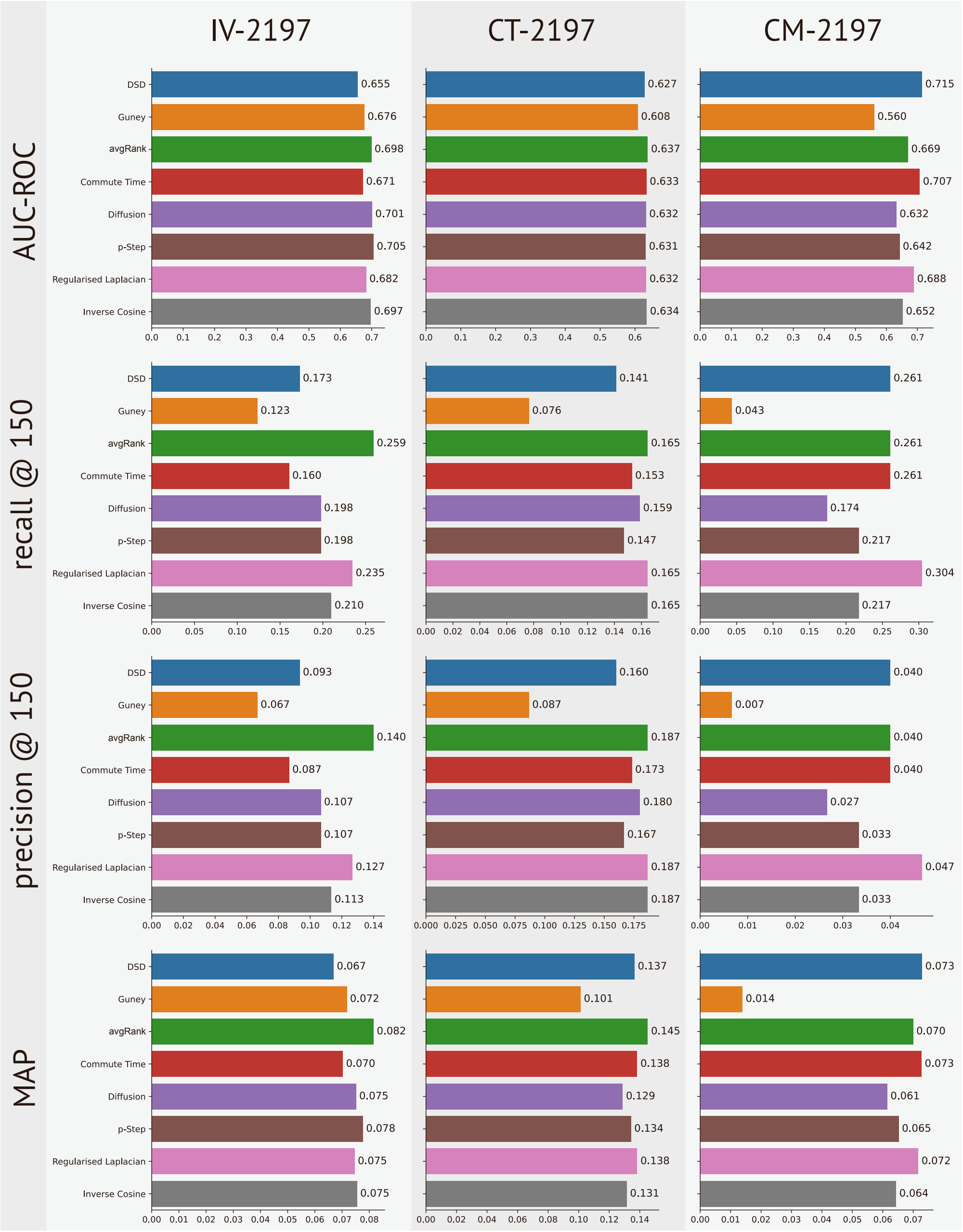

Figure S7. Classical machine learning metrics obtained by the network medicine approach on the Gysi et al. PPI. We computed the area under the ROC curve (AUC-ROC), recall at top 150, precision at top 150, and mean average precision (MAP) for three different evaluation sets. They correspond to *in vitro* evidence (IV-2197), clinical trials evidence (CT-2197), and CMAP evidence (CM-2197). For each type of evidence, we show results obtained by our network medicine approach and competitors. Our approach includes five different kernels on graphs (commute time, diffusion, inverse cosine, p-step kernel, and regularised Laplacian), and a combined ranking across the kernels (avgRank). The competitors include DSD[47], and the Guney distance[48].

Figure S7 shows traditional machine learning metrics for comparing the performance across different network medicine methods. When evaluating on the *in vitro* dataset (IV-2197), we observe that the average ranking aggregation (avgRank) is the best performing method in the recall and precision at top 150, and MAP metrics. The best performance in the AUC-ROC metric is achieved by calculating the score using our network medicine approach with the p-Step kernel.

Evaluating on the clinical trial dataset (CT-2197), we observe that the best performer throughout the four evaluation metrics is the average ranking (avgRank). In the recall and precision at top 150, it is matched only by using our network medicine

approach with the regularised Laplacian or inverse cosine kernels.

Finally, evaluating on CMAP data (CM-2197), we observe that the regularised Laplacian is the best performing method in the recall and precision at top 150, and MAP metrics. The best performance in the AUC-ROC metric is achieved by the DSD method[47] with a very similar performance achieved by calculating the score using our network medicine approach with the commute time kernel.

All in all, the main pattern we observe when comparing the performance using traditional machine learning metrics is that our network medicine approach is very robust in comparison to the competitors. This especially true for the recall and precision metrics which are, arguably, the most interesting metrics to observe when planning drug repurposing experiments.

6 Note S6. Comparison between our network medicine approach and methods by Gysi et al

Gysi et al.[38] tested 918 drugs *in vitro* against SARS-CoV-2. They proposed computational approaches to predict drugs with efficacy against SARS-CoV-2. However, the problem from Gisy et al. is slightly different from ours. Out of the 918 experimentally screened drugs, 833 were approved, with the remaining 85 drugs distributed between investigational, experimental, nutraceutical, and withdrawn. Our problem, however, focuses on ranking approved drugs exclusively. Nevertheless, we thought it was interesting to compare the performance of our network medicine approach with Gysi et al.'s AI based methods. We did this by running our network medicine approach to rank the 918 drugs used in their evaluation. For a fair comparison, we used the same dataset than Gysi et al.[38], as described below.

- PPI with 18,505 proteins and 327,924 interactions.
- 24,648 associations between 6253 and 3910 targets used by Gysi et al.[38]. The dataset contains data from DrugBank (`https://go.drugbank.com/`), and other additional 25 drugs with targets curated by the literature.
- 332 host proteins identified by Gordon et al.[49].
- A set of 918 drugs (E918) that were tested *in vitro* against SARS-CoV-2 on Vero E6 cell lines[38].
- Evaluation set of drugs with *in vitro* evidence, which contains 77 compounds with weak or strong evidence on the *in vitro* experiment (we refer to this evaluation setting as E918-IV, see Table S4).
- Evaluation set of drugs with clinical trials evidence, which contains 37 drugs that are both in E918 set and clinical trials of April 15th 2020 (we refer to this evaluation setting as E918-CT, see Table S4).

In addition, we used drugs predicted by Connectivity Map (CMAP)[44,45] as an additional source of evidence. For running CMAP, we used the consensus dataset by Ghandikota et al.[46] as the COVID-19 signature, with genes that are up- or down-regulated in different *in vitro* and *in vivo* models of SARS-CoV-2 infections. Our query on CMAP resulted in 19 drugs with significant CMAP τ score (< -90) that overlap with the list of 918 drugs tested *in vitro* (we refer to this evaluation setting as E918-CM, see Table S4).

We evaluated the performance of our network medicine methods and competitors on different types of evidence for COVID-19: E918-IV (*in vitro* evidence), E918-CT (clinical trials), and CMAP evidence (E918-CM). Our approach includes five different kernels (commute time, diffusion, inverse cosine, p-Step with $p = 2$, and regularised Laplacian), and an aggregated ranking (avgRank). As competitors. we considered methods used by Gysi et al.[38], which includes AI-based methods (based on Graph Neural Networks - GNN), diffusion-based methods (variations of DSD[47]), proximity-based methods (variations of the Guney's distance[48]), and cRank[REF] (a combination of all the rankings). We then computed the same evaluation metrics as Gysi et al., that is, the AUC-ROC (area under the ROC curve), recall at top 100, and precision at top 100). These results are shown in Figure S8.

We observed that for all evaluation datasets, our approach has an AUC-ROC higher than the diffusion-based methods, and similar or higher than the proximity based methods. We also have a higher AUC-ROC than the AI based methods in all types of evidence except for clinical trials (E918-CT). Our performance is always higher than a random classifier (AUC-ROC > 0.5). In contrast, A4 (AI-based method), and the diffusion based methods (variations of DSD) have an AUC below 0.5 for CMAP evidence, and D3 and D5 (variations of DSD) have an AUC-ROC below 0.5 for *in vitro* evidence.

We also observed that our matrix decomposition model predicts earlier drugs with *in vitro* or CMAP evidence. As shown in Figure S8, we have the largest recall and precision at top 100 for these sets of drugs. The only scenario in which we do not have the higher AUC-ROC nor the higher recall is for predicting drugs in clinical trials on April 15th 2020. In this case, the AI-based methods (GNN) have the best performance. Nevertheless, in this scenario our approach has the highest recall and precision compared to all proximity and diffusion based methods.

Finally, in Figures S9, S10, and S11, we compare prediction scores of drugs on each evaluation set (E918-IV, E918-CT, and E918-CM) in terms of the Wilcoxon-Mann-Whitney p-values. In the work by Gysi et al.[38] the pre-computed embeddings are available only for the AI-based methods. For the other methods, only the rankings are available. Therefore, we computed the raw scores of a proximity based method (the Guney distance[48]) and a diffusion based method (the DSD method[47]). We used the original implementation of these two methods, and due to considerable computational costs, we used the Guney distance[48] as the representative for all the Proximity based methods, and DSD[47] as the representative for all the diffusion based methods. We observed that our approach (based on kernels) and DSD are the only methods with significant p-values in all evaluation sets. Notably, the AI based methods are only aligned with the clinical trial data, but not to the other two sources of evidence.

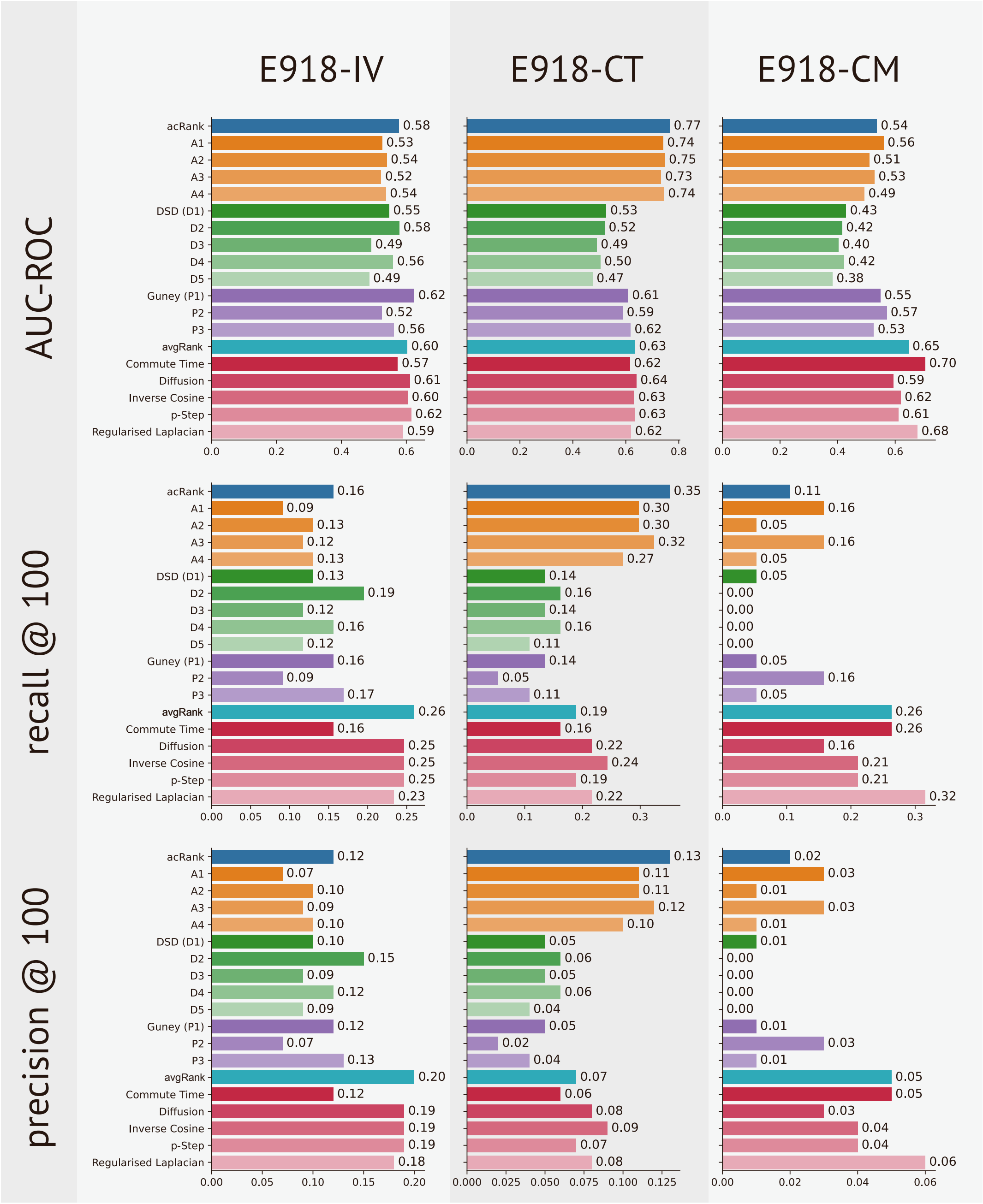

Figure S8. Performance comparison of our methods to the methods used in Gysi et al.[38]. We observe that our network medicine approach is competitive across all evidence types with the Proximity based methods (based on the Guney distance), as well as the diffusion methods (based on the DSD method). In these cases, our network medicine approach is competitive in the AUC-ROC metric, but stronger in the precision and recall metrics. When compared to the GNN based methods by Gysi et al. , we see that the AI based methods perform quite well on the clinical trial evidence, but an overall lower performance on other types of evidence.

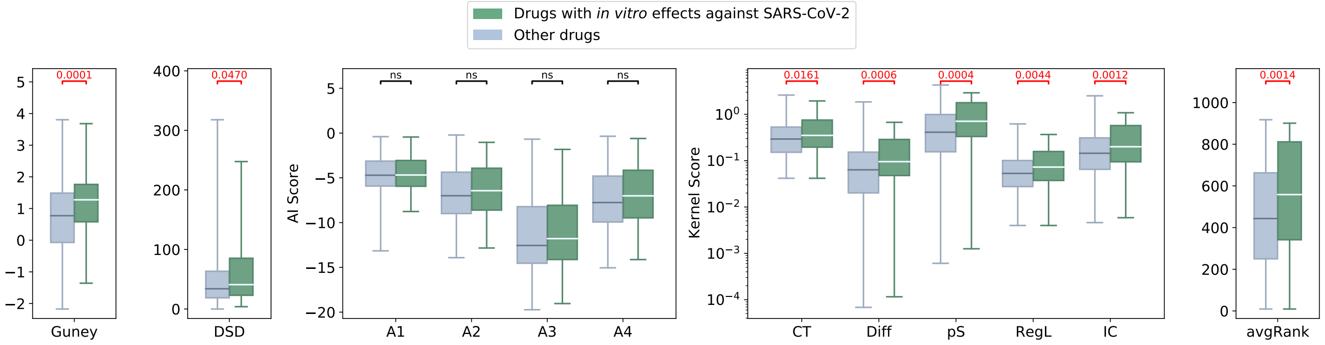

Figure S9. Score comparison between our network medicine approach, and methods used in Gysi et al.[38] on the E918-IV (*in vitro*) evaluation set. Notably, none of the AI methods assign significantly different scores to drugs with *in vitro* effect vs. all other drugs in this evaluation set. The Guney distance, the DSD method, the average ranking (avgRank), and all of our network medicine approach methods do align to this type of evidence.

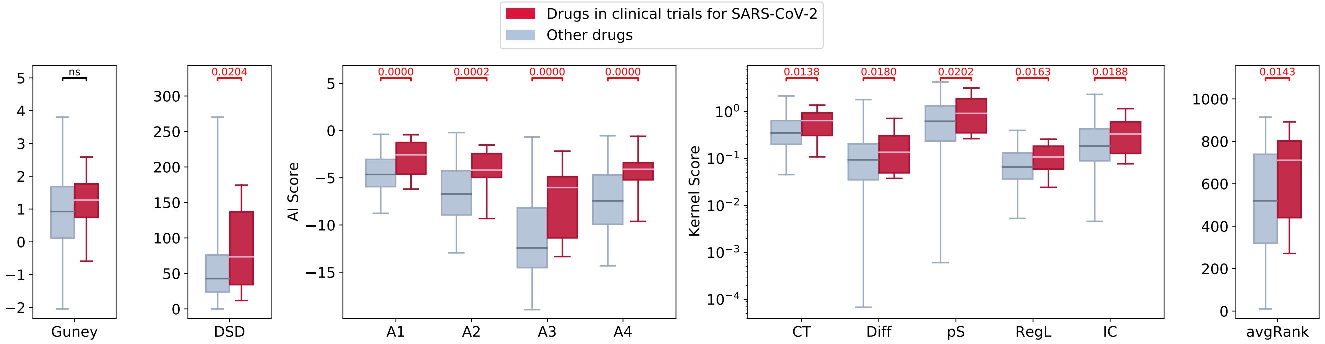

Figure S10. Score comparison between our network medicine approach, and methods used in Gysi et al.[38] on the E918-CT (clinical trials) evaluation set. For this evaluation setting, with the exception of the Guney distance, every method assigns significantly different scores to drugs in Clinical trials on April 15th, 2020.

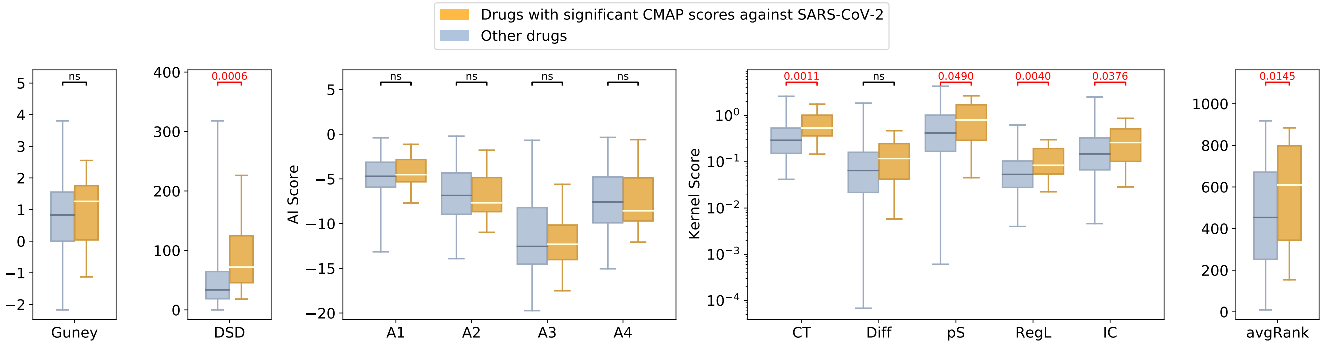

Figure S11. Score comparison between our network medicine approach, and methods used in Gysi et al.[38] on the E918-CM evaluation set. For this evaluation setting, the AI is also unable to assign significantly different scores to drugs with significant CMAP scores. This is also the case for the Guney distance, and our network medicine score calculated on the Diffusion kernel.

7 Note S7. Evaluation of the network medicine approach on additional PPIs

We evaluated the network medicine approach on two additional interactomes: the Cheng et al.[40] PPI, and the HuRI PPI[41], as described in Table S3. We considered 1853 FDA-approved drugs with targets in these interactomes. Out of the 1853 drugs, 78 have *in vitro* evidence (IV-1853 set), 153 have clinical trials evidence (CT-1853 set), and 21 have CMAp evidence. Details on our evaluation dataset are described in Section 4.

In Figure S12, we compare prediction scores between drugs with evidence for COVID-19 and the remaining drugs, on the different PPIs and types of evidence. We also show the corresponding Wilcoxon-Mann-Whitney p-values. Compared to the competitors (DSD[47], and the Guney distance[48]), our network medicine is the only one with predictions significantly aligned with *in vitro*, and CMAP evidence in all PPIs.

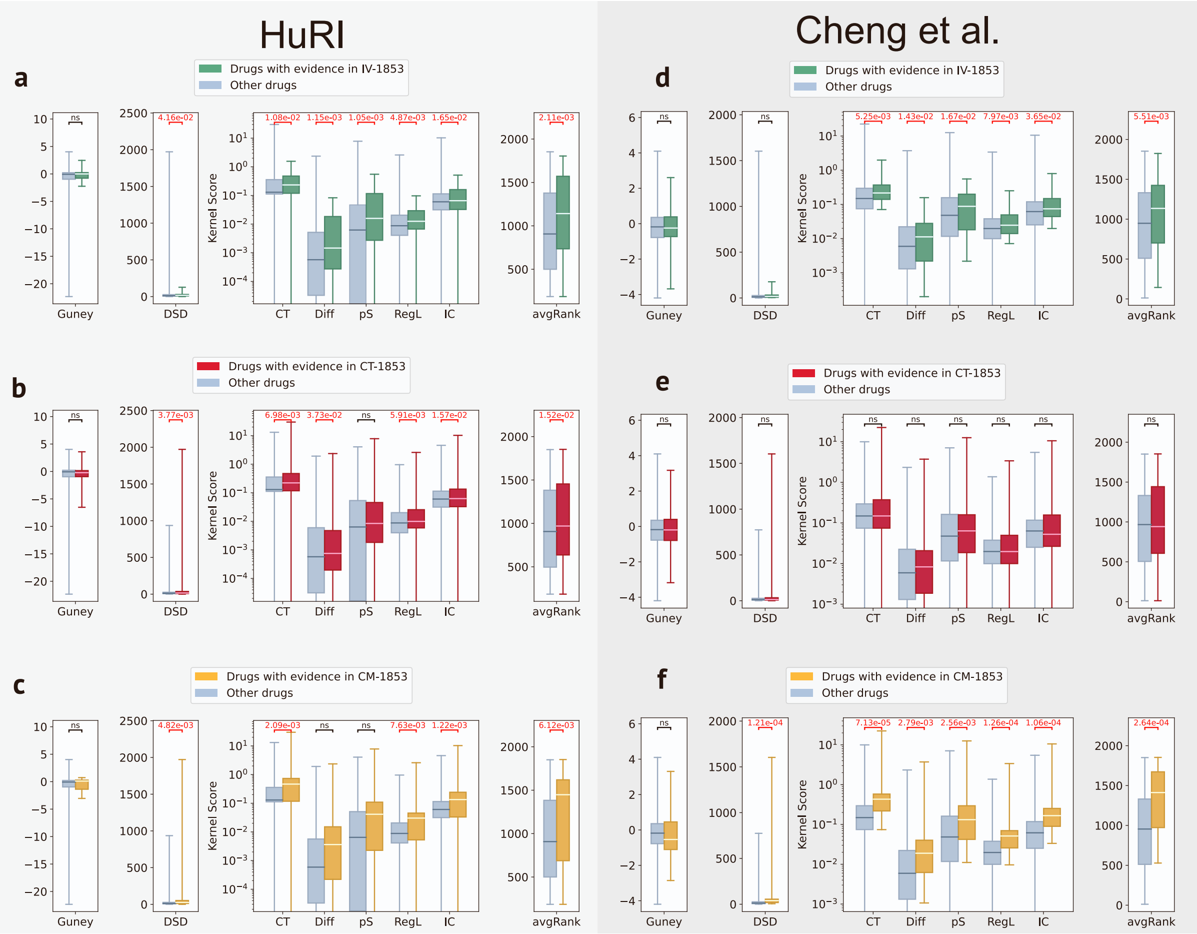

Figure S12. Evaluation of the network medicine approach on additional interactomes. We used boxplots to compare prediction scores between drugs with and without evidence for COVID-19 on three different evaluation sets: *in vitro* evidence (IV-1853), clinical trials evidence (CT-1853), and CMAP evidence (CM-1853). For each comparison, we computed the Wilcoxon-Mann-Whitney p-value (one tailed test). Non-significant p-values (below 0.05) are indicated with 'ns'. Significant p-values are shown in red. We show results obtained by our network medicine approach and competitors. Our approach includes five different kernels on graphs (commute time, diffusion, inverse cosine, p-step kernel, and regularised Laplacian), and a combined ranking across the kernels (avgRank). The competitors include DSD[47], and the Guney distance[48].

The only scenario in which our approach does not have a significant alignment is when we evaluate predictions for clinical trials evidence on the Cheng et al. PPI. In this case, none of the competitors had significant p-values. Notice that, however, in the HuRI PPI prediction scores are significantly different between drugs on clinical trials and the remaining drugs for both our approach and DSD.

It is also interesting to see how our network medicine approach and the competitors perform with traditional machine learning metrics. We show the area under the ROC curve (AUC-ROC), recall at top 150, precision at top 150, and MAP for the Cheng et al., and HuRI PPIs in Figures S13, and S14 respectively. We noticed that in most scenarios the kernel-based

methods have the highest values of AUC-ROC, and MAP. However, even when the AUC-ROC, and MAP are not the highest, our approach predicts earlier on the top 150. That is, in every case, our network medicine approach has the highest recall and precision at the top 150.

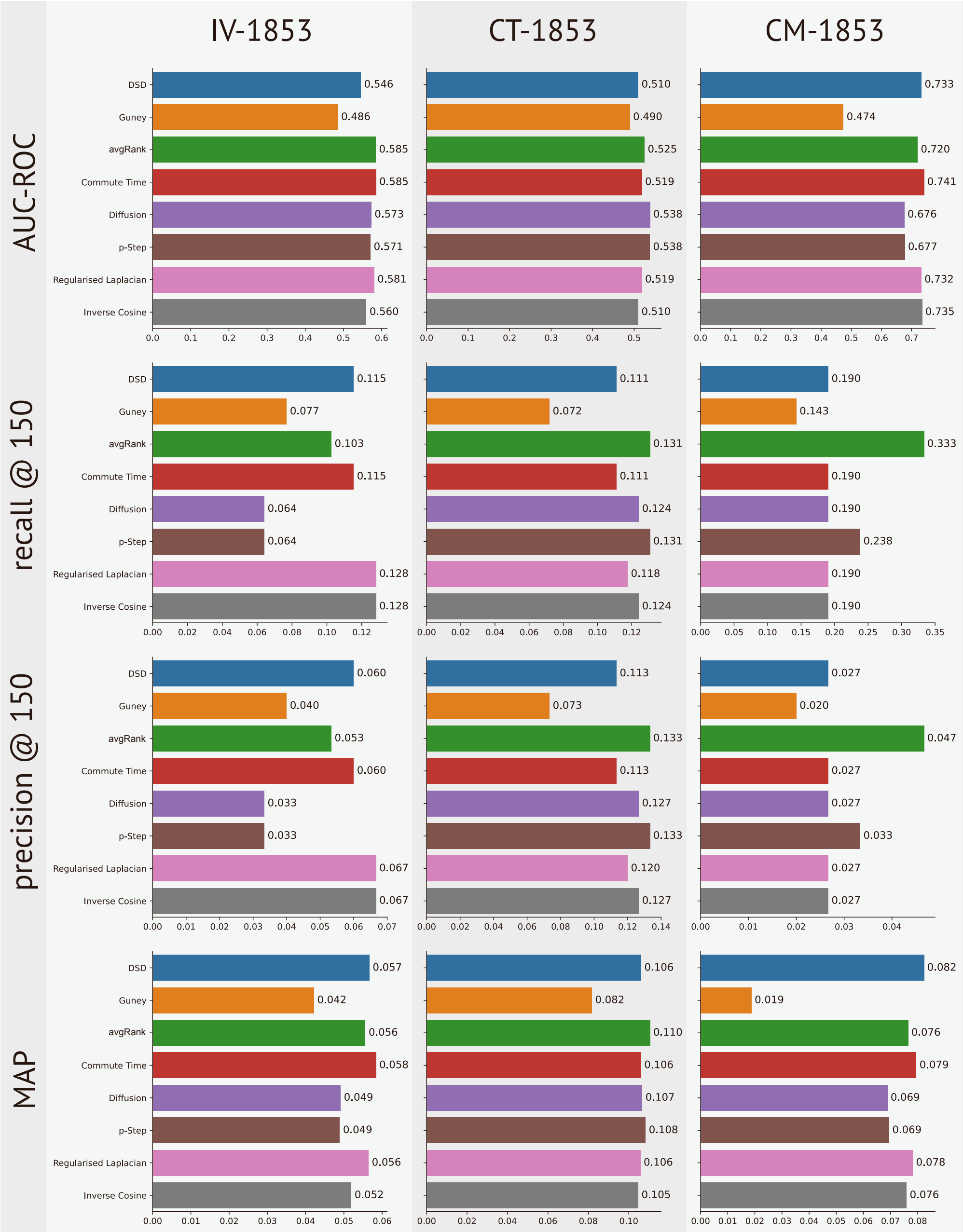

Figure S13. Classical machine learning metrics obtained by the network medicine approach on Cheng et al. PPI. We computed the area under the ROC curve (AUC-ROC), recall at top 150, precision at top 150, and mean average precision (MAP) for three different evaluation sets. They correspond to *in vitro* evidence (IV-1853), clinical trials evidence (CT-1853), and CMAP evidence (CM-1853). For each type of evidence, we show results obtained by our network medicine approach and competitors. Our approach includes five different kernels on graphs (commute time, diffusion, inverse cosine, p-step kernel, and regularised Laplacian), and a combined ranking across the kernels (avgRank). The competitors include DSD[47], and the Guney distance[48].

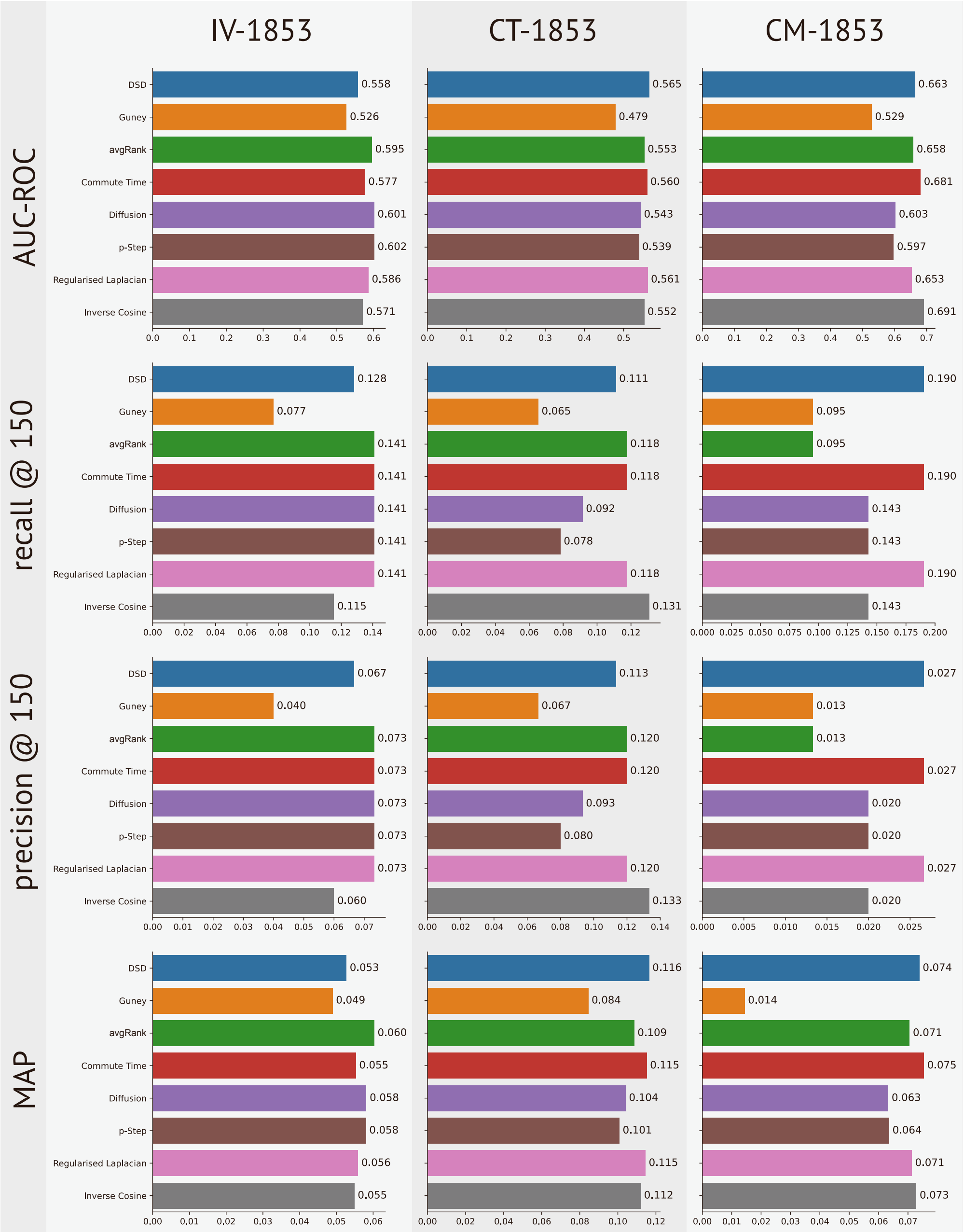

Figure S14. Classical machine learning metrics obtained by the network medicine approach on HuRI et al PPI. We computed the area under the ROC curve (AUC-ROC), recall at top 150, precision at top 150, and mean average precision (MAP) for three different evaluation sets. They correspond to *in vitro* evidence (IV-1853), clinical trials evidence (CT-1853), and CMAP evidence (CM-1853). For each type of evidence, we show results obtained by our network medicine approach and competitors. Our approach includes five different kernels on graphs (commute time, diffusion, inverse cosine, p-step kernel, and regularised Laplacian), and a combined ranking across the kernels (avgRank). The competitors include DSD[47], and the Guney distance[48].

Therefore, similarly to results with the Gysi PPI[38] used throughout the manuscript, our predictions on additional PPIs are the most consistently aligned with the three sources of evidence for COVID-19 (*in vitro*, clinical trials, and CMAP).

8 Note S8. Integration of gene expression for network medicine

We compared results obtained with the binary and weighted versions of the network medicine approach on our three evaluation sets (*in vitro*, clinical trials, and CMAP).

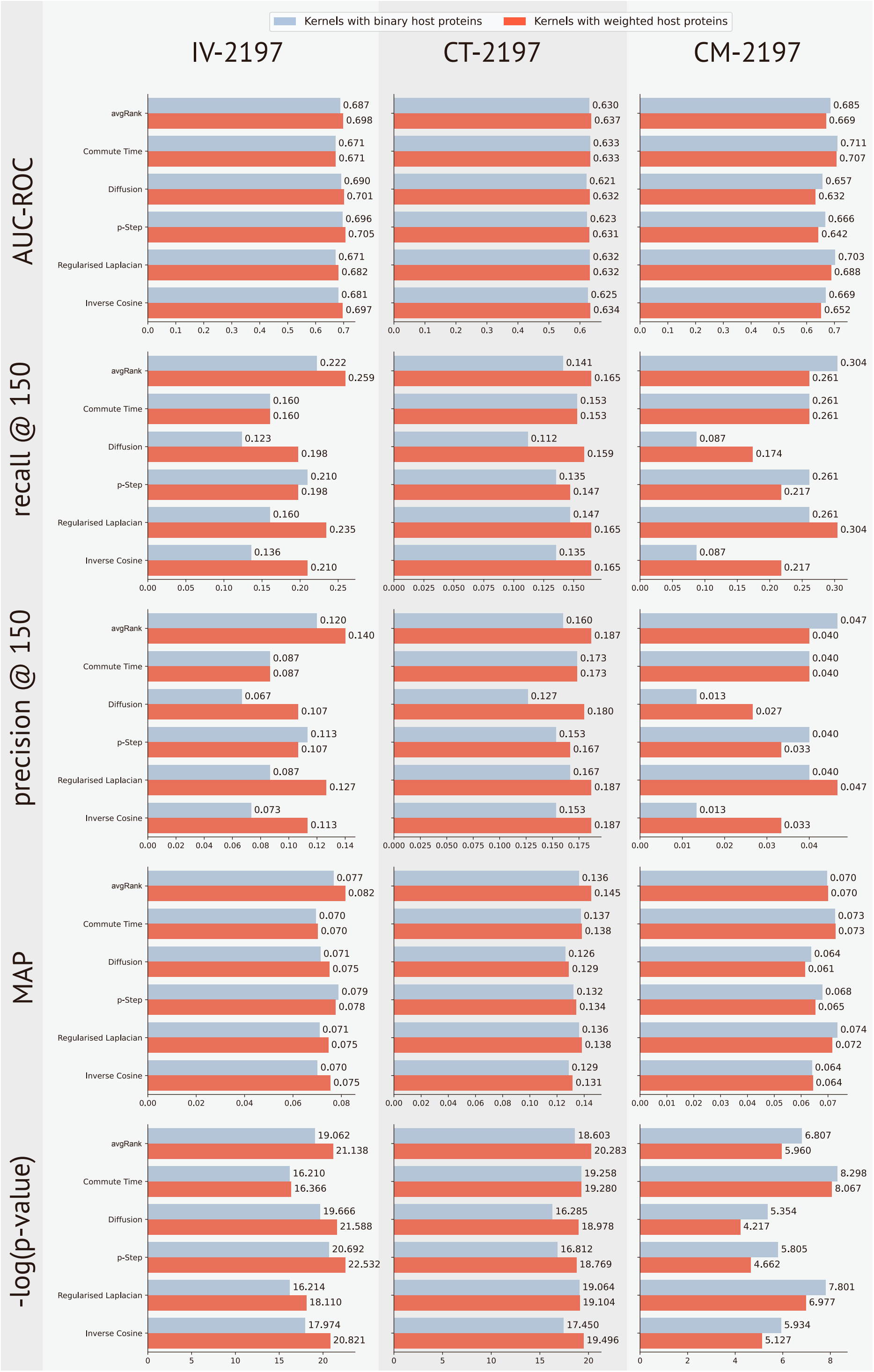

Figure S15. Comparison between binary and weighted kernel scores. We observe consistent improvement on the results of the weighted version for *in vitro* (IV-2197) and clinical trial (CT-2197) evidence. For CMAP (CM-2197), the AUC-ROC, MAP, and p-value metrics are comparable, while a notable improvement is observed in the recall and precision at top 150 metrics. Notice that we show the negative logarithm of the p-values. Thus, the higher the value, the more significant the p-value is.

Figure S15, Figure S16 and Table S5 present, for each evaluation set, four different machine learning metrics for evaluating the performance (AUC-ROC, recall@top 150, precision@top 150, MAP); as well as, and the Wilcoxon-Mann-Whitney p-value obtained comparing prediction scores between set of drugs (as described in the paper).

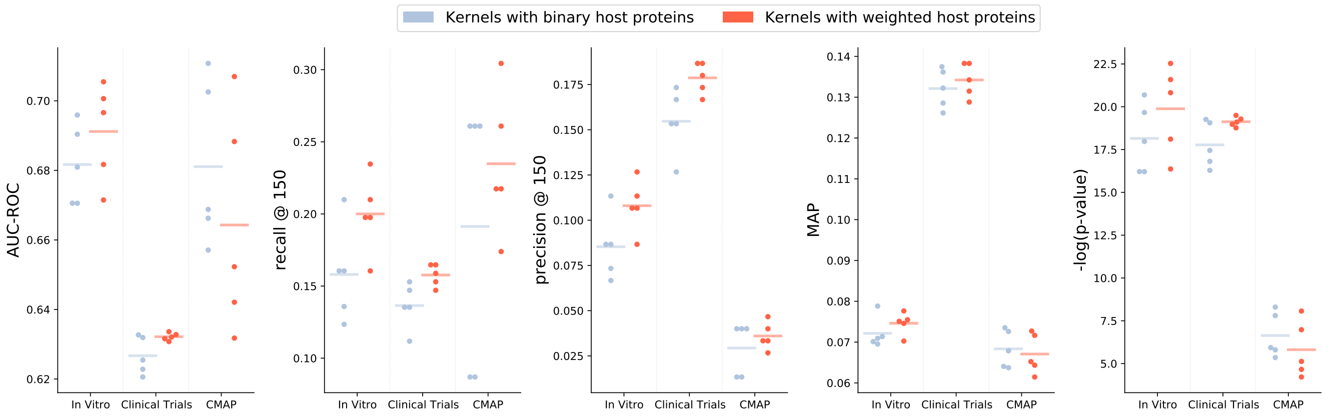

Figure S16. Distribution of five different performance metrics (y-axis) across the five kernels used by the network medicine approach. Each point corresponds to an evaluation metric obtained by a kernel on a specific type of evidence (x-axis). The horizontal lines correspond to the average values. When comparing the binary vs. weighted versions of the kernel methods, we observe a consistent improvement in the weighted version for all performance measures on *in vitro* and Clinical Trials evidence. For CMAP, the AUC-ROC, MAP, and p-value metrics are comparable, while a notable improvement is observed in the recall and precision at top 150 metrics. Notice that we show the negative logarithm of the p-values. Thus the higher the value, the more significant the p-value is.

**Evidence**	**Metric**	**Mean**		**Standard deviation**	
		**Binary**	**Weighted**	**Binary**	**Weighted**
In Vitro	recall @ 150	0.169	**0.210**	0.040	0.034
	precision @ 150	0.091	**0.113**	0.021	0.018
	MAP	0.073	**0.076**	0.004	0.004
	AUC	0.683	**0.692**	0.010	0.013
	-log(p-value)	18.303	**20.093**	1.844	2.351
**Clinical Trials**	recall @ 150	0.137	**0.159**	0.014	0.007
	precision @ 150	0.156	**0.180**	0.016	0.008
	MAP	0.133	**0.136**	0.005	0.006
	AUC	0.627	**0.633**	0.005	0.002
	-log(p-value)	17.912	**19.318**	1.240	0.535
**CMAP**	recall @ 150	0.210	**0.239**	0.097	0.046
	precision @ 150	0.032	**0.037**	0.015	0.007
	MAP	**0.069**	0.068	0.004	0.005
	AUC	**0.682**	0.665	0.021	0.029
	-log(p-value)	**6.667**	5.835	1.181	1.469

Table S5. Average and standard deviation of evaluation metrics across the five different kernels (commute time, diffusion, inverse cosine, p-step, and regularised Laplacian). The largest mean values are highlighted in bold. We compared results between kernel-based methods with (weighted) and without (binary) weights on the host proteins. We observed a consistent improvement in the weighted version for all performance measures on *in vitro* and Clinical Trials evidence. For CMAP, the AUC, MAP, and p-value metrics do not show improvement, while a notable increase is observed in the recall and precision at top 150 metrics. Notice that we show the negative logarithm of the p-values. Thus the higher the value, the more significant the p-value is.

Our results show that the AUC-ROC and MAP increase, and p-values became more significant in the weighted version for

both *in vitro* and clinical trials evidence. Importantly, the gene expression data is helping in retrieving drugs with evidence earlier in our ranking.

In fact, there is a consistent increase in the recall and precision for all types of evidence in the weighted version. On average, the recall increased 31%, 16.9%, and 50% for *in vitro*, clinical trials and CMAp evidence, respectively. Similar improvements can be seen for the precision. Notice that for the CMAP evaluation, recall and precision became higher even for kernels for which the AUC-ROC, and MAP did not increase, as shown in Figure S15. The AUC-ROC increases on average 1.4% and 0.9% on *in vitro* and clinical trials, respectively. Proportionally, MAP increases more by incorporating gene expression data into the network medicine approach. It has an average increase of 4.1% for *in vitro* evidence, and 1,3% for clinical trials evidence.

9 Note S9. Overlap between our predictions and predictions by Gordon et al

Gordon et al. identified 69 compounds that target 66 out of the 332 host proteins used by SARS-CoV-2 infection and replication[49]. A subset of these and 28 additional compounds were screened in viral assays[49]. Two sets of pharmacological agents displayed antiviral activity: inhibitors of mRNA translation and predicted regulators of the sigma-1 and sigma-2 receptors.

Altogether, the analyses by Gordon et al. resulted in four lists of candidate drugs against COVID-19, described below:

Set 1 **(Chemoinformatics)** 37 compounds identified by chemoinformatics searches.

Set 2 **(Literature)** 32 compounds identified by target- and pathway-specific literature search.

Set 3 **(Multiple)** 75 compounds tested for *in vitro* activity against SARS-CoV-2. It contains a subset of 47 compounds identified by 1 and 2, plus 13 to expand testing of the sigma-1 and sigma-2 receptors and mRNA translation targets, and 15 additional molecules that had been prioritized by other methods.

Set 4 **(In vitro)** a subset of 3, consisting of 13 compounds with *in vitro* activity against COVID-19.

Some of our top predictions include compounds in these lists. In the top 20, our matrix decomposition approach predicts Chloroquine (set 1), Azithromycin (set 3), Nitazoxanide (set 3), and Hydroxychloroquine (set 4); and our network medicine approach predicts Progesterone (set 3).

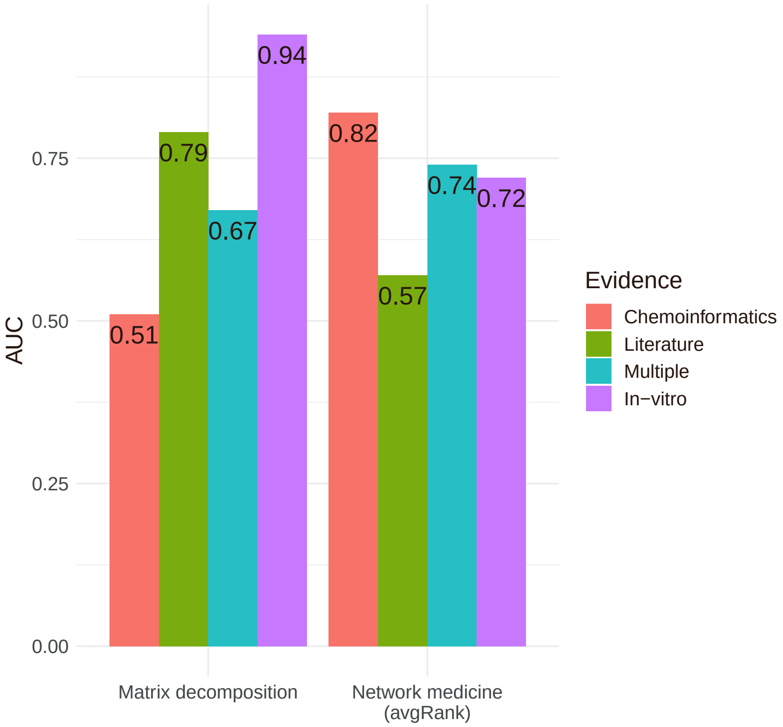

Figure S17. Area under the ROC curve for predicting drugs identified by Gordon et al.[49], which are classified into four different sets. The first set (Chemoinformatics) includes drugs identified by chemoinformatics search. The second set (Literature) contains compounds found by target- and pathway-specific literature search. The third set has compounds identified by multiple sources (Multiple), including chemoinformatics, literature, and other methods. Finally, the last set (*in vitro*) includes a subset of the third set with *in vitro* activity in the assays by Gordon et al.[49].

Another interesting question is how the entire set of drugs selected by Gordon et al. is ranked across our predictions. To answer this question, we formulated an equivalent binary classification problem, so that we could use ROC curve analysis. For each set of drugs, we labelled the compounds within the set as “positive”, and the remaining ranked drugs as “negative”. Thus, for the matrix decomposition approach, our dataset with 126 drugs contains 5 compounds from set 1, 3 drugs from set 2, 10

drugs from set 3, and 1 drug from set 4 that we labelled as positive. For the network medicine approach, our dataset of 2197 approved drugs, contains 14 drugs from set 1, 7 from set 2, and 27 from set 3, and 3 drugs from set 4. Results are shown in Figure S17, which presents the AUC ROC for our two approaches, separately on the 4 different sets of drugs.

Our matrix decomposition model has a better alignment with predictions by Gordon et al.[49] for drugs with *in vitro* efficacy in theirs assays ("*in vitro*" - set 4), compounds identified by target- and pathway-specific literature ("Literature" - set 2), and multiple sources of evidence ("Multiple", set 3). This is in accordance with the data used by this approach, which contains associations between viruses and drugs based on the literature.

For the network medicine approach, we show the results obtained by the avgRank model, which has an AUC larger than 0.5 for all sets identified by Gordon et al.[49]. Our network medicine approach is best at prioritizing drugs identified by chemoinformatics search and a possible reason for this is that these drugs often target a host factor or its close by neighbours.

10 Note S10. CoREx Network Combination

We begin by using InterPro[50] predictions $R \in \mathbb{R}^{n \times t}$ for n proteins and t GO terms. We then build a network of functional similarities T, defined as:

$$T_{ij} = \frac{|N_i \cap N_j|}{|N_i \cup N_j|}$$

where N_i and N_j are the sets of all GO terms above a threshold τ that are associated to proteins i and j respectively in R, that is, $N_i = \{k|R_{ik} > \tau\}$, and $N_j = \{k|R_{jk} > \tau\}$. Therefore, T_{ij} is the Jaccard similarity between sets of GO terms that are assigned by InterPro to proteins i and j.

Given p networks $W^{(r)}$ with $r \in (1 \cdots p)$, we combine them into a single network W using a weighted linear combination. The vector of weights $\hat{c} \in \mathbb{R}^p$ is learnt by minimising the square of the difference between T and the linear combination:

$$\left(\hat{c}, \hat{b}\right) = \underset{c,b}{\operatorname{argmin}} \sum_{i,j} \left(b + \sum_{r=1}^{p} c_r W_{ij}^{(r)} - T_{ij} \right)^2$$

and $\hat{b}$ is used to remove the bias in T. This linear regression can be solved efficiently, and we can interpret each learnt coefficient c_r as representing how much each network r contributes to the combination.

11 Note S11. Analysis of the top predictions by the two approaches

11.1 Matrix decomposition approach

Tables S1 and S2 show the top predicted drugs using our matrix decomposition approach. Table S2 shows predictions of type A — drugs under development, but not approved, for SARS-CoV-2 according to the Andersen et al.[14] dataset (their developmental stages were used for training).

We observed that these predicted BSA drugs are known to interfere with SARS-CoV-2 infection through different molecular mechanisms. The nucleotide analogue inhibitors Remdesivir[51] and Fapinavir[52] inhibit the RNA-dependent RNA polymerase (RdRp) of SARS-CoV-2. Lopinavir/ritonavir is a protease inhibitor, which may inhibit the 3Clike protease of SARS-CoV-2, and Chloroquine/Hydroxychloroquine might interfere with the replication of SARS-CoV-2 by multiple mechanisms[53]. Arbidol has been found effective against SARS-CoV-2 *in vitro* by inhibiting viral attachment and release of SARS-CoV-2 from intracellular vesicles[30].

The drug with the highest predicted score was Favipiravir, already known to be in phase II of clinical trials for COVID-19 according to the Andersen et al. dataset. Favipiravir is a RNA polymerase inhibitor that was developed in Japan as an antiviral treatment for influenza. In October 2020, already 37 clinical trials were registered in ClinicalTrials.gov to assess its efficacy and safety for COVID-19 patients[26]. A systematic review of the randomised clinical trials suggest that Favipiravir have shown low efficacy for patients with mild or moderate COVID-19[26]. In fact, other drugs in Table 2, including Remdesivir, Lopinavir and Hydroxychloroquine have shown little to no clinical efficacy in COVID-19 patients according to the WHO Solidarity Trial, conducted in 405 hospitals in 30 countries[33].

However, although several of these BSA drugs have been found effective *in vitro* or in vivo against SARS-CoV-2, many of them have proven with little to no efficacy in clinical trials in COVID-19 patients. For instance, our drug with the highest predicted score is Favipiravir, already known to be in phase II of clinical trials for COVID-19 according to the Andersen et al.[14] dataset. In October 2020, already 37 clinical trials were registered in ClinicalTrials.org to assess its efficacy and safety for COVID-19 patients[26]. A systematic review of the randomised clinical trials suggests that Favipiravir has shown low efficacy for patients with mild or moderate COVID-19[26]. In fact, other drugs in Table S2, including Remdesivir, Lopinavir and Hydroxychloroquine have shown little to no clinical efficacy in COVID-19 patients according to the WHO Solidarity Trial, the largest clinical trial conducted in more than 11,000 patients across 405 hospitals in 30 countries[33].

The lack of efficacy of BSA drugs that were under development at the time of release of the Andersen et al. dataset[14] suggests that it is important to consider predicted BSA drugs that were not yet under development against SARS-CoV-2 — i.e., our predictions of type B.

Our top predicted drugs of type B, not under development for SARS-CoV-2 according to the Andersen et al.[14] dataset, are shown in Table S1. Overall, we found that several of these drugs are already in clinical trials for COVID-19. We comment more in detail on a few entries in the table.

Tenofovir, our top predicted drug, is a broad-spectrum antiviral drug active against HIV and hepatitis B, and it was found to interfere with the SARS CoV-2 ribonucleic acid (RNA)-dependent RNA polymerase (RdRp), an enzyme indispensable for SARS-CoV-2 replication, in experiments *in vitro*[54], and *in vivo*[55]. In addition, a recent phase II clinical trials in two hospitals in France show that Tenofovir, in combination with Emtricitabine, accelerated the natural clearance of nasopharyngeal SARS-CoV-2 viral burden[56].

Lamiduvine is the second predicted BSA drug in Table S1. The potential of repurposing Lamivudine for COVID-19 is currently under investigation. There is evidence that indicates that Lamiduvine might work as an inhibitor of the SARS-CoV-2 RdRp RNA polymerase[57]. Yet, further experiments *in vitro* and *in vivo* are needed to confirm this hypothesis.

Interestingly, we also predicted the anti-malarial drug Artesunate, which has been found effective against SARS-CoV-2 in several *in vitro* assays[58]. In fact, Artesunate is one of the three drugs that have been selected for the currently ongoing WHO Solidary Plus Clinical Trials to assess its efficacy in COVID-19 patients[25].

Rank	Drug name (ID)	Main ATC Category	Additional curated evidence for COVID-19
1	Fostamatinib (DB12010)	Blood and blood forming organs (B)	several clinical trials (NCT04579393, NCT04581954, NCT04629703), *in silico* evidence[59]
2	NADH (DB00157)		in Clinical trials (NCT04604704), *in silico* evidence[60–62]
3	Copper (DB09130)		Combinatorial therapy[63], *in silico* evidence[64]
4	Cannabidiol (DB09061)	Nervous System (N)	In Clinical Trials (NCT04467918)
5	Glutathione (DB00143)	Various (V)	In Clinical Trials CTRI/2021/01/030793
6	Doxorubicin (DB00997)	Antineoplastic and immunomodulating agents (L)	*in silico* evidence[65]
7	Flavin adenine dinucleotide (DB03147)		*in silico* evidence[64]
8	Verapamil (DB00661)	Cardiovascular System (C)	multiple clinical trials (NCT04351763, NCT04330300, NCT04467931)
9	Zinc (DB01593)	Cardiovascular System (C)	included in more than 60 clinical trials, *in silico* evidence[64]
10	Zinc acetate (DB14487)	Cardiovascular System (C), Alimentary tract and metabolism (A)	included in more than 60 clinical trials, *in silico* evidence[64]
11	Zinc chloride (DB14533)	Blood and blood forming organs, Cardiovascular System (B)	included in more than 60 clinical trials, *in silico* evidence[64]
12	Moexipril (DB00691)	Cardiovascular System (C)	in clinical trials (NCT04467931)
13	Conjugated estrogens (DB00286)	Genito-urinary system and sex hormones (G)	NA
14	Clozapine (DB00363)	Nervous System (N)	NA
15	Rifampicin (DB01045)	Antiinfectives for systemic use (J)	*in silico* evidence[66]
16	Amitriptyline (DB00321)	Nervous System (N)	*in vitro* evidence[38]
17	Phenobarbital (DB01174)	Nervous System (N)	NA
18	Desipramine (DB01151)	Nervous System (N)	NA
19	Progesterone (DB00396)	Genito-urinary system and sex hormones (G)	in several clinical trials (NCT04365127, NCT04539626, NCT04865029 - combinatorial therapy)
20	Ethanol (DB00898)	Dermatologicals (D), Various (V)	in more than 100 clinical trials.

Table S6. Kernel-based top-20 predicted drugs. For each drug we show whether there is evidence from other *in silico* approaches, *in vitro* experiments, or clinical trials.

11.2 Network medicine approach

To obtain the scores for the 2197 FDA-approved drugs, we used the average ranking (avgRank) across the five different graph kernels. In our calculations, we used the interactome provided by Gysi et al.[38], and the SARS-CoV-2 host-proteins were weighted in our model using gene expression data from nasopharyngeal swabs[67] (GEO:GSE152075).

We started by analysing whether there was a trend among the top predicted drugs. Figure S18 below shows the distribution of main Anatomical, Therapeutic and Chemical (ATC) classification of the top-20 predicted drugs. We observed that half of the predicted drugs belong to either Nervous System (N) or Cardiovascular System (C) drugs. In fact, we found an enrichment of nervous system drugs (psychoanaleptics, N06) and cardiovascular system drugs (vasoprotectives, C05) amongst the top-100 predictions (p = 0.01 and p = 0.03, respectively, hypergeometric Test of Significance with FDR multiple testing correction).

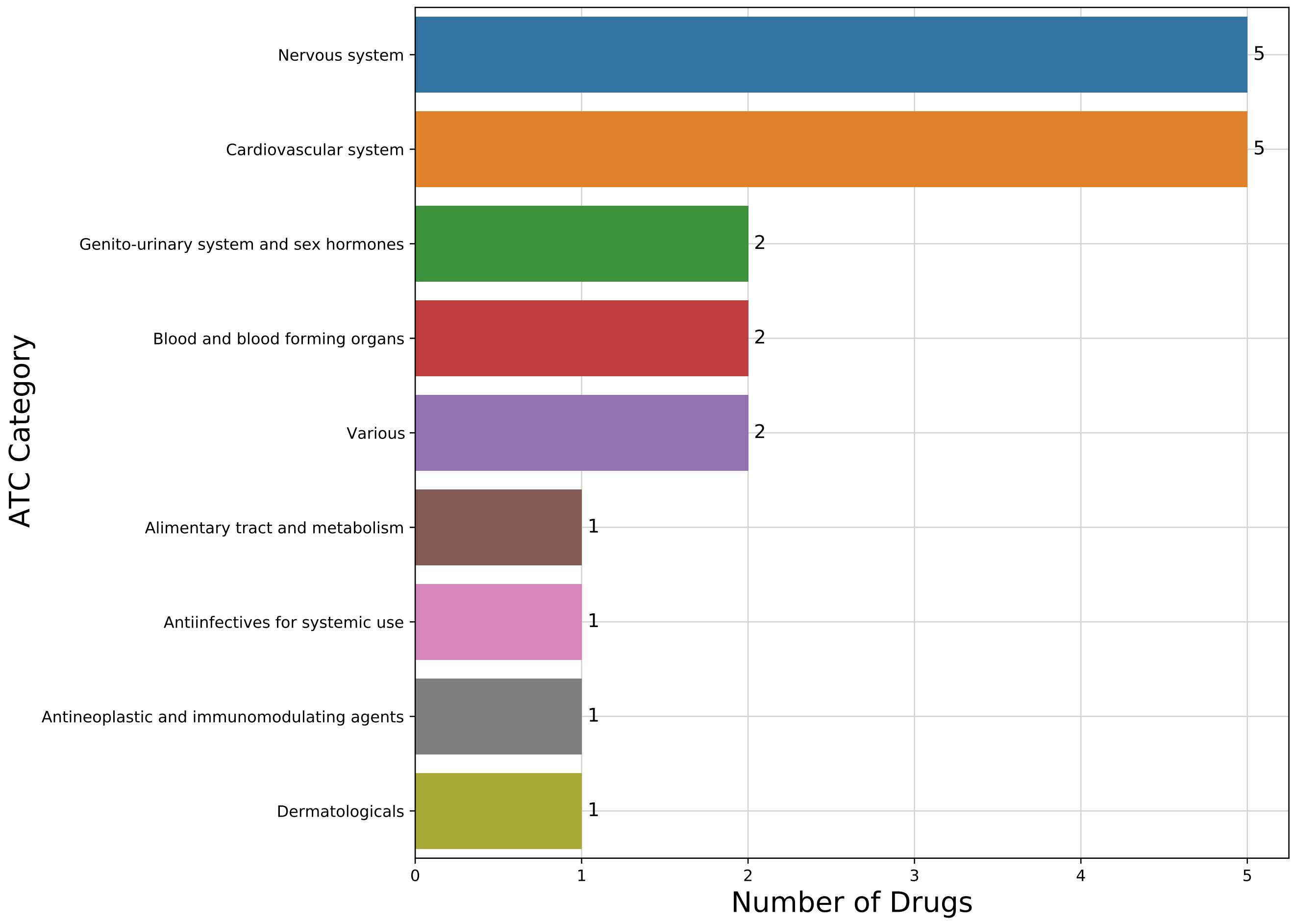

Figure S18. Distribution of main Anatomical, Therapeutic and Chemical (ATC) categories of the top-20 predicted drugs with our network medicine approach

We further analysed each of these top-20 predicted drugs in more detail (see Table S6).

The top scoring drug is Fostamatinib, that was identified as a candidate for rapid repurposing for COVID-19 patients through a high content screen for Mucin-1 (MUC1) reducing compounds[68]. Elevated levels of MUC1 predict the development of Acute Lung Injury (ALI). By its MUC1 reducing effect, the potential of Fostamatinib to manage the acute respiratory distress syndrome (ARDS), often fatal in COVID-19 patients is highglighted[68]. Furthermore, there is evidence that Fostamatinib counteracts the antiinflammatory response caused by the anti-spike IgG, instrumental in the worsening of COVID-19 cases[69].

Our top predictions also include drugs that interact with the SARS-CoV-2 host receptor protein, angiotensin-converting enzyme 2 (ACE2) and/or the ACE2/TMPRSS2 pathway[70,71]. For example, cannabidiol (top 4) has been shown to modulate ACE2 expression in COVID-19 gateway tissues, providing scientific rationale for clinical trials[72]. Conjugated estrogens (top 13) are linked to the reduced effect of SARS-CoV-2 on females[73], and there is promising evidence of using them as therapeutics for COVID-19 patients through their interaction with the ACE2/TMPRSS2 pathway[73,74]. Another drug used in hormone therapy that is on our top predictions is Progesterone. It shows promising therapeutic characteristics to improve the immune dysregulation that leads to the COVID-19 cytokine storm[75].

Another interesting group of drugs in Table S6 is zinc, zinc acetate, and zinc chloride (top 9, 10, and 11 respectively). Noticeably, most risk groups described for COVID-19 are associated with zinc deficiency[76], and there is encouraging evidence that zinc supplements are helpful to prevent pathogen entry[76]. Treatment with zinc acetate and zinc gluconate have also accelerated the recovery of COVID-19 patients[77], and they are linked to rapid resolution of COVID-19n shortness of breath[78].

In the ranking, there are drugs that have been found useful in other applications for COVID-19. For instance, vitamin supplements ingredients NADH (top 2), and Flavin adenine dinucleotide (top 7) have been identified as potential biomarkers for fluorescence, label free detection of COVID-19 on early stages employing portable optical detection systems[79]. There is evidence suggesting that copper (top 3) in nasal lavages might be both safe and could be used to reduce or even stop viral transmission[80].

It is important to note that our network medicine approach works by modelling the mechanistic effects on the interactome. This means that a high score points to a high probability of molecular interplay between the drug and the COVID-19 disease module. However, while our kernel methods, like most network approaches[38], can quantify the perturbation on the interactome but cannot predict in which way the host will ultimately be affected by such perturbations. Therefore, some drugs that interact closely to the COVID-19 disease module, do not show therapeutic potential. Examples are Clozapine, that was linked to increased risk of COVID-19 infection[81,82], and Rifampicin, with associated warnings against using it together with Ritonavir, as well as caution when prescribing it in combination with Rifapentine for HIV/COVID-19 co-infection due to potential drug-drug interactions[83].

12 Note S12. Analysis of the overlap between both approaches

We analyzed the overlap between predictions by both approaches. Out of 126 drugs predicted by our matrix decomposition model, 80 were also considered by the network medicine approach.

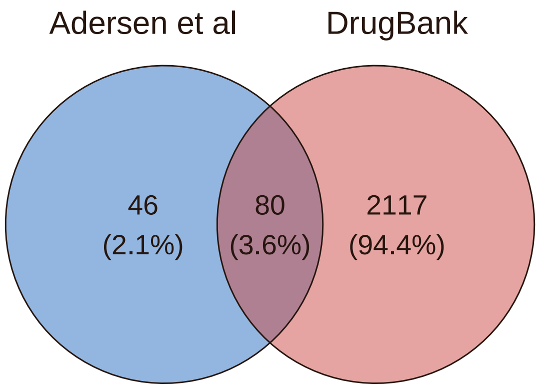

Figure S19. Venn diagram of drug sets used by the matrix decomposition and network medicine approaches. 80 were ranked by both approaches. Our matrix decomposition model uses data by Andersen et al.[14] (blue set on the left). Our network medicine approach uses all FDA-approved drugs in DrugBank that have known targets in the interactome (red set on the right).

Figure S20 shows where the 80 drugs in the intersection were ranked by the network medicine approach. The drugs in the overlap tend to be ranked in the top section of the ranking.

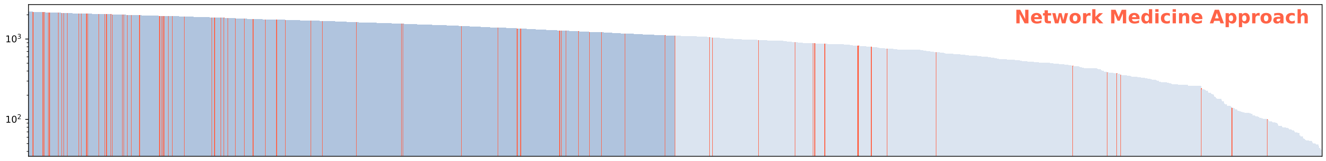

Figure S20. The 2197 drugs ranked by the network medicine approach, sorted by the output score of the method (higher to lower). Orange bars correspond to BSAs that are also ranked by the matrix decomposition approach. We observe that the vast majority of BSAs are ranked high by the network medicine approach. This suggests that BSAs are, perhaps unsurprisingly, good repurposing candidates for a viral disease such as COVID-19.

Among the top predictions by the matrix decomposition model which are also ranked high by the network medicine approach we find, for example, Ritonavir. It is the 4th best in the matrix decomposition model and is among the top 30 predictions by the network medicine approach. It acts not only as a HIV protease inhibitor, but also as a pharmacokinetic enhancer of other protease inhibitors, mainly due to its potent inhibition of the cytochrome P450 3A4 isoenzyme[84]. Thus, it can be considered both a direct acting antiviral drug, and a host targeted agent.

In addition, there are drugs predicted by both approaches that target only host factors. For example, Amiodarone (on the top 20 by the matrix decomposition model) is on the top 100 predictions by the network medicine approach. It was originally proposed as an antiarrhythmic, but also has proven efficacy against RNA viruses *in vitro* (including SARS-CoV-1)[85,86], and it went into clinical trials Phase 3 for treating Ebola (NCT02307591). Its antiviral activities are probably related to its interference with the endocytic pathway, and additional mechanisms[85].

Another interesting example is Tamoxifen, which was ranked on the top 30 predictions by both approaches. It is a selective estrogen receptor modulator used to treat or reduce the risk of breast cancer[87]. Later, Tamoxifen, and other estrogen receptor modulators have shown *in vitro* and *in vivo* activity against a wide range of human pathogens, including viruses, fungi, parasites, and bacteria[88]. The *in vitro* efficacy of Tamoxifen against HIV replication was attributed to inhibition of PKC and interaction with other targets in the NF-κB pathway[89]. Another study suggested that Tamoxifen inhibits HCV replication by interfering with the association of estrogen receptor alpha with RNA-dependent RNA polymerase NS5B, which affects the formation of the replication complex[90].

13 Note S13. Supplementary Datasets

Data	Location
Drug-Virus dataset	Andersen et al.[14], available for download at https://drugvirus.info/
Predictions from our matrix decomposition approach	Supplementary File 1
protein-protein interaction network (Gysi et al.)	Gysi et al.[38]
Drug target associations	Gysi et al.[38]
HuRI interactome (SwissProt only)	Supplementary File 2
Cheng et al. interactome (SwissProt only)	Supplementary File 3
Drug target associations (SwissProt only)	Supplementary File 4
Host Proteins (336 proteins - UniProt accession numbers)	Supplementary File 5
Host Proteins (336 proteins - Entrez IDs)	Supplementary File 6
DrugBank to ATC category map	Supplementary File 7
DrugBank entries with significant τ score in CMAP	Supplementary File 8
DrugBank entries with *in vitro* evidence	Supplementary File 9
DrugBank entries in Clinical Trials in December 1, 2020	Supplementary File 10
Predictions from our network medicine approach (on 2197 FDA approved drugs)	Supplementary File 11
COVID-19 signature for CMAP query (106 upregulated genes and 41 downregulated genes)	Table S2 in work by Ghandikota et al.[46]
Raw counts of RNAseq data expression data from 430 COVID-19 patients, and 54 controls[67]	https://www.ncbi.nlm.nih.gov/gds (GEO[91,92] accession number GSE152075[67])
Weights assigned to the host proteins by the kernel-based methods	Supplementary File 12

Table S7. Datasets and Supplementary Files:. This table summarises the datasets used throughout our experiments, as well as the results. When possible, we provide the data as a supplementary file available from Mendeley Data at http://dx.doi.org/10.17632/p7y5wmschg.1. References to the original sources are provided when redistribution rights are not granted.

References

1. Campillos, M., Kuhn, M., Gavin, A.-C., Jensen, L. J. & Bork, P. Drug target identification using side-effect similarity. *Science* **321**, 263–266 (2008).
2. Sirota, M. *et al.* Discovery and preclinical validation of drug indications using compendia of public gene expression data. *Sci Transl Med* **3**, 96ra77, DOI: 10.1126/scitranslmed.3001318 (2011).
3. Cami, A., Arnold, A., Manzi, S. & Reis, B. Predicting adverse drug events using pharmacological network models. *Sci. translational medicine* **3**, 114ra127–114ra127 (2011).
4. Lee, D. D. & Seung, H. S. Learning the parts of objects by non-negative matrix factorization. *Nature* **401**, 788–791 (1999).
5. Lee, D. D. & Seung, H. S. Algorithms for non-negative matrix factorization. In *Advances in neural information processing systems*, 556–562 (2001).
6. Dowden, H. & Munro, J. Trends in clinical success rates and therapeutic focus. *Nat. reviews. Drug discovery* **18**, 495 (2019).
7. Sosnina, E. A. *et al.* Recommender systems in antiviral drug discovery. *ACS omega* **5**, 15039–15051 (2020).
8. Bakal, G., Kilicoglu, H. & Kavuluru, R. Non-negative matrix factorization for drug repositioning: experiments with the repodb dataset. In *AMIA Annual Symposium Proceedings*, vol. 2019, 238 (American Medical Informatics Association, 2019).
9. Brown, A. S. & Patel, C. J. A standard database for drug repositioning. *Sci. data* **4**, 1–7 (2017).
10. Ceddia, G., Pinoli, P., Ceri, S. & Masseroli, M. Matrix factorization-based technique for drug repurposing predictions. *IEEE journal biomedical health informatics* **24**, 3162–3172 (2020).
11. Ding, C., Li, T., Peng, W. & Park, H. Orthogonal nonnegative matrix t-factorizations for clustering. In *Proceedings of the 12th ACM SIGKDD international conference on Knowledge discovery and data mining*, 126–135 (2006).

12. Li, T. & Ding, C. The relationships among various nonnegative matrix factorization methods for clustering. In *Sixth International Conference on Data Mining (ICDM'06)*, 362–371 (IEEE, 2006).

13. Tang, X. *et al.* Indicator regularized non-negative matrix factorization method-based drug repurposing for covid-19. *Front. Immunol.* **11**, 3824 (2021).

14. Andersen, P. I. *et al.* Discovery and development of safe-in-man broad-spectrum antiviral agents. *Int. J. Infect. Dis.* (2020).

15. Gayvert, K. M., Madhukar, N. S. & Elemento, O. A data-driven approach to predicting successes and failures of clinical trials. *Cell chemical biology* **23**, 1294–1301 (2016).

16. Cipolat, M. M. & Sprinz, E. Covid-19 pneumonia in an hiv-positive woman on antiretroviral therapy and undetectable viral load in porto alegre, brazil. *The Braz. J. Infect. Dis.* (2020).

17. Hall Jr, D. C. & Ji, H.-F. A search for medications to treat covid-19 via in silico molecular docking models of the sars-cov-2 spike glycoprotein and 3cl protease. *Travel. medicine infectious disease* 101646 (2020).

18. Gautret, P. *et al.* Hydroxychloroquine and azithromycin as a treatment of covid-19: results of an open-label non-randomized clinical trial. *Int. journal antimicrobial agents* 105949 (2020).

19. Kalil, A. C. Treating covid-19—off-label drug use, compassionate use, and randomized clinical trials during pandemics. *Jama* **323**, 1897–1898 (2020).

20. Gendrot, M. *et al.* Antimalarial artemisinin-based combination therapies (act) and covid-19 in africa: In vitro inhibition of sars-cov-2 replication by mefloquine-artesunate. *Int. J. Infect. Dis.* **99**, 437–440 (2020).

21. Zhavoronkov, A. Geroprotective and senoremediative strategies to reduce the comorbidity, infection rates, severity, and lethality in gerophilic and gerolavic infections. *Aging (Albany NY)* **12**, 6492 (2020).

22. Stebbing, J. *et al.* Covid-19: combining antiviral and anti-inflammatory treatments. *The Lancet Infect. Dis.* **20**, 400–402 (2020).

23. Laterre, P. F. *et al.* Association of interleukin 7 immunotherapy with lymphocyte counts among patients with severe coronavirus disease 2019 (covid-19). *JAMA network open* **3**, e2016485–e2016485 (2020).

24. Monneret, G. *et al.* Immune monitoring of interleukin-7 compassionate use in a critically ill covid-19 patient. *Cell. & molecular immunology* **17**, 1001–1003 (2020).

25. Who's solidarity clinical trial enters a new phase with three new candidate drugs. https://www.who.int/news/item/11-08-2021-who-s-solidarity-clinical-trial-enters-a-new-phase-with-three-new-candidate-drugs. Accessed: August 2021.

26. Hassanipour, S. *et al.* The efficacy and safety of favipiravir in treatment of covid-19: A systematic review and meta-analysis of clinical trials. *Sci. reports* **11**, 1–11 (2021).

27. Wang, M. *et al.* Remdesivir and chloroquine effectively inhibit the recently emerged novel coronavirus (2019-ncov) in vitro. *Cell research* **30**, 269–271 (2020).

28. Cai, Q. *et al.* Experimental treatment with favipiravir for covid-19: an open-label control study. *Engineering* (2020).

29. Du, Y.-X. & Chen, X.-P. Favipiravir: pharmacokinetics and concerns about clinical trials for 2019-ncov infection. *Clin. Pharmacol. & Ther.* (2020).

30. Wang, X. *et al.* The anti-influenza virus drug, arbidol is an efficient inhibitor of sars-cov-2 in vitro. *Cell Discov.* **6**, 1–5 (2020).

31. Omolo, C. A., Soni, N., Fasiku, V. O., Mackraj, I. & Govender, T. Update on therapeutic approaches and emerging therapies for sars-cov-2 virus. *Eur. J. Pharmacol.* **883**, 173348 (2020).

32. Cao, B. *et al.* A trial of lopinavir–ritonavir in adults hospitalized with severe covid-19. *New Engl. J. Medicine* (2020).

33. Consortium, W. S. T. Repurposed antiviral drugs for covid-19—interim who solidarity trial results. *New Engl. journal medicine* **384**, 497–511 (2021).

34. Grein, J. *et al.* Compassionate use of remdesivir for patients with severe covid-19. *New Engl. J. Medicine* **382**, 2327–2336 (2020).

35. Zhai, M. Z., Lye, C. T. & Kesselheim, A. S. Need for transparency and reliable evidence in emergency use authorizations for coronavirus disease 2019 (covid-19) therapies. *JAMA Intern. Medicine* (2020).

36. Réa-Neto, Á. *et al.* An open-label randomized controlled trial evaluating the efficacy of chloroquine/hydroxychloroquine in severe covid-19 patients. *Sci. reports* **11**, 1–10 (2021).

37. Galeano, D., Li, S., Gerstein, M. & Paccanaro, A. Predicting the frequencies of drug side effects. *Nat. Commun.* **11**, 1–14 (2020).

38. Gysi, D. M. *et al.* Network medicine framework for identifying drug-repurposing opportunities for COVID-19. *PNAS* **118**, DOI: 10.1073/pnas.2025581118 (2021).

39. Wishart, D. S. *et al.* DrugBank 5.0: a major update to the DrugBank database for 2018. *Nucleic Acids Res.* **46**, D1074–D1082, DOI: 10.1093/nar/gkx1037 (2018).

40. Cheng, F. *et al.* Network-based approach to prediction and population-based validation of in silico drug repurposing. *Nat. Commun.* **9**, 1–12, DOI: 10.1038/s41467-018-05116-5 (2018). Number: 1 Publisher: Nature Publishing Group.

41. Luck, K. *et al.* A reference map of the human binary protein interactome. *Nature* **580**, 402–408, DOI: 10.1038/s41586-020-2188-x (2020).

42. Consortium, T. U. UniProt: a worldwide hub of protein knowledge. *Nucleic Acids Res.* **47**, D506–D515, DOI: 10.1093/nar/gky1049 (2019).

43. Riva, L. *et al.* Discovery of SARS-CoV-2 antiviral drugs through large-scale compound repurposing. *Nature* 1–11, DOI: 10.1038/s41586-020-2577-1 (2020). Publisher: Nature Publishing Group.

44. Lamb, J. *et al.* The Connectivity Map: Using Gene-Expression Signatures to Connect Small Molecules, Genes, and Disease. *Science* (2006). Publisher: American Association for the Advancement of Science.

45. Subramanian, A. *et al.* A Next Generation Connectivity Map: L1000 Platform and the First 1,000,000 Profiles. *Cell* **171**, 1437–1452.e17, DOI: 10.1016/j.cell.2017.10.049 (2017).

46. Ghandikota, S., Sharma, M. & Jegga, A. G. Secondary analysis of transcriptomes of SARS-CoV-2 infection models to characterize COVID-19. *Patterns* **2**, 100247, DOI: https://doi.org/10.1016/j.patter.2021.100247 (2021).

47. Cao, M. *et al.* Going the Distance for Protein Function Prediction: A New Distance Metric for Protein Interaction Networks. *PLOS ONE* **8**, e76339, DOI: 10.1371/journal.pone.0076339 (2013). Publisher: Public Library of Science.

48. Guney, E., Menche, J., Vidal, M. & Barábasi, A.-L. Network-based in silico drug efficacy screening. *Nat. Commun.* **7**, 10331, DOI: 10.1038/ncomms10331 (2016).

49. Gordon, D. E. *et al.* A SARS-CoV-2 protein interaction map reveals targets for drug repurposing. *Nature* **583**, 459–468, DOI: 10.1038/s41586-020-2286-9 (2020).

50. Mitchell, A. L. *et al.* InterPro in 2019: improving coverage, classification and access to protein sequence annotations. *Nucleic Acids Res.* **47**, D351–D360, DOI: 10.1093/nar/gky1100 (2019).

51. Kokic, G. *et al.* Mechanism of SARS-CoV-2 polymerase stalling by remdesivir. *Nat. Commun.* **12**, 279, DOI: 10.1038/s41467-020-20542-0 (2021).

52. Naydenova, K. *et al.* Structure of the SARS-CoV-2 RNA-dependent RNA polymerase in the presence of favipiravir-RTP. *Proc. Natl. Acad. Sci.* **118**, DOI: 10.1073/pnas.2021946118 (2021). ISBN: 9782021946116 Publisher: National Academy of Sciences Section: Biological Sciences.

53. Uzunova, K., Filipova, E., Pavlova, V. & Vekov, T. Insights into antiviral mechanisms of remdesivir, lopinavir/ritonavir and chloroquine/hydroxychloroquine affecting the new SARS-CoV-2. *Biomed. & Pharmacother.* **131**, 110668, DOI: 10.1016/j.biopha.2020.110668 (2020).

54. Clososki, G. C. *et al.* Tenofovir Disoproxil Fumarate: New Chemical Developments and Encouraging in vitro Biological Results for SARS-CoV-2. *J. Braz. Chem. Soc.* 1552–1556 (2020).

55. Park, S.-J. *et al.* Antiviral Efficacies of FDA-Approved Drugs against SARS-CoV-2 Infection in Ferrets. *mBio* **11**, DOI: 10.1128/mBio.01114-20 (2020).

56. Parienti, J.-J. *et al.* Effect of Tenofovir Disoproxil Fumarate and Emtricitabine on nasopharyngeal SARS-CoV-2 viral load burden amongst outpatients with COVID-19: A pilot, randomized, open-label phase 2 trial. *EClinicalMedicine* **38**, 100993, DOI: 10.1016/j.eclinm.2021.100993 (2021).

57. García-Trejo, J. J., Ortega, R. & Zarco-Zavala, M. Putative Repurposing of Lamivudine, a Nucleoside/Nucleotide Analogue and Antiretroviral to Improve the Outcome of Cancer and COVID-19 Patients. *Front. Oncol.* **11**, 664794, DOI: 10.3389/fonc.2021.664794 (2021).

58. Zhou, Y. *et al.* In vitro efficacy of artemisinin-based treatments against SARS-CoV-2. *Sci. Reports* **11**, 14571, DOI: 10.1038/s41598-021-93361-y (2021).

59. Liu, S., Zheng, Q. & Wang, Z. Potential covalent drugs targeting the main protease of the SARS-CoV-2 coronavirus. *Bioinformatics* **36**, 3295–3298, DOI: 10.1093/bioinformatics/btaa224 (2020).

60. Hall, D. C. & Ji, H.-F. A search for medications to treat COVID-19 via in silico molecular docking models of the SARS-CoV-2 spike glycoprotein and 3CL protease. *Travel. Medicine Infect. Dis.* **35**, 101646, DOI: 10.1016/j.tmaid.2020.101646 (2020).

61. Yao, Y., Luo, Z. & Zhang, X. In silico evaluation of marine fish proteins as nutritional supplements for COVID-19 patients. *Food Funct.* **11**, 5565–5572, DOI: 10.1039/D0FO00530D (2020).

62. Martorana, A., Gentile, C. & Lauria, A. In Silico Insights into the SARS CoV-2 Main Protease Suggest NADH Endogenous Defences in the Control of the Pandemic Coronavirus Infection. *Viruses* **12**, 805, DOI: 10.3390/v12080805 (2020).

63. Mittra, I. *et al.* Resveratrol and Copper for treatment of severe COVID-19: an observational study (RESCU 002). *medRxiv* 2020.07.21.20151423, DOI: 10.1101/2020.07.21.20151423 (2020).

64. Barh, D. *et al.* Multi-omics-based identification of SARS-CoV-2 infection biology and candidate drugs against COVID-19. *Comput. Biol. Medicine* 104051, DOI: 10.1016/j.compbiomed.2020.104051 (2020).

65. Al-Motawa, M. S. *et al.* Vulnerabilities of the SARS-CoV-2 Virus to Proteotoxicity—Opportunity for Repurposed Chemotherapy of COVID-19 Infection. *Front. Pharmacol.* **11**, 1579, DOI: 10.3389/fphar.2020.585408 (2020).

66. Pathak, Y., Mishra, A., Choudhir, G., Kumar, A. & Tripathi, V. Rifampicin and Letermovir as potential repurposed drug candidate for COVID-19 treatment: insights from an in-silico study. *Pharmacol. reports: PR* **73**, 926–938, DOI: 10.1007/s43440-021-00228-0 (2021).

67. Lieberman, N. A. P. *et al.* In vivo antiviral host transcriptional response to SARS-CoV-2 by viral load, sex, and age. *PLOS Biol.* **18**, 1–17, DOI: 10.1371/journal.pbio.3000849 (2020). Publisher: Public Library of Science.

68. Kost-Alimova, M. *et al.* A High-Content Screen for Mucin-1-Reducing Compounds Identifies Fostamatinib as a Candidate for Rapid Repurposing for Acute Lung Injury. *Cell Reports Medicine* **1**, 100137, DOI: 10.1016/j.xcrm.2020.100137 (2020).

69. Hoepel, W. *et al.* High titers and low fucosylation of early human anti–SARS-CoV-2 IgG promote inflammation by alveolar macrophages. *Sci. Transl. Medicine* (2021). Publisher: American Association for the Advancement of Science.

70. Zhou, P. *et al.* A pneumonia outbreak associated with a new coronavirus of probable bat origin. *Nature* **579**, 270–273, DOI: 10.1038/s41586-020-2012-7 (2020).

71. Hoffmann, M. *et al.* SARS-CoV-2 Cell Entry Depends on ACE2 and TMPRSS2 and Is Blocked by a Clinically Proven Protease Inhibitor. *Cell* **181**, 271–280.e8, DOI: 10.1016/j.cell.2020.02.052 (2020). Publisher: Elsevier.

72. Wang, B. *et al.* In search of preventive strategies: novel high-cbd cannabis sativa extracts modulate ace2 expression in covid-19 gateway tissues. *Aging* **12**, 22425–22444, DOI: 10.18632/aging.202225 (2020).

73. Suba, Z. Prevention and therapy of COVID-19 via exogenous estrogen treatment for both male and female patients: Prevention and therapy of COVID-19. *J. Pharm. & Pharm. Sci.* **23**, 75–85, DOI: 10.18433/jpps31069 (2020).

74. Ragia, G. & Manolopoulos, V. G. Inhibition of SARS-CoV-2 entry through the ACE2/TMPRSS2 pathway: a promising approach for uncovering early COVID-19 drug therapies. *Eur. J. Clin. Pharmacol.* **76**, 1623–1630, DOI: 10.1007/s00228-020-02963-4 (2020).

75. Mauvais-Jarvis, F., Klein, S. L. & Levin, E. R. Estradiol, Progesterone, Immunomodulation, and COVID-19 Outcomes. *Endocrinology* **161**, DOI: 10.1210/endocr/bqaa127 (2020).

76. Wessels, I., Rolles, B. & Rink, L. The Potential Impact of Zinc Supplementation on COVID-19 Pathogenesis. *Front. Immunol.* **11**, 1712, DOI: 10.3389/fimmu.2020.01712 (2020).

77. Finzi, E. & Harrington, A. Zinc treatment of outpatient COVID-19: A retrospective review of 28 consecutive patients. *J. Med. Virol.* **93**, 2588–2590, DOI: 10.1002/jmv.26812 (2021). _eprint: https://onlinelibrary.wiley.com/doi/pdf/10.1002/jmv.26812.

78. Finzi, E. Treatment of SARS-CoV-2 with high dose oral zinc salts: A report on four patients. *Int. J. Infect. Dis.* **99**, 307–309, DOI: 10.1016/j.ijid.2020.06.006 (2020).

79. Rehman, A. u. & Qureshi, S. A. The role of primary and secondary bio-molecules in optical diagnosis of pandemic COVID-19 outbreak. *Photodiagnosis Photodyn. Ther.* **31**, 101953, DOI: 10.1016/j.pdpdt.2020.101953 (2020).

80. Radulesco, T., Lechien, J. R., Saussez, S., Hopkins, C. & Michel, J. Safety and Impact of Nasal Lavages During Viral Infections Such as SARS-CoV-2. *Ear, Nose & Throat J.* **100**, 188S–191S, DOI: 10.1177/0145561320950491 (2021). Publisher: SAGE Publications Inc.

81. Govind, R., Freitas, D. F. d., Pritchard, M., Hayes, R. D. & MacCabe, J. H. Clozapine treatment and risk of COVID-19 infection: retrospective cohort study. *The Br. J. Psychiatry* **219**, 368–374, DOI: 10.1192/bjp.2020.151 (2021). Publisher: Cambridge University Press.

82. Consensus statement on the use of clozapine during the COVID-19 pandemic – Journal of Psychiatry & Neuroscience.

83. Tamuzi, J. L. *et al.* Implications of COVID-19 in high burden countries for HIV/TB: A systematic review of evidence. *BMC Infect. Dis.* **20**, 744, DOI: 10.1186/s12879-020-05450-4 (2020).

84. Hull, M. W. & Montaner, J. S. G. Ritonavir-boosted protease inhibitors in HIV therapy. *Annals Medicine* **43**, 375–388, DOI: 10.3109/07853890.2011.572905 (2011). Publisher: Taylor & Francis _eprint: https://doi.org/10.3109/07853890.2011.572905.

85. Stadler, K. *et al.* Amiodarone alters late endosomes and inhibits SARS coronavirus infection at a post-endosomal level. *Am J Respir Cell Mol Biol* **39**, 142–149, DOI: 10.1165/rcmb.2007-0217OC (2008).

86. Castaldo, N. *et al.* Safety and Efficacy of Amiodarone in a Patient With COVID-19. *JACC Case Rep* **2**, 1307–1310, DOI: 10.1016/j.jaccas.2020.04.053 (2020).

87. Jordan, V. C. Fourteenth Gaddum Memorial Lecture. A current view of tamoxifen for the treatment and prevention of breast cancer. *Br J Pharmacol* **110**, 507–517 (1993).

88. Montoya, M. C. & Krysan, D. J. Repurposing Estrogen Receptor Antagonists for the Treatment of Infectious Disease. *mBio* **9**, e02272–18, DOI: 10.1128/mBio.02272-18 (2018).

89. Laurence, J., Cooke, H. & Sikder, S. K. Effect of tamoxifen on regulation of viral replication and human immunodeficiency virus (HIV) long terminal repeat-directed transcription in cells chronically infected with HIV-1. *Blood* **75**, 696–703 (1990).

90. Watashi, K. *et al.* Anti-hepatitis C virus activity of tamoxifen reveals the functional association of estrogen receptor with viral RNA polymerase NS5B. *J Biol Chem* **282**, 32765–32772, DOI: 10.1074/jbc.M704418200 (2007).

91. Edgar, R., Domrachev, M. & Lash, A. E. Gene Expression Omnibus: NCBI gene expression and hybridization array data repository. *Nucleic Acids Res* **30**, 207–210, DOI: 10.1093/nar/30.1.207 (2002).

92. Barrett, T. *et al.* NCBI GEO: archive for functional genomics data sets–update. *Nucleic Acids Res* **41**, D991–995, DOI: 10.1093/nar/gks1193 (2013).
